# Enhanced visible-light photoredox catalysis with rubicene-embedded polycyclic aromatic hydrocarbons (PAHs)

**DOI:** 10.1039/d6sc01097k

**Published:** 2026-06-10

**Authors:** Chunchun Mi, Liangzhuo Ma, Xuan Wen, Yuqi Hou, Xuelin Dao, Aidong Peng, Qinqin Shi, Hui Huang

**Affiliations:** a College of Materials Science and Opto-Electronic Technology, University of Chinese Academy of Sciences Beijing 100049 China shiqinqin@ucas.ac.cn; b School of Chemical Engineering and Technology, State Key Laboratory of Chemical Engineering and Low-Carbon Technology, Tianjin University Tianjin 300072 China

## Abstract

Visible-light-absorbing, metal-free photoredox catalysts are attractive because they are inexpensive and offer tunable redox potentials. However, the use of organic photocatalysts is often limited by their incompatibility with strongly acidic or basic media and by short excited-state lifetimes. Here, we report a series of rubicene-based organic photoredox catalysts that operate under visible-light irradiation and promote substitution and addition reactions. Mechanistic studies, including radical-trapping experiments and Stern–Volmer fluorescence-quenching analyses, support a single-electron-transfer (SET) pathway. Notably, the rubicene-based catalysts exhibit excellent operational stability and higher reactivity than commercial metal-free photocatalysts, indicating broad scope and high tolerance in metal-free photoredox transformations.

## Abstract 2

A series of rubicene-based, metal-free photoredox catalysts exhibit superior stability and reactivity, enabling visible light-driven reactions with broad scope as a sustainable and cost-effective alternative to transition-metal based systems.

## Graphical abstract

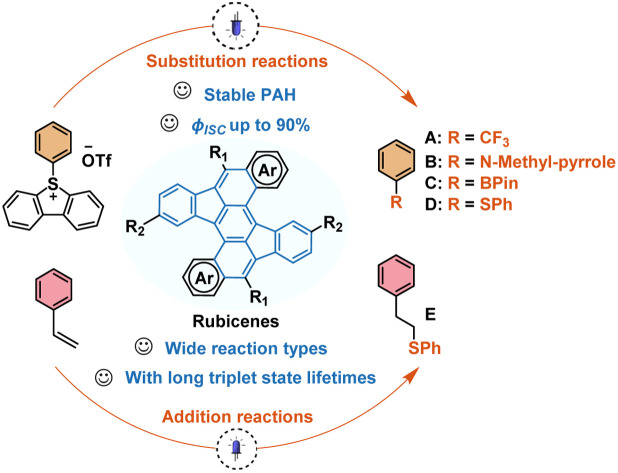

## Introduction

**Fig. 1:**
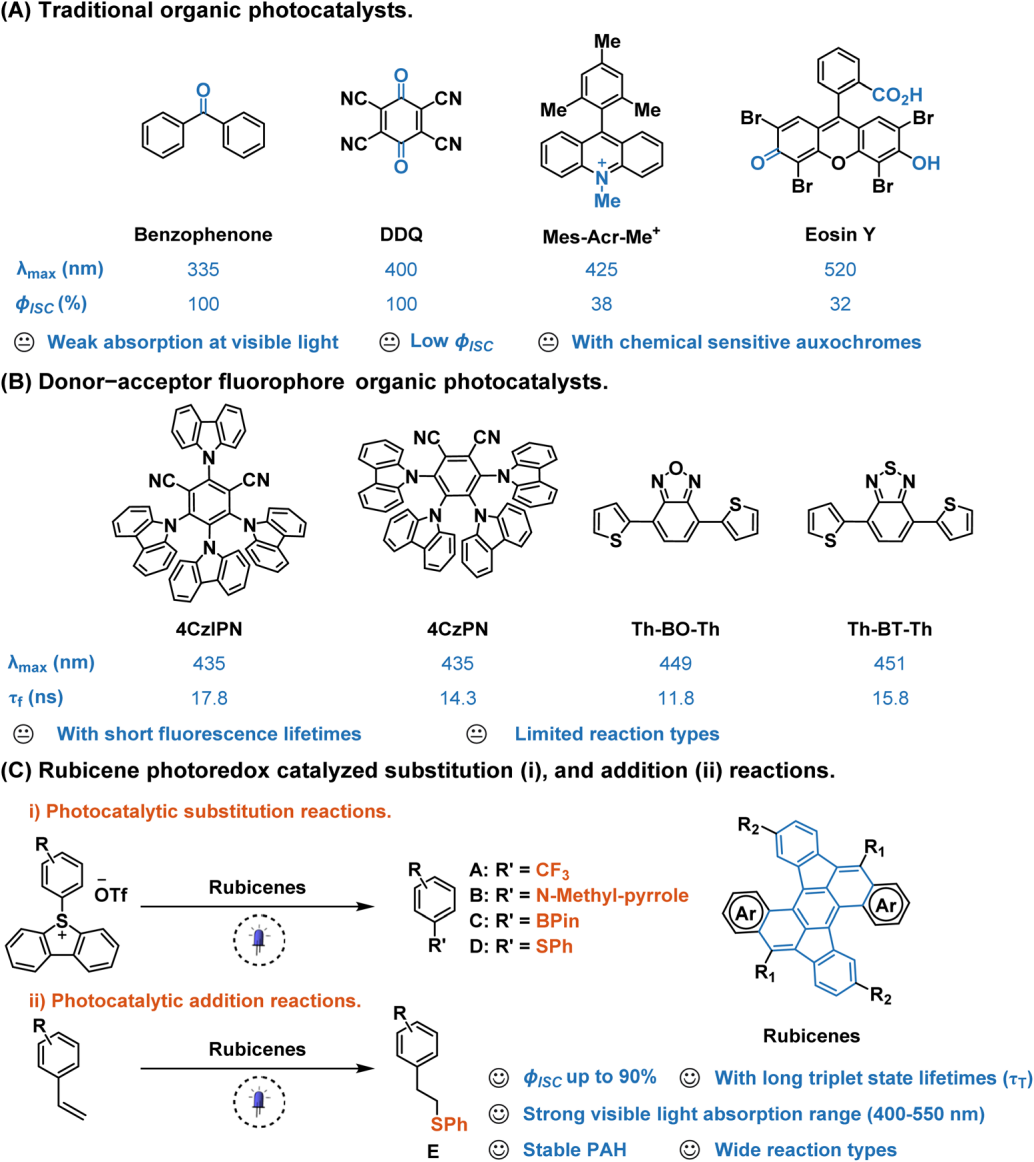
(A) Traditional organic photocatalysts. (B) Donor–acceptor fluorophore organic photocatalysts. (C) Rubicene photoredox catalyzed substitution (i) and addition (ii) reactions.

Over the past decade, visible-light-absorbing, metal-free photoredox catalysis has emerged as a powerful platform for organic synthesis owing to its mild and excellent sustainable reaction conditions.^1^ A variety of catalysts, including small molecular dyes^2^ and polymeric organic semiconductors,^3^ have been developed by virtue of suitable redox potentials and long-lived excited states (*τ*_f_ ≥ 1 ns). Among them, small molecular dyes have been extensively studied in a wide range of transformations, such as C(sp^3^)–C(sp^2^), C(sp^3^)–C(sp^3^), C(sp^2^)–C(sp^2^), C–Br, C–Cl, C–S, C–N and C–P bond-formation, as well as in alkene isomerization.^2*a*–*d*,2*f*,3*a*,4^ To function efficiently under visible light, such photocatalysts generally require both long-lived excited states and high chemical stability. For achieving extended excited-state lifetimes, most organic photoredox catalysts, including benzophenone, DDQ, acridinium and eosin Y (Fig. 1A), rely on the sensitive auxochromes such as carbonyl, ester, amide, halogen, and carboxyl groups^2*c*,*d*,3*a*,5^ to enhance intersystem crossing (*τ*_T_ ≥ 1 µs) and tune their absorption profiles. However, carbonyl, ester and carboxyl groups are susceptible to degradation under strong bases (*e.g.*, Grignard reagents and sodium hydroxide),^5,6^ whereas amides are unstable under strongly acidic conditions (*e.g.*, hydrochloric acid).^7^ To simultaneously achieve visible-light absorption and excellent chemical robustness, donor–acceptor (D–A) type molecular dyads, such as 4CzPN, 4CzIPN, Th-BO-Th, and Th-BT-Th (Fig. 1B), have been developed as efficient metal-free photoredox catalysts.^2*a*^ Nevertheless, their narrow bandgaps tend to reduce radiative decay rates and shorten fluorescence lifetimes according to the energy-gap law.^8^ Thus, there remains a clear need for the development of organic photocatalysts that combine long-lived excited states with high chemical stability under diverse reaction conditions.

Recently, Wang reported rubicene null aggregates that exhibit ultrahigh triplet yield (192%).^9^ Shi and co-workers reported a series of chalcogen-doped rubicene polycyclic aromatic hydrocarbons (PAHs) that exhibit high intersystem-crossing quantum yields (*ϕ*_ISC_ up to 90%), strong absorption in the visible region (400–550 nm) and tunable photoredox potentials (Fig. 1C).^10^ Importantly, the exceptionally chemically and thermally stable PAH frameworks are expected to tolerate harsh reaction conditions and thereby offer broad synthetic utility. Motivated by these features, we hypothesized that rubicene scaffolds could serve as promising platforms for photocatalytic reactions. Here, we develop a family of rubicene-based photocatalysts that support a broad range of visible-light-driven transformations, including trifluoromethylation, heteroarylation, borylation, thioetherification and hydromethylthiolation (Fig. 1C). Notably, these catalysts display superior efficacy and excellent tolerance under acidic and basic conditions, at elevated temperatures and in common organic solvents, highlighting the robustness of the catalytic system. In addition, radical-trapping experiments and Stern–Volmer fluorescence-quenching analyses support a single-electron-transfer (SET) process.

## Results and discussion

### Optimization of reaction conditions

Considering the advantages of biphenylsulfonium salts, such as high selectivity, high reduction potential, and easy accessibility,^11^ they were selected as nucleophiles in the challenging trifluoromethylation reaction. Trifluoromethyl groups in drug molecules are associated with enhanced blood–brain barrier penetration,^12^ increased metabolic stability and improved selectivity.^13^ Thus, we first examined the photocatalytic trifluoromethylation of a biphenylsulfonium salt (Table 1). The reaction was carried out using [CuCF_3_], generated from CuSCN, CsF and TMSCF_3_ as the trifluoromethylating reagent *in situ*, with rubicene as the photoredox catalyst in acetone:DMF (8 : 1) under 467 nm irradiation at room temperature for 13 h. Under these conditions, 4-(trifluoromethyl)-1,1′-biphenyl (A1) was obtained in 61% yield (entry 1). In view of the relatively low *ϕ*_ISC_ of rubicene (Fig. 3A), we next screened rubicene derivatives with higher *ϕ*_ISC_ values, including Ben-rubicene, O-rubicene, S-rubicene and Se-rubicene (entries 2–5). Notably, the use of S-rubicene afforded A1 in 87% yield (entry 4). As expected, no product was detected when the reaction was conducted in the absence of a photocatalyst or in the dark (entries 6–7). To exclude any contribution from trace transition metals, sublimed S-rubicene was evaluated (entry 8) to generate A1 in 84% yield, indicating that the effect of trace metal impurities is negligible.

**Table 1:** Optimization of the trifluoromethylation of biphenylsulfonium salts^a^

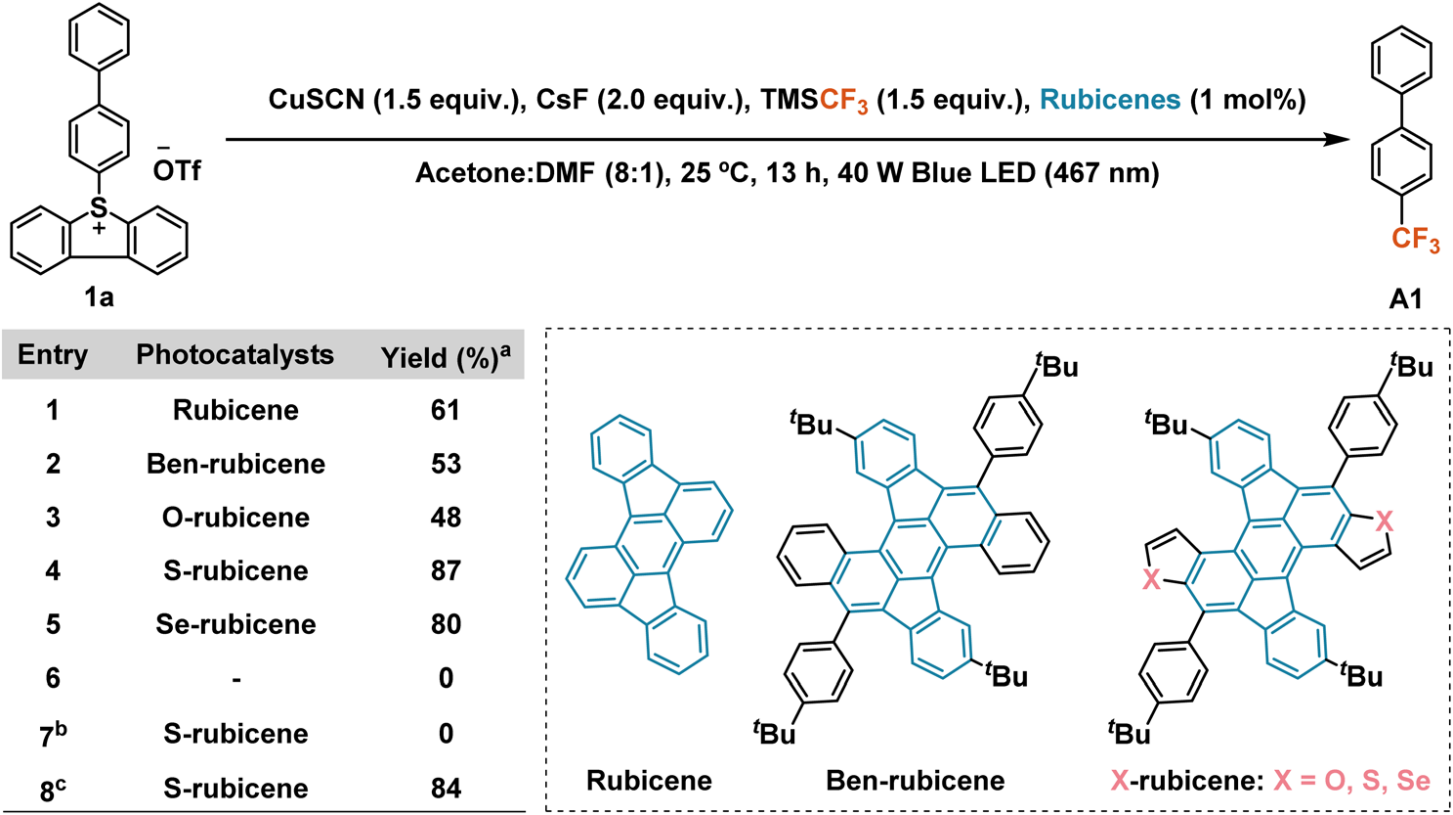

a Unless otherwise noted, a mixture of CuSCN (0.15 mmol, 1.5 equiv.), CsF (0.2 mmol, 2.0 equiv.), and TMSCF_3_ (0.15 mmol, 1.5 equiv.) in DMF (0.5 mL), biphenylsulfonium salt (1a) (0.10 mmol, 1.0 equiv.), and rubicenes (1 mol%) in acetone (4.0 mL) was irradiated with a blue LED (467 nm) lamp at 25 °C for 13 h. Isolated yields are given.

b In the dark.

c S-rubicene is purified by sublimation.

### Mechanistic investigations

**Fig. 2:**
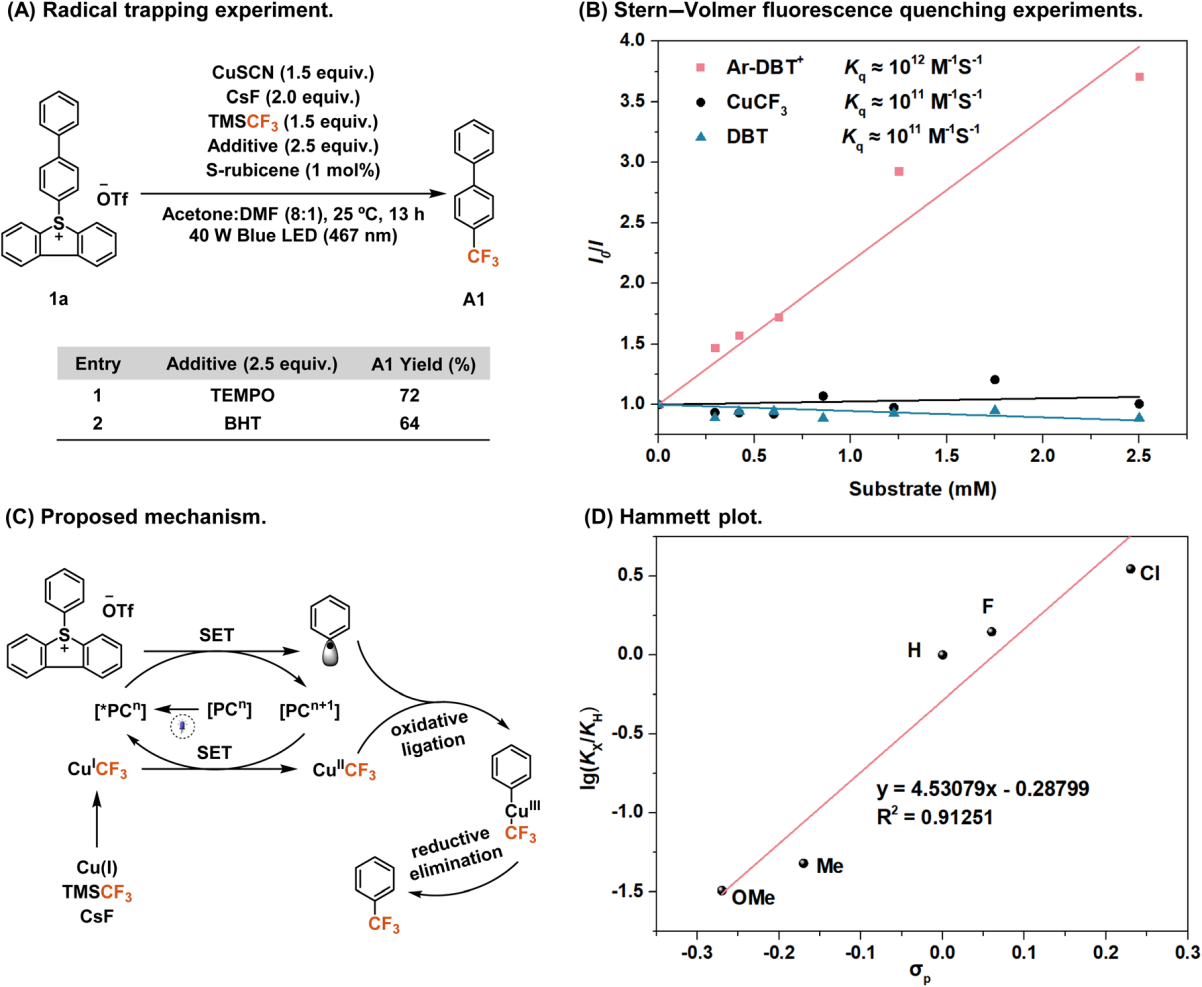
(A) The radical trapping experiments in the presence of various radical inhibitors. Conditions: a solution of CuSCN (0.15 mmol, 1.5 equiv.), CsF (0.2 mmol, 2.0 equiv.), and TMSCF_3_ (0.15 mmol, 1.5 equiv.) in DMF (0.5 mL), 1a (1.0 equiv.), S-rubicene (1 mol%), additive (2.5 equiv.), acetone (0.025 M), 25 °C, Kessil 40 W blue LED (467 nm), 13 h. (B) Stern–Volmer plot for S-rubicene in DMF/acetone (v/v: 1 : 1) (100 µM) with varying [CuCF_3_], aryl sulfonium salt and dibenzothiophene (DBT). (C) Proposed mechanism of the S-rubicene photoredox catalyzed trifluoromethylation of biphenylsulfonium salts. (D) Hammett plot.

To elucidate the role of the rubicene photocatalysts, we performed a series of mechanistic studies. The addition of 2,2,6,6-(tetramethylpiperidin-1-yl)oxyl (TEMPO) or 2,6-di-*tert*-butyl-4-methylphenol (BHT) resulted in formation of A1 in 74% and 65% yield, respectively (entries 1–2, Fig. 2A), only slightly suppressing the photocatalytic reaction. This modest inhibition suggests that copper intermediates trap radicals more efficiently than the added radical scavengers.^14^ To further probe the mechanism, Stern–Volmer fluorescence-quenching experiments were carried out to assess electron transfer between S-rubicene and either [CuCF_3_] or the aryl sulfonium salt (SI Fig. S1 and S2). The emission of S-rubicene was efficiently quenched by aryl sulfonium salts, whereas dibenzothiophene (DBT) and [CuCF_3_] induced only weak quenching (Fig. 2B), indicating a SET process between S-rubicene and the aryl sulfonium salt. These observations are consistent with the radical-trapping experiments. Furthermore, we examined the relative rates of reactions involving aryl sulfonium salts bearing *para*-substituted benzene groups (MeO, Me, H, F and Cl; Fig. 2D). The positive Hammett *ρ* value (+4.5) indicates that the reduction of electron density at the sulfonium salt reaction center in the presence of electron-withdrawing groups (EWGs) influences the kinetics of the photocatalytic mechanism. Specifically, EWGs are expected to further diminish the electron density at the reaction center, thereby facilitating electron transfer (ET) from the S-rubicene photocatalyst. Consequently, it is plausible that ET from the catalyst to the substrate is involved in the rate-determining step of the reaction. On the basis of these results, we propose the catalytic cycle shown in Fig. 2C. First, the ground-state photocatalyst [PC^*n*^] is excited by blue light to give [*PC^*n*^]. This excited state undergoes SET with the aryl sulfonium salt to afford [PC^*n*+1^] and the corresponding aryl radical. Then, the oxidized photocatalyst [PC^*n*+1^] performs SET with [Cu^I^CF_3_], regenerating [PC^*n*^] and forming [Cu^II^CF_3_], which captures the aryl radical to generate a PhCu^III^CF_3_ intermediate. Finally, reductive elimination from this intermediate furnishes the trifluoromethylated aryl product.

**Fig. 3:**
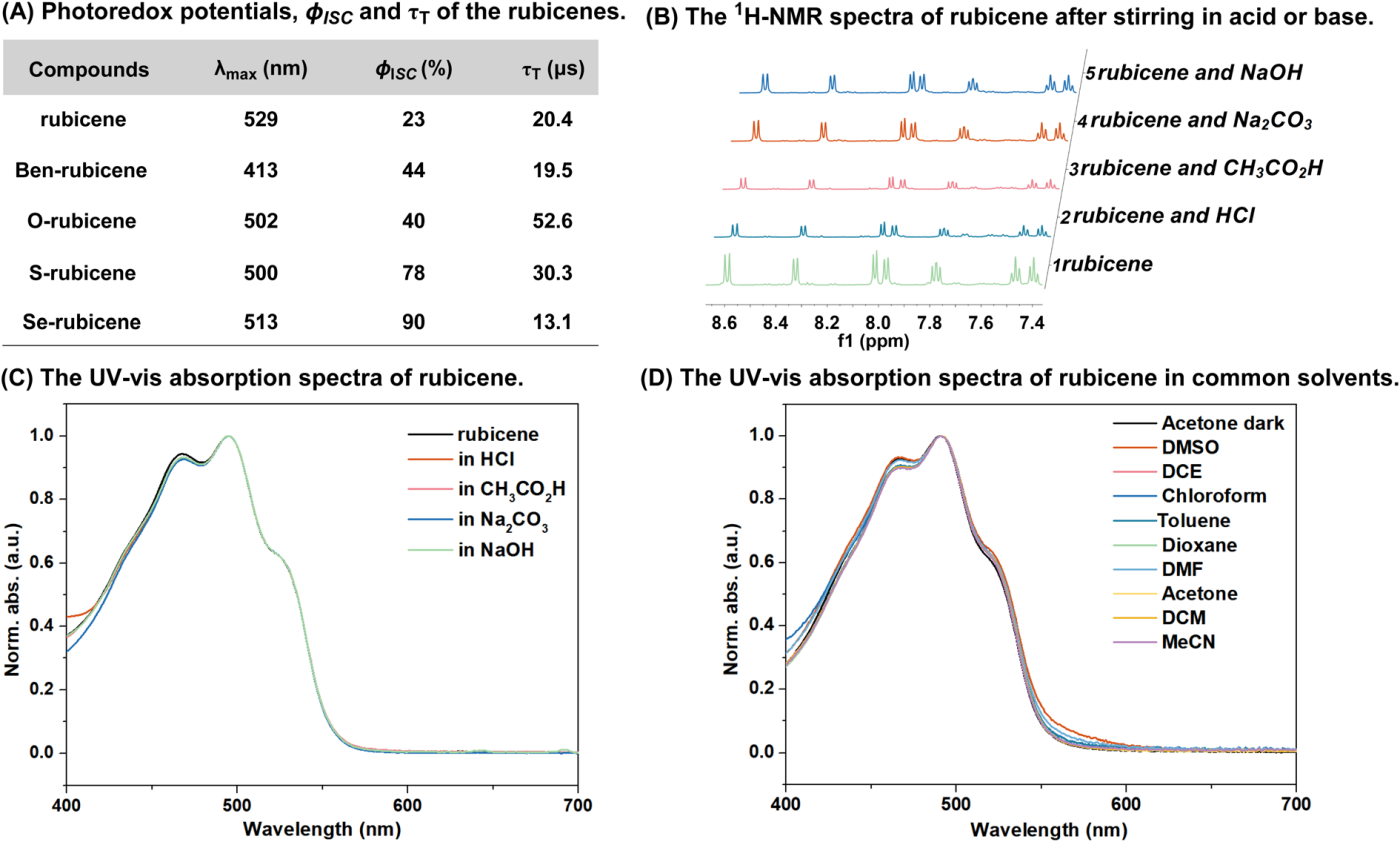
(A) Local absorbance maximum, *ϕ*_ISC_ and *τ*_T_ of the rubicenes: rubicene, Ben-rubicene, O-rubicene, S-rubicene, Se-rubicene. (B) ^1^H-NMR spectra of rubicene after stirring in acid or base (0.25 M) in acetone for 6 h. (C) The UV-vis absorption spectra of rubicene after stirring in acid or base (0.25 M) in acetone for 6 h. (D) The UV-vis absorption spectra of rubicene solution after blue light irradiation.

To gain further insight into the role of rubicene, we examined the triplet-state lifetimes (*τ*_T_) of rubicene derivatives in chloroform solution by transient absorption spectroscopy using a tunable-wavelength nanosecond laser. The triplet lifetimes of rubicene, Ben-rubicene, O-rubicene, S-rubicene and Se-rubicene were determined to be 20.4, 19.5, 52.6, 30.3 and 13.1 µs, respectively, values that are consistent with high catalytic efficiency in photoredox processes. The fluorescence lifetimes range from 1.7 ns to 4.9 ns. Notably, since the photocatalysts exhibit fluorescence lifetimes greater than 1 ns, their singlet excited states may also participate in the photoinduced electron transfer (PET) process.^2*f*^ The high intersystem-crossing quantum yields (*ϕ*_ISC_) of these rubicenes, ranging from 23% to 90%, indicate that the T_1_ excited state readily engages in bimolecular reactions with substrates and can efficiently mediate both energy transfer (EnT) and electron transfer (ET).^2*f*^ In addition, the excited states of rubicenes display strong oxidizing (*E*_1/2_(*P/*P*^−^) = +0.79 to +1.75 V) and reducing (*E*_1/2_(*P*^+^/*P) = −1.58 to −2.34 V) abilities under visible-light excitation. These redox potentials are comparable to or exceed those of many metal complexes and organic dyes,^2*a*,*f*^ suggesting broad substrate compatibility (Fig. 3A). To assess the chemical stability of rubicenes, 1 mol% of rubicene was stirred in strongly acidic or basic media for 6 h. The ^1^H-NMR and UV-vis absorption spectra showed negligible changes (Fig. 3B and C). It is worth pointing out that the absorption spectra recorded before and after treatment showed no decrease in intensity (Fig. S4), further indicating excellent chemical robustness. Furthermore, rubicene solutions were irradiated with blue light for 6 h in common organic solvents including DMSO, DCE and chloroform (Fig. 3D). The UV-vis spectra remained essentially unchanged, demonstrating that rubicenes are photostable under visible-light irradiation in a range of solvents.

### Substrate scope

**Fig. 4:**
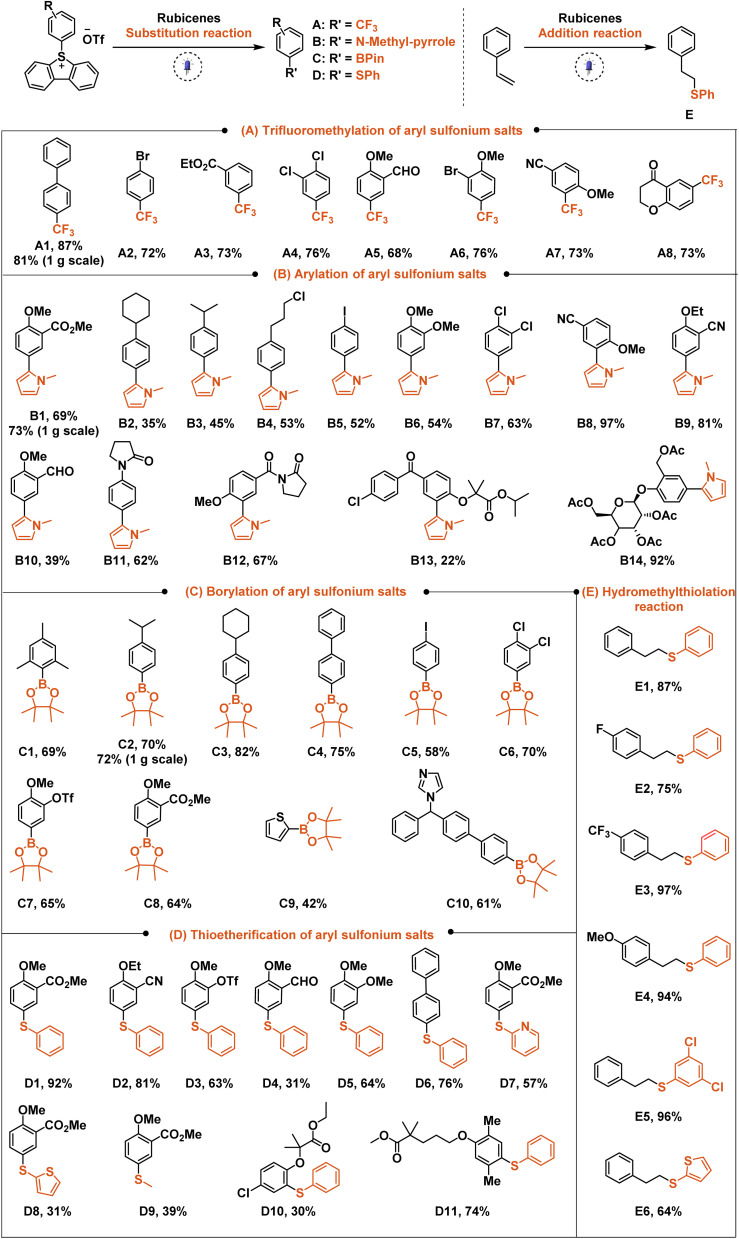
The substrate scope. (A) Trifluoromethylation of aryl sulfonium salts. Reaction conditions: CuSCN (1.5 equiv.), CsF (2.0 equiv.), TMSCF_3_ (1.5 equiv.) in DMF (0.3 M) for 30 min, aryl sulfonium salts (1.0 equiv.), S-rubicene (1 mol%), acetone (0.025 M), Kessil 40 W blue LED (467 nm) lamp, 25 °C, N_2_, 12 h. (B) Arylation of aryl sulfonium salts. Reaction conditions: aryl sulfonium salts (1.0 equiv.), *N*-methyl pyrrole (20.0 equiv.), S-rubicene (1 mol%), KOAc (5.0 equiv.), DMSO (0.2 M), Kessil 40 W blue LED (440 nm) lamp, 25 °C, N_2_, 35 h. (C) Borylation of aryl sulfonium salts. Reaction conditions: aryl sulfonium salts (1.0 equiv.), B_2_Pin_2_ (5.0 equiv.), rubicene (2 mol%), pyridine (5.0 equiv.), acetone (0.25 M), Kessil 40 W blue LED (427 nm) lamp, 25 °C, N_2_, 35 h. (D) Thioetherification of aryl sulfonium salts. Reaction conditions: aryl sulfonium salts (1.0 equiv.), RSSR (5.0 equiv.), S-rubicene (2 mol%), pyridine (5.0 equiv.), acetone (0.5 M), Kessil 40 W blue LED (440 nm) lamp, 25 °C, N_2_, 25 h. (E) Hydromethylthiolation reaction. Reaction conditions: styrene (1.0 equiv.), RSSR (5.0 equiv.), Se-rubicene (1 mol%), 2,6-dimethyl-1,4-dihydro-pyridine-3,5-dicarboxylic acid di-*tert*-butyl ester (Hantzsch ester) (2.0 equiv.), MeCN (0.1 M), Kessil 40 W blue LED (440) lamp, 25 °C, N_2_, 25 h. Isolated yields are shown.

With optimized reaction conditions in hand, we next examined the trifluoromethylation of various substrates (Fig. 4A). Phenylsulfonium salts bearing halogen (A2, A4 and A6), ester (A3), aldehyde (A5), alkoxy (A5, A6 and A7), cyano (A7) and carbonyl (A8) substituents were well tolerated, affording the desired products in ≥68% yield. Notably, *para*-bromo-, 3,4-dichloro- and 3-bromo-4-methoxy-substituted benzene sulfonium salts gave the corresponding trifluoromethylated products A2, A4 and A6 in 72%, 76% and 76% yields, respectively, providing useful handles for further derivatization. To demonstrate the versatility of the rubicene-based photoredox catalyst, we next investigated a series of additional transformations (Fig. 4B–E). N-methylpyrroles are key motifs in many biomolecules, pharmaceuticals,^15^ and conducting copolymers.^16^ The optimized conditions (SI Table S2) were compatible with halogen (B4, B5, B7 and B13), ester (B1 and B13), aldehyde (B10), alkoxy (B1, B6, B8, B9, B10, B12, B13 and B14), cyano (B8 and B9) and amide (B11 and B12) substituents on phenylsulfonium salts, delivering the desired products in 22–97% yield. Late-stage functionalization of bioactive molecules B13 and B14 furnished the corresponding products in 22% and 92% yield, respectively, illustrating that the photocatalytic protocol can be applied to structurally complex substrates. Organoboron compounds, valued for their low toxicity, mild reaction conditions and broad synthetic utility,^17^ have attracted considerable attention. Here, we report a transition-metal-free borylation of aryl sulfonium salts using rubicene as a photoredox catalyst (Fig. 4C, C1–C10). *Para*-iodo-, 3,4-dichloro- and 3-trifluoromethanesulfonate-substituted benzene sulfonium salts afforded the corresponding borylated products C5, C6 and C7 in 58%, 70% and 65% yield, respectively, again providing opportunities for further downstream derivatization. We then explored thioetherification (Fig. 4D), a widely used transformation in synthetic chemistry.^18^ Phenylsulfonium salts bearing ester (D1, D7 and D8–D11), cyano (D2), trifluoromethanesulfonate (D3), aldehyde (D4), halogen (D10) and alkoxy (D1–D5 and D7–D11) groups were well tolerated, affording thioether products in 30–92% yield. The method was also suitable for the synthesis of heteroaromatic thioethers (D7 and D8) and an alkyl thioether (D9), which were obtained in 57%, 31% and 39% yield, respectively. More importantly, late-stage functionalization of bioactive molecules was likewise achieved, affording thioether products D10 and D11 in 30% and 74% yield, respectively. Furthermore, we demonstrated the applicability of this platform to hydromethylthiolation using styrenes as substrates (Fig. 4E). The reaction tolerated halogen (E2, E3 and E5) and alkoxy (E4) substituents, delivering the corresponding products in 75–97% yield. The method also enabled the synthesis of a heteroaromatic thioether (E6) in 64% yield. Finally, gram-scale reactions were conducted to obtain products A1, B1 and C2. Under optimal conditions with slightly longer reaction times, products A1, B1 and C2 were obtained in 81%, 73% and 72% yields, respectively (Fig. 4), which are comparable to the yields (87%, 69% and 70%) achieved under small-scale conditions.

**Fig. 5:**
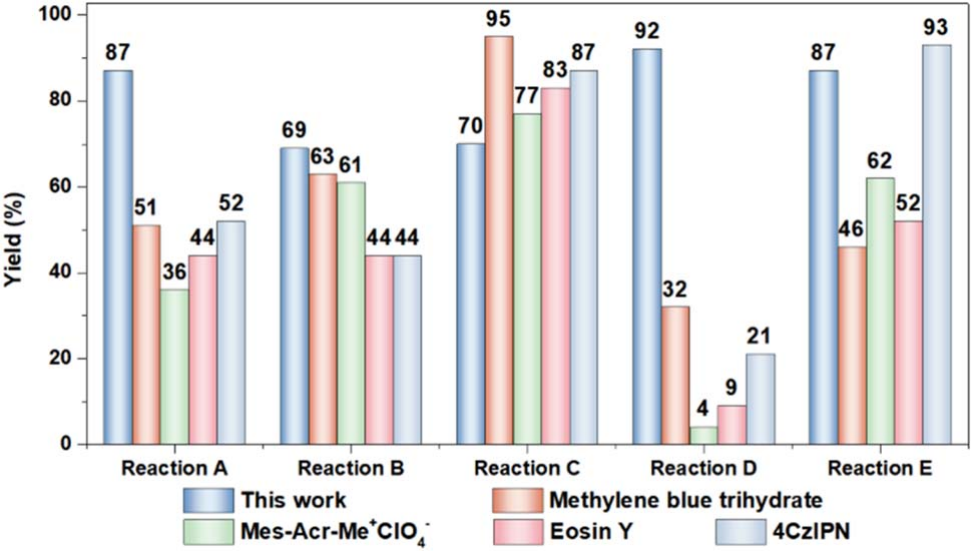
Comparison between the rubicene organic catalysts and other organic catalysts. Isolated yields are given.

We next benchmarked the catalytic performance of the rubicene-based photocatalyst against commercial organic photocatalysts in the A, B, C, D and E reactions (Fig. 5). The results show that in reaction D, using S-rubicene as the photocatalyst afforded the desired product in 92% yield, whereas the yields with commercial photocatalysts were all below 32%. Similarly, in reaction A, S-rubicene gave the desired product in 87% yield, while the yields with commercial photocatalysts were all below 52%. Furthermore, across all five reactions (A–E), the rubicene-based photocatalysts consistently exhibited high catalytic efficiency, whereas the commercial photocatalysts showed high efficiency only in selected reactions. Together, these comparisons indicate that rubicene-based photocatalysts offer a robust and versatile choice for synthetic applications.

## Conclusions

In summary, we have developed a class of rubicene-based photocatalysts that enable highly efficient photoredox transformations, including trifluoromethylation, heteroarylation, borylation, thioetherification of aryl sulfonium salts, and hydromethylthiolation. These reactions proceed under visible light and benefit from the exceptional chemical stability, strong oxidizing and reducing abilities, long excited-state lifetimes and high triplet quantum yields of the rubicene scaffolds. The methods show broad substrate scope and high functional-group tolerance and are applicable to the late-stage modification of structurally complex bioactive molecules. Radical-trapping experiments and Stern–Volmer fluorescence-quenching analyses support a SET mechanism. The combination of robustness, tunable redox properties and long-lived excited states positions rubicene as promising photocatalysts for scalable and more sustainable chemical manufacturing.

## Author contributions

Q.S. conceived the idea and supervised the projects. C.M. conducted the research. C.M., L.M. and X.W. performed the experiments. Y.H. performed the spectroscopic tests. C.M., H.H., A.P. and Q.S. wrote the manuscript.

## Conflicts of interest

There is no conflict of interest to report.

## Supplementary Material

SC-OLF-D6SC01097K-s001

## Data Availability

All data needed to evaluate the conclusions in the paper are present in the paper and/or the supplementary information (SI). Supplementary information: ^1^H, ^13^C, and ^19^F NMR spectra, HRMS data, synthetic procedures, and optimization and mechanistic studies. See DOI: https://doi.org/10.1039/d6sc01097k.
