# Supplementary material for: Enhanced visible-light photoredox catalysis with rubicene-embedded polycyclic aromatic hydrocarbons (PAHs)
Source: Chem Sci. 2026 Jun 10;17(31):14963–70. doi: 10.1039/d6sc01097k (PMC13289785; doi:10.1039/d6sc01097k)
Supplement: Supplementary file 1 [file SC-017-D6SC01097K-s001.pdf]

Supplementary Information (SI) for Chemical Science.
This journal is © The Royal Society of Chemistry 2026

Supplementary information

Enhanced Visible-Light Photoredox Catalysis with Rubicene-Embedded Polycyclic Aromatic Hydrocarbons (PAHs)

Chunchun Mi,[a†] Liangzhuo Ma,[a†] Xuan Wen,[a] Yuqi Hou,[a] Xuelin Dao,[a] Aidong Peng,[a] Qinqin Shi,*[a] and Hui Huang[b]

[a] College of Materials Science and Opto-Electronic Technology, University of Chinese Academy of Sciences, Beijing 100049

[b] School of Chemical Engineering and Technology, State Key Laboratory of Chemical Engineering and Low-Carbon Technology, Tianjin University, Tianjin 300072

[†]These authors contributed equally to this work.

*Corresponding Author: shiqinqin@ucas.ac.cn

Contents

I. Measurements and characterization....3
II. Materials....3
II.1. Materials....3
II.2. Preparation of substrates....4
II.2.1. The synthesis of aryl sulfonium salts....4
II.2.2. Substrate scope....5
III. Optimization studies....22
IV. Mechanistic studies....25
IV.1. Fluorescence Quenching Experiments....25
V. Materials characterization....26
VI. NMR spectra....29
References....92

I. Measurements and characterization

Nuclear magnetic resonance (NMR) spectra were obtained on a JNM-ECZ 500 MHz nuclear magnetic resonance spectrometer. Chemical shifts are given in parts per million (δ) downfield from tetramethylsilane using the residual solvent signal $CDCl_3$ (7.26 ppm) or $C_2Cl_4D_2$ (6.00 ppm), or d^3-acetonitrile (1.94 ppm) as internal standard. ^{1}H NMR information is given in the following format: multiplicity (s, singlet; d, doublet; t, triplet; m, multiplet), coupling constant (s) (J) in Hertz (Hz), number of protons. The prefix app is occasionally applied when the true signal multiplicity is unresolved. ^{13}C NMR spectra were reported in ppm (δ) relative to residual $CDCl_3$ (77.00 ppm), or $C_2Cl_4D_2$ (73.78 ppm) or d^3-acetonitrile (1.32 ppm). Electrospray ionization mass spectrometry (ESI-MS) was obtained on a Q Exactive Focus LCMS (Thermo Scientific Company, USA). Gas-chromatographic mass spectrometry (GC-MS) was tested by SHIMADZU GCMS-QP2020 NX, which was using helium as carrier gas and using anthracene as standard substance. Electron ionization mass spectrometry (EI-MS) was obtained on a Exactive-GC (Thermo Scientific Company, USA). UV-Vis absorption spectra were measured on a Cary 60 UV-vis Spectrophotometer. The fluorescence spectra and fluorescence quantum yields (PLQY) of the materials in diluted chloroform solution were measured with an Edinburgh FLS1000 transient fluorescence spectrometer. All film samples were spin-cast on quartz glass.

II. Materials

II.1. Materials

All reactions were carried out in flame-dried glassware under nitrogen atmosphere with the exclusion of air and moisture using standard Schlenk techniques. The solvents N,N-dimethylformamide (**DMF**), acetone, dimethyl sulfoxide (**DMSO**), 1,2-dichloroethane (**DCE**), chloroform, toluene, dioxane, dichloromethane (**DCM**) and acetonitrile (**MeCN**) were purified by FLEANO solvent purification systems (FL-MD-5). All reactions were stirred using Teflon-coated magnetic stir bars. Elevated temperatures were maintained using Thermostat-controlled silicone oil baths. Organic solutions were concentrated using a rotary evaporator with a diaphragm vacuum pump. The analytical TLC was performed on silica gel GF254 plates. The TLC plates were visualized by ultraviolet light (λ = 254 nm). Purification of products was accomplished by column chromatography on silica gel.

The compounds N-methyl pyrrole, bis(pinacolato)diboron (**B_2Pin_2**), diphenyl disulfide (**PhSSPh**), 2,2'-dithiodipyridine, 2-thienyl disulfide, 4-fluorostyrene, 4-(trifluoromethyl)styrene, methylene blue trihydrate, 4CzIPN and 4-methoxystyrene were purchased from Bide Pharmatech Co., Ltd. The dimethyl disulfide and butyl acetate (**BuOAc**) were purchased from Macklin Biochemical Technology Co., Ltd. The potassium acetate, cuprous thiocyanate and CsF were purchased from Energy-Chemical. The pyridine and styrene were purchased from Beijing Inno Chem Science & Technology Co., Ltd. The (trifluoromethyl)trimethylsilane (**$TMSCF_3$**) was purchased from Beijing Wokai Biotechnology Co., Ltd. The 2,6-dimethyl-1,4-dihydro-pyridine-3,5-dicarboxylic acid di-tert-butyl ester (**Hantzsch esters**) was purchased from Shanghai Haohong Scientific Co., Ltd. The 3,3',5,5'-tetrachlorodiphenyl disulfide, 9-mesityl-10-methylacridinium perchlorate (**Mes-Acr-$Me^+ClO_4^-$**), solvent red 43 (**Eosin Y**) and lithium bis(trifluoromethanesulphonyl)imide (**LiTFSI**) were purchased from Adamas-Beta Co., Ltd. The P($g_4$2T-TT) was purchased from Organtec Ltd. 5-(4-methoxy-3-(methoxycarbonyl)phenyl)-5*H*-dibenzo[*b,d*]thiophen-5-ium trifluoromethanesulfonate, 5-(4-methoxy-3-(((trifluoromethyl)sulfonyl)oxy)phenyl)-5*H*-dibenzo[*b,d*]thiophen-5-ium trifluoromethanesulfonate, 5-(4-isopropylphenyl)-5*H*-dibenzo[*b,d*]thiophen-5-ium trifluoromethanesulfonate, 5-(5-cyano-2-methoxyphenyl)-5*H*-dibenzo[*b,d*]thiophen-5-ium trifluoromethanesulfonate, 5-(3-(acetoxymethyl)-4-

(((2*S*,3*R*,4*R*,6*R*)-3,4,5-triacetoxy-6-(acetoxymethyl)tetrahydro-2*H*-pyran-2-yl)oxy)phenyl)-5*H*-dibenzo[*b,d*]thiophen-5-ium trifluoromethanesulfonate, 5-(4-(2-oxopyrrolidin-1-yl)phenyl)-5*H*-dibenzo[*b,d*]thiophen-5-ium trifluoromethanesulfonate, 5-(thiophen-2-yl)-5*H*-dibenzo[*b,d*]thiophen-5-ium trifluoromethanesulfonate, 5-(4-oxochroman-6-yl)-5*H*-dibenzo[*b,d*]thiophen-5-ium trifluoromethanesulfonate and 5-(3-bromo-4-methoxyphenyl)-5*H*-dibenzo[*b,d*]thiophen-5-ium trifluoromethanesulfonate were synthesized as literature reported;[1] 5-(5-chloro-2-((1-ethoxy-2-methyl-1-oxopropan-2-yl)oxy)phenyl)-5*H*-dibenzo[*b,d*]thiophen-5-ium trifluoromethanesulfonate, 5-([1,1'-biphenyl]-4-yl)-5*H*-dibenzo[*b,d*]thiophen-5-ium trifluoromethanesulfonate, 3,7-di-tert-butyl-5-(3-formyl-4-methoxyphenyl)-5*H*-dibenzo[*b,d*]thiophen-5-ium trifluoromethanesulfonate, 5-(3,4-dichlorophenyl)-5*H*-dibenzo[*b,d*]thiophen-5-ium trifluoromethanesulfonate, 5-(5-(4-chlorobenzoyl)-2-((1-isopropoxy-2-methyl-1-oxopropan-2-yl)oxy)phenyl)-5*H*-dibenzo[*b,d*]thiophen-5-ium trifluoromethanesulfonate, 5-(4-iodophenyl)-5*H*-dibenzo[*b,d*]thiophen-5-ium trifluoromethanesulfonate, 3,7-di-tert-butyl-5-(4-(3-chloropropyl)phenyl)-5*H*-dibenzo[*b,d*]thiophen-5-ium trifluoromethanesulfonate, 3,7-di-tert-butyl-5-(2-methoxy-5-(2-oxopyrrolidine-1-carbonyl)phenyl)-5*H*-dibenzo[*b,d*]thiophen-5-ium trifluoromethanesulfonate, 5-(3-chloro-6-methyl-5,5-dioxido-11-oxo-6,11-dihydrodibenzo[*c,f*][*1,2*]thiazepin-8-yl)-5*H*-dibenzo[*b,d*]thiophen-5-ium trifluoromethanesulfonate and 5-(4'-((1*H*-imidazol-1-yl)(phenyl)methyl)-[1,1'-biphenyl]-4-yl)-5*H*-dibenzo[*b,d*]thiophen-5-ium trifluoromethanesulfonate were synthesized as literature reported;[2] 5-(4-((5-methoxy-4,4-dimethyl-5-oxopentyl)oxy)-2,5-dimethylphenyl)-5*H*-dibenzo[*b,d*]thiophen-5-ium trifluoromethanesulfonate, 5-(3,4-dimethoxyphenyl)-5*H*-dibenzo[*b,d*]thiophen-5-ium trifluoromethanesulfonate, 5-(4-cyclohexylphenyl)-5*H*-dibenzo[*b,d*]thiophen-5-ium trifluoromethanesulfonate, 5-mesityl-5*H*-dibenzo[*b,d*]thiophen-5-ium trifluoromethanesulfonate and 5-(3-(ethoxycarbonyl)phenyl)-5*H*-dibenzo[*b,d*]thiophen-5-ium trifluoromethanesulfonate were synthesized as literature reported;[3] 5-(4-bromophenyl)-5*H*-dibenzo[*b,d*]thiophen-5-ium trifluoromethanesulfonate was synthesized as literature reported;[4] rubicene was synthesized as literature reported;[5] Ben-rubicene was synthesized as literature reported;[6] O-rubicene was synthesized as literature reported;[7] S-rubicene and Se-rubicene were synthesized as literature reported.[8]

II.2. Preparation of substrates

II.2.1. The synthesis of aryl sulfonium salts

5-(3-cyano-4-ethoxyphenyl)-5*H*-dibenzo[*b,d*]thiophen-5-ium trifluoromethanesulfonate

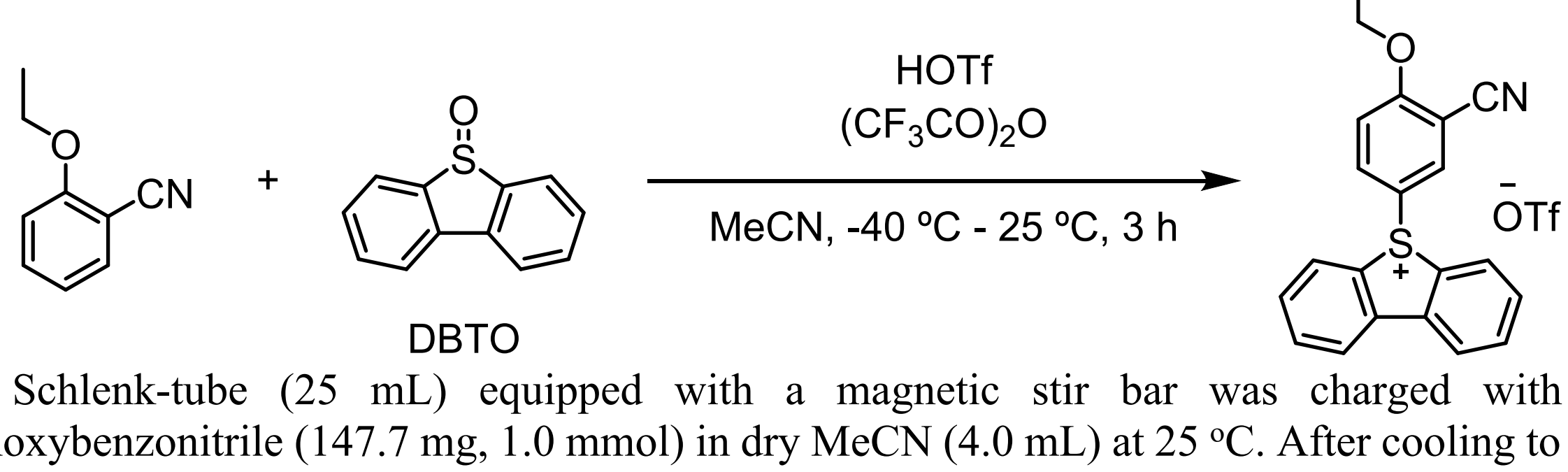

A Schlenk-tube (25 mL) equipped with a magnetic stir bar was charged with 2-ethoxybenzonitrile (147.7 mg, 1.0 mmol) in dry MeCN (4.0 mL) at 25 ºC. After cooling to -40 ºC, trifluoromethanesulfonic acid (464.2 mg, 3.0 mmol) and trifluoroacetic anhydride (645.2 mg, 3.0 mmol) were added to the stirred reaction mixture. After dibenzo[*b,d*]thiophene 5-oxide (DBTO) (300.4 mg, 1.5 mmol) was added to the stirred reaction mixture in small portions over 2 minutes, the reaction mixture was stirred at -40 ºC for 1 h. Afterwards, the Schlenk-tube was taken out from the cold bath and warmed to 25 ºC in air. After stirring at 25 ºC for another 3

h, the reaction mixture was diluted with DCM (30.0 mL) and poured into saturated Na_2CO_3 solution (30.0 mL). Then, the mixture was poured into a separatory funnel, and the layers were separated. After collecting the organic layers, the aqueous layer was further extracted with DCM (3 × *ca.* 20.0 mL). The combined DCM layer was dried over Na_2SO_4, filtered, and the solvent was removed under reduced pressure. Finally, the residue was purified by chromatography on silica gel eluting with DCM:MeOH (100:0 to 96:4, *v/v*) to afford 5-(3-cyano-4-ethoxyphenyl)-5*H*-dibenzo[*b,d*]thiophen-5-ium trifluoromethanesulfonate (460.3 mg, 96%) as a white solid.

^{1}H NMR (500 MHz, CD_3CN): δ 8.34 (d, *J* = 7.8 Hz, 2H), 8.05 (d, *J* = 8.1 Hz, 2H), 7.96 (t, *J* = 7.8 Hz, 2H), 7.89 (d, *J* = 2.6 Hz, 1H), 7.75-7.71 (m, 2H), 7.69 (dd, *J* = 9.4, 2.6 Hz, 1H), 7.23 (d, *J* = 9.2 Hz, 1H), 4.25 (q, *J* = 7.0 Hz, 2H), 1.40 (t, *J* = 7.0 Hz, 3H).

^{13}C NMR (126 MHz, CD_3CN): δ 166.25, 140.40, 138.29, 138.13, 135.62, 132.87, 132.73, 128.95, 125.68, 117.20, 116.58, 114.90, 105.34, 67.58, 14.43.

^{19}F NMR (471 MHz, CD_3CN): δ -79.23.

HRMS (ESI) *m/z* calcd for $C_{21}H_{16}NOS^+$ $[M\text{-}OTf]^+$: 330.0948, found 330.0939.

II.2.2. Substrate scope

Trifluoromethylation of aryl sulfonium salts

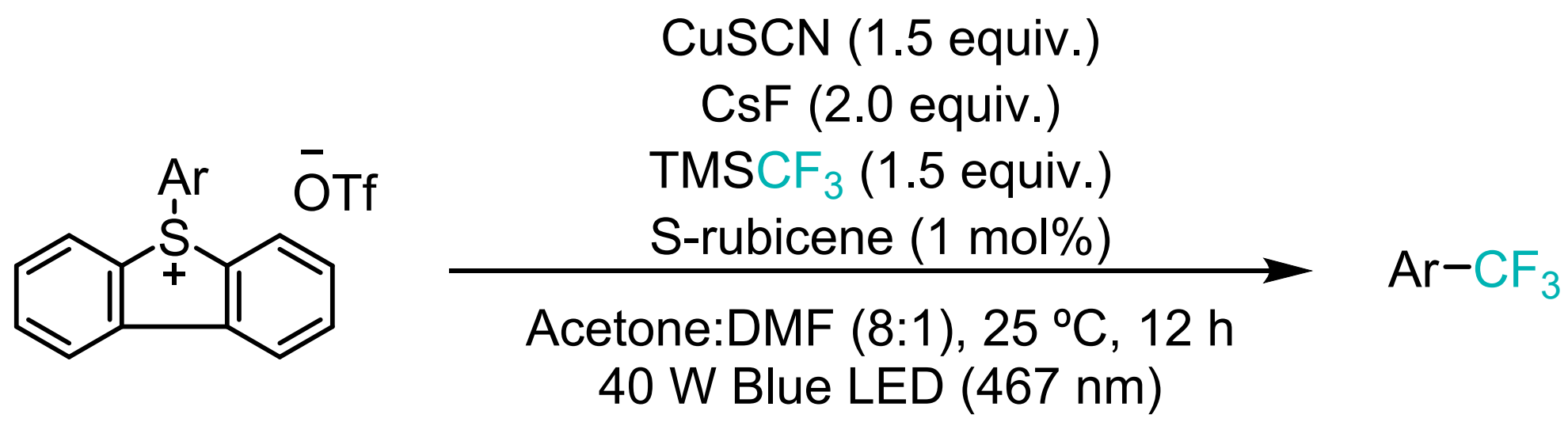

General Procedure:

In a glove-box under nitrogen atmosphere, the $TMSCF_3$ (1.5 equiv.) was added to a solution of the CuSCN (1.5 equiv.) and CsF (2.0 equiv.) in DMF (0.3 M) at 25 °C for 30 minutes. Then this mixture was added to a solution of the aryl sulfonium salts 1 (1.0 equiv.) and S-rubicene (1 mol%) in anhydrous acetone (0.025 M). After the vial was sealed and removed from glove-box, the resulting solution was irradiated with a kessil 40W blue LED (467 nm) lamp at 25 °C for 12 h. Finally, the reaction mixture was concentrated in vacuo and purified by column chromatography (dry-loaded into silica-gel, eluent as specified for individual compounds).

4-(trifluoromethyl)-1,1'-biphenyl (**A1**)

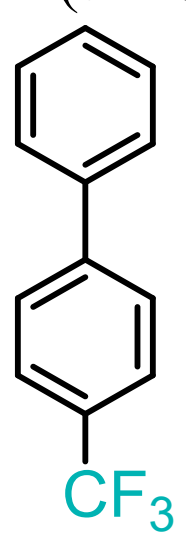

The concentrated mixture was purified by chromatography on silica gel with PE as eluent to afford a colorless solid **A1** (38.7 mg, 87%).

1 g scale reaction:

Prepared as described in General Procedure: $TMSCF_3$ (1306.5 mg, 9.0 mmol), CuSCN (1117.1 mg, 9.0 mmol), CsF (1832.7 mg, 12.0 mmol), 5-([1,1'-biphenyl]-4-yl)-5*H*-dibenzo[*b,d*]thiophen-5-ium trifluoromethanesulfonate (2922.5 mg, 6.0 mmol, 1.0 equiv.) and

S-rubicene (49.3 mg, 0.06 mmol), after purification by column chromatography on silica gel with PE as eluent to afford a colorless solid **A1** (1079.9 mg, 81%).

^{1}H NMR (500 MHz, $CDCl_3$): δ 7.72 (s, 4H), 7.63 (d, *J* = 7.3 Hz, 2H), 7.50 (t, *J* = 7.5 Hz, 2H), 7.44 (t, *J* = 7.3 Hz, 1H).

^{13}C NMR (126 MHz, $CDCl_3$): δ 144.70, 139.73, 129.31 (q, *J* = 32.5 Hz), 128.97, 128.17, 127.39, 127.26, 125.69 (q, *J* = 3.6 Hz), 124.31 (q, *J* = 271.9 Hz).

^{19}F NMR (471 MHz, $CDCl_3$): δ -62.25.

The NMR spectra are matching with the reference.[9]

1-bromo-4-(trifluoromethyl)benzene (**A2**)

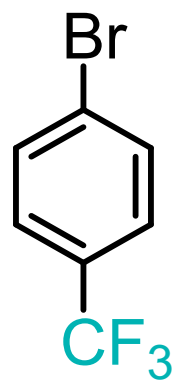

The concentrated mixture was purified by chromatography on silica gel with PE as eluent to afford a colorless oil **A2** (32.4 mg, 72%).

^{1}H NMR (500 MHz, $CDCl_3$): δ 7.61 (d, *J* = 8.2 Hz, 2H), 7.49 (d, *J* = 8.2 Hz, 2H).

^{13}C NMR (126 MHz, $CDCl_3$): δ 132.04, 129.55 (q, *J* = 33.0 Hz), 126.83 (q, *J* = 3.0 Hz), 126.44, 123.89 (q, *J* = 272.0 Hz).

^{19}F NMR (471 MHz, $CDCl_3$): δ -62.98.

The NMR spectra are matching with the reference.[9]

ethyl 3-(trifluoromethyl)benzoate (**A3**)

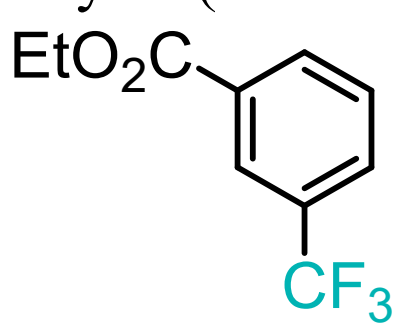

The concentrated mixture was purified by chromatography on silica gel with PE as eluent to afford a colorless liquid **A3** (31.9 mg, 73%).

^{1}H NMR (500 MHz, $CDCl_3$): δ 8.28 (s, 1H), 8.20 (d, *J* = 7.9 Hz, 1H), 7.76 (d, *J* = 7.8 Hz, 1H), 7.53 (t, *J* = 7.8 Hz, 1H), 4.39 (q, *J* = 7.1 Hz, 2H), 1.39 (t, *J* = 7.1 Hz, 3H).

^{13}C NMR (126 MHz, $CDCl_3$): δ 165.14, 132.67, 131.29, 130.89 (q, *J* = 33.1 Hz), 129.19 (q, *J* = 3.2 Hz), 128.90, 126.34 (q, *J* = 3.5 Hz), 123.65 (q, *J* = 272.4 Hz), 61.44, 14.09.

^{19}F NMR (471 MHz, $CDCl_3$): δ -62.79.

The NMR spectra are matching with the reference.[10]

1,2-dichloro-4-(trifluoromethyl)benzene (**A4**)

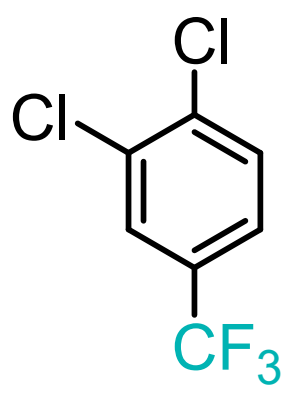

The concentrated mixture was purified by chromatography on silica gel with PE as eluent to afford a colorless liquid **A4** (32.7 mg, 76%).

^{1}H NMR (500 MHz, $CDCl_3$): δ 7.72 (s, 1H), 7.58 (d, *J* = 8.2 Hz, 1H), 7.47 (d, *J* = 8.2 Hz, 1H).

^{13}C NMR (126 MHz, $CDCl_3$): δ 136.60, 133.44, 130.98, 130.34 (q, *J* = 33.7 Hz), 127.57, 124.51, 122.97 (q, *J* = 272.6 Hz).

^{19}F NMR (471 MHz, $CDCl_3$): δ -62.75.

The NMR spectra are matching with the reference.[11]

2-methoxy-5-(trifluoromethyl)benzaldehyde (**A5**)

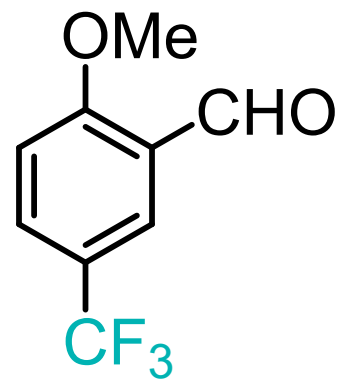

The concentrated mixture was purified by chromatography on silica gel with PE as eluent to afford a colorless oil **A5** (27.8 mg, 68%).

^{1}H NMR (500 MHz, $CDCl_3$): δ 10.46 (s, 1H), 8.09 (d, J = 2.1 Hz, 1H), 7.79 (dd, J = 8.8, 2.3 Hz, 1H), 7.09 (d, J = 8.8 Hz, 1H), 4.00 (s, 3H).

^{13}C NMR (126 MHz, $CDCl_3$): δ 188.45, 163.64, 132.50 (q, J = 3.1 Hz), 125.99 (q, J = 2.5 Hz), 124.52, 123.76 (q, J = 271.4 Hz), 123.23 (q, J = 33.8 Hz), 112.06, 56.08.

^{19}F NMR (471 MHz, $CDCl_3$): δ -61.90.

The NMR spectra are matching with the reference.[9]

2-bromo-1-methoxy-4-(trifluoromethyl)benzene (**A6**)

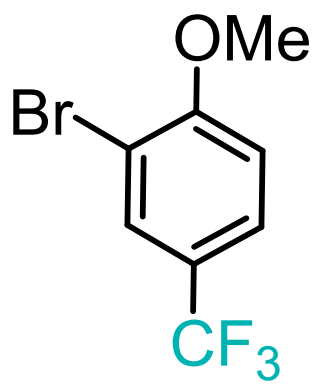

The concentrated mixture was purified by chromatography on silica gel with PE as eluent to afford a colorless liquid **A6** (38.8 mg, 76%).

^{1}H NMR (500 MHz, $CDCl_3$): δ 7.79 (d, J = 1.7 Hz, 1H), 7.53 (dd, J = 8.5, 1.0 Hz, 1H), 6.94 (d, J = 8.6 Hz, 1H), 3.93 (s, 3H).

^{13}C NMR (126 MHz, $CDCl_3$): δ 158.35, 130.47, 125.91 (q, J = 3.6 Hz), 123.88 (q, J = 33.5 Hz), 123.47 (q, J = 271.6 Hz), 111.78, 111.34, 56.36.

^{19}F NMR (471 MHz, $CDCl_3$): δ -61.52.

The NMR spectra are matching with the reference.[12]

4-methoxy-3-(trifluoromethyl)benzonitrile (**A7**)

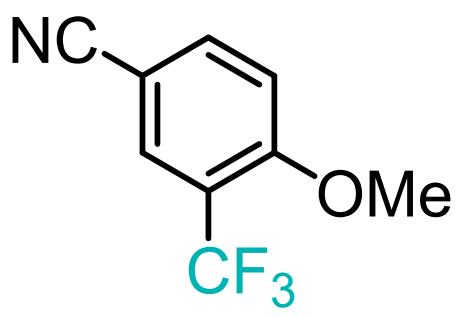

The concentrated mixture was purified by chromatography on silica gel with PE as eluent to afford a oily liquid **A7** (29.4 mg, 73%).

^{1}H NMR (500 MHz, $CDCl_3$): δ 7.86 (s, 1H), 7.81 (dd, J = 8.7, 1.8 Hz, 1H), 7.09 (d, J = 8.7 Hz, 1H), 3.99 (s, 3H).

^{13}C NMR (126 MHz, $CDCl_3$): δ 160.54, 137.58, 131.28 (q, J = 5.5 Hz), 122.29 (q, J = 272.9 Hz), 119.95 (q, J = 32.2 Hz), 117.82, 112.66, 103.79, 56.38.

^{19}F NMR (471 MHz, $CDCl_3$): δ -63.20.

The NMR spectra are matching with the reference.[13]

6-(trifluoromethyl)chroman-4-one (**A8**)

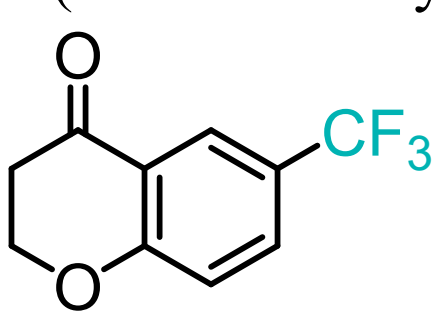

The concentrated mixture was purified by chromatography on silica gel with PE as eluent to afford a white solid **A8** (31.5 mg, 73%).

^{1}H NMR (500 MHz, $CDCl_3$): δ 8.18 (d, J = 1.9 Hz, 1H), 7.69 (dd, J = 8.7, 2.3 Hz, 1H), 7.09 (d, J = 8.7 Hz, 1H), 4.60 (t, J = 6.4 Hz, 2H), 2.86 (t, J = 6.4 Hz, 2H).

^{13}C NMR (126 MHz, $CDCl_3$): δ 190.39, 163.80, 132.22 (q, J = 3.2 Hz), 125.02 (q, J = 3.7 Hz), 123.94 (q, J = 33.5 Hz), 123.73 (q, J = 271.7 Hz), 120.90, 118.88, 67.25, 37.37.

^{19}F NMR (471 MHz, $CDCl_3$): δ -62.08.

HRMS (EI) *m/z* calcd for $C_{10}H_7F_3O_2$ $[M]^+$: 216.0398, found 216.0387.

Arylation of aryl sulfonium salts

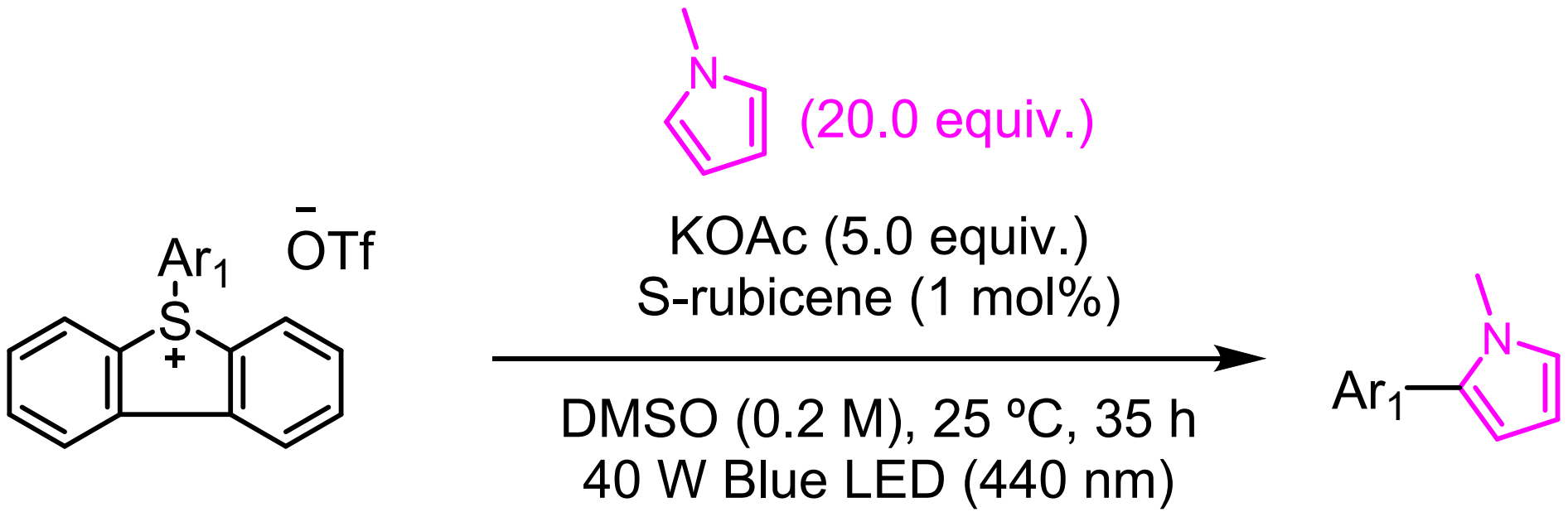

General Procedure:

In a glove-box under nitrogen atmosphere, the aryl (20.0 equiv.) was added to a solution of the aryl sulfonium salts 1 (1.0 equiv.), KOAc (5.0 equiv.) and S-rubicene (1 mol%) in anhydrous DMSO (0.2 M). After the vial was sealed and removed from glove-box, the resulting solution was irradiated with a kessil 40W blue LED (440 nm) lamp at 25 °C for 35 h. Finally, the reaction mixture was concentrated in vacuo and purified by column chromatography (dry-loaded into silica-gel, eluent as specified for individual compounds).

methyl 2-methoxy-5-(1-methyl-1H-pyrrol-2-yl)benzoate (**B1**)

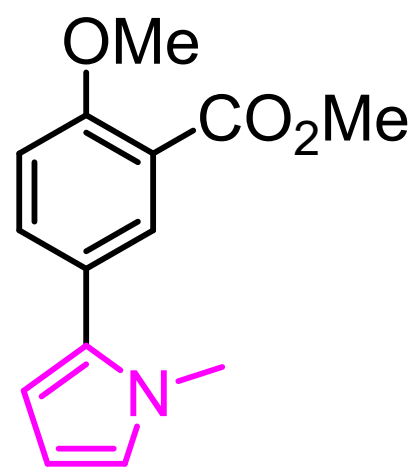

The concentrated mixture was purified by chromatography on silica gel with PE and EA (100:0 to 83:17, *v/v*) as eluent to afford a colorless liquid **B1** (33.8 mg, 69%).

1 g scale reaction:

Prepared as described in General Procedure: 5-(4-methoxy-3-(methoxycarbonyl)phenyl)-5*H*-dibenzo[*b,d*]thiophen-5-ium trifluoromethanesulfonate (3489.8 mg, 7.0 mmol, 1.0 equiv.), KOAc (3470.3 mg, 35.0 mmol), N-Methyl pyrrole (11389.9 mg, 140.0 mmol) and S-rubicene (57.3 mg, 0.07 mmol), after purification by column chromatography on silica gel with PE and EA (100:0 to 87:17, *v/v*) as eluent to afford a colorless liquid **B1** (1253.5 mg, 73%).

^{1}H NMR (500 MHz, $CDCl_3$): δ 7.83 (d, J = 2.4 Hz, 1H), 7.50 (dd, J = 8.6, 2.3 Hz, 1H), 7.02 (d, J = 8.7 Hz, 1H), 6.70 (s, 1H), 6.19 (d, J = 1.9 Hz, 2H), 3.94 (s, 3H), 3.90 (s, 3H), 3.63 (s, 3H).

^{13}C NMR (126 MHz, $CDCl_3$): δ 166.45, 157.95, 133.63, 133.07, 131.83, 125.52, 123.34, 119.80, 112.02, 108.40, 107.64, 56.07, 52.05, 34.84.

The NMR spectra are matching with the reference.[1]

2-(4-cyclohexylphenyl)-1-methyl-1*H*-pyrrole (**B2**)

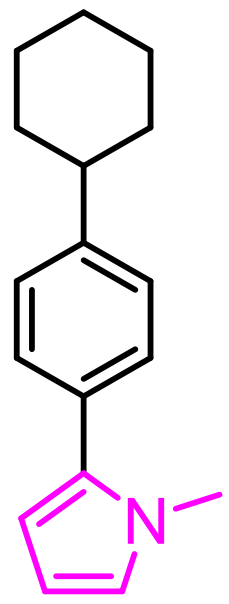

The concentrated mixture was purified by chromatography on silica gel with PE as eluent to afford a red solid **B2** (16.4 mg, 35%).

^{1}H NMR (500 MHz, $CDCl_3$): δ 7.32 (d, J = 8.0 Hz, 2H), 7.23 (d, J = 8.1 Hz, 2H), 6.70 (s, 1H), 6.19 (s, 2H), 3.66 (s, 3H), 2.55-2.50 (m, 1H), 1.92-1.85 (m, 4H), 1.43 (h, J = 12.7 Hz, 4H), 1.31-1.23 (m, 2H).

^{13}C NMR (126 MHz, $CDCl_3$): δ 146.62, 134.70, 130.81, 128.59, 126.75, 123.24, 108.29, 107.60, 44.24, 34.99, 34.42, 26.89, 26.16.

HRMS (EI) *m/z* calcd for $C_{17}H_{21}N$ $[M]^+$: 239.1674, found 239.1667.

2-(4-isopropylphenyl)-1-methyl-1*H*-pyrrole (**B3**)

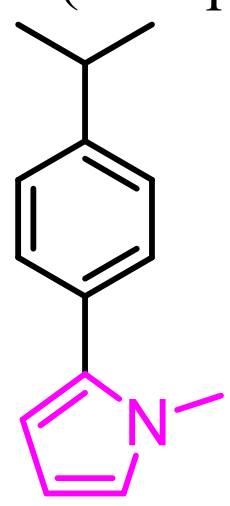

The concentrated mixture was purified by chromatography on silica gel with PE as eluent to afford a yellow liquid **B3** (17.8 mg, 45%).

^{1}H NMR (500 MHz, $CDCl_3$): δ 7.34 (d, J = 8.1 Hz, 2H), 7.27 (d, J = 2.5 Hz, 2H), 6.71 (s, 1H), 6.21 (s, 2H), 3.67 (s, 3H), 2.95 (p, J = 6.9 Hz, 1H), 1.30 (d, J = 6.9 Hz, 6H).

^{13}C NMR (126 MHz, $CDCl_3$): δ 147.37, 134.67, 130.81, 128.63, 126.37, 123.24, 108.30, 107.61, 34.98, 33.80, 23.95.

The NMR spectra are matching with the reference.[1]

2-(4-(3-chloropropyl)phenyl)-1-methyl-1*H*-pyrrole (**B4**)

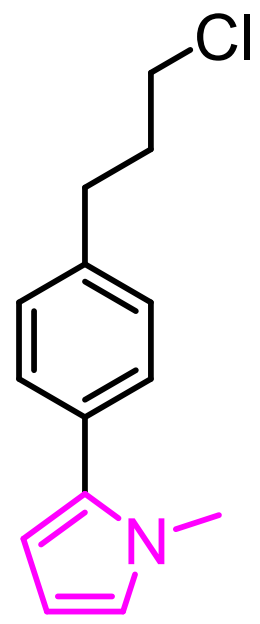

The concentrated mixture was purified by chromatography on silica gel with PE and EA (100:0 to 82:18, *v/v*) as eluent to afford an orange oil **B4** (24.6 mg, 53%).

^{1}H NMR (500 MHz, $CDCl_3$): δ 7.34 (d, J = 8.0 Hz, 2H), 7.23 (d, J = 8.0 Hz, 2H), 6.71 (s, 1H), 6.20 (s, 2H), 3.66 (s, 3H), 3.57 (t, J = 6.5 Hz, 2H), 2.81 (t, J = 7.4 Hz, 2H), 2.12 (p, J = 6.7 Hz, 2H).

^{13}C NMR (126 MHz, $CDCl_3$): δ 139.20, 134.41, 131.24, 128.75, 128.52, 123.47, 108.44, 107.69, 44.24, 35.02, 33.92, 32.43.

HRMS (EI) *m/z* calcd for $C_{14}H_{16}ClN$ $[M]^+$: 233.0971, found 233.0962.

2-(4-iodophenyl)-1-methyl-1*H*-pyrrole (**B5**)

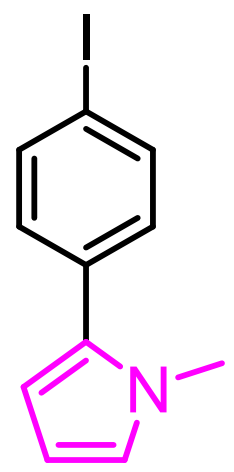

The concentrated mixture was purified by chromatography on silica gel with PE and EA (100:0 to 90:10, *v/v*) as eluent to afford a white solid **B5** (29.3 mg, 52%).

^{1}H NMR (500 MHz, $CDCl_3$): δ 7.72 (d, *J* = 8.2 Hz, 2H), 7.14 (d, *J* = 8.2 Hz, 2H), 6.73 (s, 1H), 6.23 (d, *J* = 3.3 Hz, 1H), 6.21-6.20 (m, 1H), 3.66 (s, 3H).

^{13}C NMR (126 MHz, $CDCl_3$): δ 137.45, 133.39, 132.77, 130.24, 124.21, 109.02, 107.99, 92.11, 35.07.

The NMR spectra are matching with the reference.[14]

2-(3,4-dimethoxyphenyl)-1-methyl-1*H*-pyrrole (**B6**)

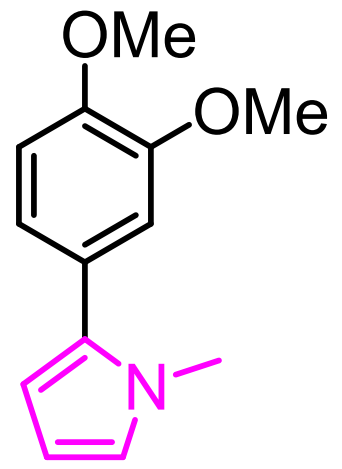

The concentrated mixture was purified by chromatography on silica gel with PE and EA (100:0 to 82:18, *v/v*) as eluent to afford a brown liquid **B6** (23.4 mg, 54%).

^{1}H NMR (500 MHz, $CDCl_3$): δ 6.95-6.90 (m, 3H), 6.70 (s, 1H), 6.20-6.17 (m, 2H), 3.92 (s, 3H), 3.90 (s, 3H), 3.64 (s, 3H).

^{13}C NMR (126 MHz, $CDCl_3$): δ 148.66, 148.17, 134.47, 126.20, 123.02, 121.21, 112.46, 111.00, 108.04, 107.57, 55.92, 55.89, 34.88.

The NMR spectra are matching with the reference.[15]

2-(3,4-dichlorophenyl)-1-methyl-1*H*-pyrrole (**B7**)

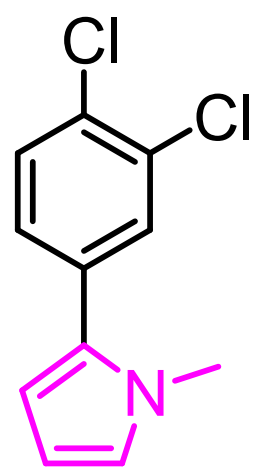

The concentrated mixture was purified by chromatography on silica gel with PE as eluent to afford a colorless liquid **B7** (28.4 mg, 63%).

^{1}H NMR (500 MHz, $CDCl_3$): δ 7.49 (d, *J* = 1.8 Hz, 1H), 7.46 (d, *J* = 8.3 Hz, 1H), 7.23 (dd, *J* = 8.3, 1.8 Hz, 1H), 6.73 (s, 1H), 6.25 (dd, *J* = 3.3, 1.7 Hz, 1H), 6.20 (t, *J* = 3.0 Hz, 1H), 3.67 (s, 3H).

^{13}C NMR (126 MHz, $CDCl_3$): δ 133.30, 132.40, 132.01, 130.64, 130.27, 129.99, 127.56, 124.63, 109.64, 108.14, 35.10.

The NMR spectra are matching with the reference.[16]

4-methoxy-3-(1-methyl-1*H*-pyrrol-2-yl)benzonitrile (**B8**)

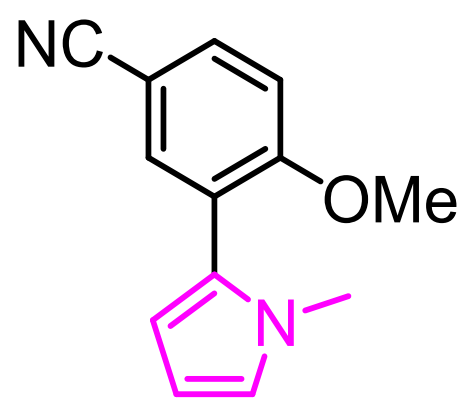

The concentrated mixture was purified by chromatography on silica gel with PE and EA (100:0 to 80:20, *v/v*) as eluent to afford a purple solid **B8** (41.0 mg, 97%).

^{1}H NMR (500 MHz, $CDCl_3$): δ 7.64 (dd, *J* = 8.6, 1.7 Hz, 1H), 7.56 (d, *J* = 1.7 Hz, 1H), 7.00 (d, *J* = 8.7 Hz, 1H), 6.75 (s, 1H), 6.22 (t, *J* = 2.8 Hz, 1H), 6.16 (s, 1H), 3.88 (s, 3H), 3.48 (s, 3H).

^{13}C NMR (126 MHz, $CDCl_3$): δ 160.58, 135.71, 133.56, 128.51, 123.97, 123.46, 118.94, 111.26, 110.08, 107.91, 104.10, 55.82, 34.61.

The NMR spectra are matching with the reference.[1]

2-ethoxy-5-(1-methyl-1*H*-pyrrol-2-yl)benzonitrile (**B9**)

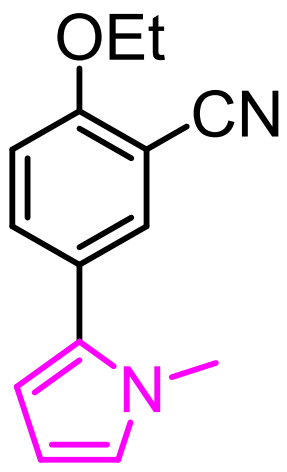

The concentrated mixture was purified by chromatography on silica gel with PE and EA (98:2 to 84:16, *v/v*) as eluent to afford a white solid **B9** (36.6 mg, 81%).

^{1}H NMR (500 MHz, $CDCl_3$): δ 7.56 (d, *J* = 1.5 Hz, 1H), 7.53 (dd, *J* = 8.5, 1.8 Hz, 1H), 6.98 (d, *J* = 8.7 Hz, 1H), 6.71 (s, 1H), 6.18 (s, 2H), 4.19 (q, *J* = 7.0 Hz, 2H), 3.62 (s, 3H), 1.51 (t, *J* = 7.0 Hz, 3H).

^{13}C NMR (126 MHz, $CDCl_3$): δ 159.43, 134.51, 133.46, 131.99, 126.26, 123.93, 116.38, 112.27, 108.94, 107.93, 102.08, 64.84, 34.91, 14.53.

HRMS (EI) *m/z* calcd for $C_{14}H_{14}N_2O$ $[M]^+$: 226.1106, found 226.1096.

2-methoxy-5-(1-methyl-1*H*-pyrrol-2-yl)benzaldehyde (**B10**)

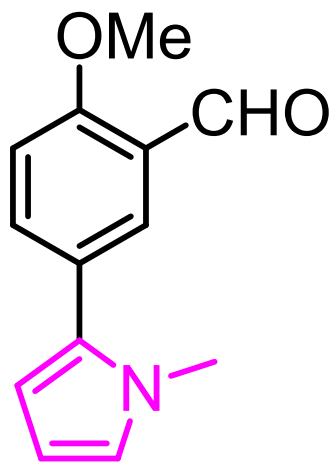

The concentrated mixture was purified by chromatography on silica gel with PE and EA (100:0 to 82:18, *v/v*) as eluent to afford a green solid **B10** (16.7 mg, 39%).

^{1}H NMR (500 MHz, CD_3CN): δ 10.44 (s, 1H), 7.74 (d, *J* = 2.3 Hz, 1H), 7.66 (dd, *J* = 8.7, 2.4 Hz, 1H), 7.19 (d, *J* = 8.6 Hz, 1H), 6.73 (t, *J* = 2.5 Hz, 1H), 6.15 (dd, *J* = 3.4, 1.9 Hz, 1H), 6.09 (t, *J* = 3.0 Hz, 1H), 3.96 (s, 3H), 3.61 (s, 3H).

^{13}C NMR (126 MHz, CD_3CN): δ 190.15, 161.75, 136.88, 133.60, 128.25, 127.13, 125.57, 124.90, 113.66, 109.31, 108.48, 56.83, 35.41.

HRMS (EI) *m/z* calcd for $C_{13}H_{13}NO_2$ $[M]^+$: 215.0946, found 215.0937.

1-(4-(1-methyl-1H-pyrrol-2-yl)phenyl)pyrrolidin-2-one (**B11**)

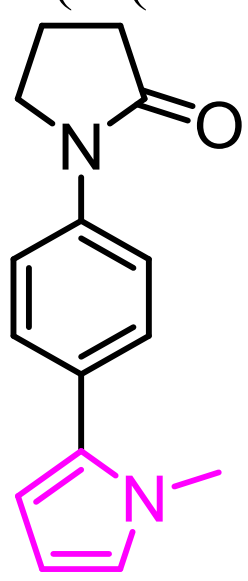

The concentrated mixture was purified by chromatography on silica gel with PE and EA (92:8 to 34:66, *v/v*) as eluent to afford a white solid **B11** (29.8 mg, 62%).

^{1}H NMR (500 MHz, $CDCl_3$): δ 7.65 (d, *J* = 8.7 Hz, 2H), 7.40 (d, *J* = 8.5 Hz, 2H), 6.71 (s, 1H), 6.20 (t, *J* = 3.9 Hz, 2H), 3.90 (t, *J* = 7.0 Hz, 2H), 3.66 (s, 3H), 2.64 (t, *J* = 8.1 Hz, 2H), 2.19 (p, *J* = 7.6 Hz, 2H).

^{13}C NMR (126 MHz, $CDCl_3$): δ 174.24, 138.03, 134.03, 129.53, 128.98, 123.61, 119.79, 108.53, 107.74, 48.75, 35.05, 32.73, 18.03.

The NMR spectra are matching with the reference.[1]

1-(4-methoxy-3-(1-methyl-1*H*-pyrrol-2-yl)benzoyl)pyrrolidin-2-one (**B12**)

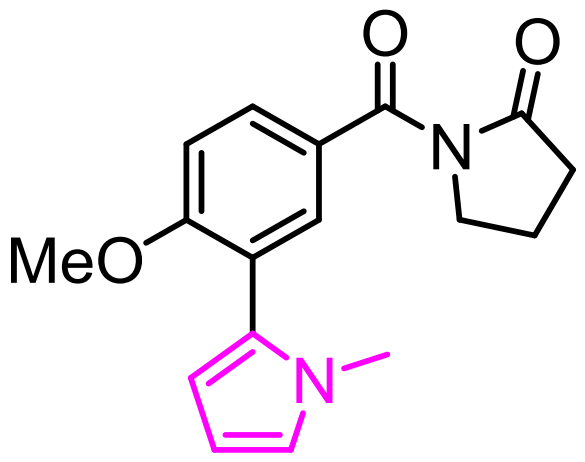

The concentrated mixture was purified by chromatography on silica gel with PE and EA (92:8 to 47:53, *v/v*) as eluent to afford a brown solid **B12** (39.7 mg, 67%).

^{1}H NMR (500 MHz, $CDCl_3$): δ 7.71 (dd, *J* = 8.6, 2.3 Hz, 1H), 7.60 (d, *J* = 2.4 Hz, 1H), 6.96 (d, *J* = 8.7 Hz, 1H), 6.72 (s, 1H), 6.21 (t, *J* = 3.0 Hz, 1H), 6.16 (d, *J* = 3.3 Hz, 1H), 3.94 (t, *J* = 7.1 Hz, 2H), 3.87 (s, 3H), 3.49 (s, 3H), 2.60 (t, *J* = 7.9 Hz, 2H), 2.13 (q, *J* = 7.4 Hz, 2H).

^{13}C NMR (126 MHz, $CDCl_3$): δ 174.60, 169.90, 160.69, 134.25, 131.31, 129.92, 126.01, 122.69, 121.77, 109.76, 109.44, 107.53, 55.66, 46.81, 34.58, 33.37, 17.75.

HRMS (EI) *m/z* calcd for $C_{17}H_{18}N_2O_3$ $[M]^+$: 298.1317, found 298.1307.

isopropyl 2-(4-(4-chlorobenzoyl)-2-(1-methyl-1H-pyrrol-2-yl)phenoxy)-2-methylpropanoate (**B13**)

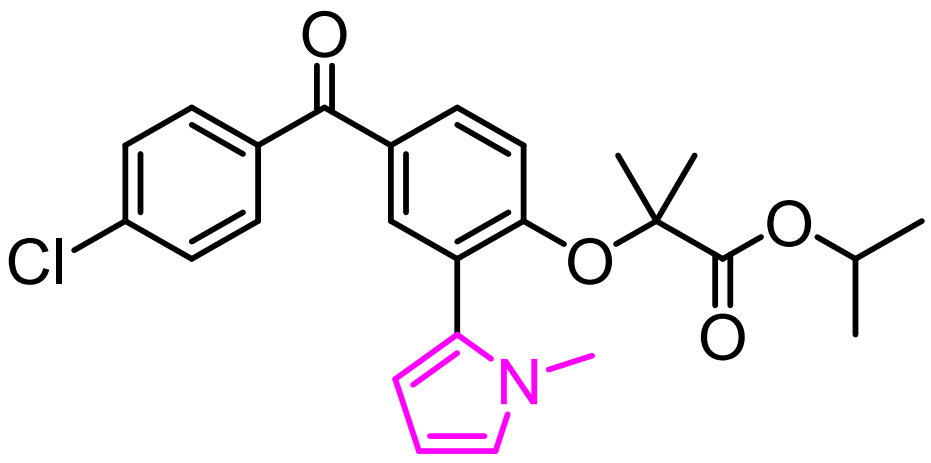

The concentrated mixture was purified by chromatography on silica gel with PE and EA (100:0 to 91:9, *v/v*) as eluent to afford a yellow solid **B13** (18.9 mg, 22%).

^{1}H NMR (500 MHz, $CDCl_3$): δ 7.76 (d, J = 2.1 Hz, 1H), 7.73 (d, J = 8.4 Hz, 3H), 7.45 (d, J = 8.4 Hz, 2H), 6.83 (d, J = 8.5 Hz, 1H), 6.73 (s, 1H), 6.20-6.19 (m, 1H), 6.13 (d, J = 2.8 Hz, 1H), 5.08 (p, J = 6.2 Hz, 1H), 3.58 (s, 3H), 1.55 (s, 6H), 1.21 (d, J = 6.3 Hz, 6H).

^{13}C NMR (126 MHz, $CDCl_3$): δ 194.15, 173.06, 157.52, 138.41, 136.25, 134.91, 131.12, 130.78, 130.36, 130.23, 128.56, 124.93, 122.83, 115.52, 109.51, 107.44, 80.02, 69.34, 34.81, 25.18, 21.55.

The NMR spectra are matching with the reference.[1]

(*2R,4R,5R,6S*)-2-(acetoxymethyl)-6-(2-(acetoxymethyl)-4-(1-methyl-1*H*-pyrrol-2-yl)phenoxy)tetrahydro-2*H*-pyran-3,4,5-triyl triacetate (**B14**)

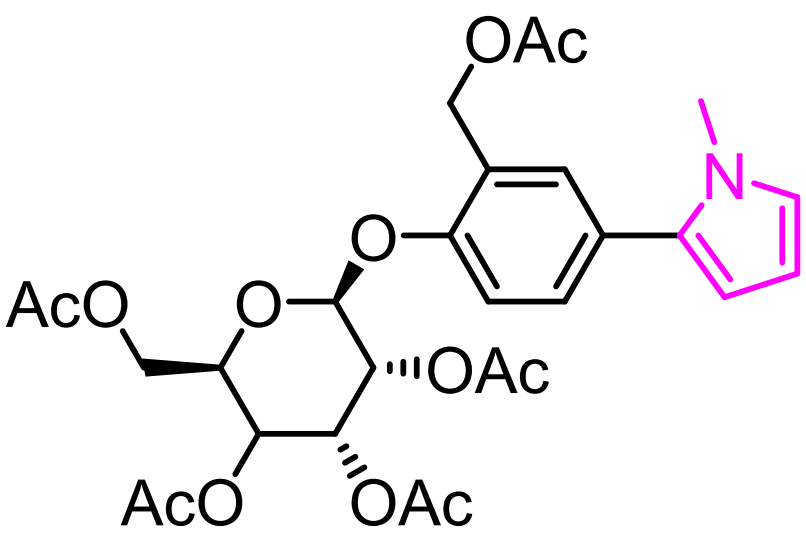

The concentrated mixture was purified by chromatography on silica gel with PE and EA (100:0 to 50:50, *v/v*) as eluent to afford a yellow liquid **B14** (105.9 mg, 92%).

^{1}H NMR (500 MHz, $CDCl_3$): δ 7.37 (s, 1H), 7.29 (d, *J* = 8.4 Hz, 1H), 7.10 (d, *J* = 8.3 Hz, 1H), 6.70 (s, 1H), 6.17 (s, 2H), 5.32 (d, *J* = 6.3 Hz, 2H), 5.21-5.17 (m, 2H), 5.11-5.08 (m, 2H), 4.29 (dd, *J* = 11.9, 4.8 Hz, 1H), 4.20 (d, *J* = 11.5 Hz, 1H), 3.88 (s, 1H), 3.62 (s, 3H), 2.10 (d, *J* = 5.4 Hz, 6H), 2.08 (s, 3H), 2.05 (s, 6H).

^{13}C NMR (126 MHz, $CDCl_3$): δ 170.64, 170.50, 170.19, 169.34, 169.26, 153.32, 133.50, 129.72, 129.54, 128.78, 126.12, 123.55, 115.65, 108.57, 107.73, 99.25, 72.57, 72.01, 70.94, 68.27, 61.86, 60.92, 34.89, 20.92, 20.63, 20.57.

The NMR spectra are matching with the reference.[1]

Borylation of aryl sulfonium salts

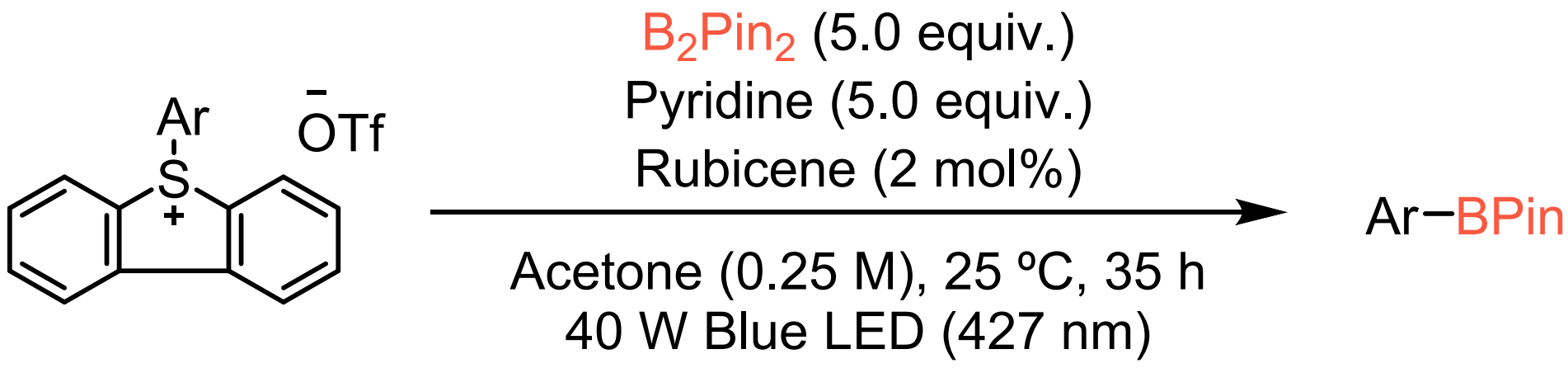

General Procedure:

In a glove-box under nitrogen atmosphere, the pyridine (5.0 equiv.) was added to a solution of the aryl sulfonium salts 1 (1.0 equiv.), bis(pinacolato)diboron (B_2Pin_2) (5.0 equiv.) and rubicene (2 mol%) in anhydrous acetone (0.25 M). After the vial was sealed and removed from glove-box, the resulting solution was irradiated with a kessil 40W blue LED (427 nm) lamp at 25 °C for 35 h. Finally, the reaction mixture was concentrated in vacuo and purified by column chromatography (dry-loaded into silica-gel, eluent as specified for individual compounds).

2-mesityl-4,4,5,5-tetramethyl-1,3,2-dioxaborolane (**C1**)

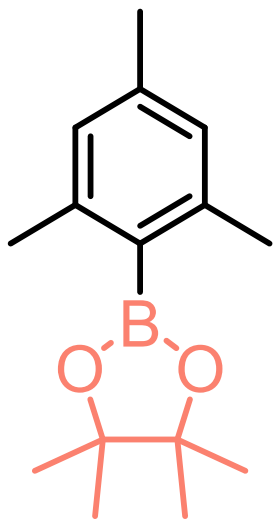

The concentrated mixture was purified by chromatography on silica gel with PE and EA (100:0 to 93:7, *v/v*) as eluent to afford a yellow oil **C1** (33.7 mg, 69%).

^{1}H NMR (500 MHz, $CDCl_3$): δ 6.77 (s, 2H), 2.37 (s, 6H), 2.24 (s, 3H), 1.37 (s, 12H).

^{13}C NMR (126 MHz, $CDCl_3$): δ 142.08, 138.87, 127.40, 83.40, 24.91, 22.16, 21.20.

The NMR spectra are matching with the reference.[17]

2-(4-isopropylphenyl)-4,4,5,5-tetramethyl-1,3,2-dioxaborolane (**C2**)

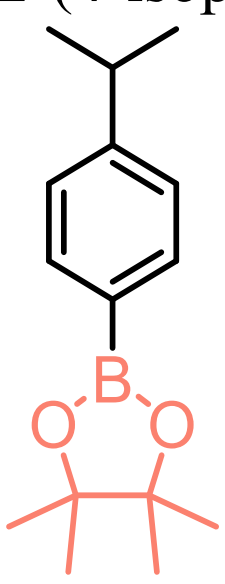

The concentrated mixture was purified by chromatography on silica gel with PE and EA (100:0 to 91:9, *v/v*) as eluent to afford a yellow solid **C2** (34.5 mg, 70%).

1 g scale reaction:

Prepared as described in General Procedure: 5-(4-isopropylphenyl)-5*H*-dibenzo[*b,d*]thiophen-5-ium trifluoromethanesulfonate (3394.2 mg, 7.5 mmol, 1.0 equiv.), pyridine (2981.6 mg, 37.5

mmol), bis(pinacolato)diboron (B_2Pin_2) (9667.3 mg, 37.3 mmol) and rubicene (52.4 mg, 0.15 mmol), after purification by column chromatography on silica gel with PE and EA (100:0 to 91:9, *v/v*) as eluent to afford a yellow solid **C2** (1329.5 mg, 72%).

^{1}H NMR (500 MHz, $CDCl_3$): δ 7.75 (d, *J* = 7.7 Hz, 2H), 7.24 (d, *J* = 7.8 Hz, 2H), 2.91 (p, *J* = 6.9 Hz, 1H), 1.33 (s, 12H), 1.25 (d, *J* = 6.9 Hz, 6H).

^{13}C NMR (126 MHz, $CDCl_3$): δ 152.30, 134.91, 125.89, 83.59, 34.33, 24.83, 23.82.

The NMR spectra are matching with the reference.[18]

2-(4-cyclohexylphenyl)-4,4,5,5-tetramethyl-1,3,2-dioxaborolane (**C3**)

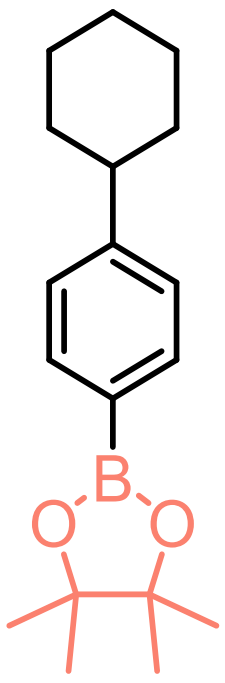

The concentrated mixture was purified by chromatography on silica gel with PE and EA (100:0 to 91:9, *v/v*) as eluent to afford an orange solid **C3** (46.9 mg, 82%).

^{1}H NMR (500 MHz, $CDCl_3$): δ 7.74 (d, *J* = 7.9 Hz, 2H), 7.22 (d, *J* = 7.9 Hz, 2H), 2.53-2.48 (m, 1H), 1.85 (t, *J* = 12.9 Hz, 4H), 1.44-1.37 (m, 4H), 1.33 (s, 12H), 1.29-1.24 (m, 2H).

^{13}C NMR (126 MHz, $CDCl_3$): δ 151.47, 134.87, 126.32, 83.57, 44.84, 34.23, 26.84, 26.13, 24.82.

The NMR spectra are matching with the reference.[19]

2-([1,1'-biphenyl]-4-yl)-4,4,5,5-tetramethyl-1,3,2-dioxaborolane (**C4**)

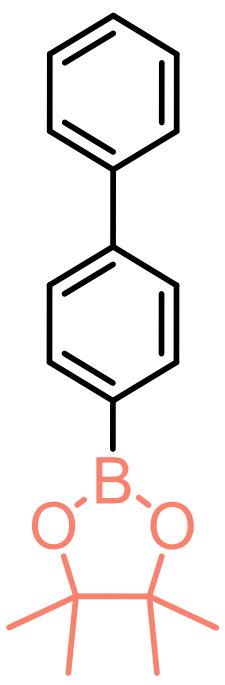

The concentrated mixture was purified by chromatography on silica gel with PE and EA (100:0 to 95:5, *v/v*) as eluent to afford a white solid **C4** (42.0 mg, 75%).

^{1}H NMR (500 MHz, $CDCl_3$): δ 7.89 (d, *J* = 7.9 Hz, 2H), 7.63 (d, *J* = 3.6 Hz, 2H), 7.61 (d, *J* = 4.3 Hz, 2H), 7.45 (t, *J* = 7.6 Hz, 2H), 7.36 (t, *J* = 7.3 Hz, 1H), 1.37 (s, 12H).

^{13}C NMR (126 MHz, $CDCl_3$): δ 143.85, 140.97, 135.23, 128.73, 127.52, 127.19, 126.43, 83.79, 24.85.

The NMR spectra are matching with the reference.[19]

2-(4-iodophenyl)-4,4,5,5-tetramethyl-1,3,2-dioxaborolane (**C5**)

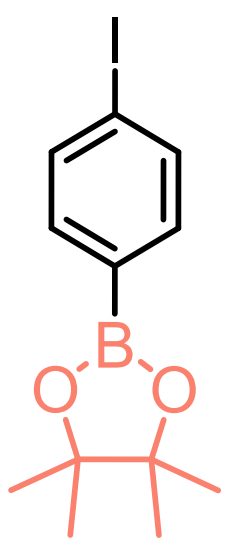

The concentrated mixture was purified by chromatography on silica gel with PE and EA (100:0 to 91:9, *v/v*) as eluent to afford an orange oil **C5** (38.3 mg, 58%).

^{1}H NMR (500 MHz, $CDCl_3$): δ 7.72 (d, J = 8.0 Hz, 2H), 7.51 (d, J = 8.1 Hz, 2H), 1.33 (s, 12H).

^{13}C NMR (126 MHz, $CDCl_3$): δ 136.91, 136.26, 98.81, 84.03, 24.84.

The NMR spectra are matching with the reference.[17]

2-(3,4-dichlorophenyl)-4,4,5,5-tetramethyl-1,3,2-dioxaborolane (**C6**)

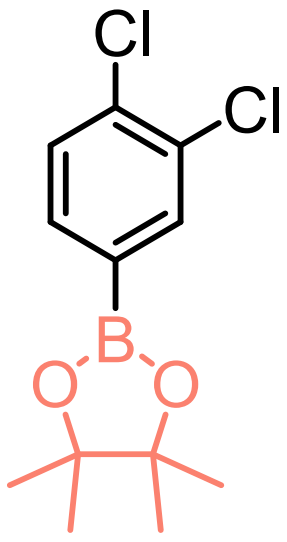

The concentrated mixture was purified by chromatography on silica gel with PE and EA (100:0 to 91:9, *v/v*) as eluent to afford an orange liquid **C6** (38.2 mg, 70%).

^{1}H NMR (500 MHz, $CDCl_3$): δ 7.86 (s, 1H), 7.59 (d, J = 7.9 Hz, 1H), 7.44 (d, J = 8.2 Hz, 1H), 1.34 (s, 12H).

^{13}C NMR (126 MHz, $CDCl_3$): δ 136.55, 135.49, 133.74, 132.25, 130.00, 84.33, 24.83.

The NMR spectra are matching with the reference.[20]

2-methoxy-5-(4,4,5,5-tetramethyl-1,3,2-dioxaborolan-2-yl)phenyl trifluoromethanesulfonate (**C7**)

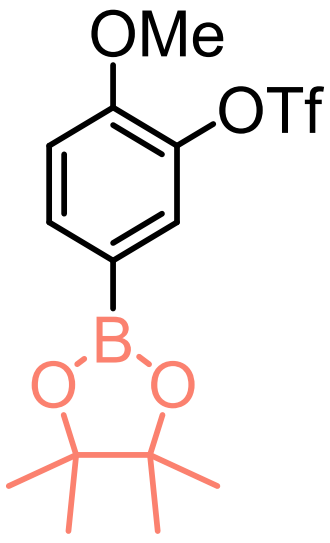

The concentrated mixture was purified by chromatography on silica gel with PE and EA (100:0 to 91:9, *v/v*) as eluent to afford a yellow solid **C7** (49.7 mg, 65%).

^{1}H NMR (500 MHz, $CDCl_3$): δ 7.76 (d, J = 8.1 Hz, 1H), 7.62 (s, 1H), 7.01 (d, J = 8.2 Hz, 1H), 3.93 (s, 3H), 1.33 (s, 12H).

^{13}C NMR (126 MHz, $CDCl_3$): δ 153.66, 138.52, 136.14, 128.31, 119.98, 117.44, 112.39, 84.12, 56.13, 24.82.

^{19}F NMR (471 MHz, $CDCl_3$): δ -73.80.

HRMS (EI) m/z calcd for $C_{14}H_{18}BF_3O_6S$ $[M]^+$: 382.0869, found 382.0858.

methyl 2-methoxy-5-(4,4,5,5-tetramethyl-1,3,2-dioxaborolan-2-yl)benzoate (**C8**)

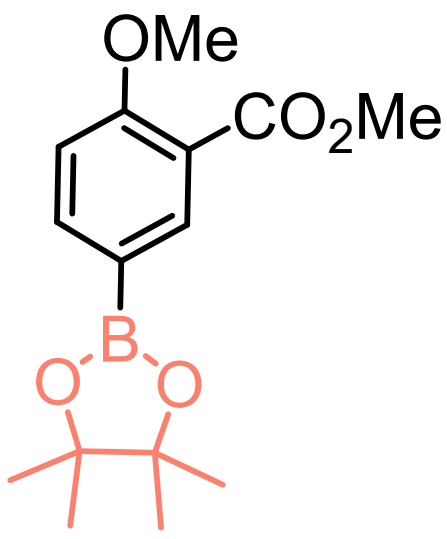

The concentrated mixture was purified by chromatography on silica gel with PE and EA (100:0 to 82:18, *v/v*) as eluent to afford a white solid **C8** (37.4 mg, 64%).

^{1}H NMR (500 MHz, $CDCl_3$): δ 8.22 (d, *J* = 1.5 Hz, 1H), 7.90 (dd, *J* = 8.4, 1.5 Hz, 1H), 6.96 (d, *J* = 8.4 Hz, 1H), 3.92 (s, 3H), 3.88 (s, 3H), 1.33 (s, 12H).

^{13}C NMR (126 MHz, $CDCl_3$): δ 166.54, 161.39, 140.20, 138.32, 119.54, 111.13, 83.84, 55.90, 51.91, 24.83.

The NMR spectra are matching with the reference.[21]

4,4,5,5-tetramethyl-2-(thiophen-2-yl)-1,3,2-dioxaborolane (**C9**)

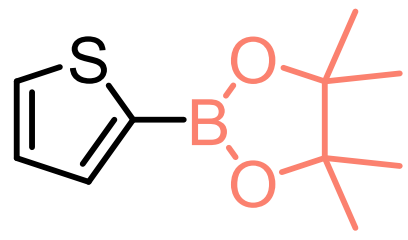

The concentrated mixture was purified by chromatography on silica gel with PE and EA (100:0 to 93:7, *v/v*) as eluent to afford an orange liquid **C9** (17.6 mg, 42%).

^{1}H NMR (500 MHz, $CDCl_3$): δ 7.66 (d, *J* = 2.4 Hz, 1H), 7.64 (d, *J* = 4.6 Hz, 1H), 7.19 (t, *J* = 3.6 Hz, 1H), 1.35 (s, 12H).

^{13}C NMR (126 MHz, $CDCl_3$): δ 137.14, 132.35, 128.20, 84.07, 24.76.

The NMR spectra are matching with the reference.[22]

1-(phenyl(4'-(4,4,5,5-tetramethyl-1,3,2-dioxaborolan-2-yl)-[1,1'-biphenyl]-4-yl)methyl)-1*H*-imidazole (**C10**)

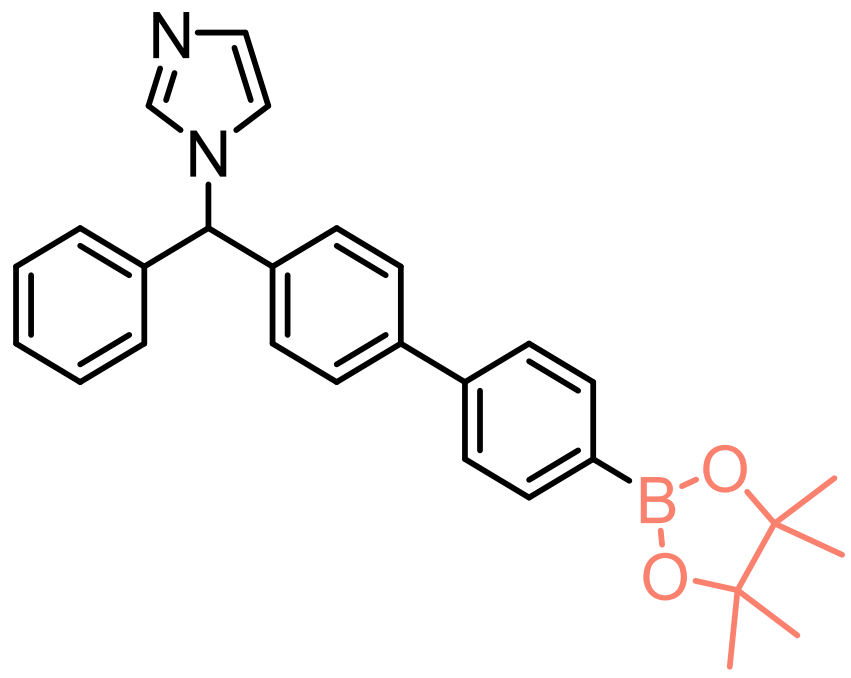

The concentrated mixture was purified by chromatography on silica gel with PE and EA (100:0 to 91:9, *v/v*) as eluent to afford a yellow solid **C10** (53.2 mg, 61%).

^{1}H NMR (500 MHz, $CDCl_3$): δ 7.89 (d, *J* = 8.0 Hz, 2H), 7.60 (t, *J* = 7.8 Hz, 4H), 7.4-7.34 (m, 4H), 7.18-7.14 (m, 6H), 6.56 (s, 1H), 1.36 (s, 12H).

^{13}C NMR (126 MHz, $CDCl_3$): δ 142.79, 141.09, 138.94, 138.32, 135.31, 128.91, 128.46, 128.05, 127.62, 126.35, 83.85, 64.88, 24.85.

HRMS (ESI) *m/z* calcd for $C_{28}H_{30}BN_2O_2^+$ $[M+H]^+$: 437.2395, found 437.2386.

Thioetheration of aryl sulfonium salts

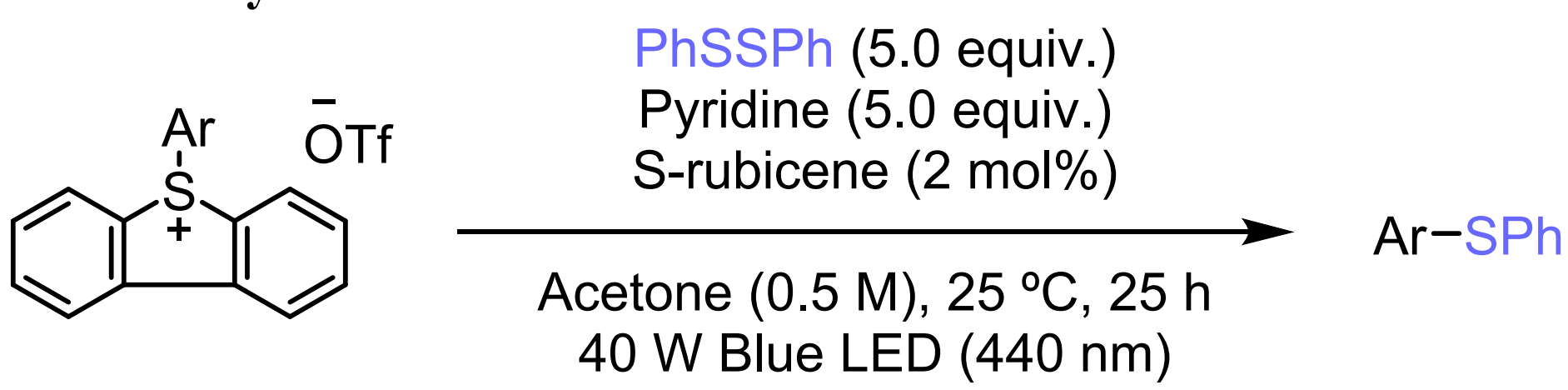

General Procedure:

In a glove-box under nitrogen atmosphere, the pyridine (5.0 equiv.) was added to a solution of the aryl sulfonium salts 1 (1.0 equiv.), diphenyl disulfide (PhSSPh) (5.0 equiv.) and S-rubicene (2 mol%) in anhydrous acetone (0.5 M). After the vial was sealed and removed from glove-box, the resulting solution was irradiated with a kessil 40W blue LED (440 nm) lamp at 25 °C for 25 h. Finally, the reaction mixture was concentrated in vacuo and purified by column chromatography (dry-loaded into silica-gel, eluent as specified for individual compounds).

methyl 2-methoxy-5-(phenylthio)benzoate (**D1**)

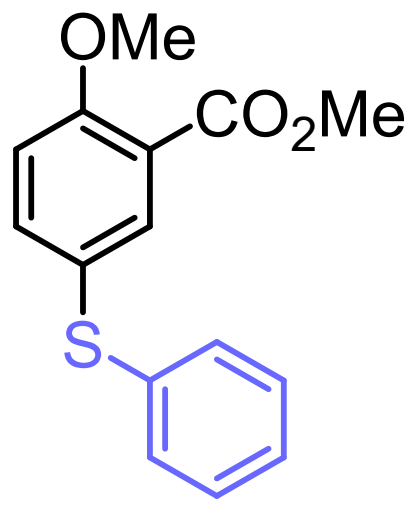

The concentrated mixture was purified by chromatography on silica gel with petroleum ether (PE) and ethyl acetate (EA) (100:0 to 91:9, *v/v*) as eluent to afford a yellow liquid **D1** (50.2 mg, 92%).

^{1}H NMR (500 MHz, CD_3CN): δ 7.75 (d, *J* = 2.6 Hz, 1H), 7.58 (dd, *J* = 8.7, 2.3 Hz, 1H), 7.31-7.28 (m, 2H), 7.23-7.20 (m, 3H), 7.10 (d, *J* = 8.8 Hz, 1H), 3.85 (s, 3H), 3.79 (s, 3H).

^{13}C NMR (126 MHz, CD_3CN): δ 166.64, 159.73, 139.23, 138.23, 136.69, 130.27, 129.96, 127.58, 125.47, 122.68, 114.68, 56.83, 52.67.

The NMR spectra are matching with the reference.[23]

2-ethoxy-5-(phenylthio)benzonitrile (**D2**)

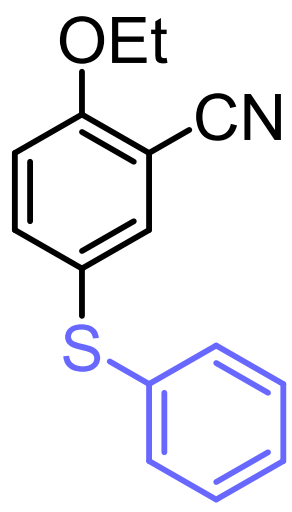

The concentrated mixture was purified by chromatography on silica gel with PE and EA (100:0 to 95:5, *v/v*) as eluent to afford a white solid **D2** (41.4 mg, 81%).

^{1}H NMR (500 MHz, CD_3CN): δ 7.68 (s, 1H), 7.62 (d, *J* = 8.9 Hz, 1H), 7.34-7.31 (m, 2H), 7.27 (d, *J* = 6.8 Hz, 3H), 7.11 (d, *J* = 8.9 Hz, 1H), 4.19 (q, *J* = 6.9 Hz, 2H), 1.42 (t, *J* = 6.9 Hz, 3H).

^{13}C NMR (126 MHz, $CDCl_3$): δ 160.18, 138.38, 137.33, 135.87, 130.06, 129.32, 127.13, 126.76, 115.71, 113.04, 103.16, 65.01, 14.45.

HRMS (EI) *m/z* calcd for $C_{15}H_{13}NOS$ $[M]^+$: 255.0718, found 255.0707.

2-methoxy-5-(phenylthio)phenyl trifluoromethanesulfonate (**D3**)

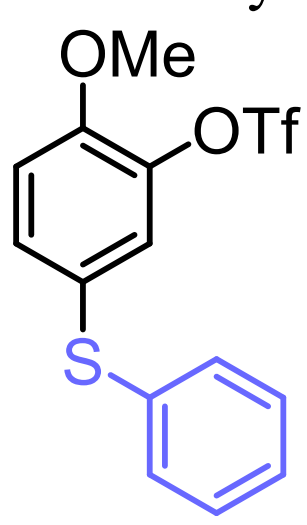

The concentrated mixture was purified by chromatography on silica gel with PE and EA (100:0 to 95:5, *v/v*) as eluent to afford a yellow oil **D3** (45.9 mg, 63%).

^{1}H NMR (500 MHz, CD_3CN): δ 7.46 (d, *J* = 8.6 Hz, 1H), 7.36-7.27 (m, 6H), 7.20 (d, *J* = 8.7 Hz, 1H), 3.91 (s, 3H).

^{13}C NMR (126 MHz, CD_3CN): δ 152.19, 139.63, 136.81, 134.64, 131.09, 130.47, 128.33, 127.70, 127.00, 120.92, 115.60, 57.39.

^{19}F NMR (471 MHz, CD_3CN): δ -74.81.

HRMS (EI) *m/z* calcd for $C_{14}H_{11}F_3O_4S_2$ $[M]^+$: 364.0051, found 364.0042.

2-methoxy-5-(phenylthio)benzaldehyde (**D4**)

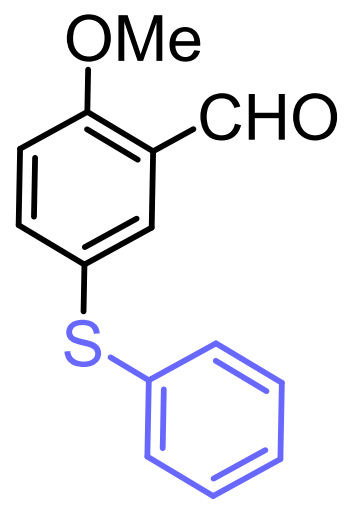

The concentrated mixture was purified by chromatography on silica gel with PE and EA (100:0 to 91:9, *v/v*) as eluent to afford a brown solid **D4** (15.2 mg, 31%).

^{1}H NMR (500 MHz, CD_3CN): δ 10.36 (s, 1H), 7.73 (d, *J* = 2.5 Hz, 1H), 7.66 (dd, *J* = 8.8, 2.6 Hz, 1H), 7.33-7.30 (m, 2H), 7.28-7.25 (m, 3H), 7.17 (d, *J* = 8.7 Hz, 1H), 3.94 (s, 3H).

^{13}C NMR (126 MHz, CD_3CN): δ 189.56, 162.76, 141.12, 137.61, 132.88, 130.70, 130.37, 127.95, 127.02, 126.49, 114.95, 57.00.

HRMS (EI) *m/z* calcd for $C_{14}H_{12}O_2S$ $[M]^+$: 244.0558, found 244.0547.

(3,4-dimethoxyphenyl)(phenyl)sulfane (**D5**)

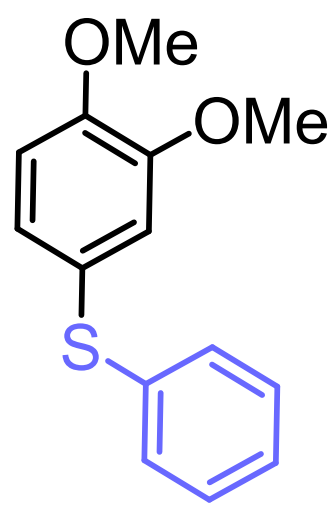

The concentrated mixture was purified by chromatography on silica gel with PE and EA (100:0 to 91:9, *v/v*) as eluent to afford an orange liquid **D5** (31.4 mg, 64%).

^{1}H NMR (500 MHz, CD_3CN): δ 7.28 (t, *J* = 7.6 Hz, 2H), 7.21-7.17 (m, 3H), 7.05-7.03 (m, 2H), 6.95 (d, *J* = 8.0 Hz, 1H), 3.81 (s, 3H), 3.76 (s, 3H).

^{13}C NMR (126 MHz, CD_3CN): δ 150.82, 150.75, 139.18, 130.13, 129.26, 127.52, 127.08, 125.10, 117.85, 113.36, 56.49, 56.44.

The NMR spectra are matching with the reference.[24]

[1,1'-biphenyl]-4-yl(phenyl)sulfane (**D6**)

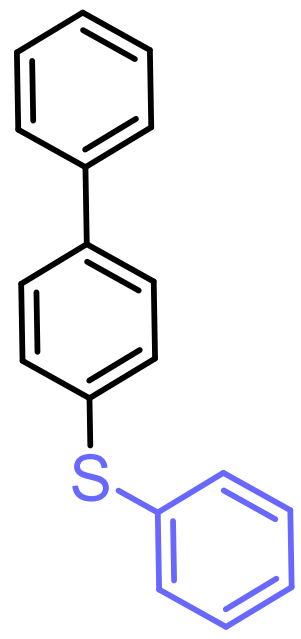

The concentrated mixture was purified by chromatography on silica gel with PE as eluent to afford a white solid **D6** (39.9 mg, 76%).

^{1}H NMR (500 MHz, CD_3CN): δ 7.63 (t, J = 8.1 Hz, 4H), 7.46 (t, J = 7.6 Hz, 2H), 7.41-7.32 (m, 8H).

^{13}C NMR (126 MHz, CD_3CN): δ 140.84, 140.81, 136.23, 135.89, 132.23, 132.18, 130.48, 129.95, 128.82, 128.67, 128.52, 127.77.

The NMR spectra are matching with the reference.[25]

methyl 2-methoxy-5-(pyridin-2-ylthio)benzoate (**D7**)

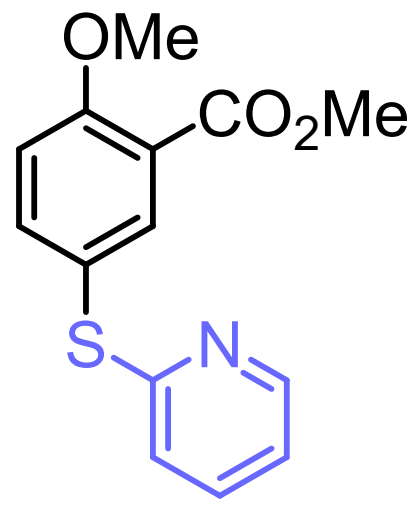

The concentrated mixture was purified by chromatography on silica gel with PE and EA (95:5 to 66:34, *v/v*) as eluent to afford a white solid **D7** (31.1 mg, 57%).

^{1}H NMR (500 MHz, $CDCl_3$): δ 8.40 (d, *J* = 4.5 Hz, 1H), 8.04 (d, *J* = 2.3 Hz, 1H), 7.71 (dd, *J* = 8.6, 2.4 Hz, 1H), 7.45 (td, *J* = 7.9, 1.7 Hz, 1H), 7.05 (d, *J* = 8.7 Hz, 1H), 6.98 (dd, *J* = 7.2, 5.0 Hz, 1H), 6.83 (d, *J* = 8.0 Hz, 1H), 3.95 (s, 3H), 3.88 (s, 3H).

^{13}C NMR (126 MHz, $CDCl_3$): δ 165.64, 161.61, 159.99, 149.47, 140.88, 139.00, 136.77, 121.29, 121.13, 120.69, 119.77, 113.24, 56.19, 52.18.

The NMR spectra are matching with the reference.[26]

methyl 2-methoxy-5-(thiophen-2-ylthio)benzoate (**D8**)

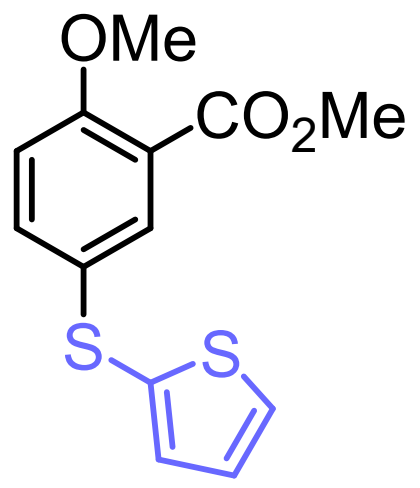

The concentrated mixture was purified by chromatography on silica gel with PE and EA (98:2 to 82:18, *v/v*) as eluent to afford a colorless liquid **D8** (17.5 mg, 31%).

^{1}H NMR (500 MHz, $CDCl_3$): δ 7.78 (d, *J* = 2.5 Hz, 1H), 7.41 (d, *J* = 1.5 Hz, 1H), 7.40 (dd, *J* = 3.0, 1.8 Hz, 1H), 7.24 (d, *J* = 3.1 Hz, 1H), 7.02 (dd, *J* = 5.4, 3.5 Hz, 1H), 6.90 (d, *J* = 8.7 Hz, 1H), 3.87 (s, 6H).

^{13}C NMR (126 MHz, $CDCl_3$): δ 165.95, 158.28, 134.61, 134.52, 133.01, 132.68, 130.59, 128.56, 127.77, 120.68, 112.91, 56.19, 52.17.

HRMS (EI) *m/z* calcd for $C_{13}H_{12}O_3S_2$ $[M]^+$: 280.0228, found 280.0222.

methyl 2-methoxy-5-(methylthio)benzoate (**D9**)

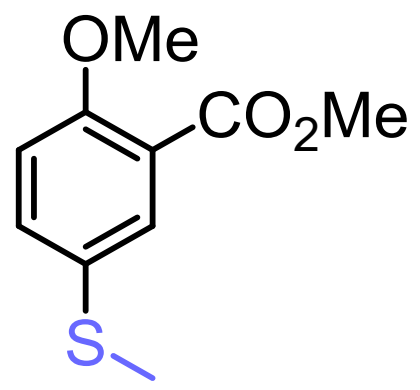

The concentrated mixture was purified by chromatography on silica gel with PE and EA (100:0 to 82:18, *v/v*) as eluent to afford a colorless liquid **D9** (16.6 mg, 39%).

^{1}H NMR (500 MHz, $CDCl_3$): δ 7.75 (d, *J* = 2.4 Hz, 1H), 7.42 (dd, *J* = 8.7, 2.4 Hz, 1H), 6.93 (d, *J* = 8.7 Hz, 1H), 3.89 (d, *J* = 1.7 Hz, 6H), 2.46 (s, 3H).

^{13}C NMR (126 MHz, $CDCl_3$): δ 166.19, 157.52, 133.68, 131.60, 128.91, 120.64, 112.85, 56.22, 52.15, 17.68.

The NMR spectra are matching with the reference.[27]

ethyl 2-(4-chloro-2-(phenylthio)phenoxy)-2-methylpropanoate (**D10**)

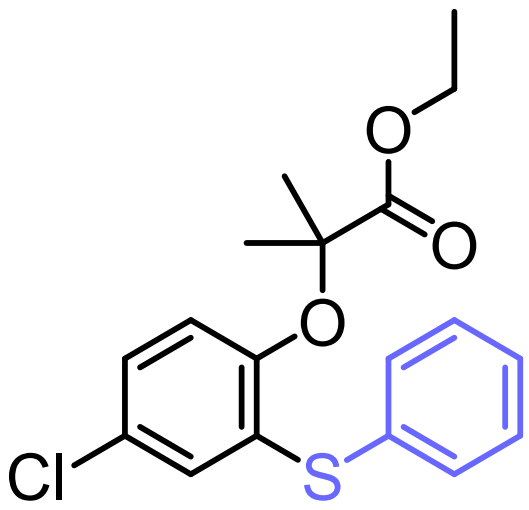

The concentrated mixture was purified by chromatography on silica gel with PE and EA (100:0 to 97:3, *v/v*) as eluent to afford a yellow liquid **D10** (21.1 mg, 30%).

^{1}H NMR (500 MHz, $CDCl_3$): δ 7.44 (d, *J* = 7.6 Hz, 2H), 7.39-7.33 (m, 3H), 7.00 (d, *J* = 8.9 Hz, 1H), 6.85 (s, 1H), 6.69 (d, *J* = 8.4 Hz, 1H), 4.25 (q, *J* = 7.2 Hz, 2H), 1.57 (s, 6H), 1.27 (t, *J* = 7.1 Hz, 3H).

^{13}C NMR (126 MHz, $CDCl_3$): δ 173.96, 151.13, 133.39, 132.58, 131.81, 129.46, 128.89, 128.24, 127.56, 126.33, 118.28, 80.50, 61.56, 24.93, 14.09.

HRMS (EI) *m/z* calcd for $C_{18}H_{19}ClO_3S$ $[M]^+$: 350.0743, found 350.0735.

methyl 5-(2,5-dimethyl-4-(phenylthio)phenoxy)-2,2-dimethylpentanoate (**D11**)

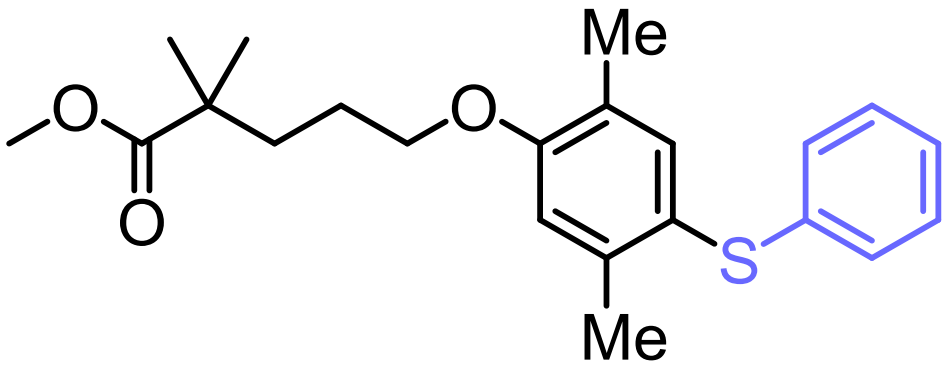

The concentrated mixture was purified by chromatography on silica gel with PE and EA (100:0 to 95:5, *v/v*) as eluent to afford a yellow oil **D11** (55.0 mg, 74%).

^{1}H NMR (500 MHz, $CDCl_3$): δ 7.29 (s, 1H), 7.19 (t, *J* = 7.7 Hz, 2H), 7.08 (t, *J* = 7.4 Hz, 1H), 7.01 (d, *J* = 7.7 Hz, 2H), 6.72 (s, 1H), 3.95 (t, *J* = 5.5 Hz, 2H), 3.67 (s, 3H), 2.33 (s, 3H), 2.17 (s, 3H), 1.79-1.71 (m, 4H), 1.23 (s, 6H).

^{13}C NMR (126 MHz, $CDCl_3$): δ 178.17, 157.87, 141.09, 138.91, 138.20, 128.75, 126.46, 125.34, 124.81, 121.17, 113.10, 67.97, 51.69, 42.04, 37.01, 25.15, 25.07, 20.84, 15.49.

HRMS (EI) *m/z* calcd for $C_{22}H_{28}O_3S$ $[M]^+$: 372.1759, found 372.1747.

Hydromethylthiolation reaction

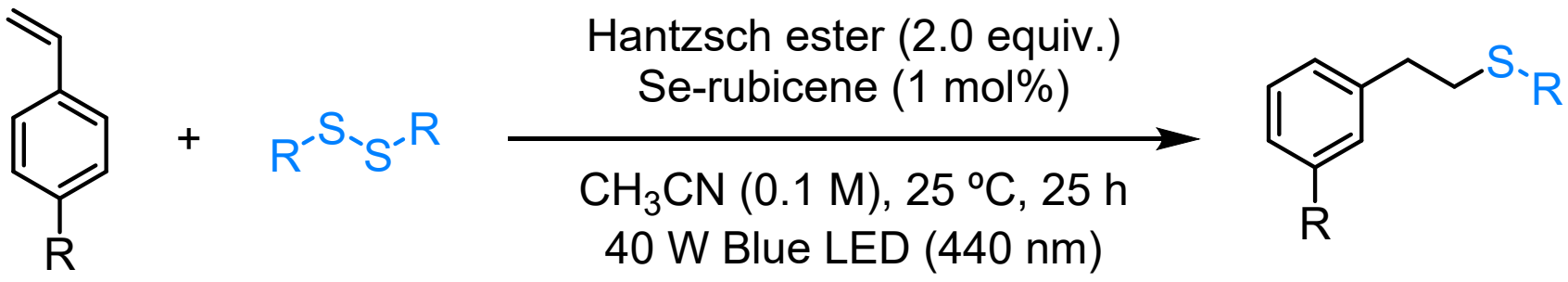

General Procedure:

In a glove-box under nitrogen atmosphere, the styrene (1.0 equiv.) was added to a solution of the 2,6-dimethyl-1,4-dihydro-pyridine-3,5-dicarboxylic acid di-tert-butyl ester (Hantzsch ester) (2.0 equiv.), ArSSAr (5.0 equiv.) and Se-rubicene (1 mol%) in anhydrous CH_3CN (0.1 M). After the vial was sealed and removed from glove-box, the resulting solution was irradiated with a kessil 40W blue LED (440 nm) lamp at 25 °C for 25 h. Finally, the reaction mixture was concentrated in vacuo and purified by column chromatography (dry-loaded into silica-gel, eluent as specified for individual compounds).

phenethyl(phenyl)sulfane (**E1**)

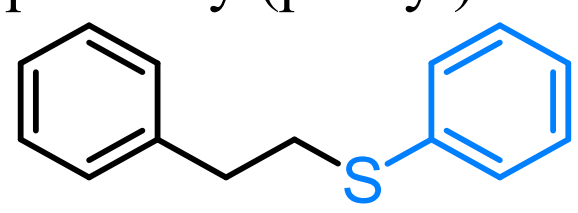

The concentrated mixture was purified by chromatography on silica gel with PE as eluent to afford a colorless liquid **E1** (37.0 mg, 87%).

^{1}H NMR (500 MHz, CD_3CN): δ 7.37-7.29 (m, 6H), 7.25-7.19 (m, 4H), 3.21 (t, *J* = 7.5 Hz, 2H), 2.91 (t, *J* = 8.0 Hz, 2H).

^{13}C NMR (126 MHz, CD_3CN): δ 141.41, 137.50, 130.01, 129.59, 129.48, 129.39, 127.33, 126.74, 35.96, 35.13.

The NMR spectra are matching with the reference.[28]

(4-fluorophenethyl)(phenyl)sulfane (**E2**)

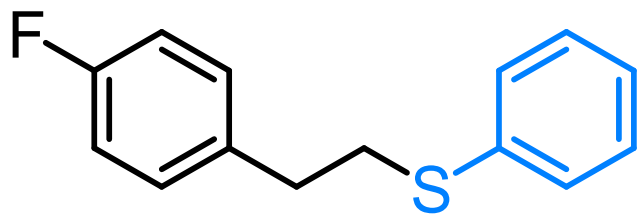

The concentrated mixture was purified by chromatography on silica gel with PE as eluent to afford a colorless liquid **E2** (34.7 mg, 75%).

^{1}H NMR (500 MHz, CD_3CN): δ 7.36-7.30 (m, 4H), 7.25-7.19 (m, 3H), 7.03 (t, *J* = 8.4 Hz, 2H), 3.18 (t, *J* = 7.6 Hz, 2H), 2.89 (t, *J* = 7.6 Hz, 2H).

^{13}C NMR (126 MHz, CD_3CN): δ 163.41, 161.48, 137.38 (d, *J* = 2.5 Hz), 131.33 (d, *J* = 7.8 Hz), 130.02, 129.54, 126.79, 115.90 (d, *J* = 21.2 Hz), 35.17, 35.09.

^{19}F NMR (471 MHz, CD_3CN): δ -118.50 (d, *J* = 7.8 Hz).

The NMR spectra are matching with the reference.[29]

phenyl(4-(trifluoromethyl)phenethyl)sulfane (**E3**)

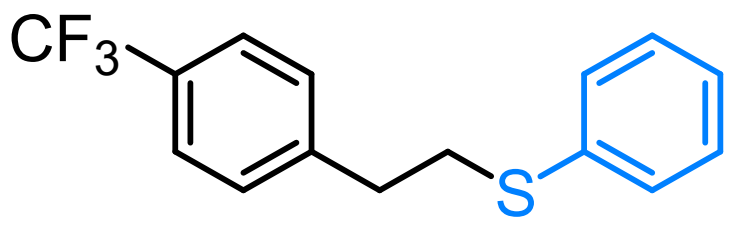

The concentrated mixture was purified by chromatography on silica gel with PE as eluent to afford a colorless liquid **E3** (54.8 mg, 97%).

^{1}H NMR (500 MHz, CD_3CN): δ 7.61 (d, *J* = 8.1 Hz, 2H), 7.41 (d, *J* = 8.0 Hz, 2H), 7.37-7.30 (m, 4H), 7.23-7.19 (m, 1H), 3.24 (t, *J* = 7.4 Hz, 2H), 2.99 (t, *J* = 7.5 Hz, 2H).

^{13}C NMR (126 MHz, CD_3CN): δ 146.05, 137.10, 130.36, 130.05, 129.73, 129.01, 128.75, 126.93, 126.29-125.94 (m), 35.67, 34.73.

^{19}F NMR (471 MHz, CD_3CN): δ -62.75.

The NMR spectra are matching with the reference.[29]

(4-methoxyphenethyl)(phenyl)sulfane (**E4**)

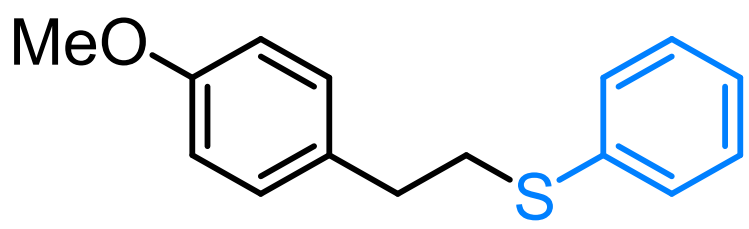

The concentrated mixture was purified by chromatography on silica gel with PE and EA (100:0 to 94:6, *v/v*) as eluent to afford a red liquid **E4** (45.9 mg, 94%).

^{1}H NMR (500 MHz, CD_3CN): δ 7.36-7.30 (m, 4H), 7.19 (t, *J* = 7.2 Hz, 1H), 7.15 (d, *J* = 8.6 Hz, 2H), 6.85 (d, *J* = 8.6 Hz, 2H), 3.75 (s, 3H), 3.17 (t, *J* = 7.5 Hz, 2H), 2.84 (t, *J* = 8.0 Hz, 2H).

^{13}C NMR (126 MHz, CD_3CN): δ 159.28, 137.61, 133.33, 130.58, 129.99, 129.39, 126.66, 114.71, 55.80, 35.35, 35.08.

The NMR spectra are matching with the reference.[29]

(3,5-dichlorophenyl)(phenethyl)sulfane (**E5**)

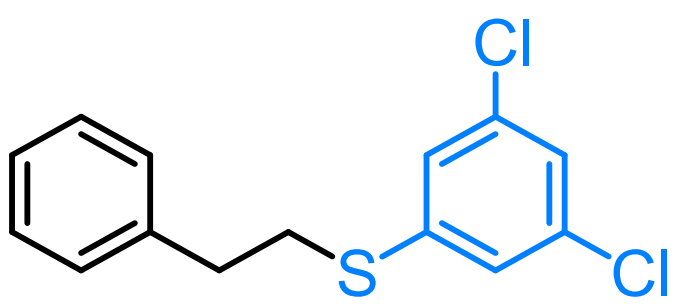

The concentrated mixture was purified by chromatography on silica gel with PE as eluent to afford a colorless oil **E5** (54.4 mg, 96%).

^{1}H NMR (500 MHz, CD_3CN): δ 7.30 (t, *J* = 7.5 Hz, 2H), 7.25-7.21 (m, 6H), 3.26 (t, *J* = 7.5 Hz, 2H), 2.93 (t, *J* = 7.5 Hz, 2H).

^{13}C NMR (126 MHz, CD_3CN): δ 142.27, 140.87, 135.89, 129.62, 129.41, 127.47, 126.57, 126.08, 35.47, 34.52.

HRMS (EI) *m/z* calcd for $C_{14}H_{12}Cl_2S$ $[M]^+$: 282.0037, found 282.0028.

2-(phenethylthio)thiophene (**E6**)

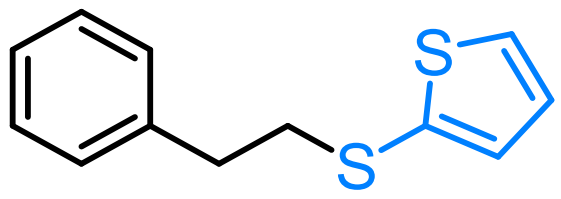

The concentrated mixture was purified by chromatography on silica gel with PE as eluent to afford a yellow liquid **E6** (27.9 mg, 64%).

^{1}H NMR (500 MHz, CD_3CN): δ 7.48-7.47 (m, 1H), 7.31-7.28 (m, 2H), 7.23-7.20 (m, 3H), 7.17-7.16 (m, 1H), 7.04 (dd, *J* = 5.4, 3.5 Hz, 1H), 3.06 (dd, *J* = 8.4, 6.8 Hz, 2H), 2.88 (t, *J* = 8.0 Hz, 2H).

^{13}C NMR (126 MHz, CD_3CN): δ 141.14, 135.14, 134.57, 130.52, 129.65, 129.40, 128.85, 127.31, 40.65, 36.26.

The NMR spectra are matching with the reference.[30]

III. Optimization studies

Table S1. Optimization of the trifluoromethylation of biphenylsulfonium salts.

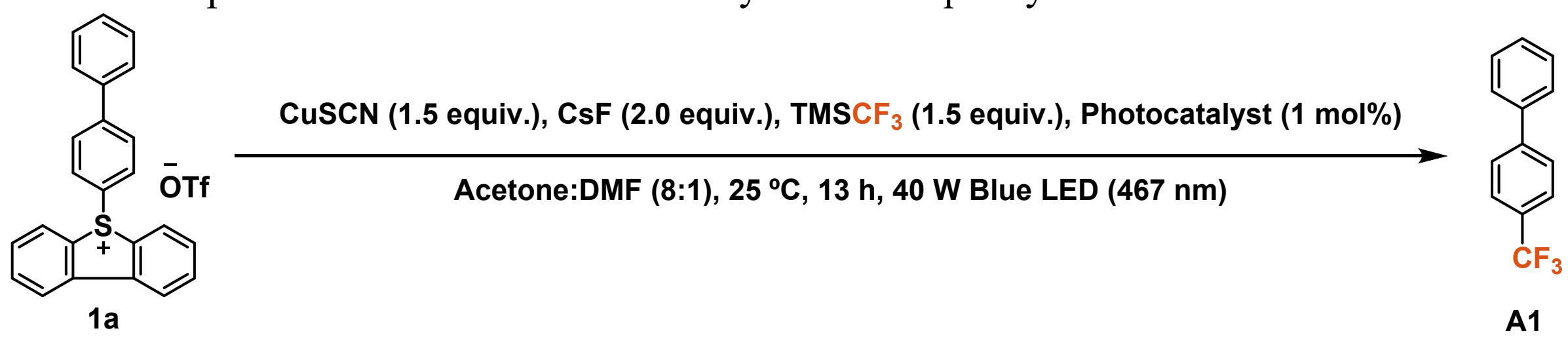

Entry	Photocatalyst	Yield[a]
1	S-rubicene (1 mol%)	87
2	Methylene blue trihydrate (1 mol%)	51
3	Mes-Acr-Me$^+$ClO$_4^-$ (1 mol%)	36
4	Eosin Y (1 mol%)	44
5	4CzIPN (1 mol%)	52

[a]: Unless otherwise noted, a mixture of (a solution of CuSCN (0.15 mmol, 1.5 equiv.), CsF (0.2 mmol, 2.0 equiv.), and $TMSCF_3$ (0.15 mmol, 1.5 equiv.) in DMF (0.5 mL), biphenylsulfonium salts(1a) (0.10 mmol, 1.0 equiv.), photocatalyst (1 mol%) in acetone (4.0 mL), irradiated with a blue LED (467 nm) lamp at 25 °C for 13h. Isolated yields was gaven.

Table S2. Optimization of the arylation of aryl sulfonium salts.

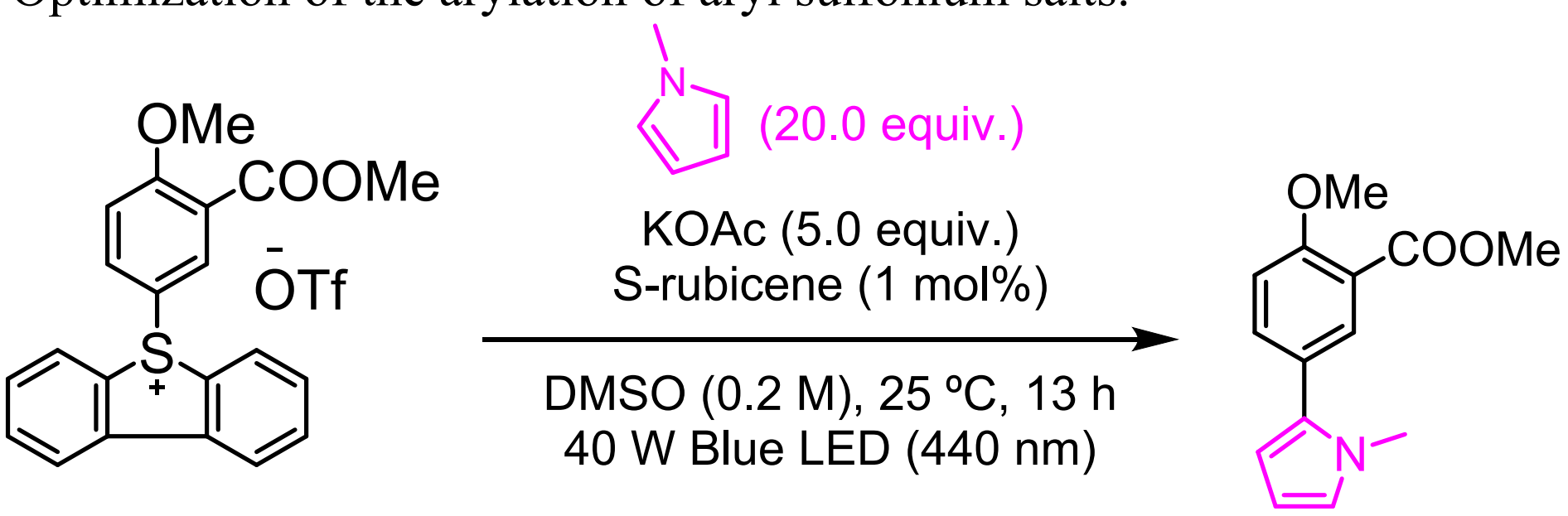

Entry	N-Methyl pyrrole	Photocatalyst	*hv* (nm)	Yield[a]
1	20.0 equiv.	S-rubicene (1 mol%)	440	69
2	20.0 equiv.	rubicene (1 mol%)	390	69

3	20.0 equiv.	Ben-rubicene (1 mol%)	427	50
4	20.0 equiv.	Methylene blue trihydrate (1 mol%)	440	63
5	20.0 equiv.	Mes-Acr-$Me^{+}ClO_4^{-}$ (1 mol%)	440	61
6	20.0 equiv.	Eosin Y (1 mol%)	440	44
7	20.0 equiv.	4CzIPN (1 mol%)	440	44
8[b]	20.0 equiv.	S-rubicene (1 mol%)	-	0
9	20.0 equiv.	-	440	60
10	10.0 equiv.	S-rubicene (1 mol%)	440	38
11	5.0 equiv.	S-rubicene (1 mol%)	440	24
12[c]	20.0 equiv.	S-rubicene (1 mol%)	440	45
13[c]	20.0 equiv.	-	440	25

[a]: Unless otherwise noted, a mixture of aryl sulfonium salts (0.05 mmol, 1.0 equiv.), N-Methyl pyrrole (1.0 mmol, 20.0 equiv.), photocatalyst (1 mol%), KOAc (5.0 equiv.) and DMSO (0.25 mL) was irradiated with a kessil 40W blue LED lamp at 25 °C for 13 h. Isolated yields were given; [b]: In the darkness. [c]: 5-(4-Isopropylphenyl)-5*H*-dibenzo[*b,d*]thiophen-5-ium trifluoromethanesulfonate was used.

Table S3. Optimization of the borylation of aryl sulfonium salts.

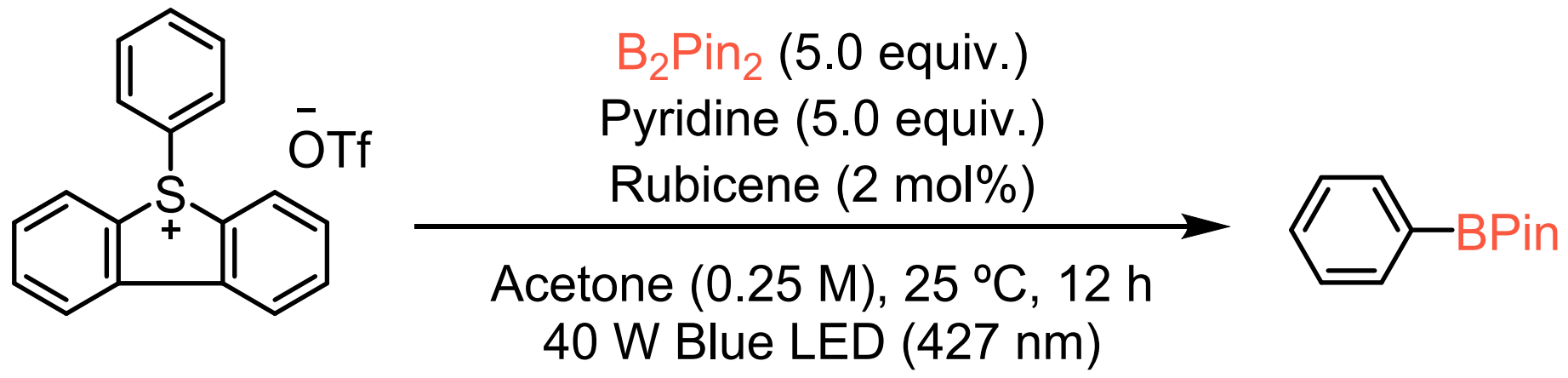

Entry	B_2Pin_2	Photocatalyst	*hv* (nm)	GC-MS (A %)[a]
1	5.0 equiv.	O-rubicene (2 mol%)	456	82
2	5.0 equiv.	rubicene (2 mol%)	427	78
3	5.0 equiv.	Ben-rubicene (2 mol%)	427	71
4[b]	5.0 equiv.	rubicene (2 mol%)	-	0
5	5.0 equiv.	-	427	66
6	2.0 equiv.	rubicene (2 mol%)	427	21
7	1.0 equiv.	rubicene (2 mol%)	427	15

[a] : Unless otherwise noted, a mixture of aryl sulfonium salts (0.05 mmol, 1.0 equiv.), B_2Pin_2 (0.25 mmol, 5.0 equiv.), photocatalyst (2 mol%), pyridine (5.0 equiv.) and acetone (0.20 mL) was irradiated with a kessil 40W blue LED lamp at 25 °C for 12 h. GC-MS area ratio was obtained using anthracene as an internal standard; [b]: In the darkness.

Table S4. Optimization of the borylation of aryl sulfonium salts.

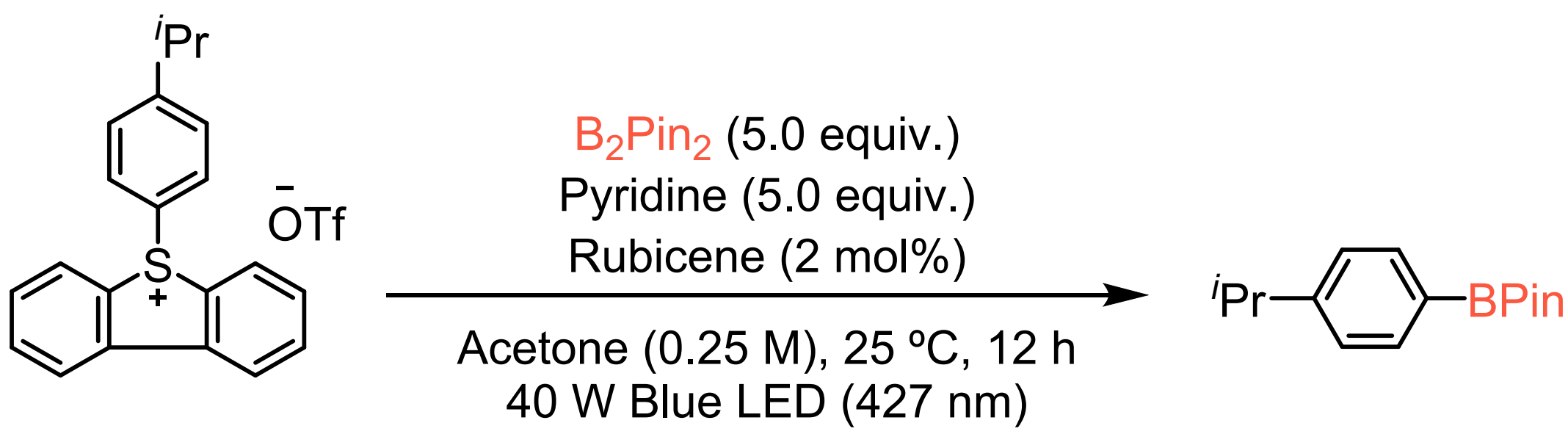

Entry	B_2Pin_2	Photocatalyst	*hv* (nm)	GC-MS (A %)[a]
1	5.0 equiv.	rubicene (2 mol%)	427	70
2	5.0 equiv.	Methylene blue trihydrate (2 mol%)	427	95
3	5.0 equiv.	Mes-Acr-$Me^{+}ClO_4^{-}$ (2 mol%)	427	77
4	5.0 equiv.	Eosin Y (2 mol%)	427	83

5	5.0 equiv.	4CzIPN (2 mol%)	427	87

[a] : Unless otherwise noted, a mixture of aryl sulfonium salts (0.05 mmol, 1.0 equiv.), B_2Pin_2 (0.25 mmol, 5.0 equiv.), photocatalyst (2 mol%), pyridine (5.0 equiv.) and acetone (0.20 mL) was irradiated with a kessil 40W blue LED lamp at 25 °C for 12 h. GC-MS area ratio was obtained using anthracene as an internal standard. Isolated yields were given.

Table S5. Optimization of the thioetherification of aryl sulfonium salts.

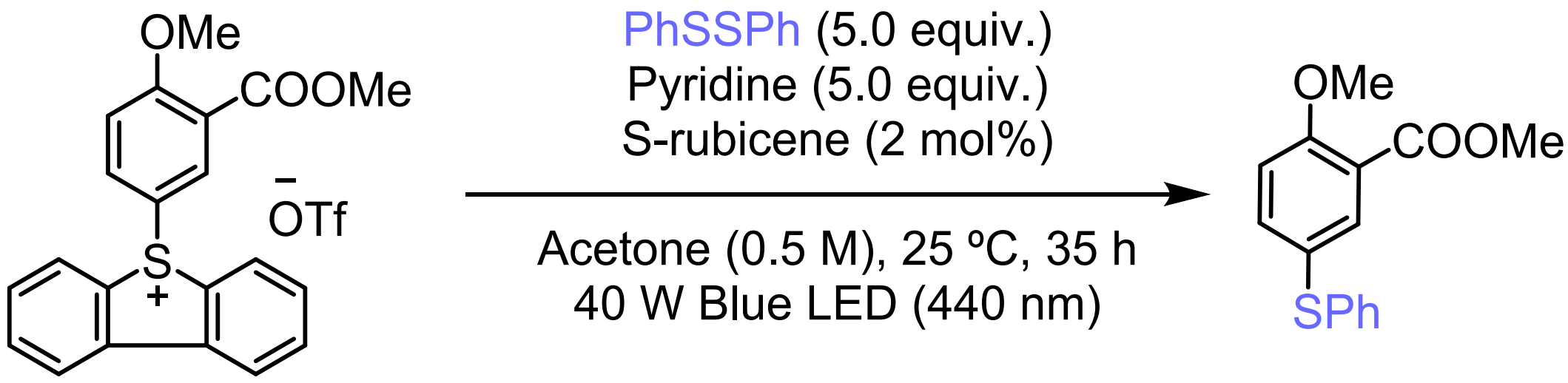

Entry	Photocatalyst	*hv* (nm)	Yield[a]
1	S-rubicene (2 mol%)	440	92
2	Ben-rubicene (2 mol%)	427	35
3	rubicene (2 mol%)	390	25
4	Methylene blue trihydrate (2 mol%)	440	32
5	Mes-Acr-Me$^+$ClO$_4^-$ (2 mol%)	440	4
6	Eosin Y (2 mol%)	440	9
7	4CzIPN (2 mol%)	440	21
8[b]	S-rubicene (2 mol%)	-	0
9	-	440	16

[a]: Unless otherwise noted, a mixture of aryl sulfonium salts (0.05 mmol, 1.0 equiv.), PhSSPh (0.25 mmol, 5.0 equiv.), photocatalyst (2 mol%), pyridine (5.0 equiv.) and acetone (0.10 mL) was irradiated with a kessil 40W blue LED lamp at 25 °C for 13 h. Isolated yields were given; [b]: In the darkness.

Table S6. Optimization of the hydromethylthiolation reaction.

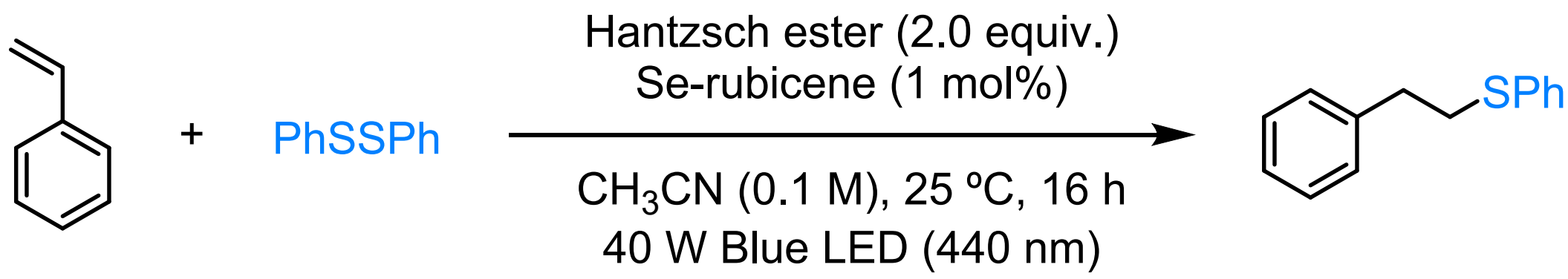

Entry	PhSSPh (equiv.)	Photocatalyst	*hv* (nm)	Additive (2.0 equiv.)	Yield[a]
1	2.0	S-rubicene (1 mol%)	440	-	20
2	2.0	O-rubicene (1 mol%)	440	-	23
3	2.0	Se-rubicene (1 mol%)	440	-	28
4	5.0	Se-rubicene (1 mol%)	440	-	32
5	5.0	Se-rubicene (1 mol%)	440	Hantzsch esters	87
6	5.0	Methylene blue trihydrate (1 mol%)	440	Hantzsch esters	46
7	5.0	Mes-Acr-Me$^+$ClO$_4^-$ (1 mol%)	440	Hantzsch esters	62
8	5.0	Eosin Y (1 mol%)	440	Hantzsch esters	52
9	5.0	4CzIPN (1 mol%)	440	Hantzsch esters	93
10	5.0	-	440	Hantzsch esters	70
11[b]	5.0	Se-rubicene (1 mol%)	-	Hantzsch esters	0
12[c]	5.0	Se-rubicene (1 mol%)	440	Hantzsch esters	64
13[c]	5.0	-	440	Hantzsch esters	8

[a] : Unless otherwise noted, a mixture of styrene (0.05 mmol, 1.0 equiv.), PhSSPh, photocatalyst (1 mol%), 2,6-dimethyl-1,4-dihydro-pyridine-3,5-dicarboxylic acid di-tert-butyl ester (Hantzsch esters) (2.0 equiv.)

and MeCN (0.50 mL) was irradiated with a kessil 40W blue LED lamp at 25 °C for 10 h. Isolated yields were given; [b]: In the darkness. [c]: Dithiophene disulfide was used.

IV. Mechanistic studies

IV.1. Fluorescence Quenching Experiments

Visible light luminescence intensities were recorded using an Edinburgh Instruments FLS-1000 spectrofluorometer. All luminescence measurements were recorded using a screw-top quartz cuvette (Hellma fluorescence quartz cuvette, 10 x 10 mm, 3.5 mL). Solutions of S-rubicene, 5-(4-methoxy-3-(methoxycarbonyl)phenyl)-5H-dibenzo[*b,d*]thiophen-5-ium trifluoromethanesulfonate, dibenzo[*b,d*]thiophene (DBT) were prepared in a mixture of DMF/acetone (*v*/*v*: 1:1), and solution of [$CuCF_3$] was prepared in DMF in a nitrogen-filled glovebox, and then was diluted with acetone to prepare DMF/acetone (*v*/*v*: 1:1) solution for measurement. The solutions were transferred to the screw-top cuvette inside the glovebox. Then the cuvette was sealed, and brought out of the glovebox for visible light luminescence measurements.

In a typical procedure, 5-(4-methoxy-3-(methoxycarbonyl)phenyl)-5*H*-dibenzo[*b,d*]thiophen-5-ium trifluoromethanesulfonate (12.7 mg, 0.025 mmol) was dissolved and diluted to a final volume of 10.0 mL (*c* = 2.5 mM of aryl sulfonium salt solution) with a stock solution of S-rubicene in DMF/acetone (*v*/*v*: 1:1) (*c* = 100 μM). The solution was then serially diluted with a 100 μM stock solution of S-rubicene. All solutions were excited at 465 nm and the emission was measured from 490 to 800 nm.

Quenching was analyzed by plotting I_0/I according to the Stern-Volmer relationship: $I_0/I = k_q\tau_0[Q]+1$ where I_0 represents the integral of the luminescence over the range of 490 to 800 nm in the absence of a quencher, I is the integral of luminescence over the range of 490 to 800 nm in the presence of a quencher, k_q represents the quenching rate constant, [Q] is the concentration of a given quencher, and τ_0 is the excited state lifetime of the emissive photocatalyst in the absence of quencher. The excited state lifetime of S-rubicene in acetone is 1.7 ns. Note: As we performed the experiment in a DMF/acetone mixture, the lifetime of S-rubicene was used approximately to calculate quenching rate constant.

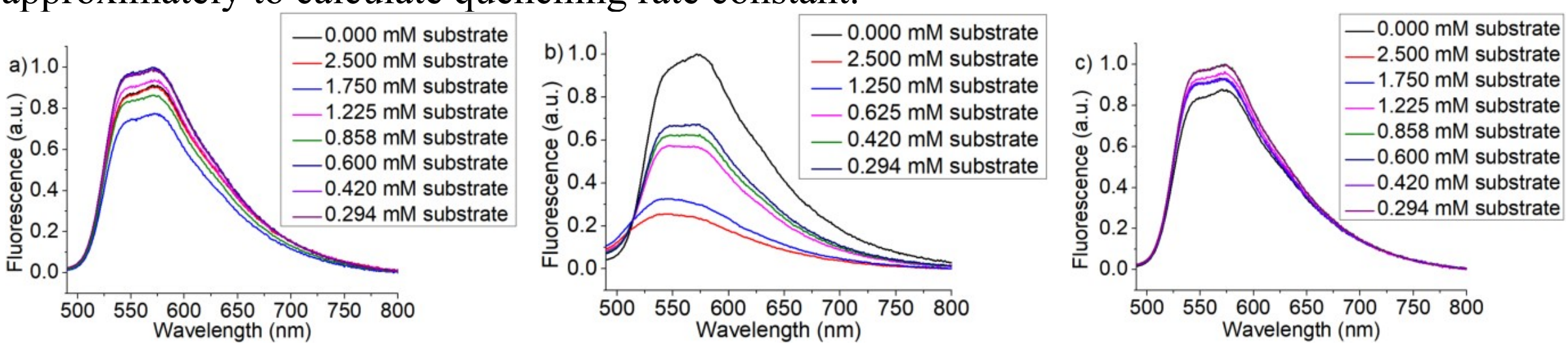

Figure S1. Emission spectra of S-rubicene in DMF/acetone (*v*/*v*: 1:1) (100 μM) with varying concentrations of [$CuCF_3$] (**a**), aryl sulfonium salt (**b**), DBT (**c**) normalized to S-rubicene with no [$CuCF_3$], aryl sulfonium salt, DBT added.

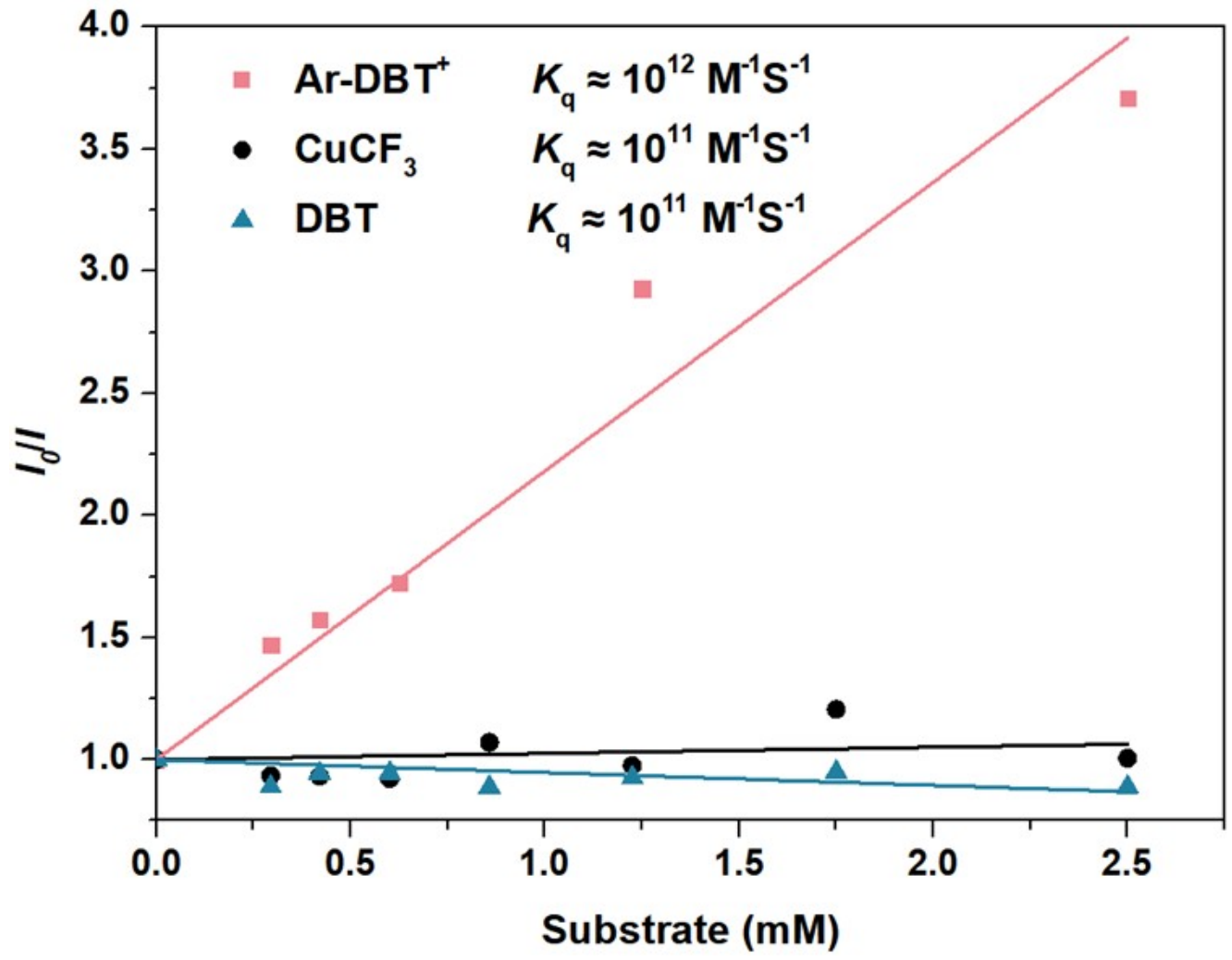

Figure S2. Stern-Volmer plot for S-rubicene in DMF/acetone (*v*/*v*: 1:1) (100 μM) with varying [$CuCF_3$], aryl sulfonium salt, DBT.

V. Materials characterization

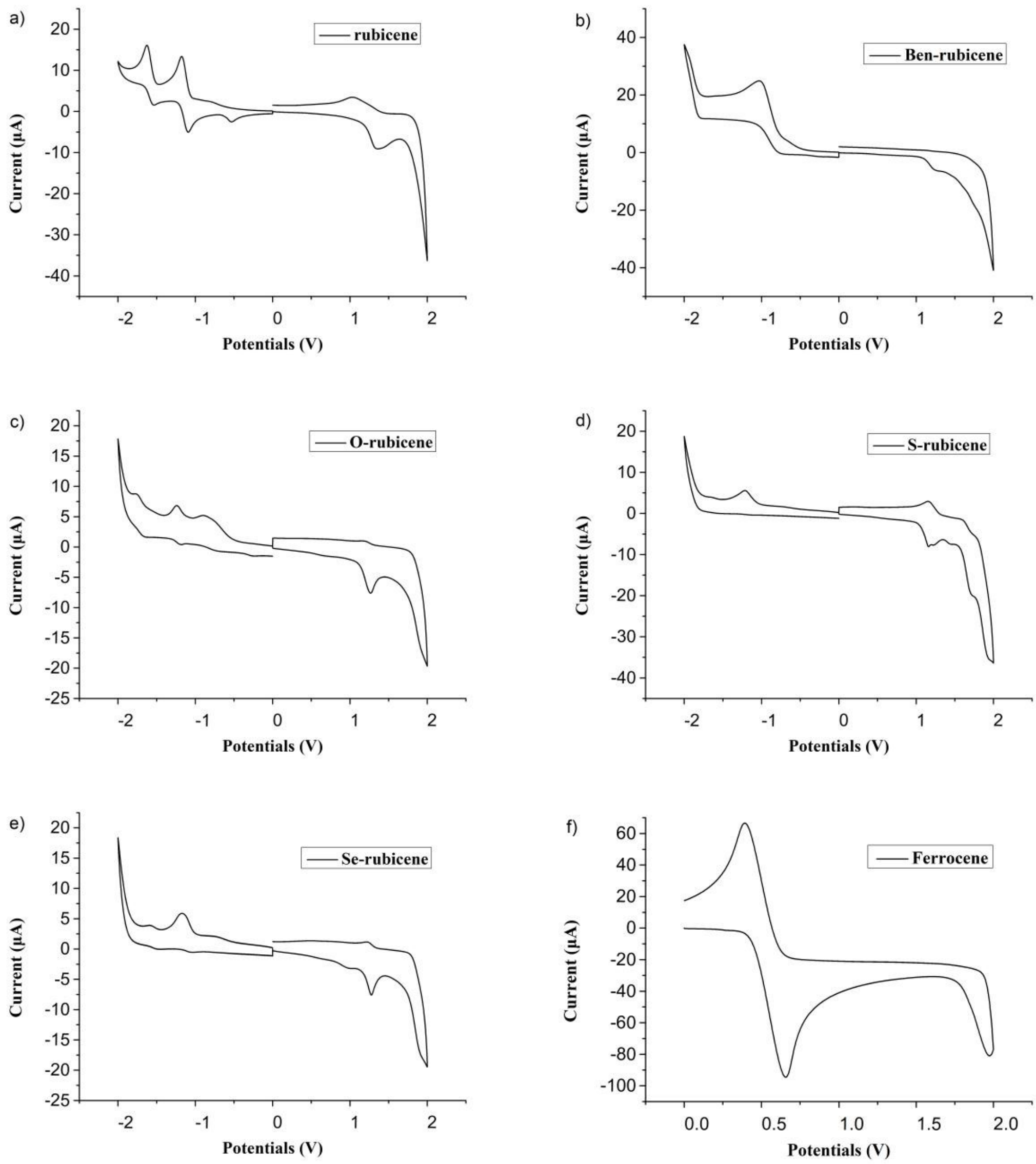

Figure S3. The cyclic voltammograms (CV) of rubicene (**a**), Ben-rubicene (**b**), O-rubicene (**c**), S-rubicene (**d**), Se-rubicene (**e**) and ferrocenium/ferrocene (**f**) in CH_2Cl_2/0.1 M $[^nBu_4N]^+[PF_6]^-$.

Table S7. Redox potentials of rubicene and common visible light photocatalysts.[a]

Photocatalyst	$E_{1/2}(P^+/^*P)$ (V)	$E_{1/2}(^*P/P^-)$ (V)	$E_{1/2}(P^+/P)$ (V)	$E_{1/2}(P/P^-)$ (V)	Excitation λ_{max} (nm)	Emission λ_{max} (nm)
rubicene	-1.58	0.79	0.73	-1.52	529	558
Ben-rubicene	-2.34	1.75	0.65	-1.24	413	424
O-rubicene	-1.75	1.45	0.67	-0.97	502	538
S-rubicene	-1.80	0.91	0.61	-1.50	500	544
Se-rubicene	-1.63	0.89	0.72	-1.46	513	558

Photocatalyst	τ_f (ns)	τ_T (μs)	ϕ_f (%)	ϕ_{ISC} (%)	E_{0-0}[b] (eV)
rubicene	4.2	20.4	9.0	23	2.31
Ben-rubicene	4.9	19.5	17.9	44	2.99
O-rubicene	2.0	52.6	1.6	40	2.42
S-rubicene	1.7	30.3	1.4	78	2.41
Se-rubicene	1.8	13.1	0.1	90	2.35

[a]All potentials are given in volts versus the saturated calomel electrode (SCE). Measurements were performed in acetonitrile at room temperature unless otherwise noted. [b]E_{0-0}, the zero-zero vibrational state excitation energy, was estimated using the medium wavelengths between the lowest fluorescence excitation peak (excitation λ_{max}) and the fluorescence peak (emission λ_{max}) and was used to calculate $E_{1/2}(P^+/^*P) = E_{1/2}(P^+/P) - E_{0-0}$ and $E_{1/2}(^*P/P^-) = E_{0-0} + E_{1/2}(P/P^-)$.

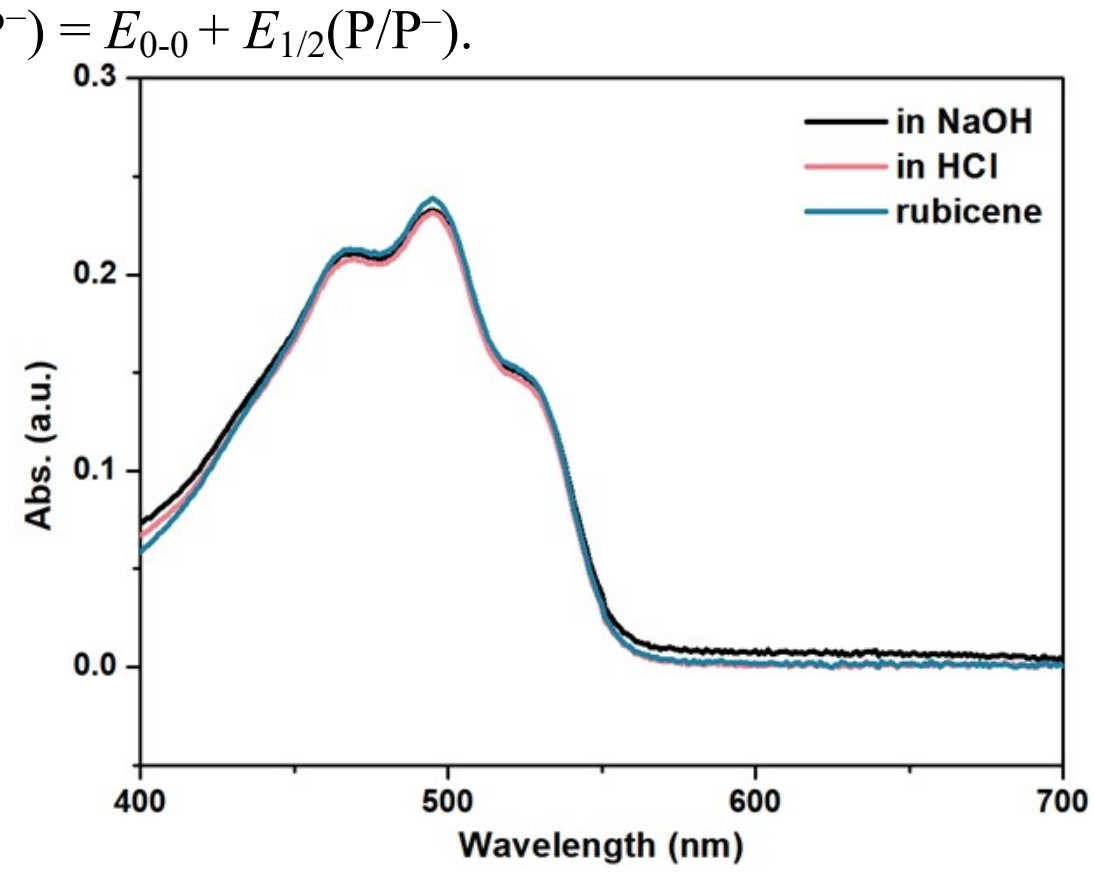

Figure S4. The UV-vis absorption of rubicene in acid or base in acetone (0.25 M) after 6h (Raw data).

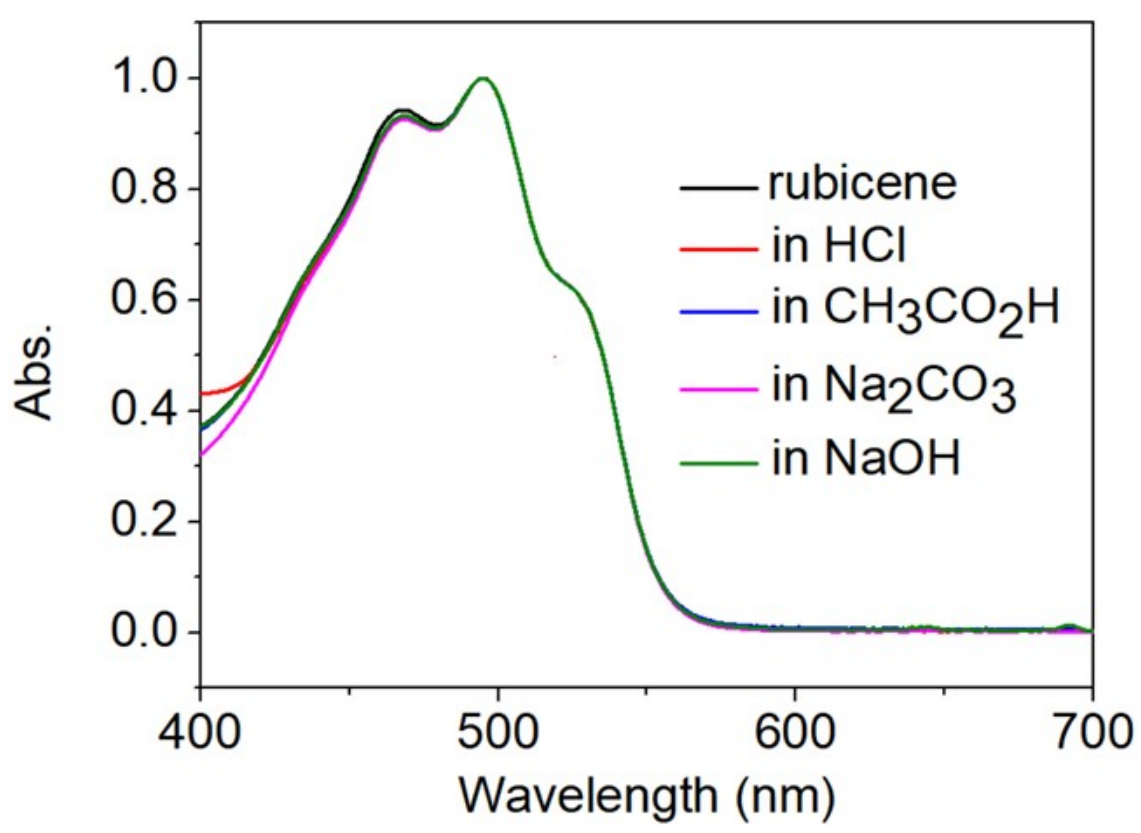

Figure S5. The UV-vis absorption of rubicene in acid or base in acetone (0.25 M) after 6h.

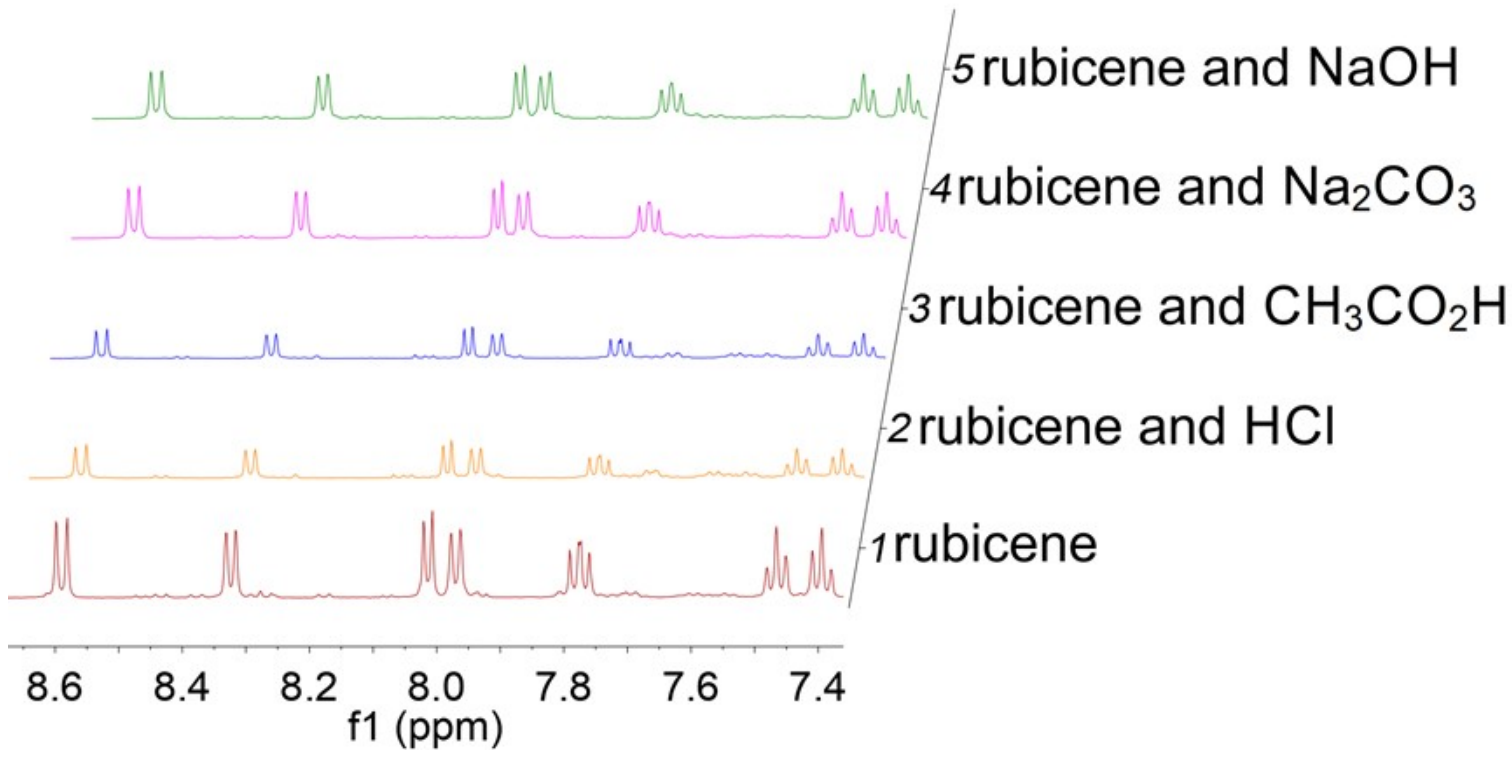

Figure S6. ^{1}H-NMR of rubicene in acid or base in acetone (0.25 M) after 6h.

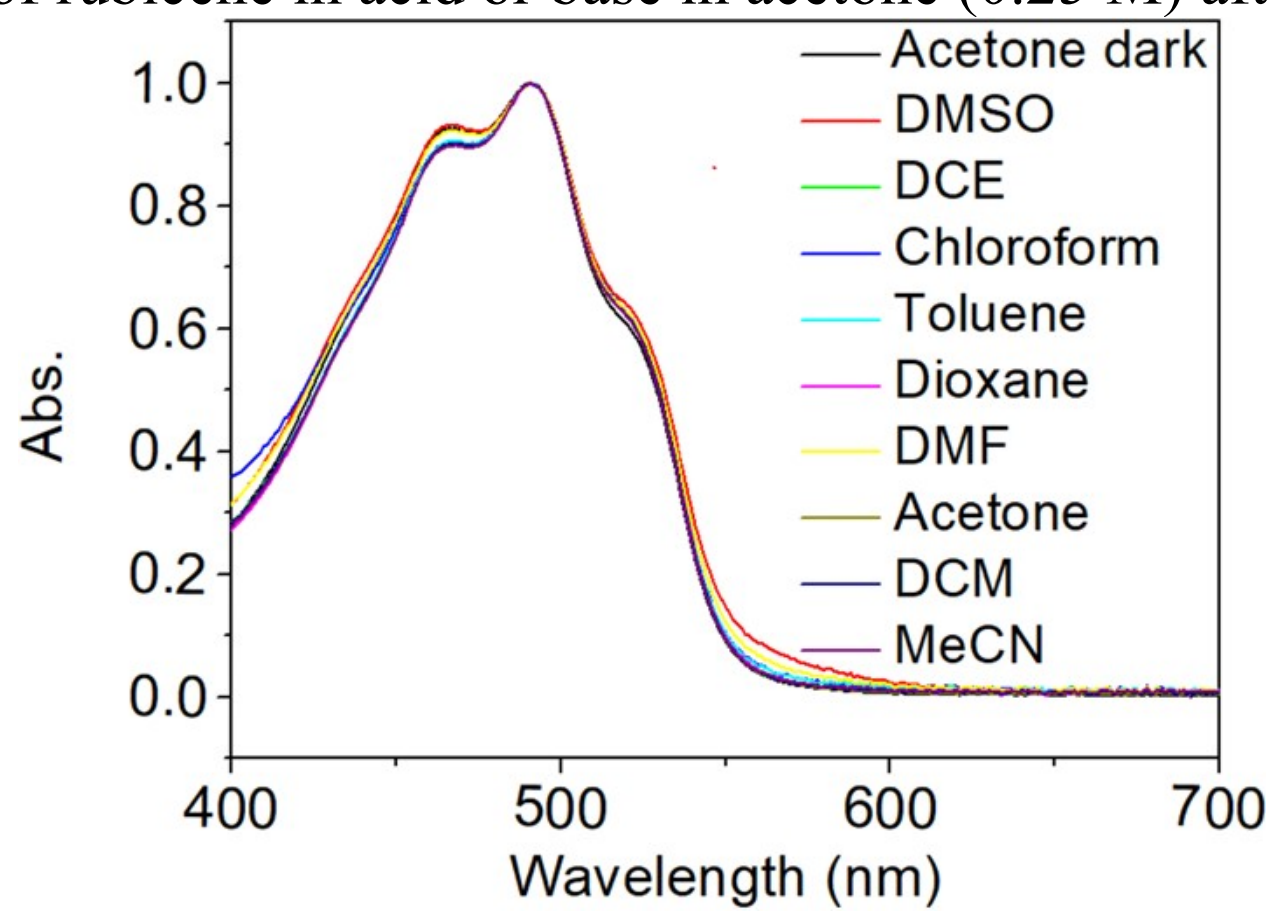

Figure S7. The UV-vis absorption of rubicene after light irradiation for 6h in different solvents (rubicene: 0.5 mM, bubbling N_2 to degas for 5 min, 40 W Blue LED (427 nm) for 6h. Dilute the solutions for 100 times in acetone.).

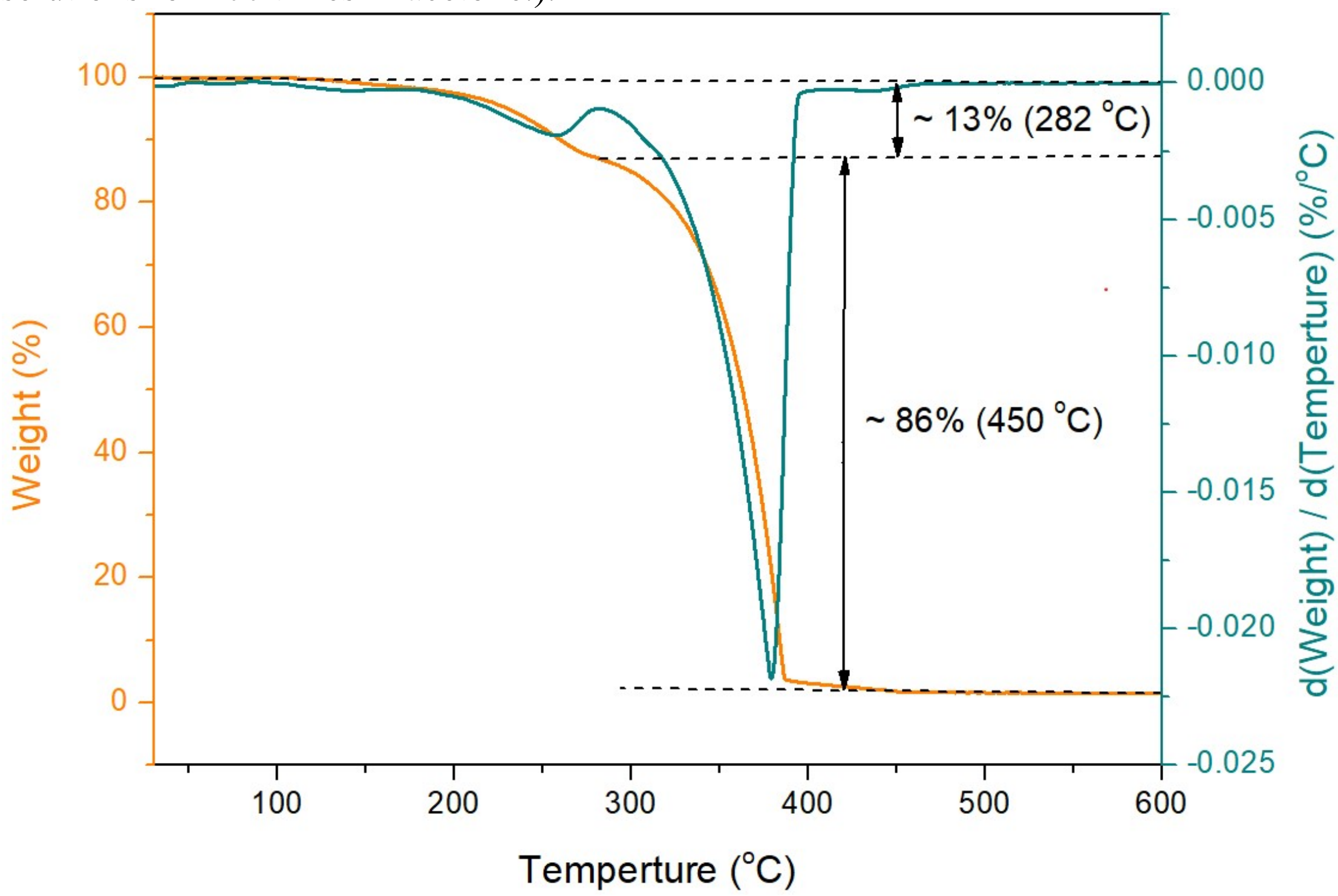

Figure S8. TGA curves of rubicene.

VI. NMR spectra

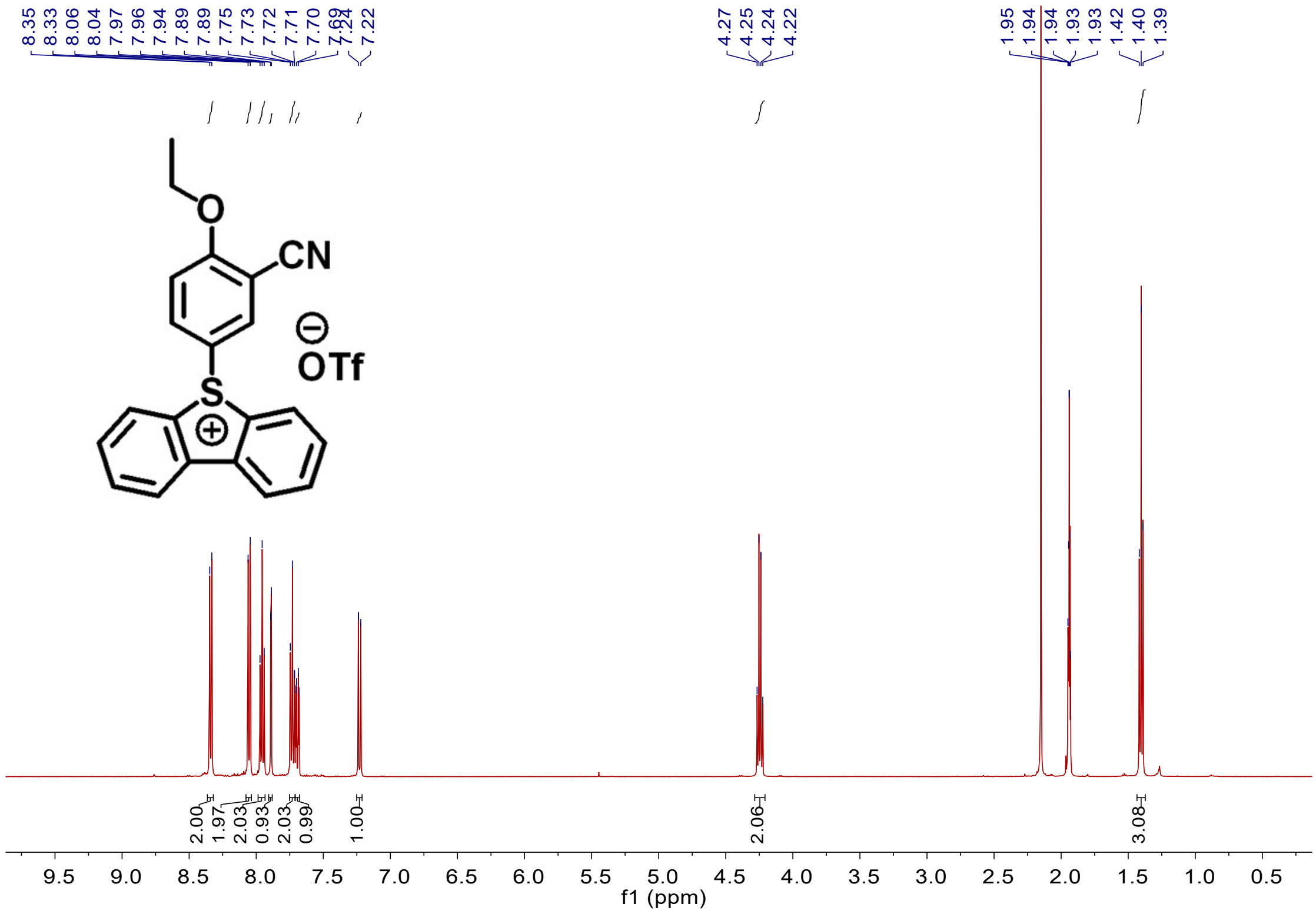

Figure S9. ^{1}H NMR (500 MHz) spectra of 1a in CD_3CN at 298 K.

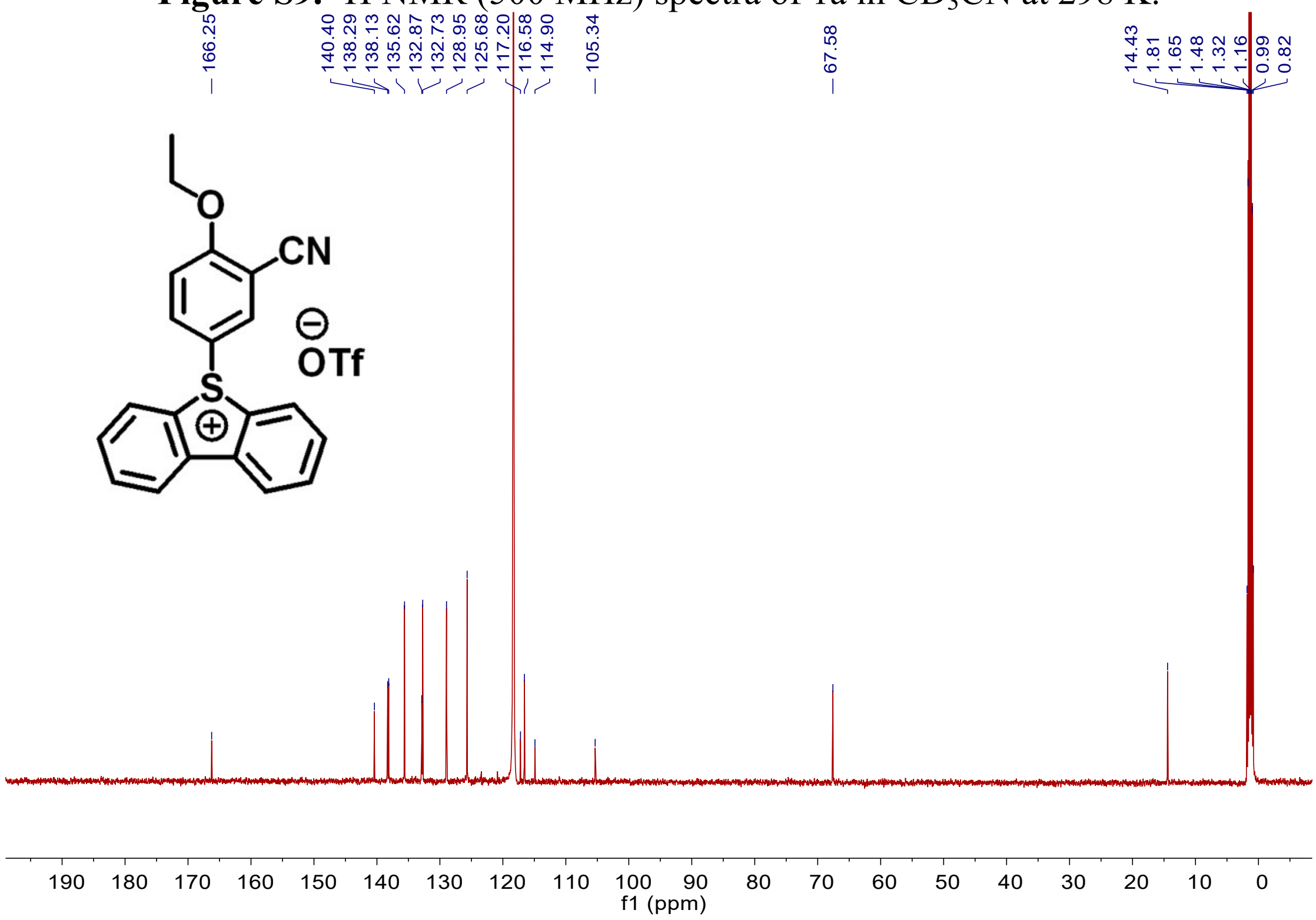

Figure S10. ^{13}C NMR (126 MHz) spectra of 1a in CD_3CN at 298 K.

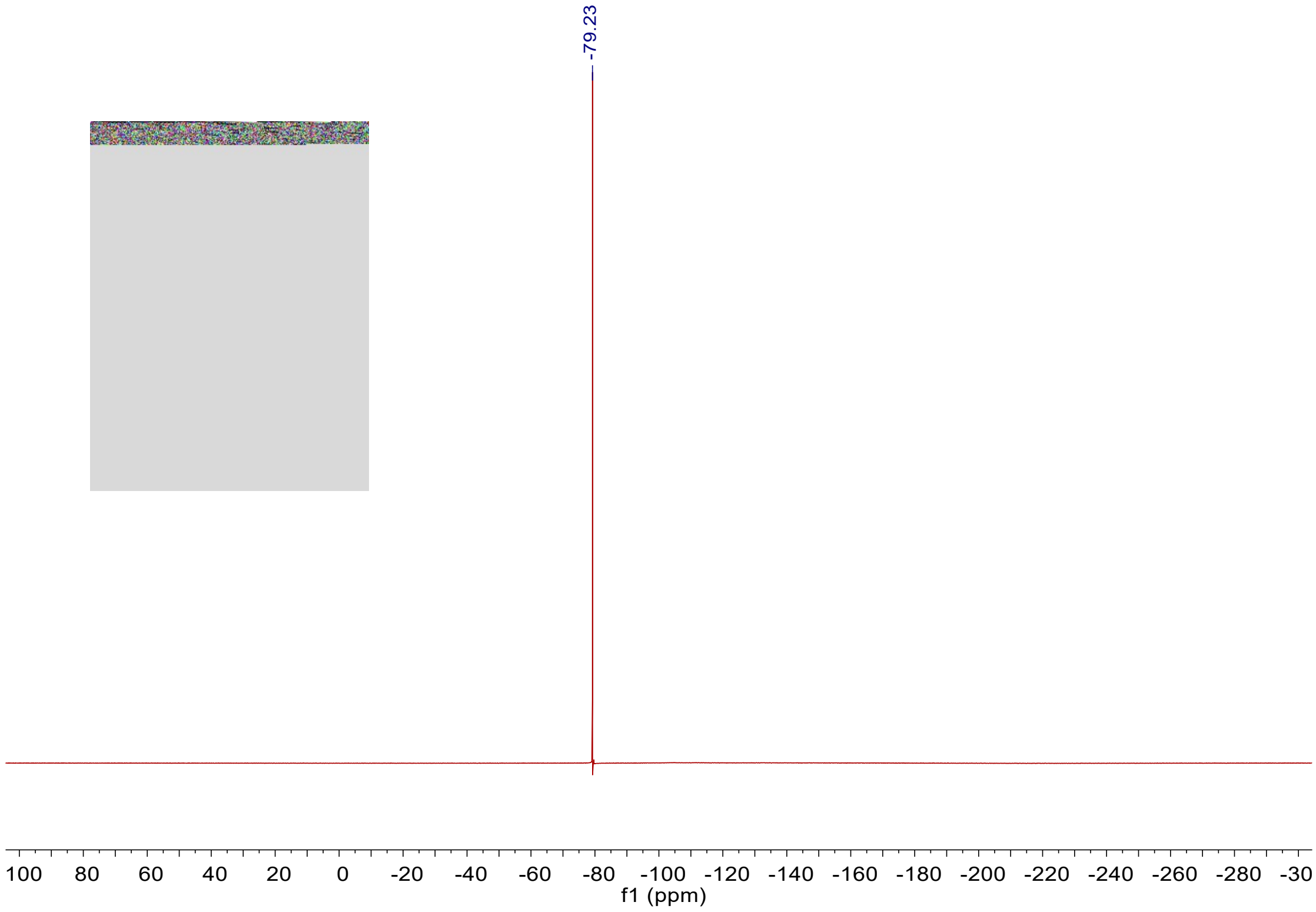

Figure S11. ^{19}F NMR (471 MHz) spectra of 1a in CD_3CN at 298 K.

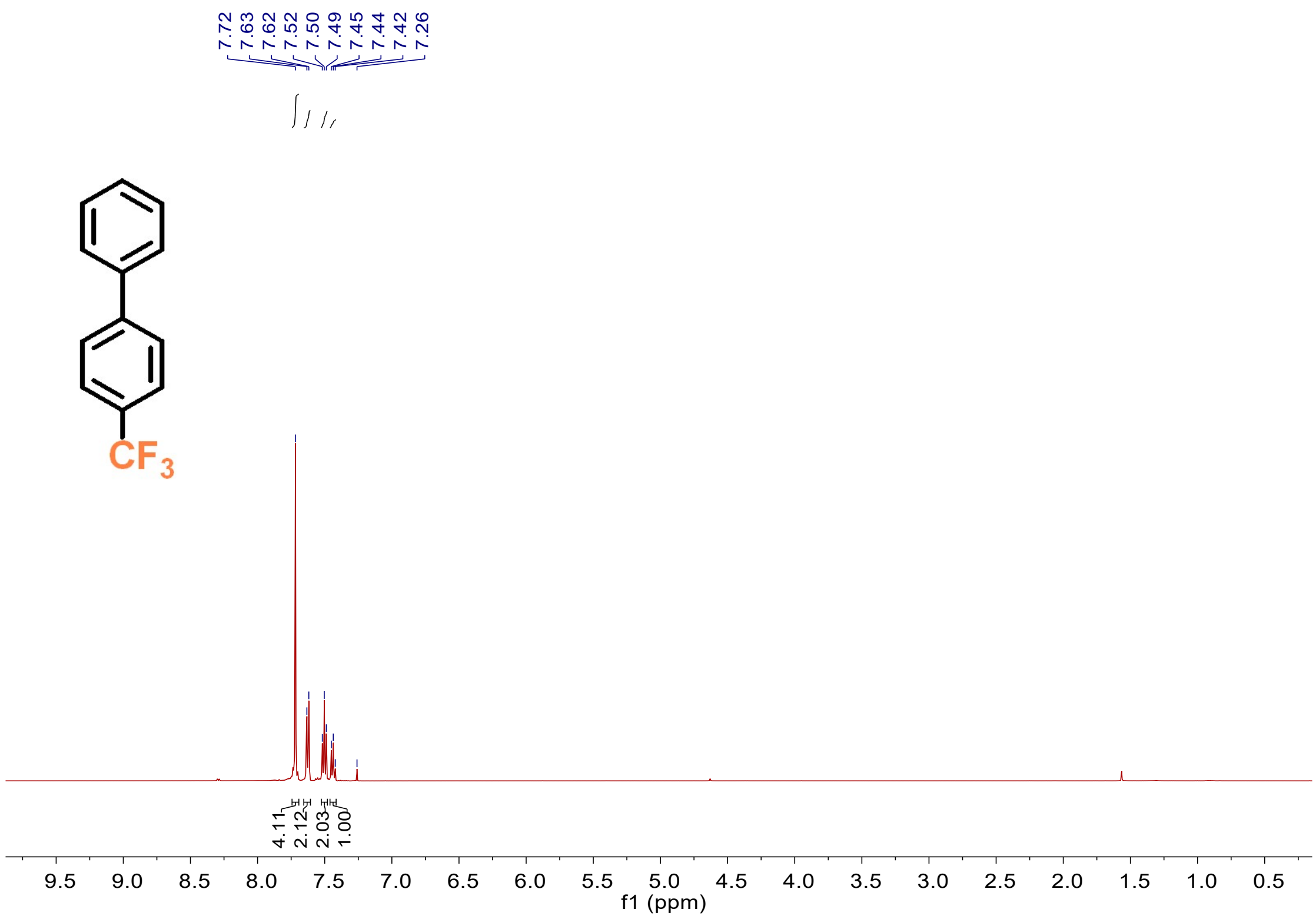

Figure S12. ^{1}H NMR (500 MHz) spectra of A1 in $CDCl_3$ at 298 K.

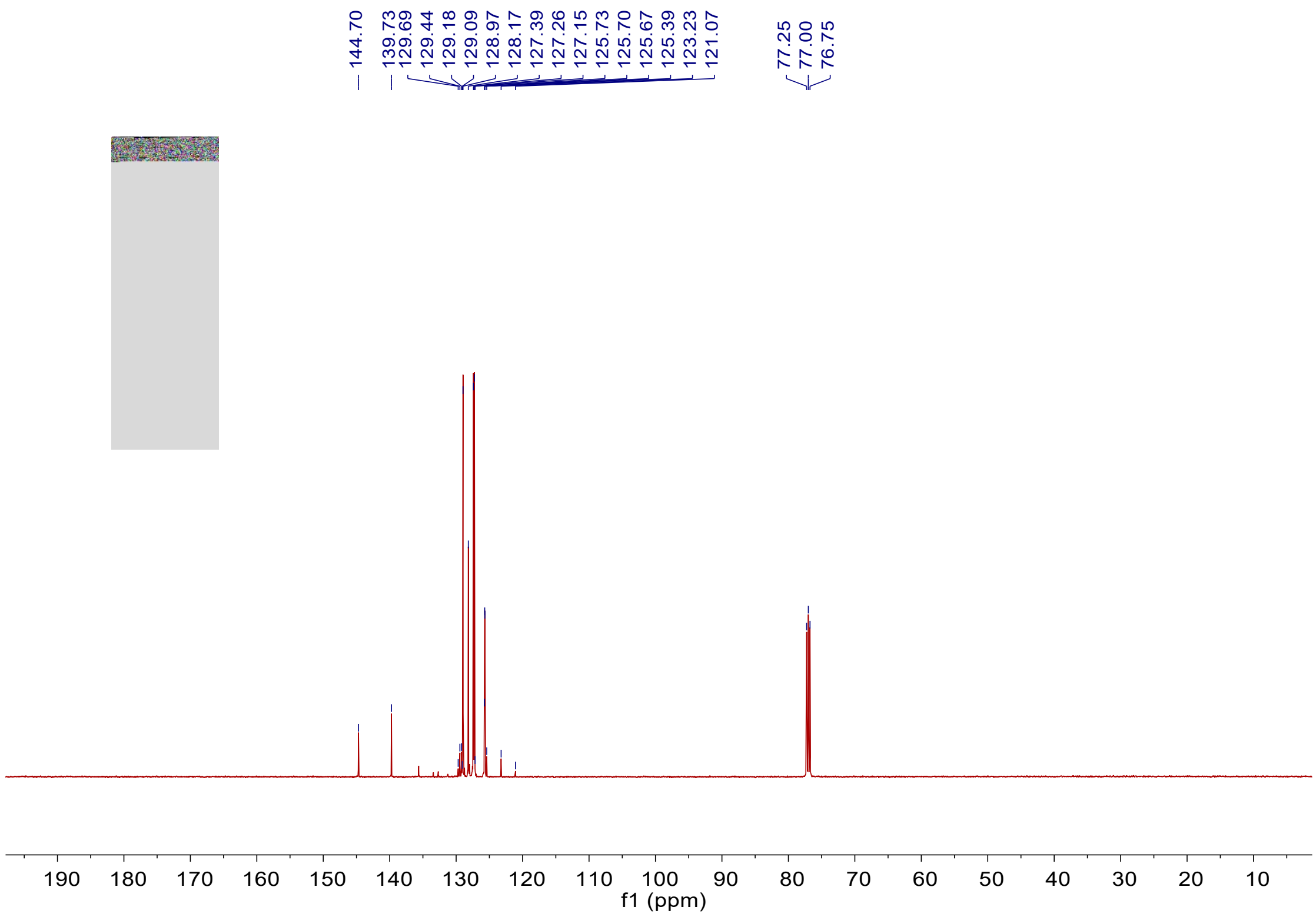

Figure S13. ^{13}C NMR (126 MHz) spectra of A1 in $CDCl_3$ at 298 K.

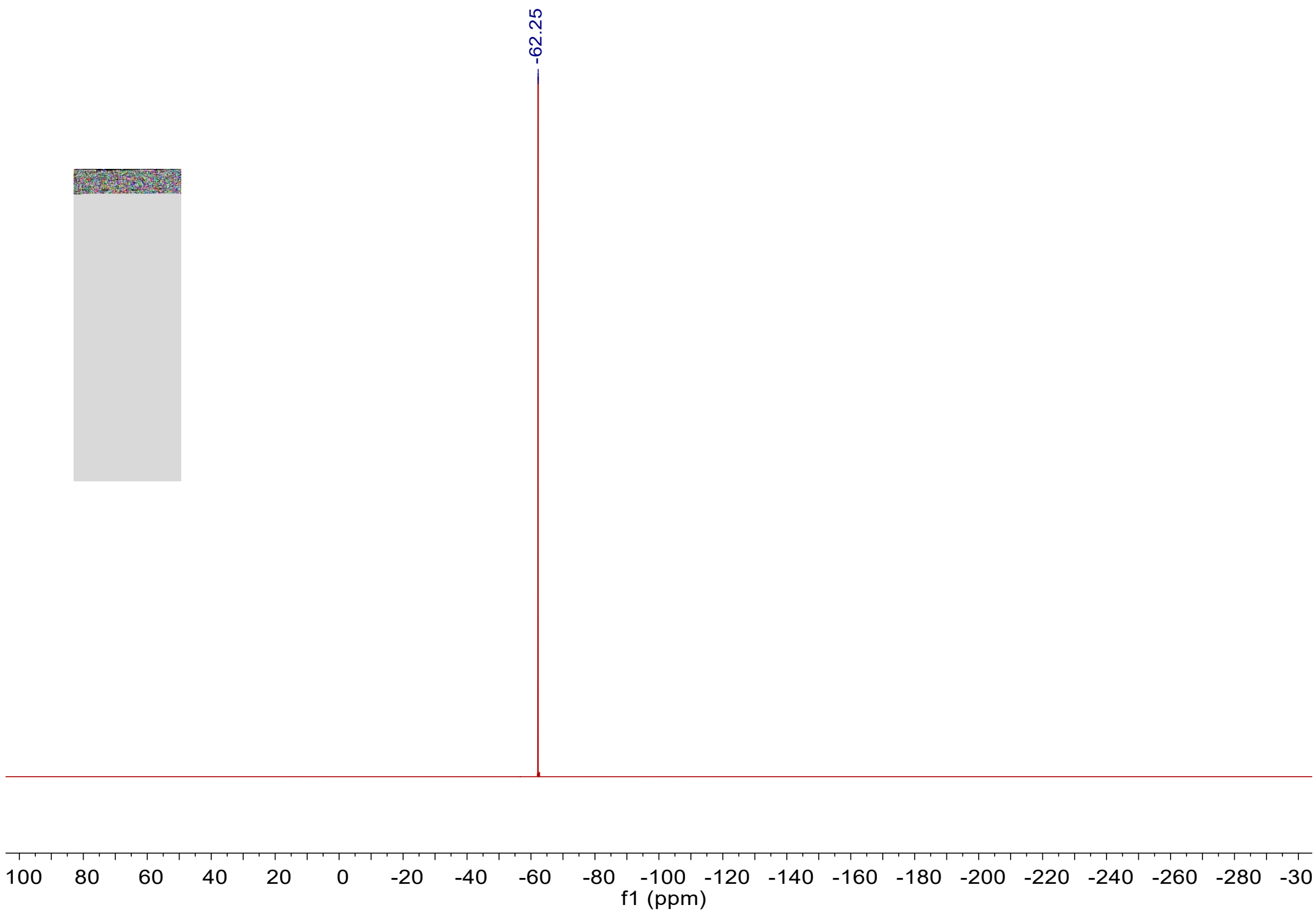

Figure S14. ^{19}F NMR (471 MHz) spectra of A1 in $CDCl_3$ at 298 K.

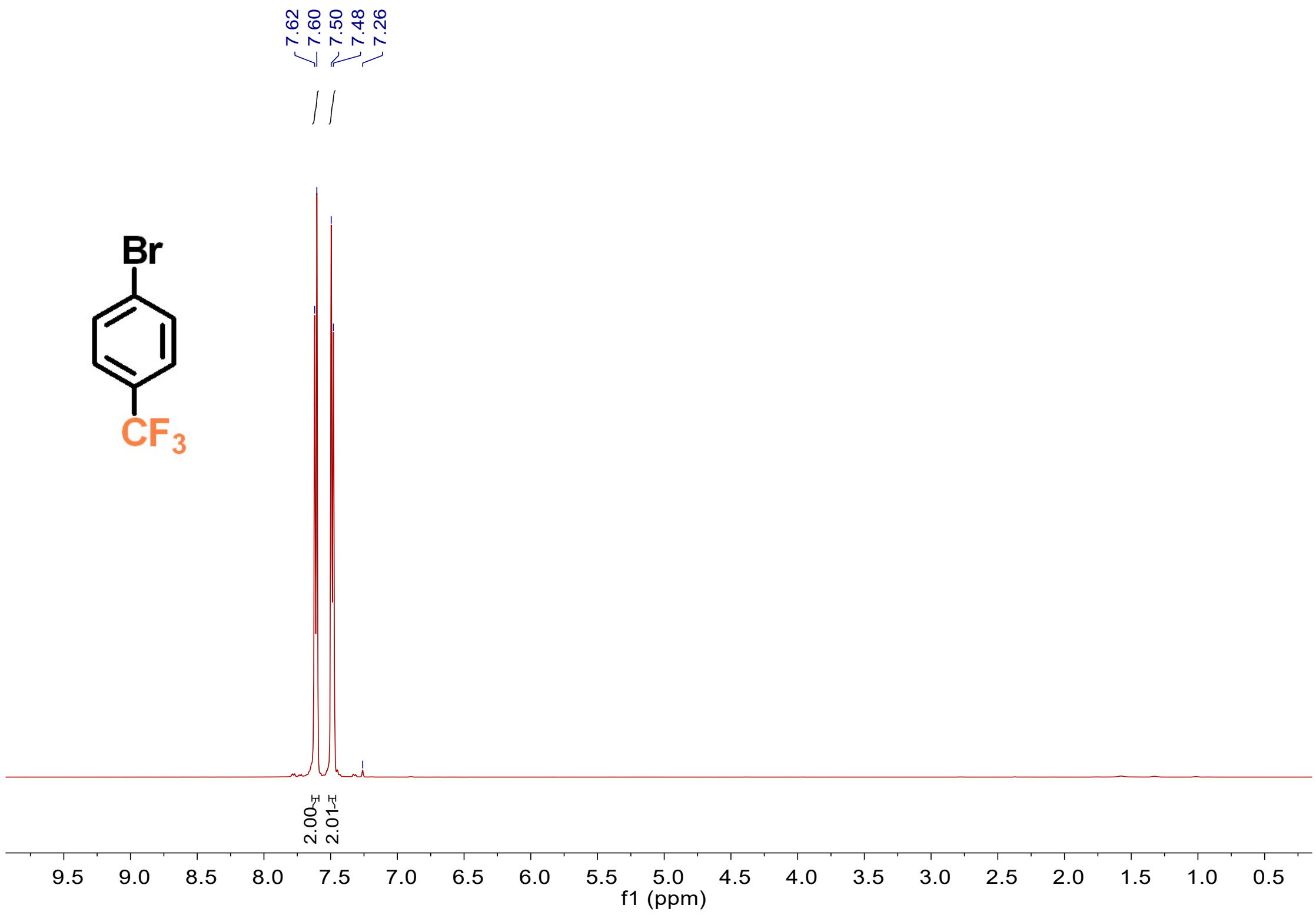

Figure S15. ^{1}H NMR (500 MHz) spectra of A2 in $CDCl_3$ at 298 K.

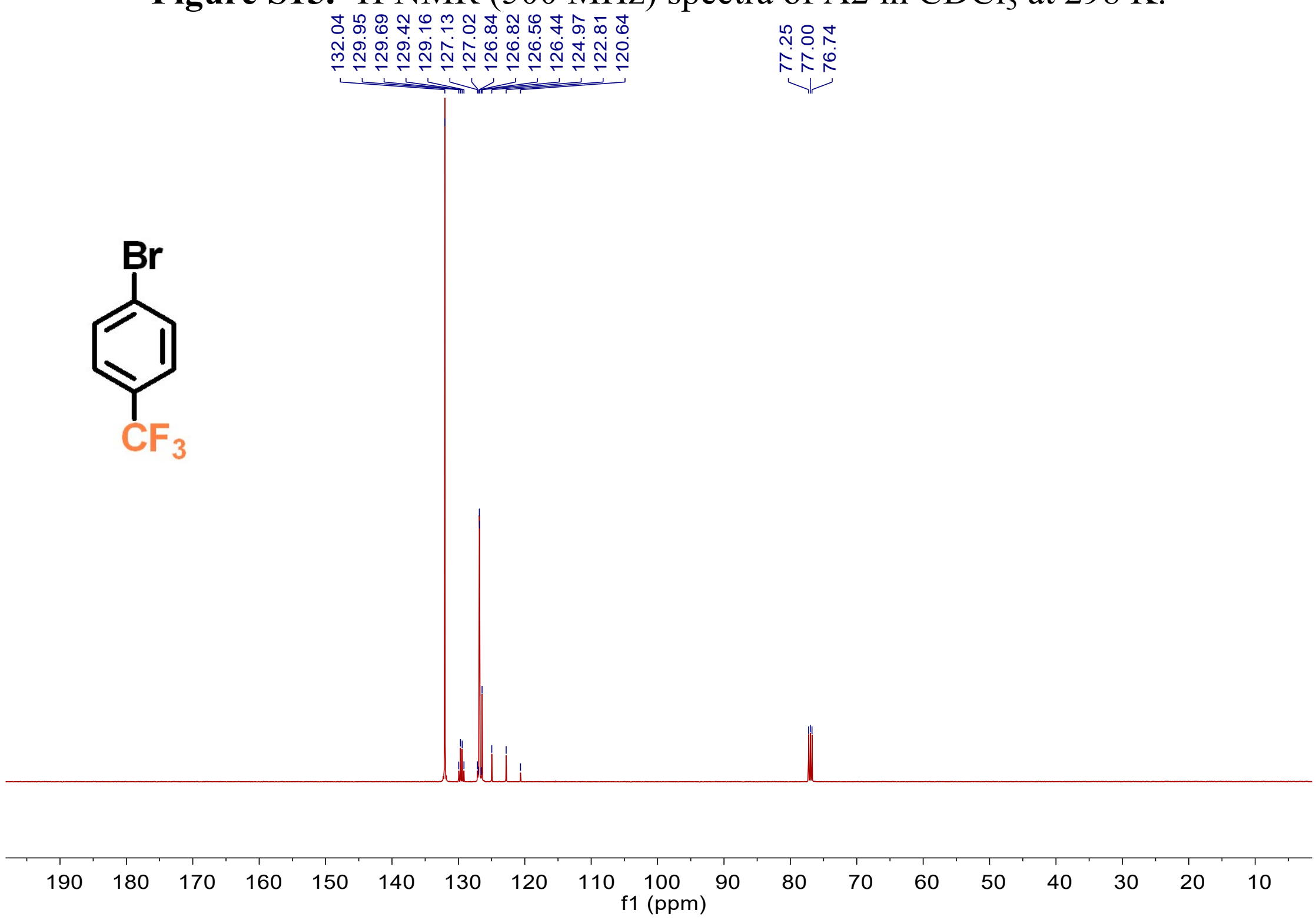

Figure S16. ^{13}C NMR (126 MHz) spectra of A2 in $CDCl_3$ at 298 K.

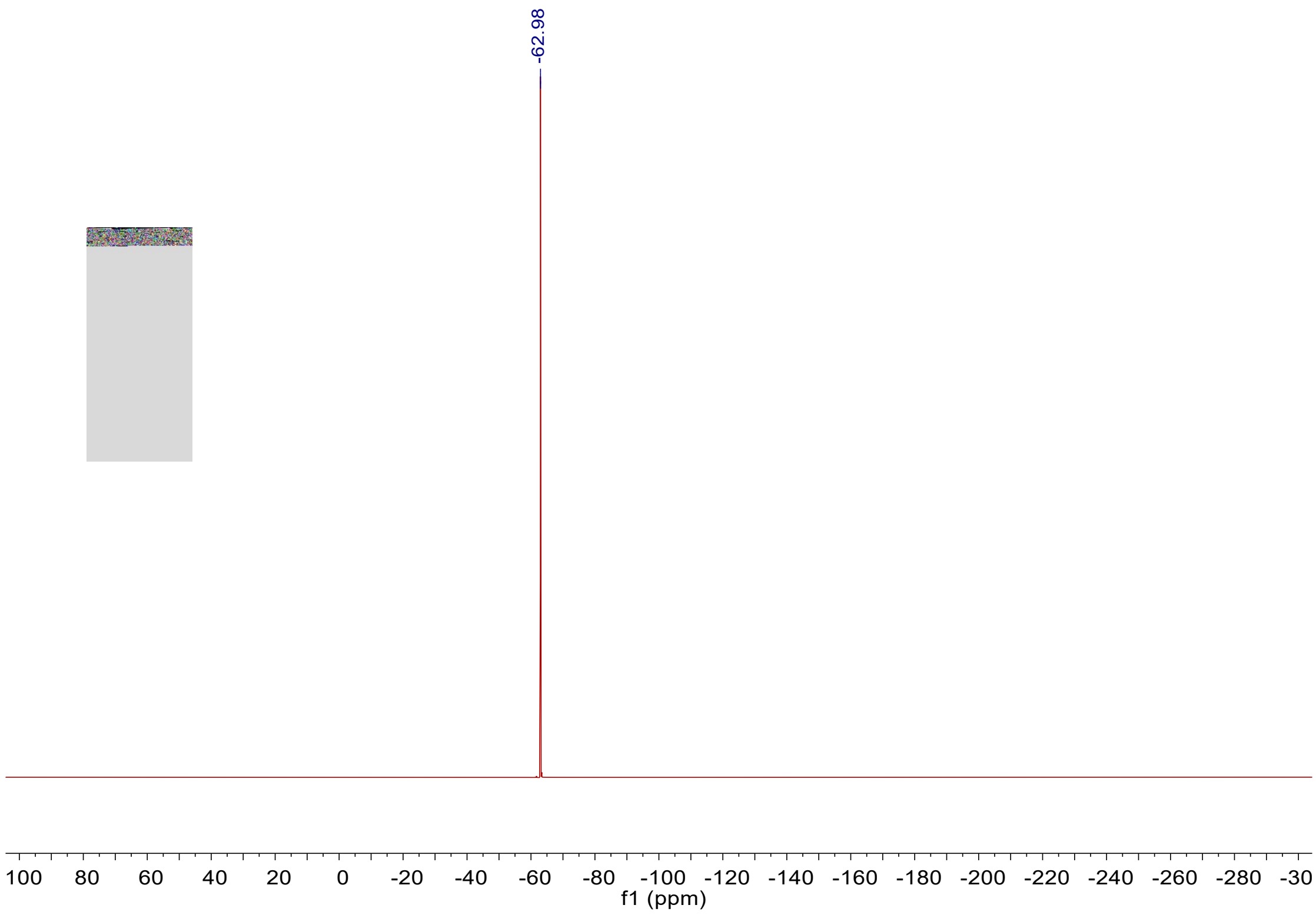

Figure S17. ^{19}F NMR (471 MHz) spectra of A2 in $CDCl_3$ at 298 K.

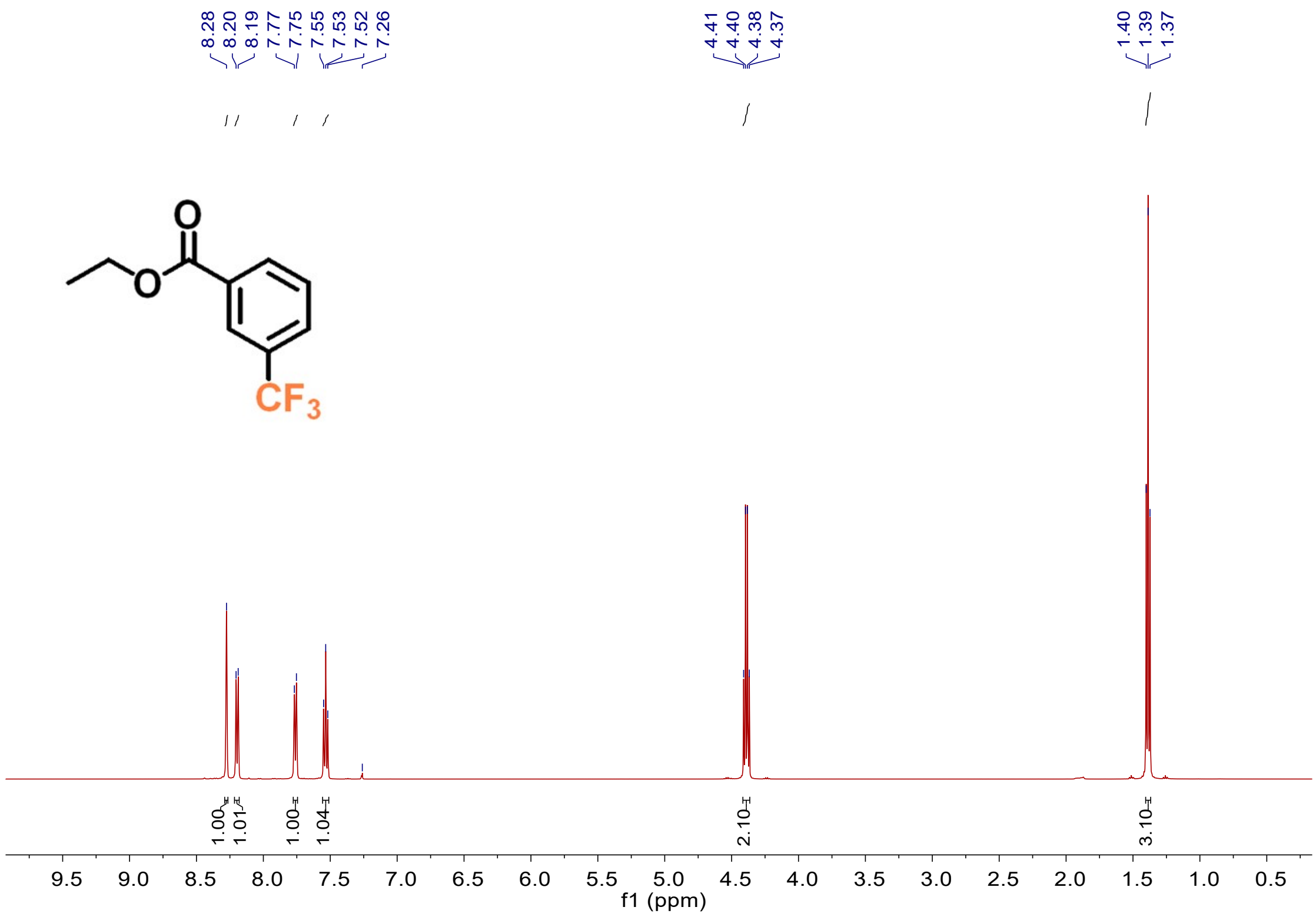

Figure S18. ^{1}H NMR (500 MHz) spectra of A3 in $CDCl_3$ at 298 K.

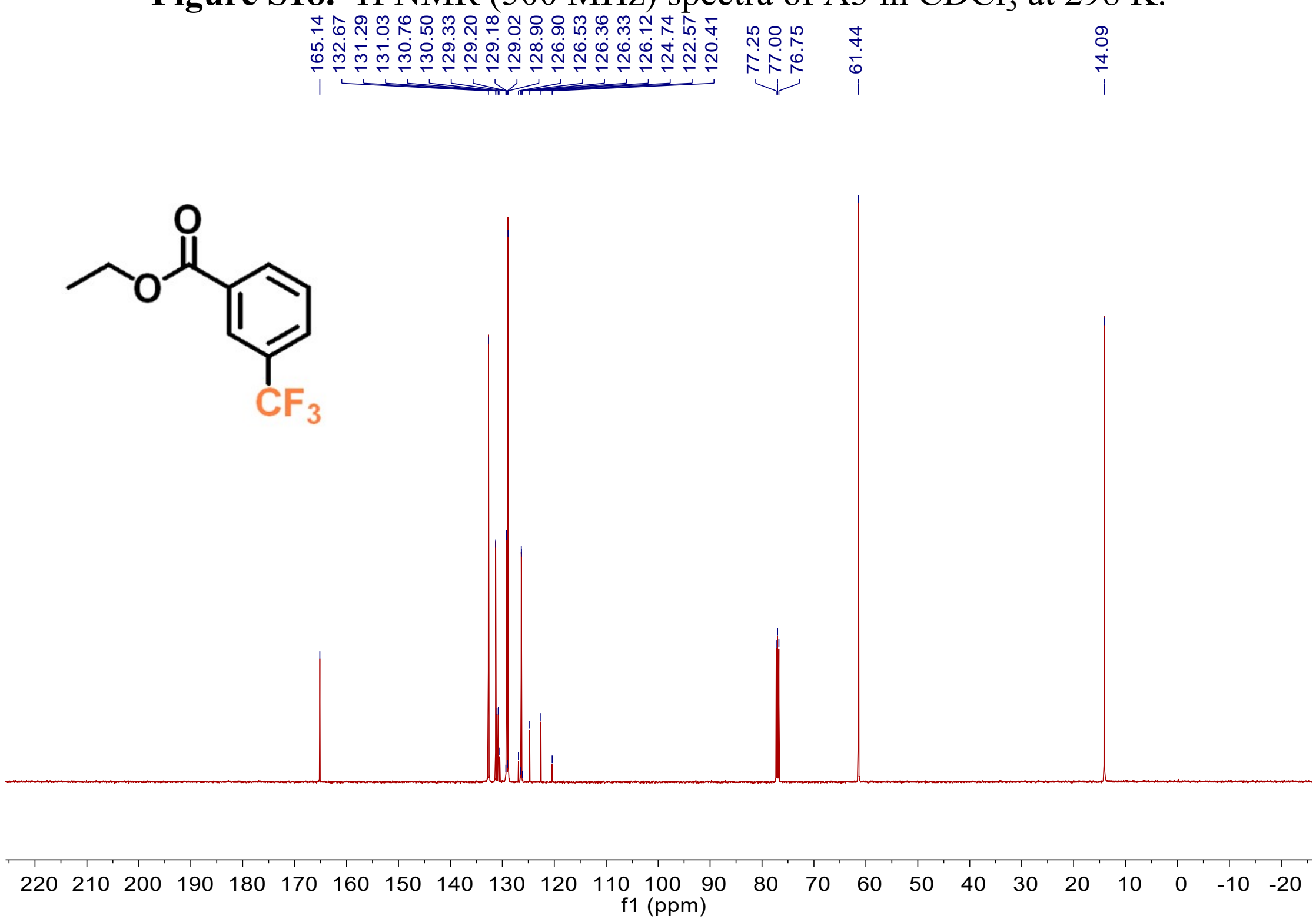

Figure S19. ^{13}C NMR (126 MHz) spectra of A3 in $CDCl_3$ at 298 K.

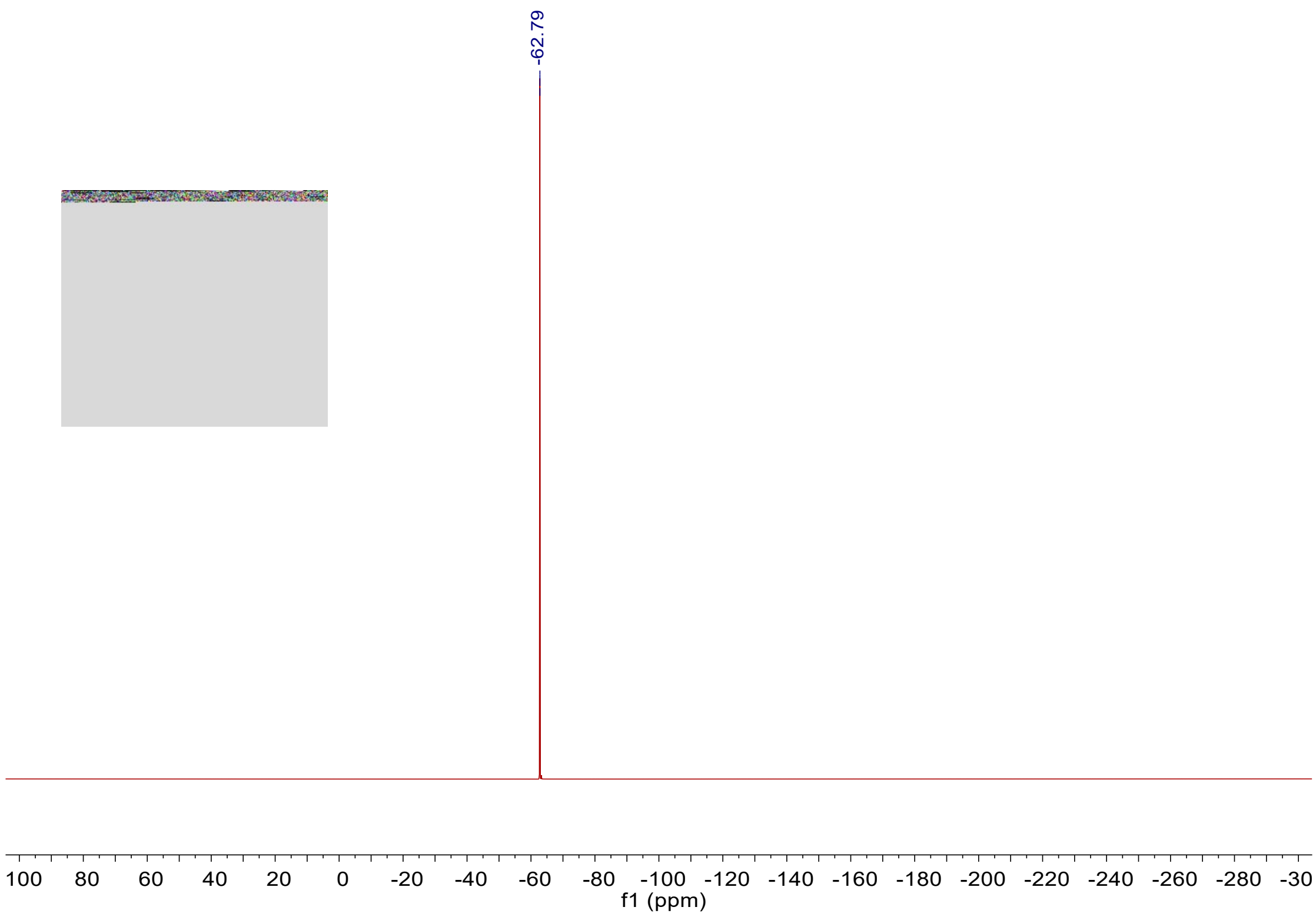

Figure S20. ^{19}F NMR (471 MHz) spectra of A3 in $CDCl_3$ at 298 K.

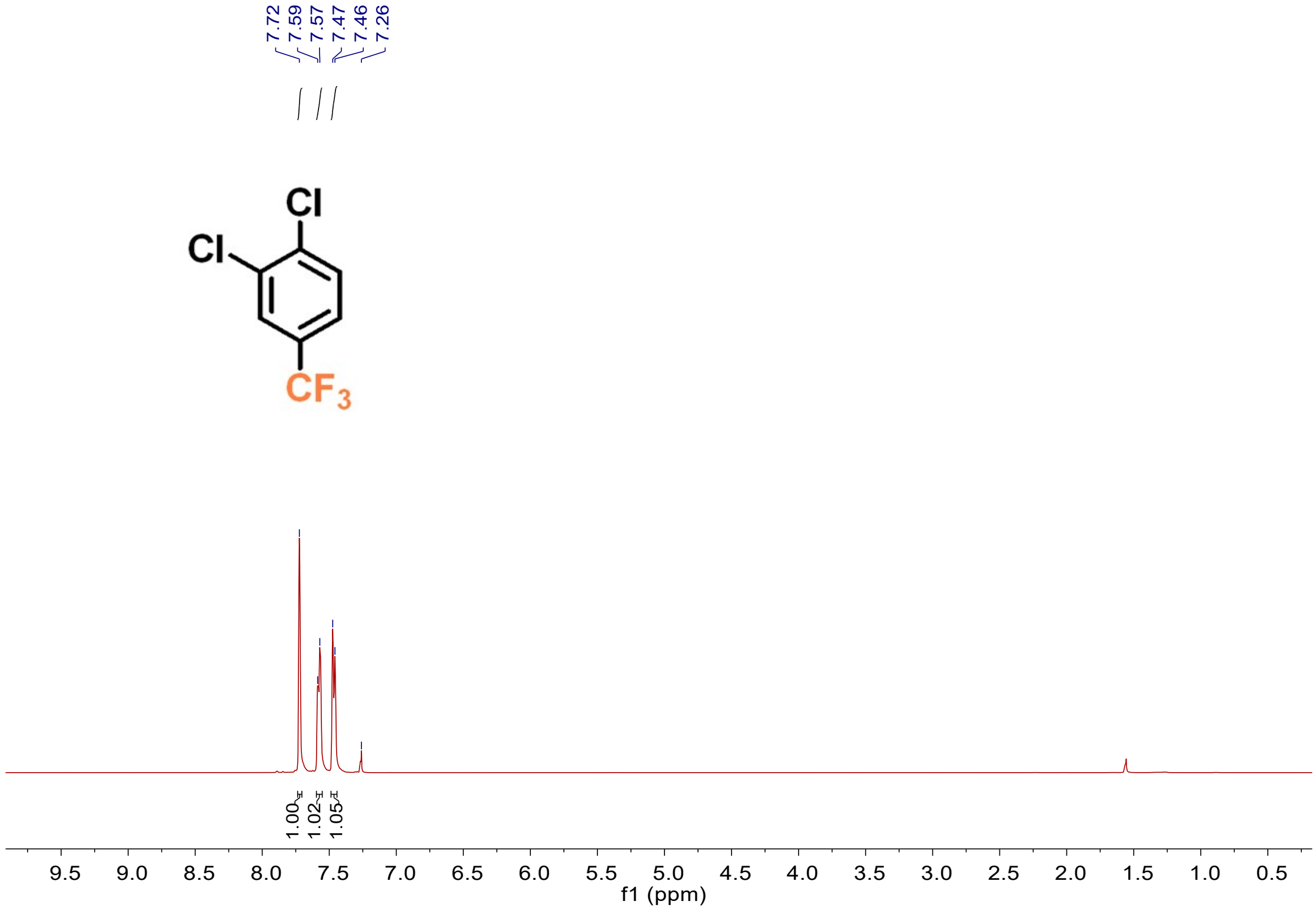

Figure S21. ^{1}H NMR (500 MHz) spectra of A4 in $CDCl_3$ at 298 K.

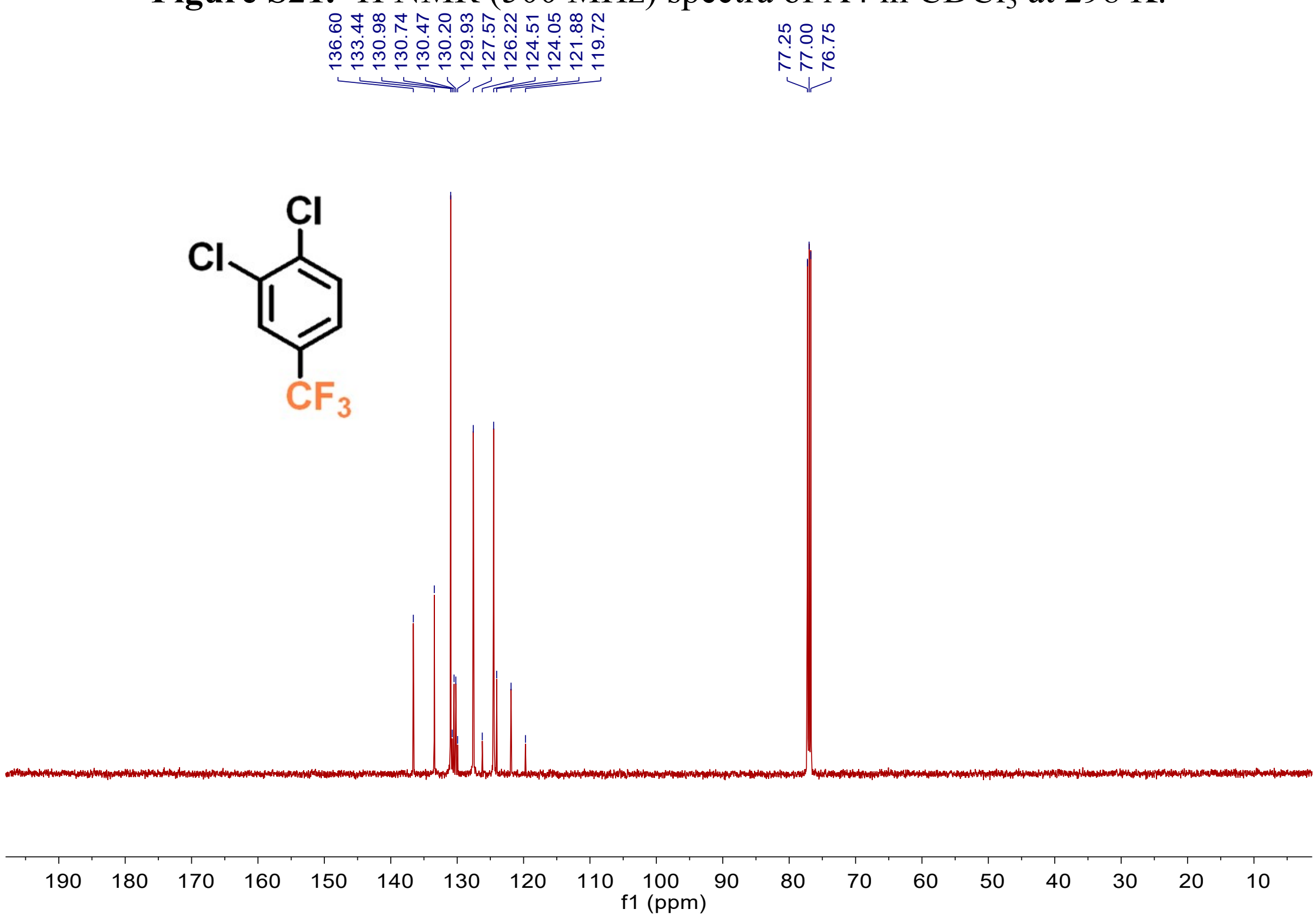

Figure S22. ^{13}C NMR (126 MHz) spectra of A4 in $CDCl_3$ at 298 K.

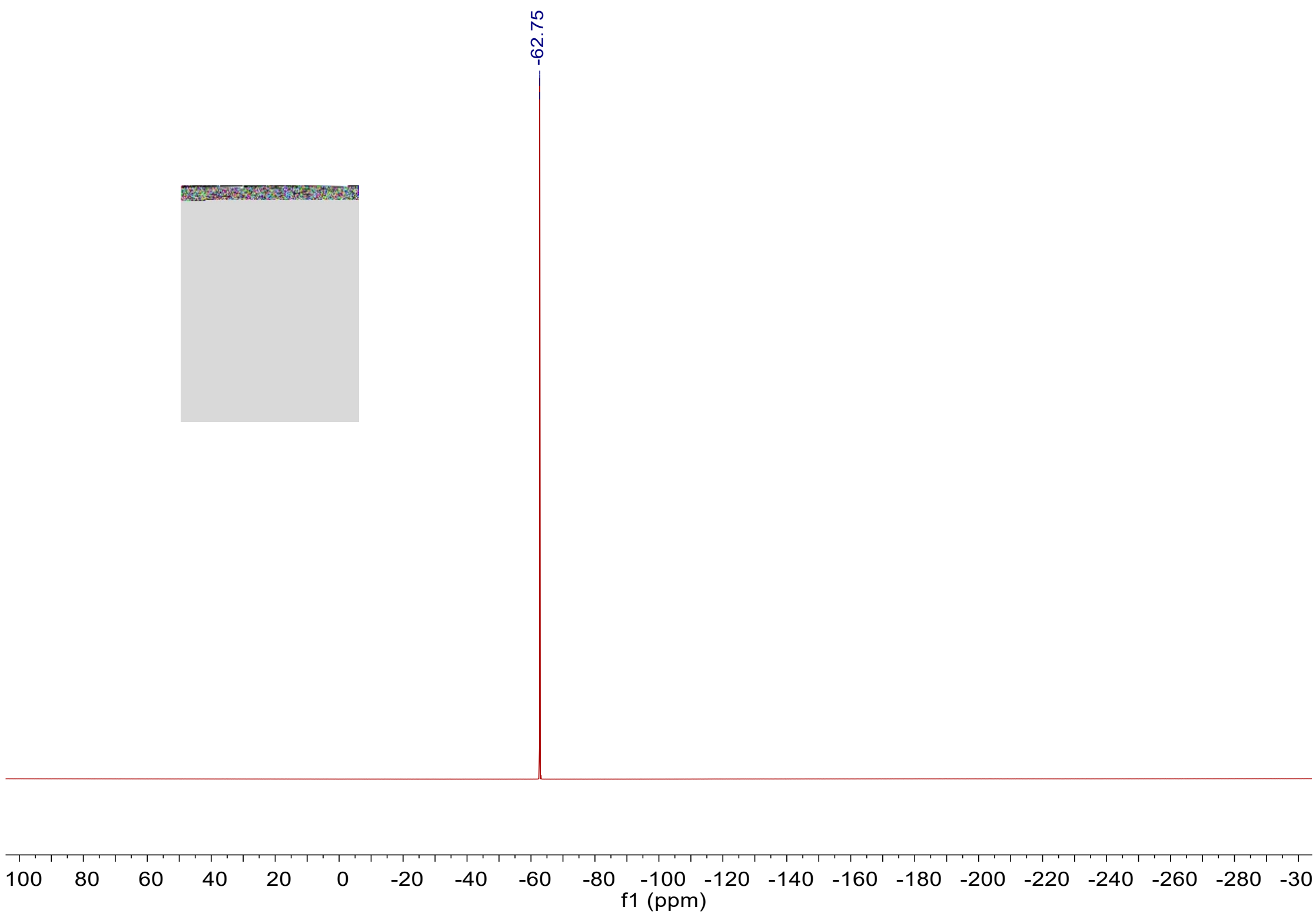

Figure S23. ^{19}F NMR (471 MHz) spectra of A4 in $CDCl_3$ at 298 K.

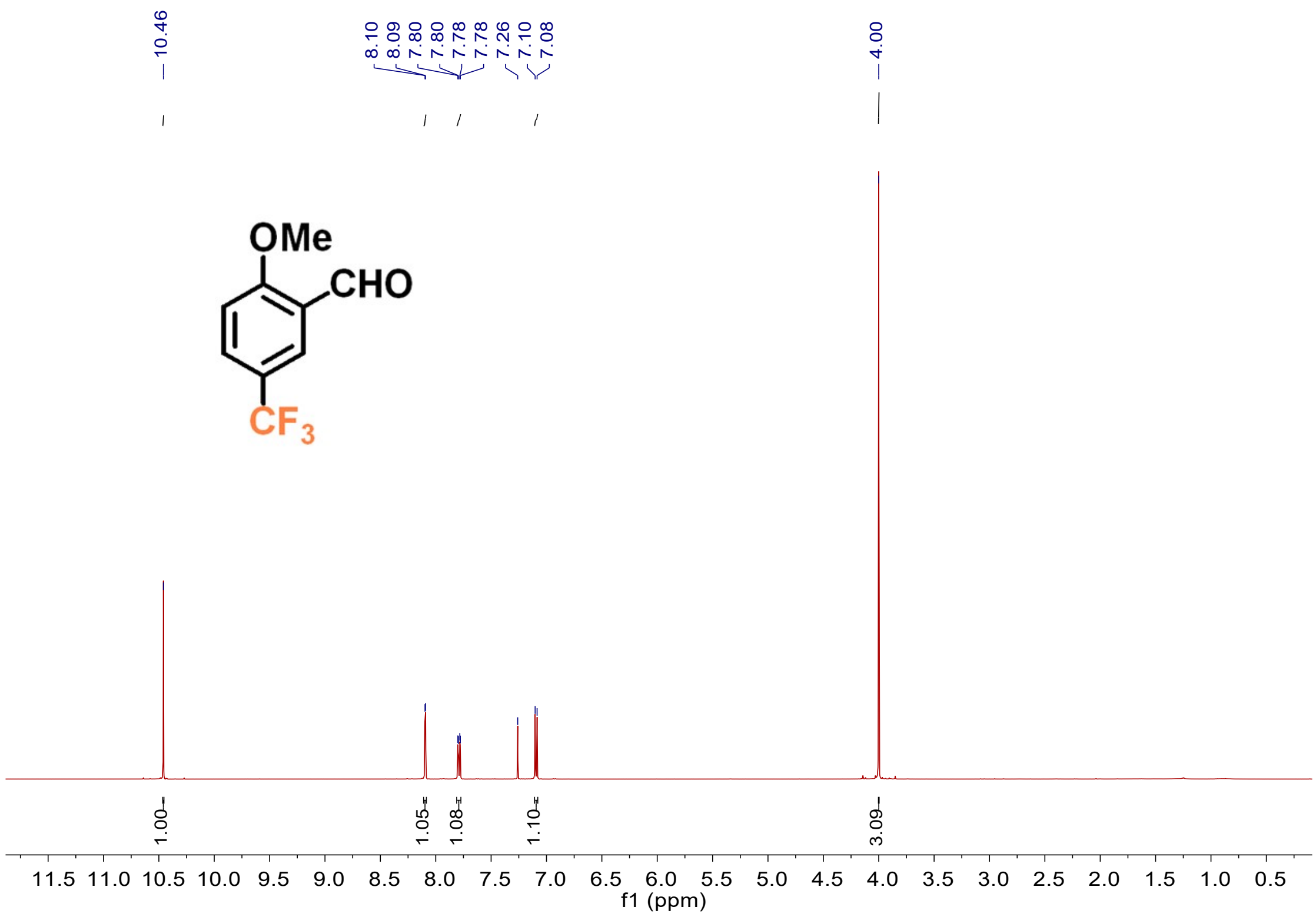

Figure S24. ^{1}H NMR (500 MHz) spectra of A5 in $CDCl_3$ at 298 K.

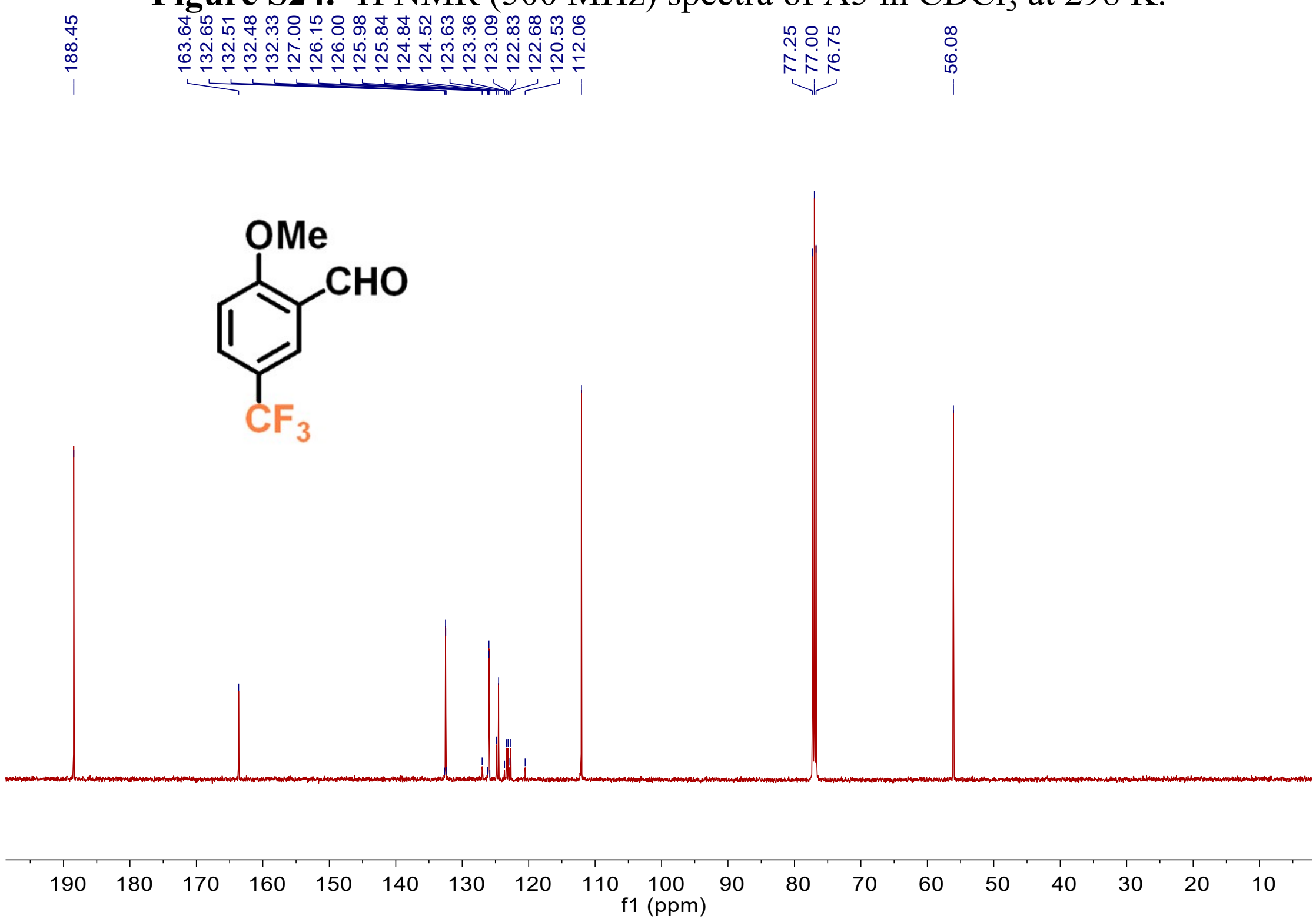

Figure S25. ^{13}C NMR (126 MHz) spectra of A5 in $CDCl_3$ at 298 K.

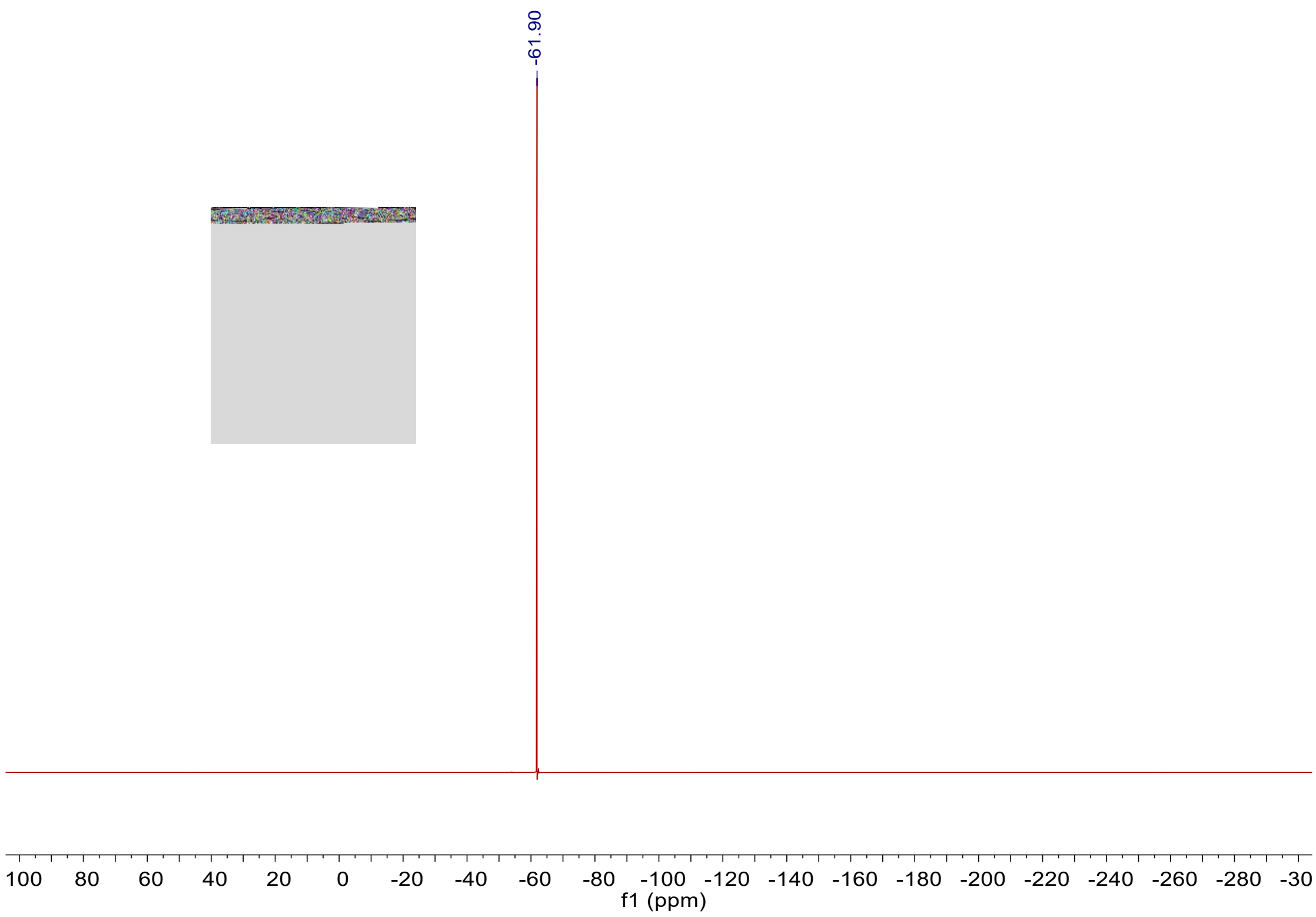

Figure S26. ^{19}F NMR (471 MHz) spectra of A5 in $CDCl_3$ at 298 K.

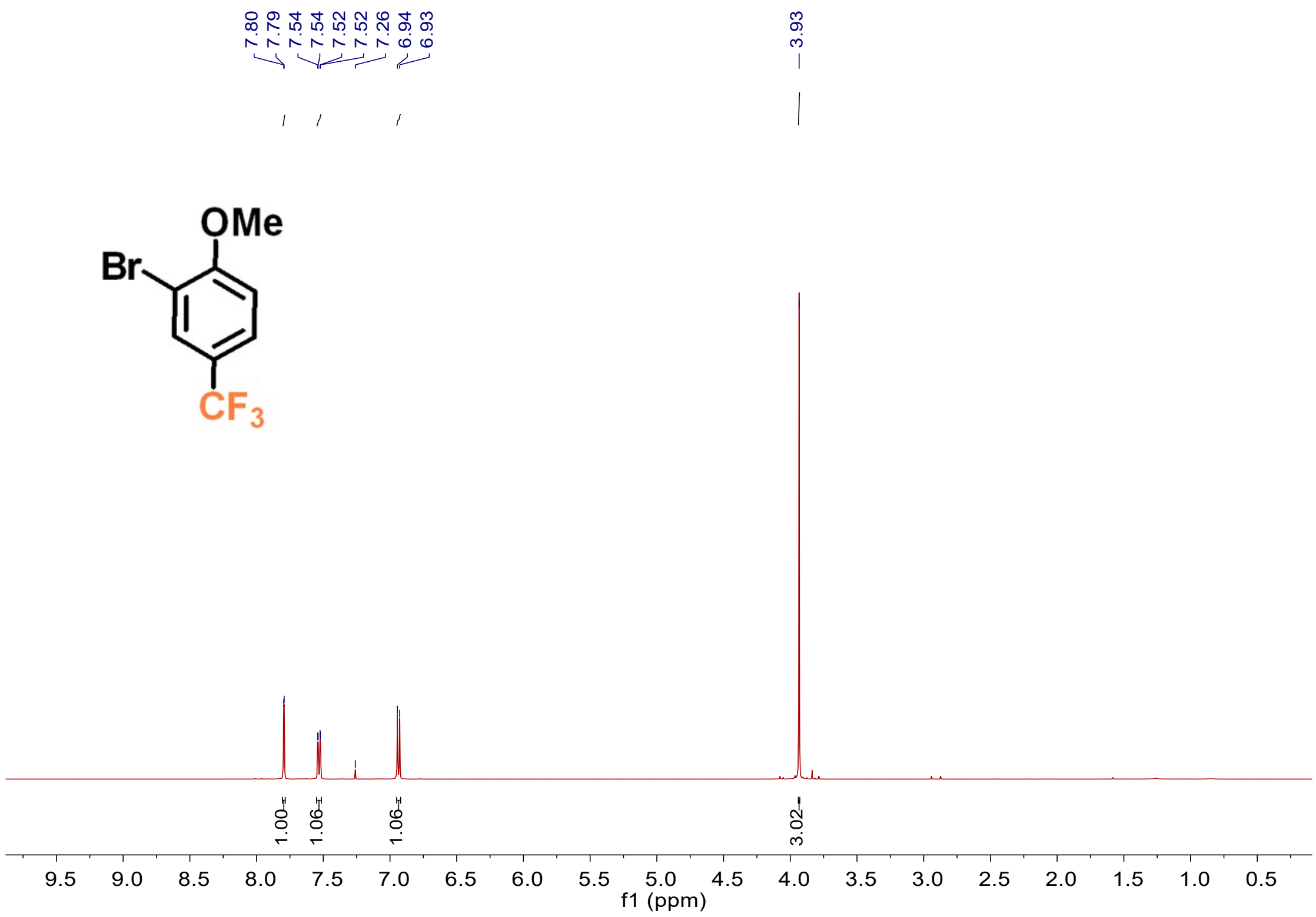

Figure S27. ^{1}H NMR (500 MHz) spectra of A6 in $CDCl_3$ at 298 K.

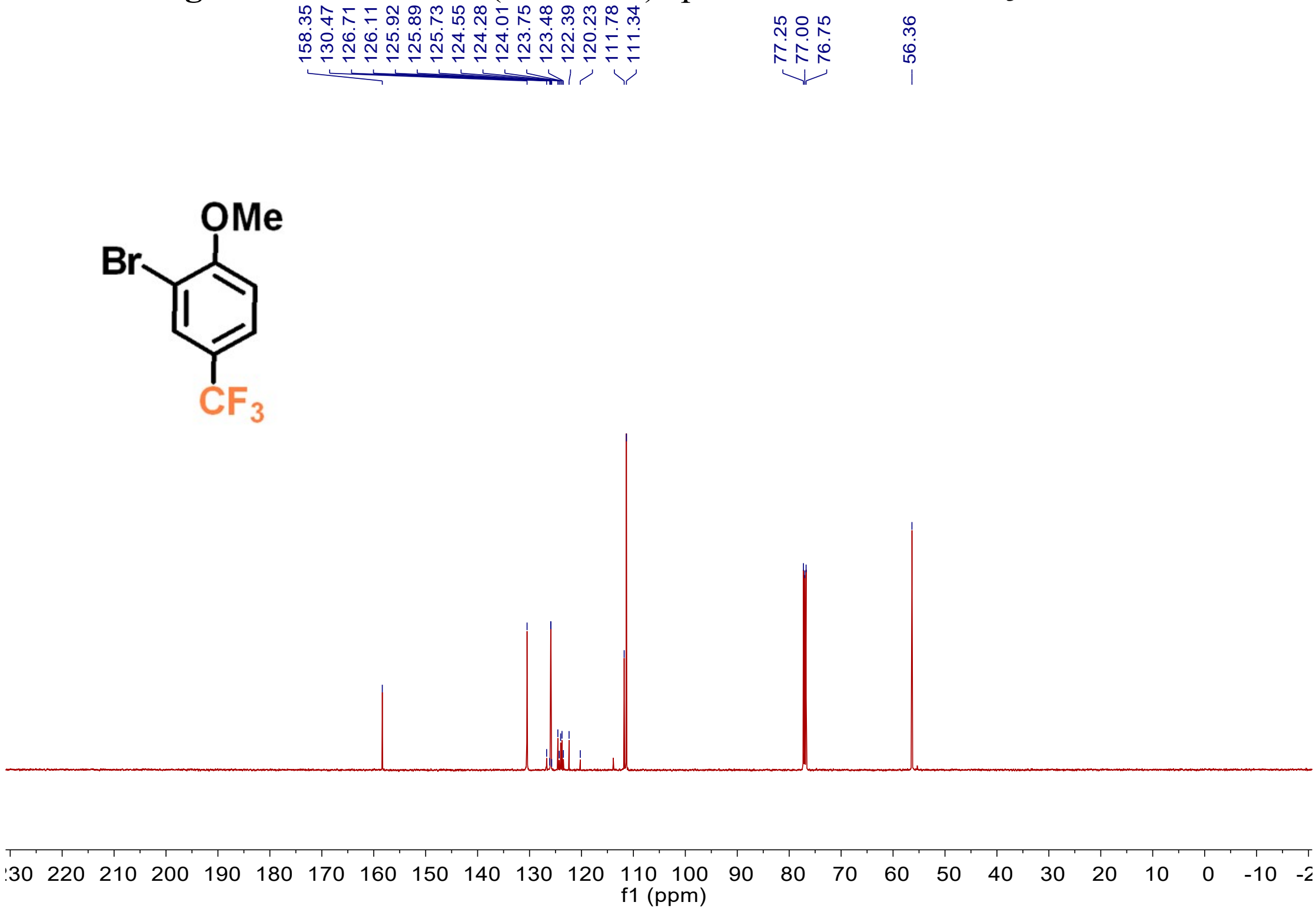

Figure S28. ^{13}C NMR (126 MHz) spectra of A6 in $CDCl_3$ at 298 K.

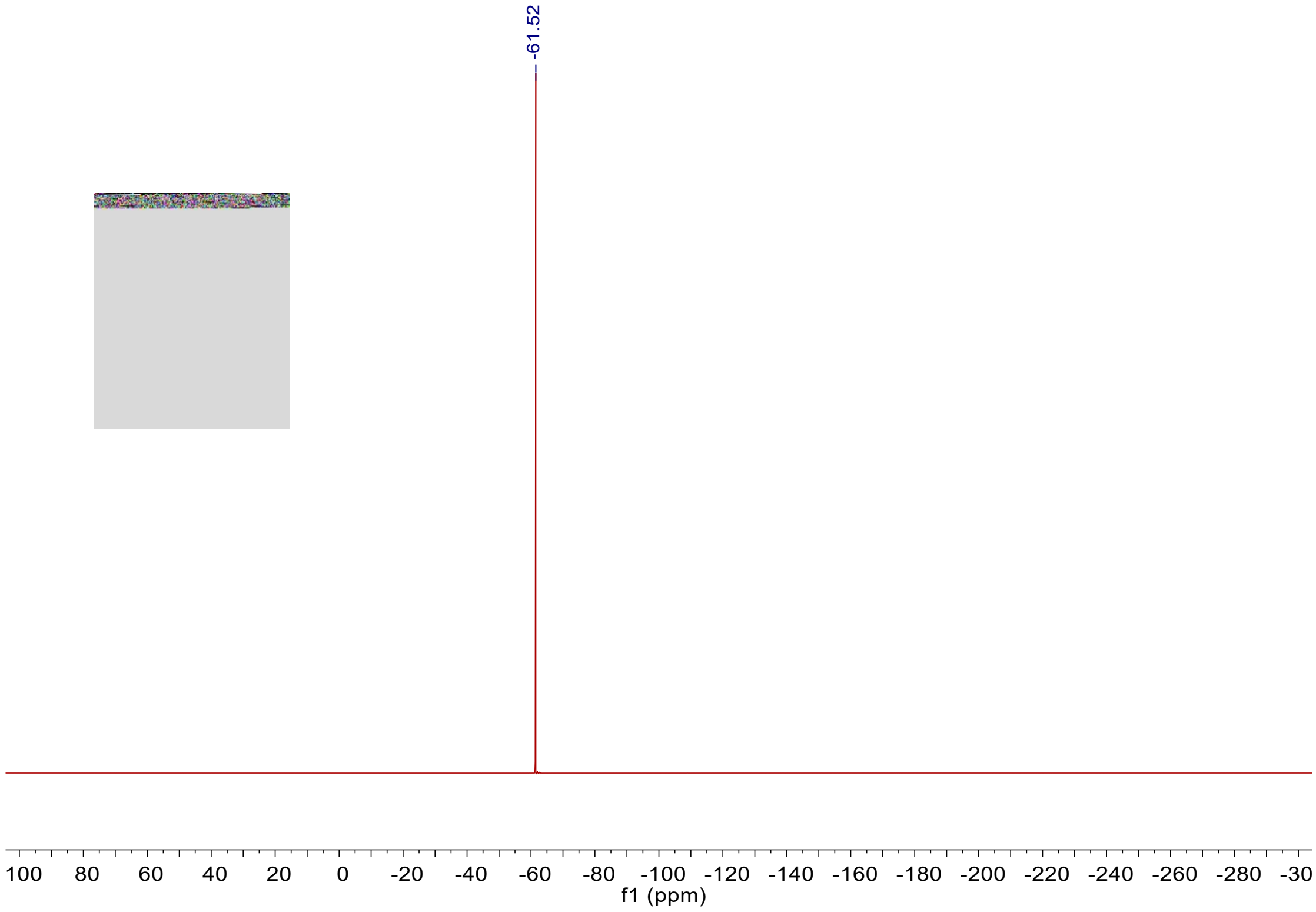

Figure S29. ^{19}F NMR (471 MHz) spectra of A6 in $CDCl_3$ at 298 K.

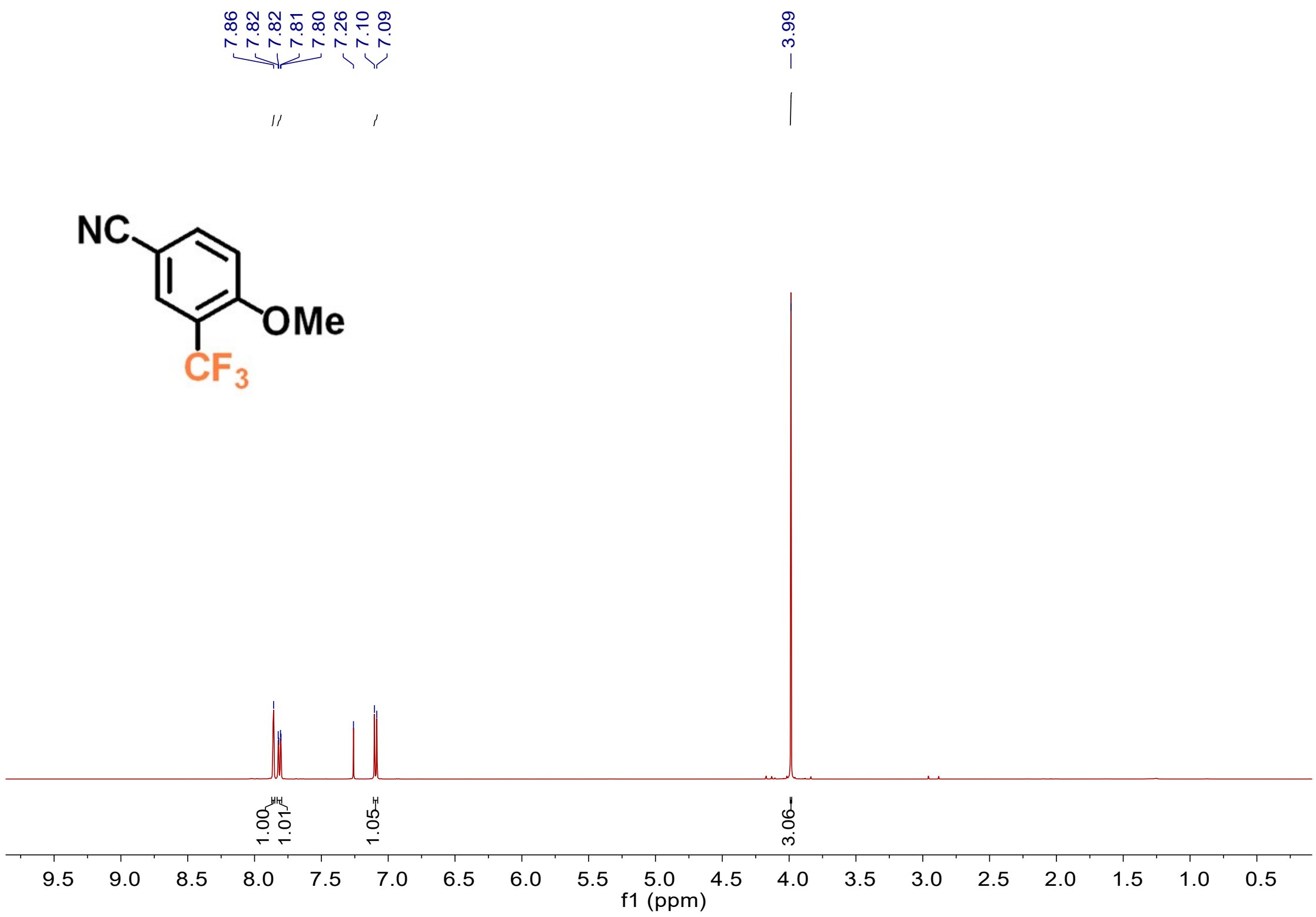

Figure S30. ^{1}H NMR (500 MHz) spectra of A7 in $CDCl_3$ at 298 K.

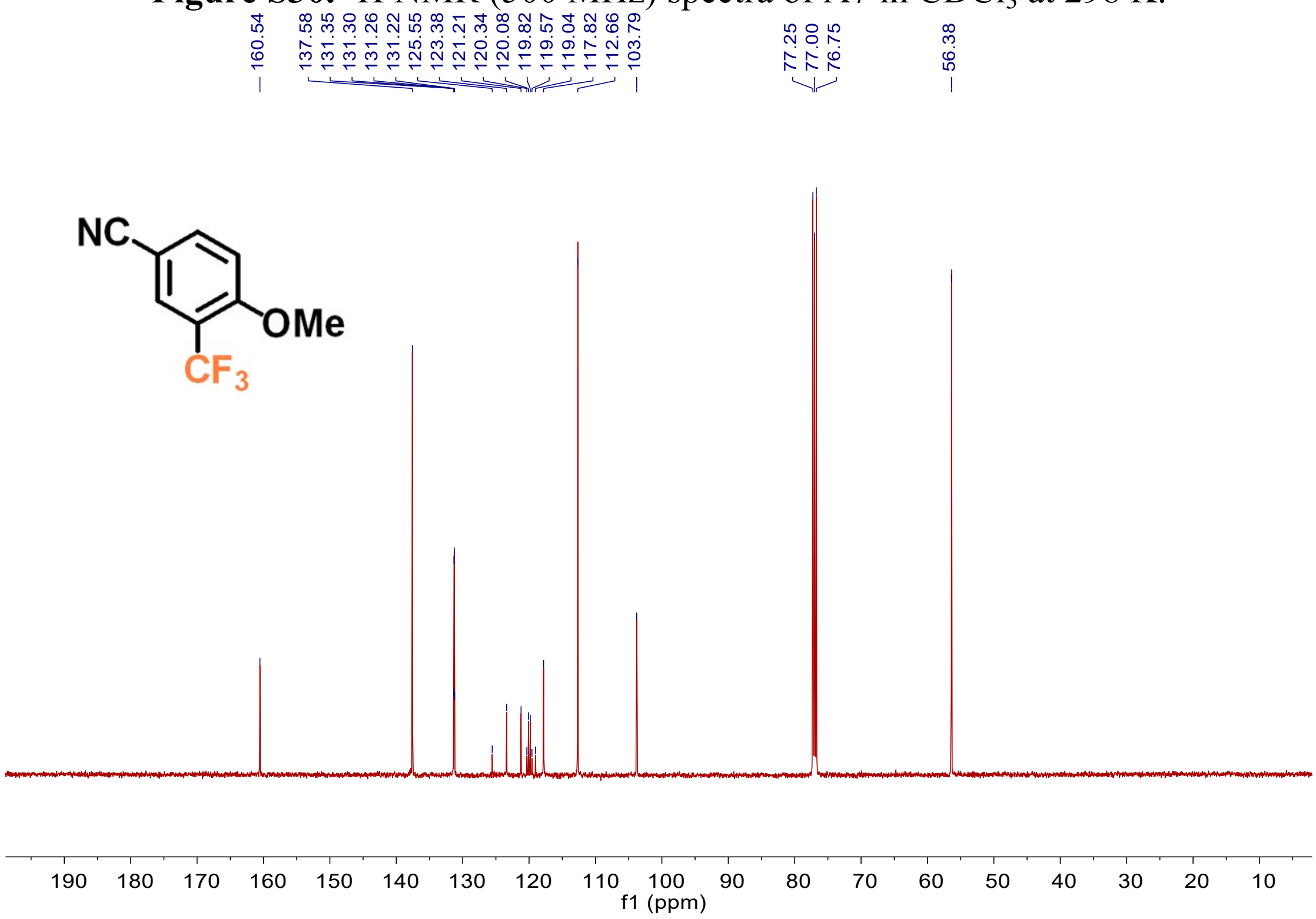

Figure S31. ^{13}C NMR (126 MHz) spectra of A7 in $CDCl_3$ at 298 K.

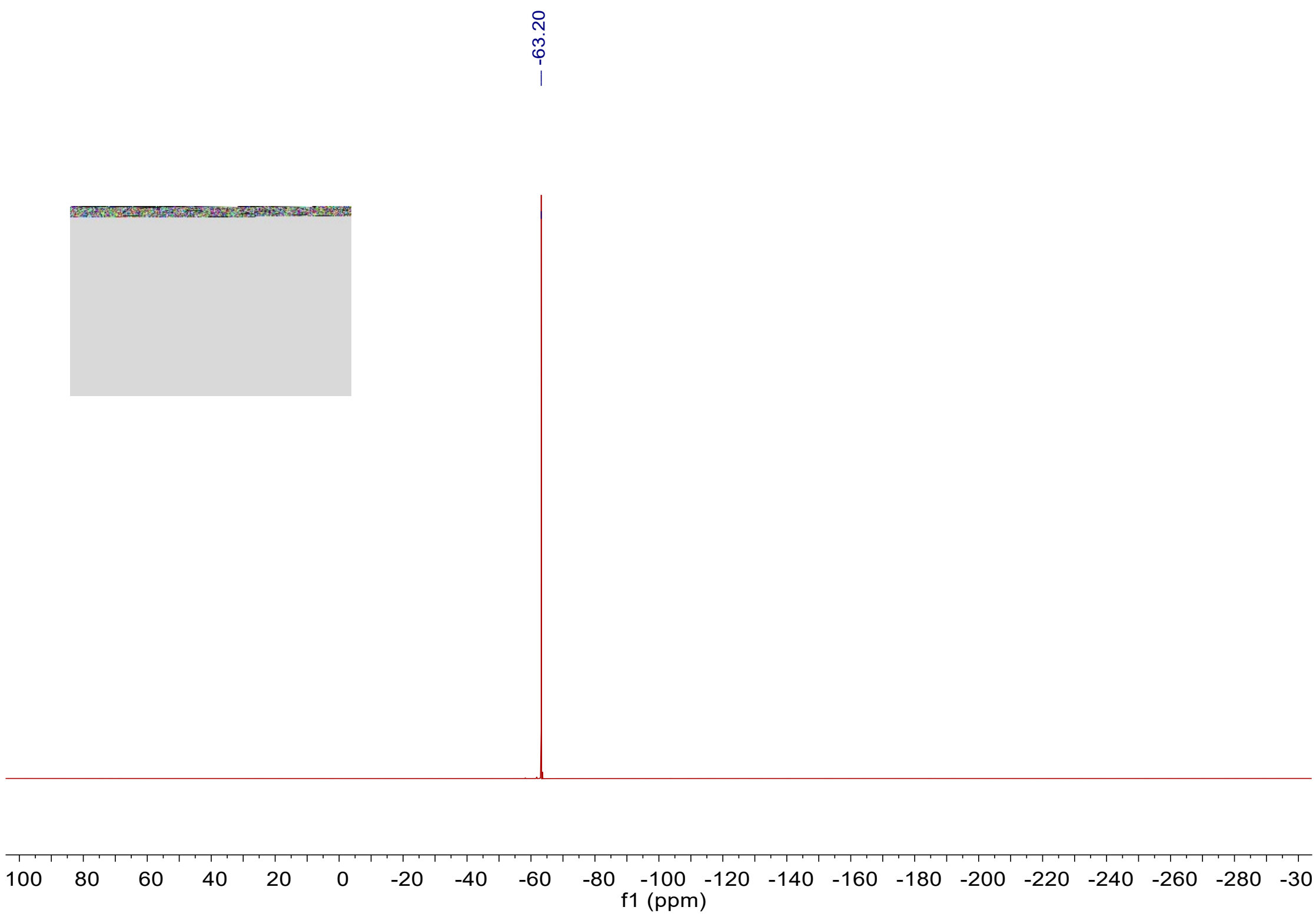

Figure S32. ^{19}F NMR (471 MHz) spectra of A7 in $CDCl_3$ at 298 K.

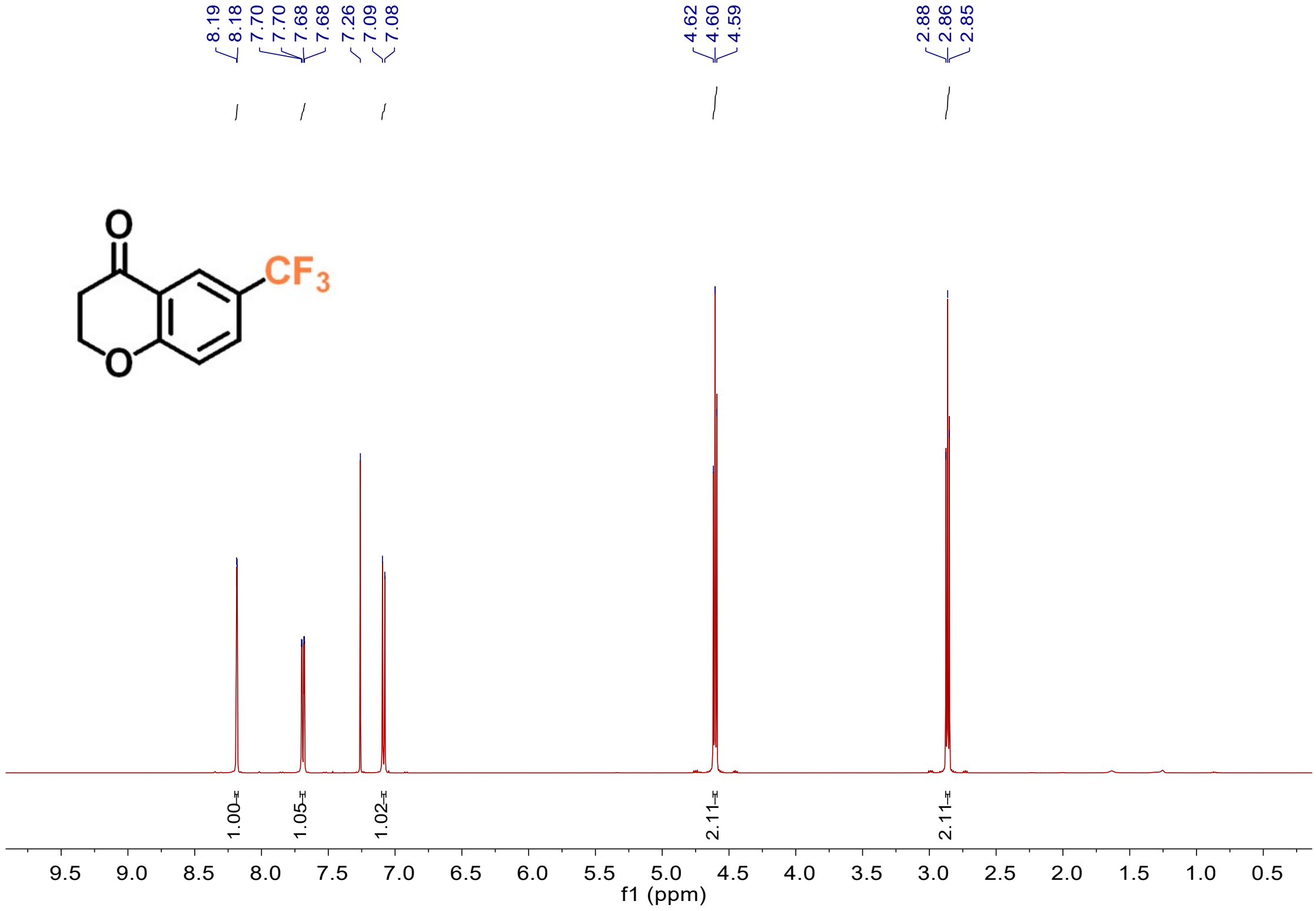

Figure S33. ^{1}H NMR (500 MHz) spectra of A8 in $CDCl_3$ at 298 K.

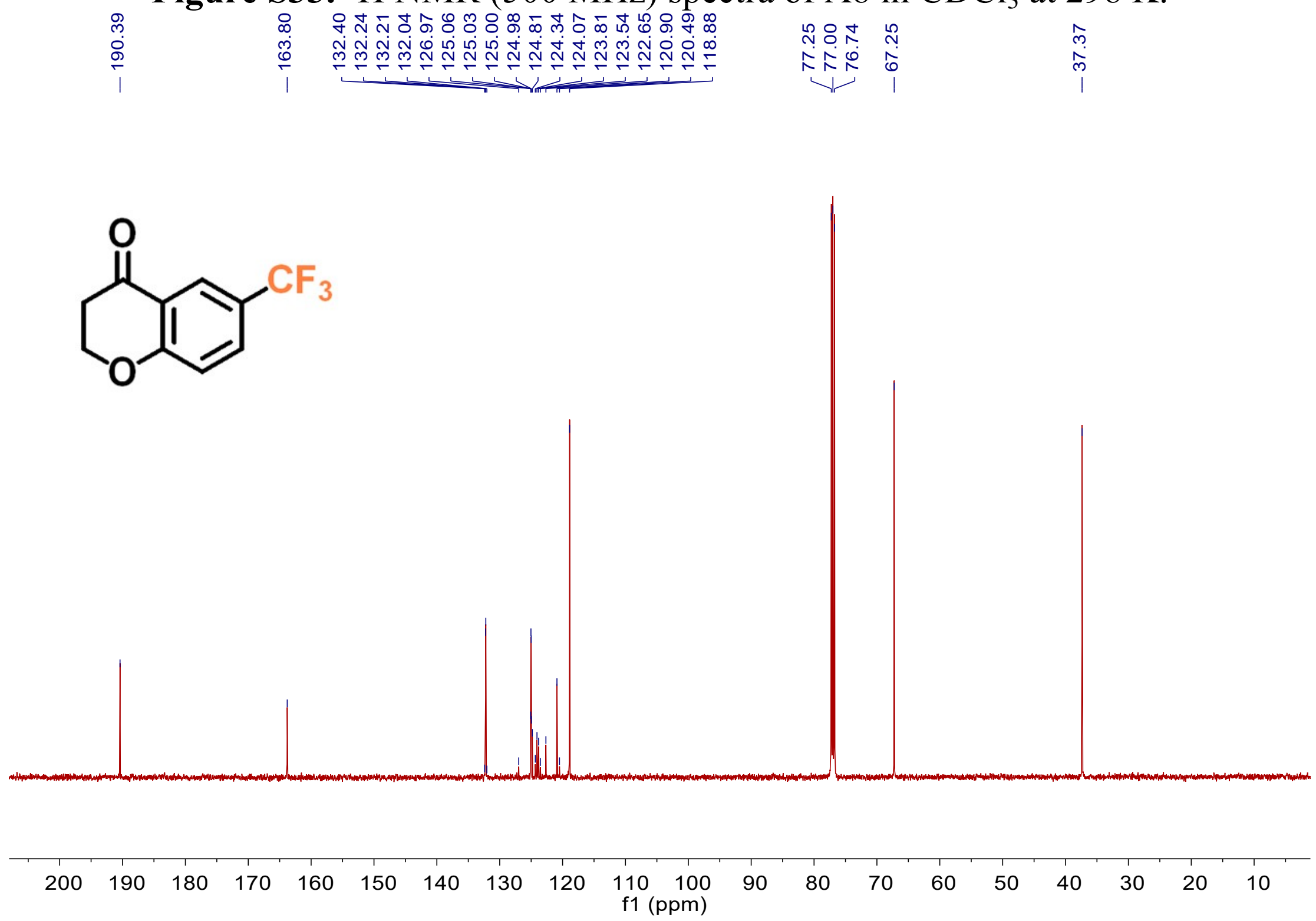

Figure S34. ^{13}C NMR (126 MHz) spectra of A8 in $CDCl_3$ at 298 K.

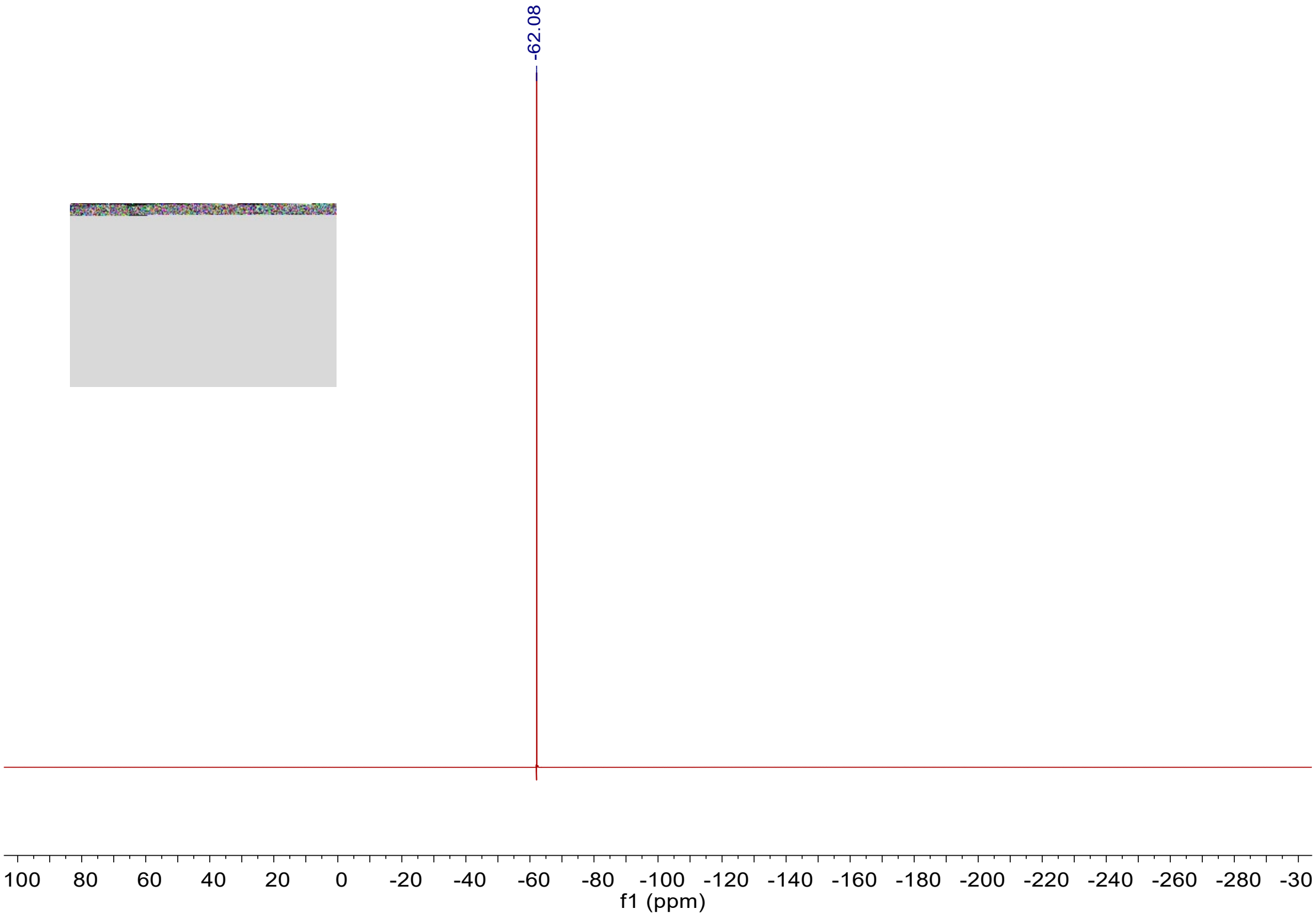

Figure S35. ^{19}F NMR (471 MHz) spectra of A8 in $CDCl_3$ at 298 K.

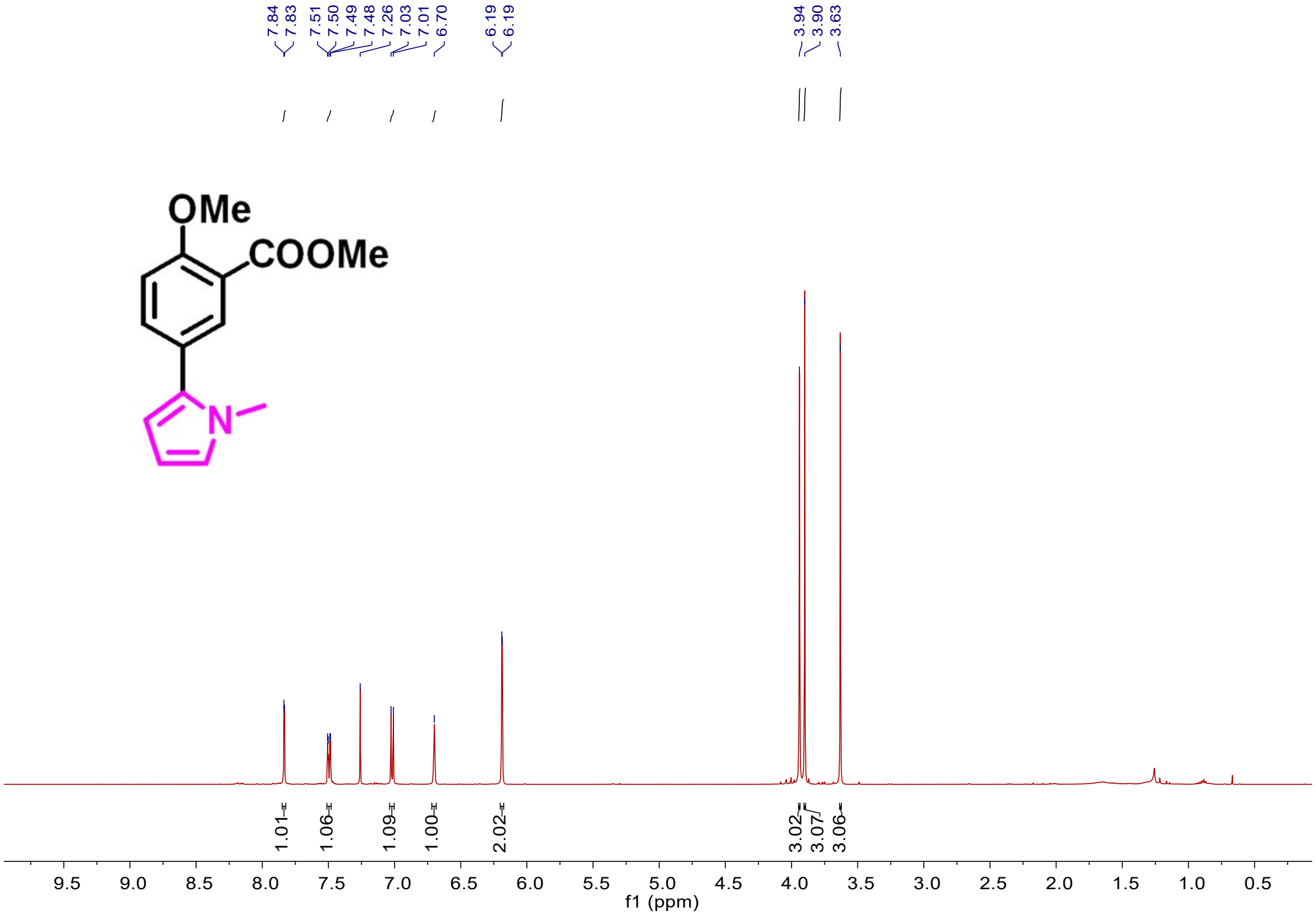

Figure S36. ^{1}H NMR (500 MHz) spectra of B1 in $CDCl_3$ at 298 K.

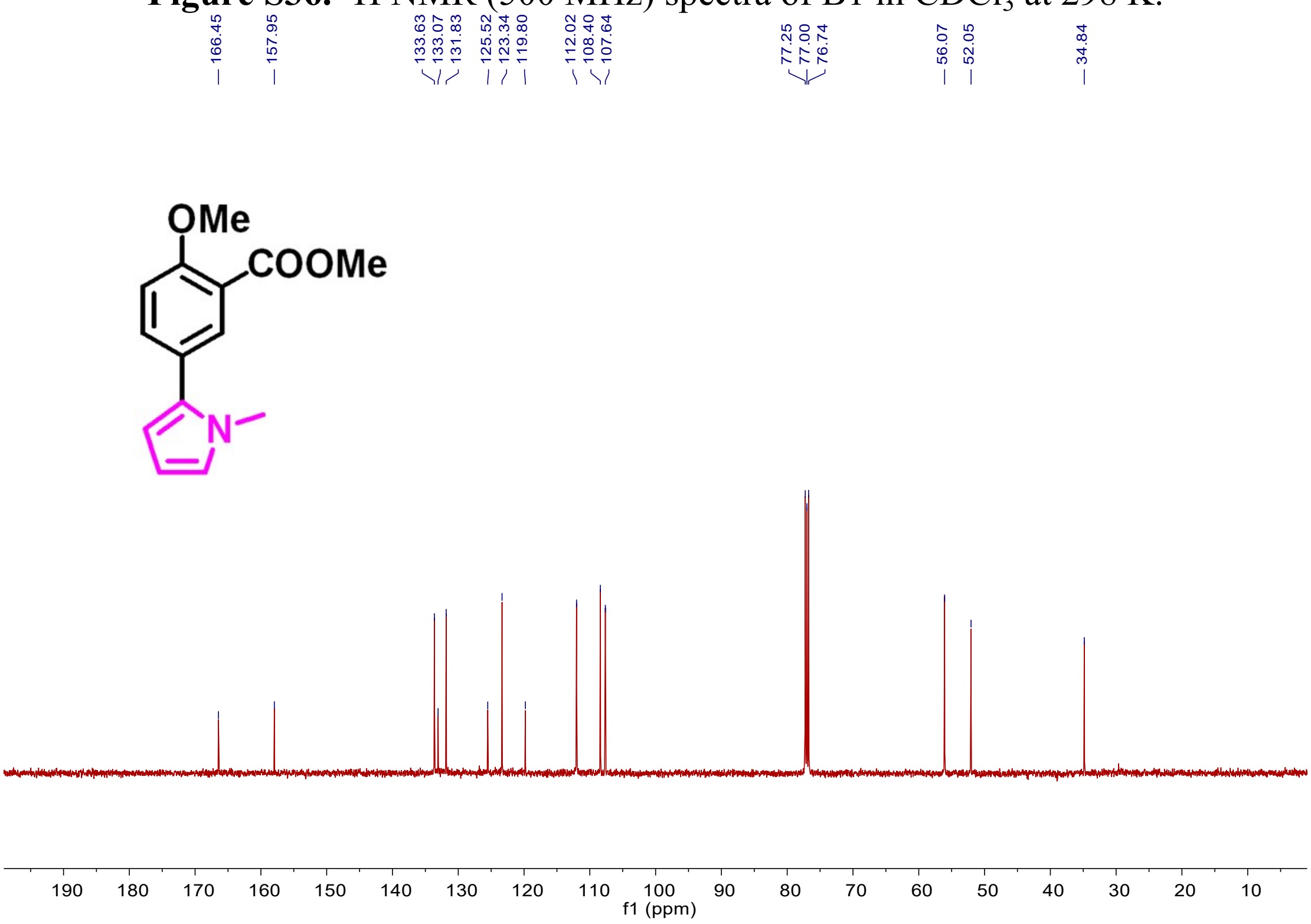

Figure S37. ^{13}C NMR (126 MHz) spectra of B1 in $CDCl_3$ at 298 K.

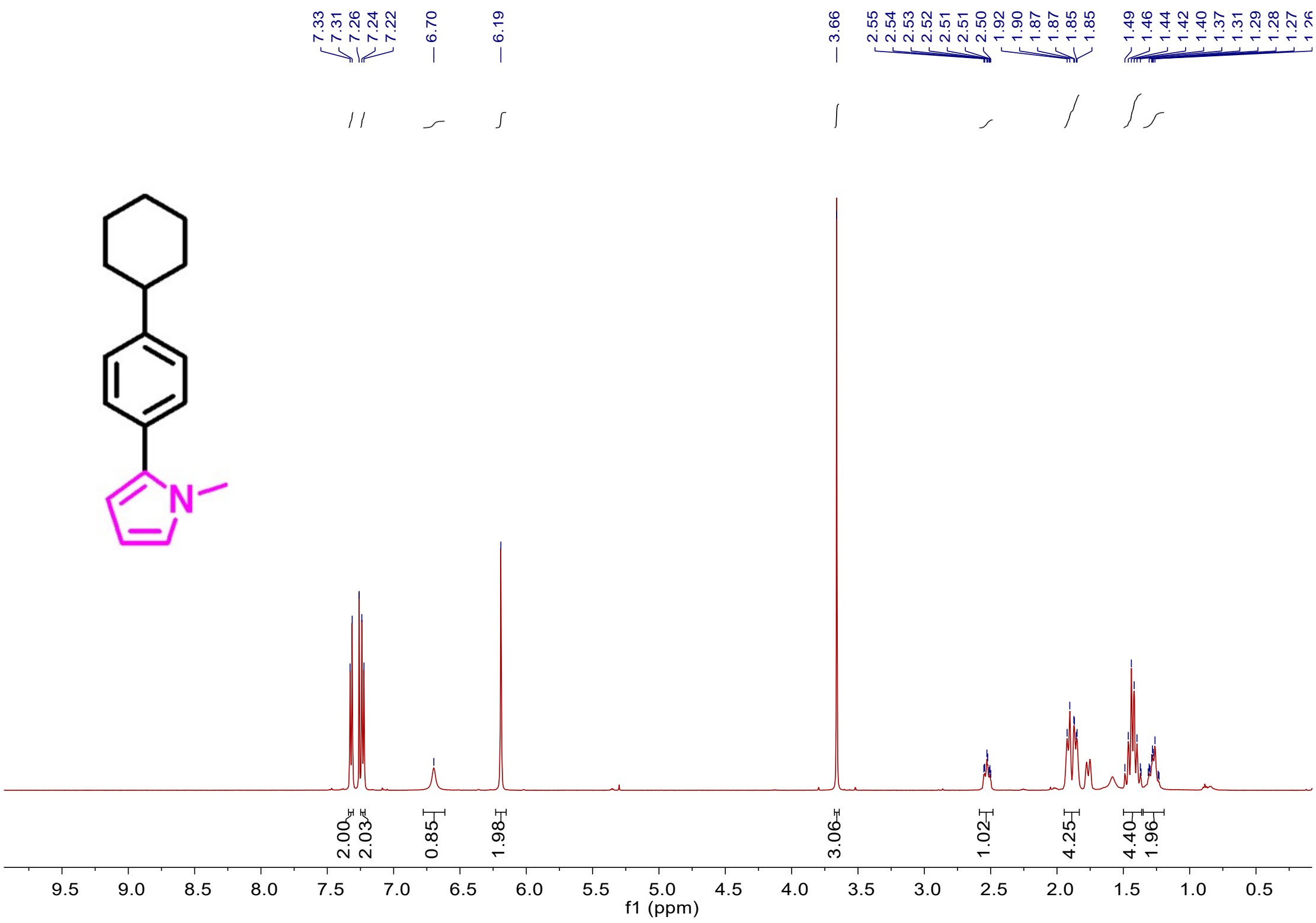

Figure S38. ^1H NMR (500 MHz) spectra of B2 in $CDCl_3$ at 298 K.

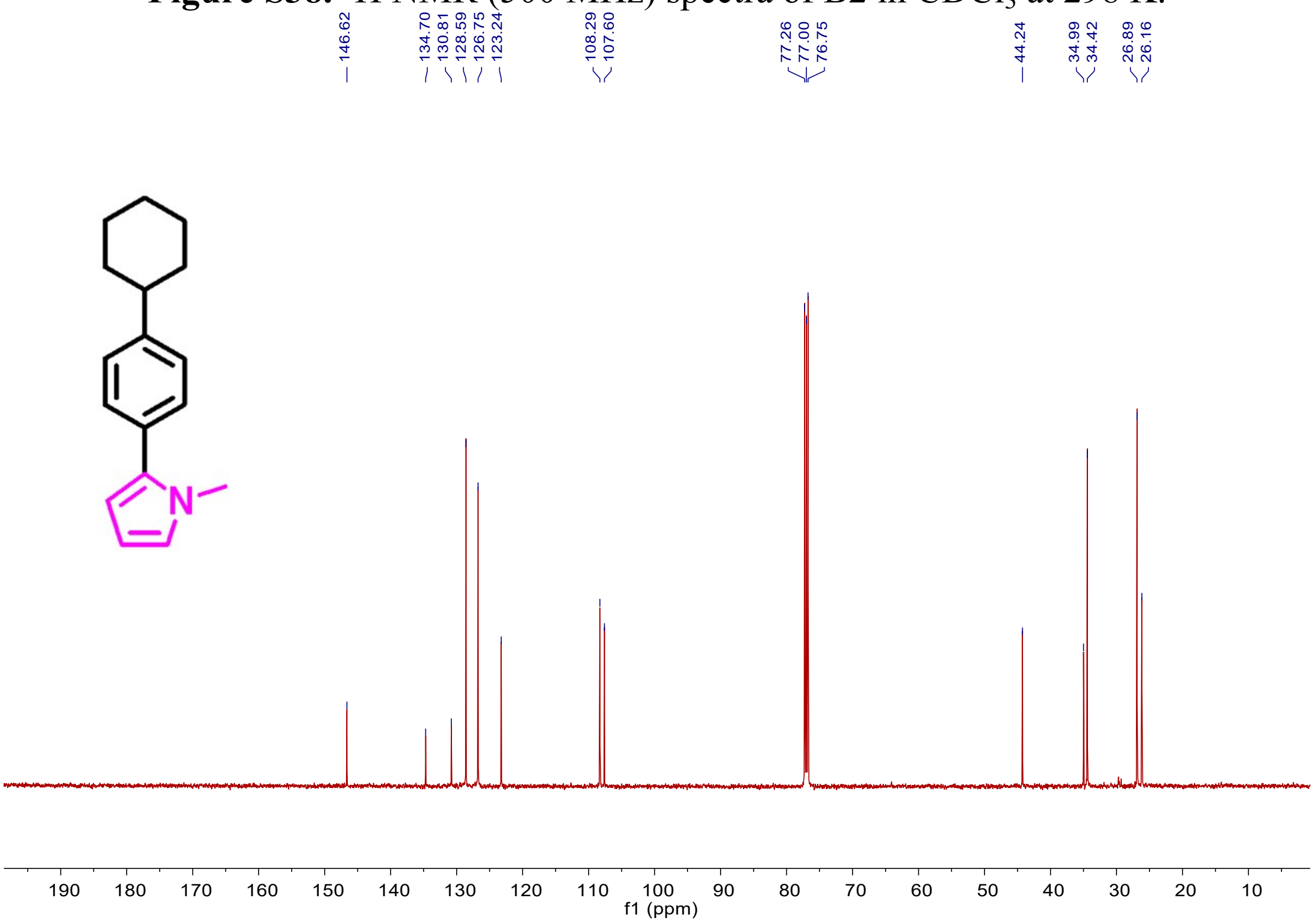

Figure S39. ^{13}C NMR (126 MHz) spectra of B2 in $CDCl_3$ at 298 K.

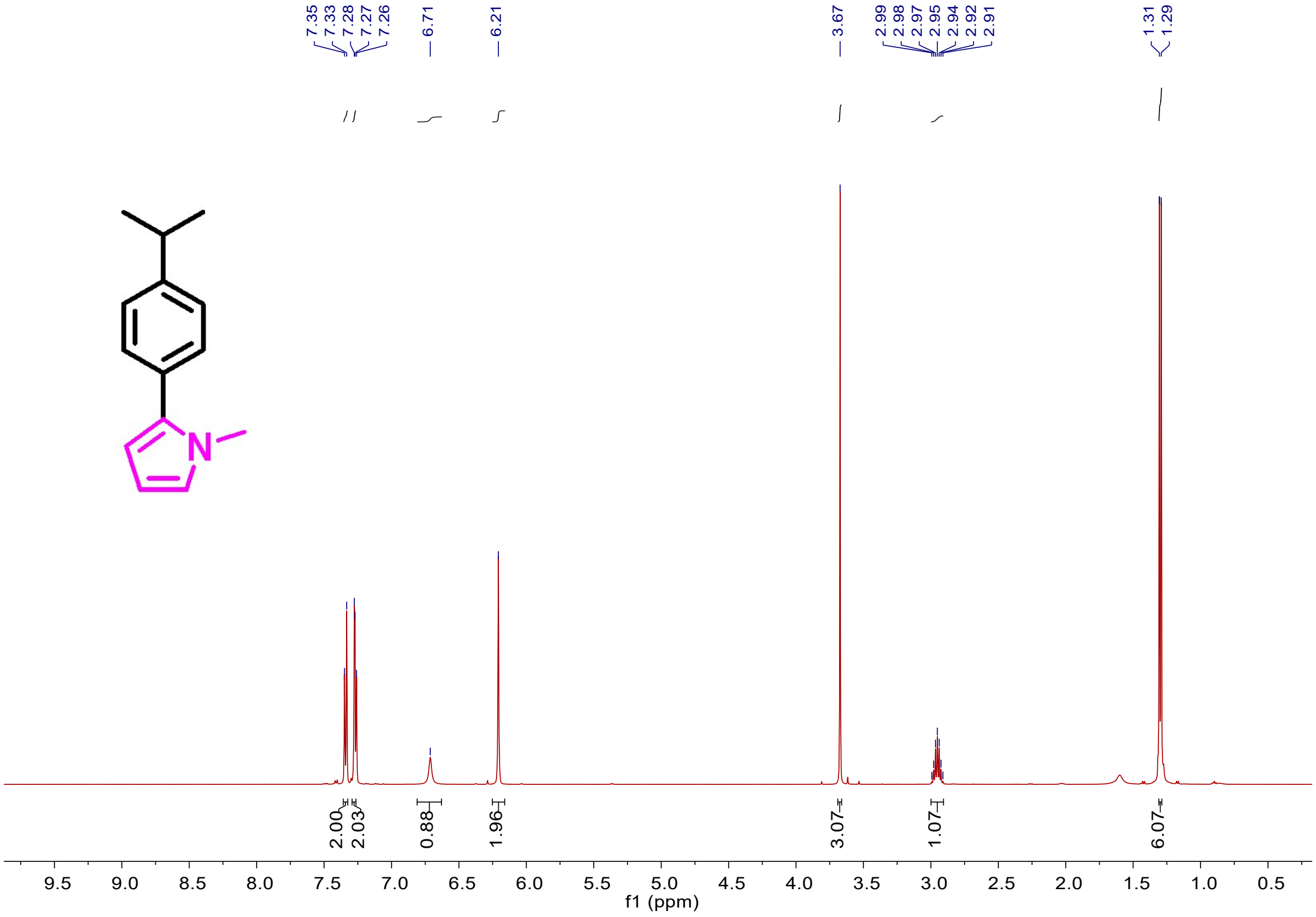

Figure S40. ^{1}H NMR (500 MHz) spectra of B3 in $CDCl_3$ at 298 K.

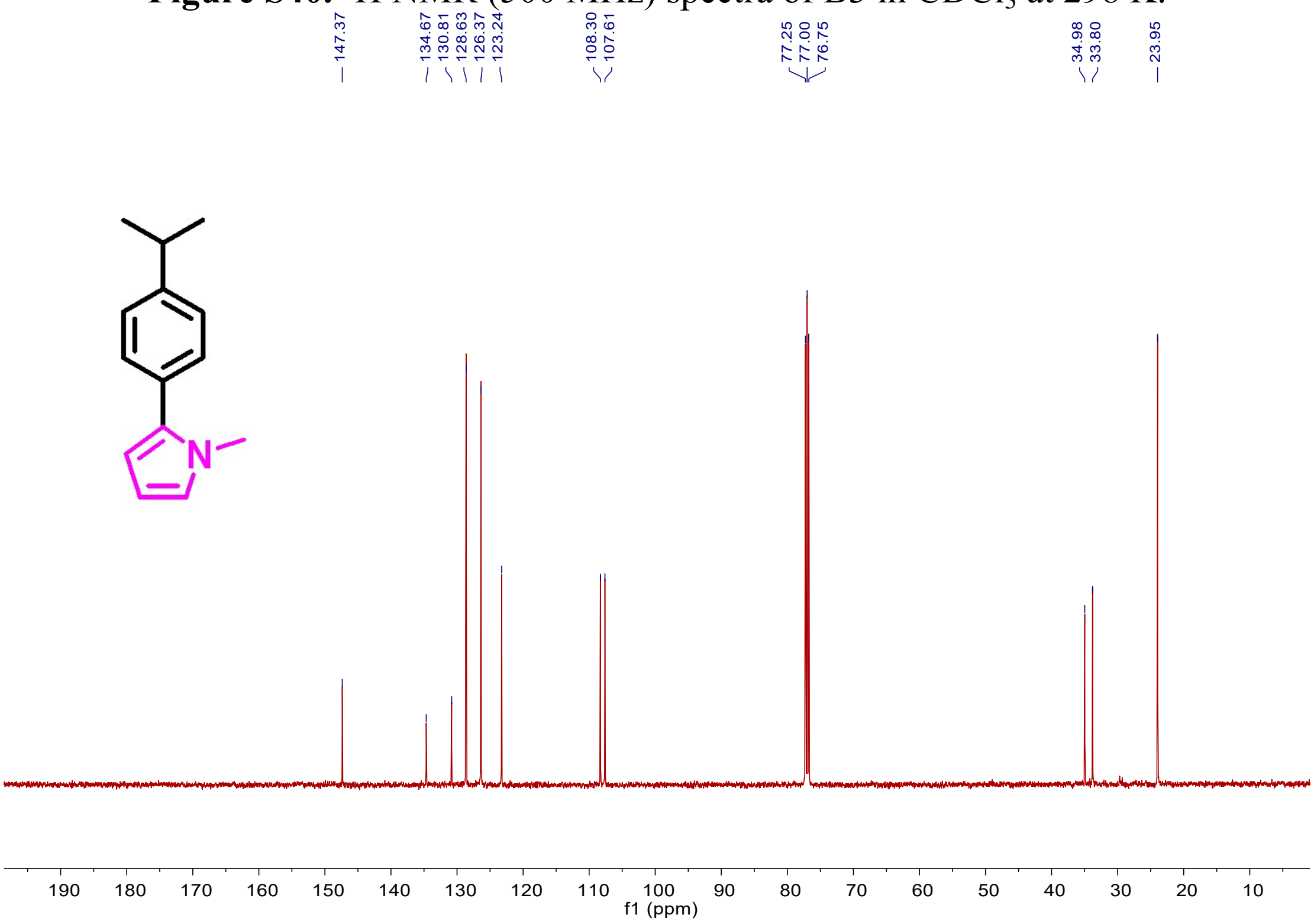

Figure S41. ^{13}C NMR (126 MHz) spectra of B3 in $CDCl_3$ at 298 K.

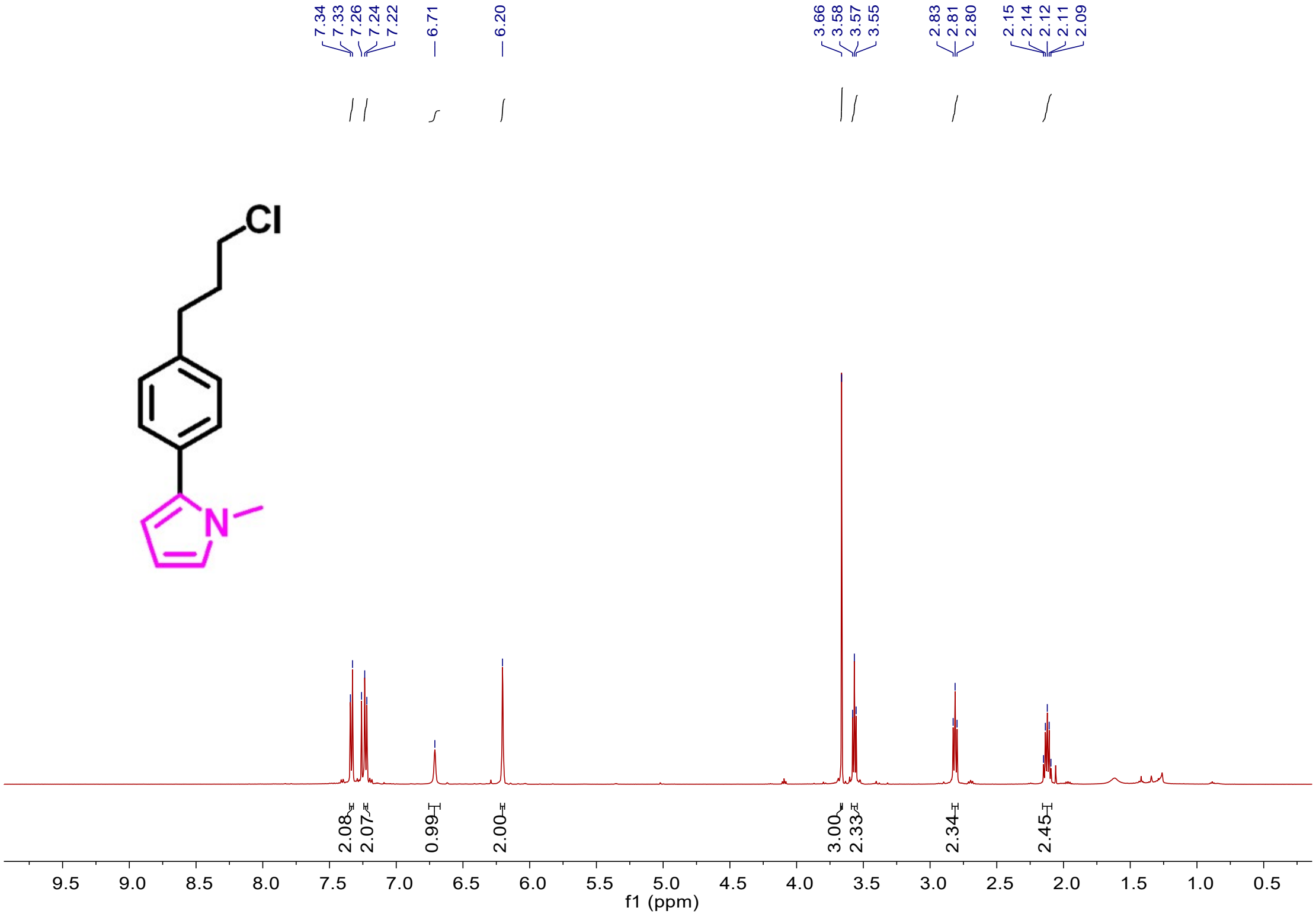

Figure S42. ^{1}H NMR (500 MHz) spectra of B4 in $CDCl_3$ at 298 K.

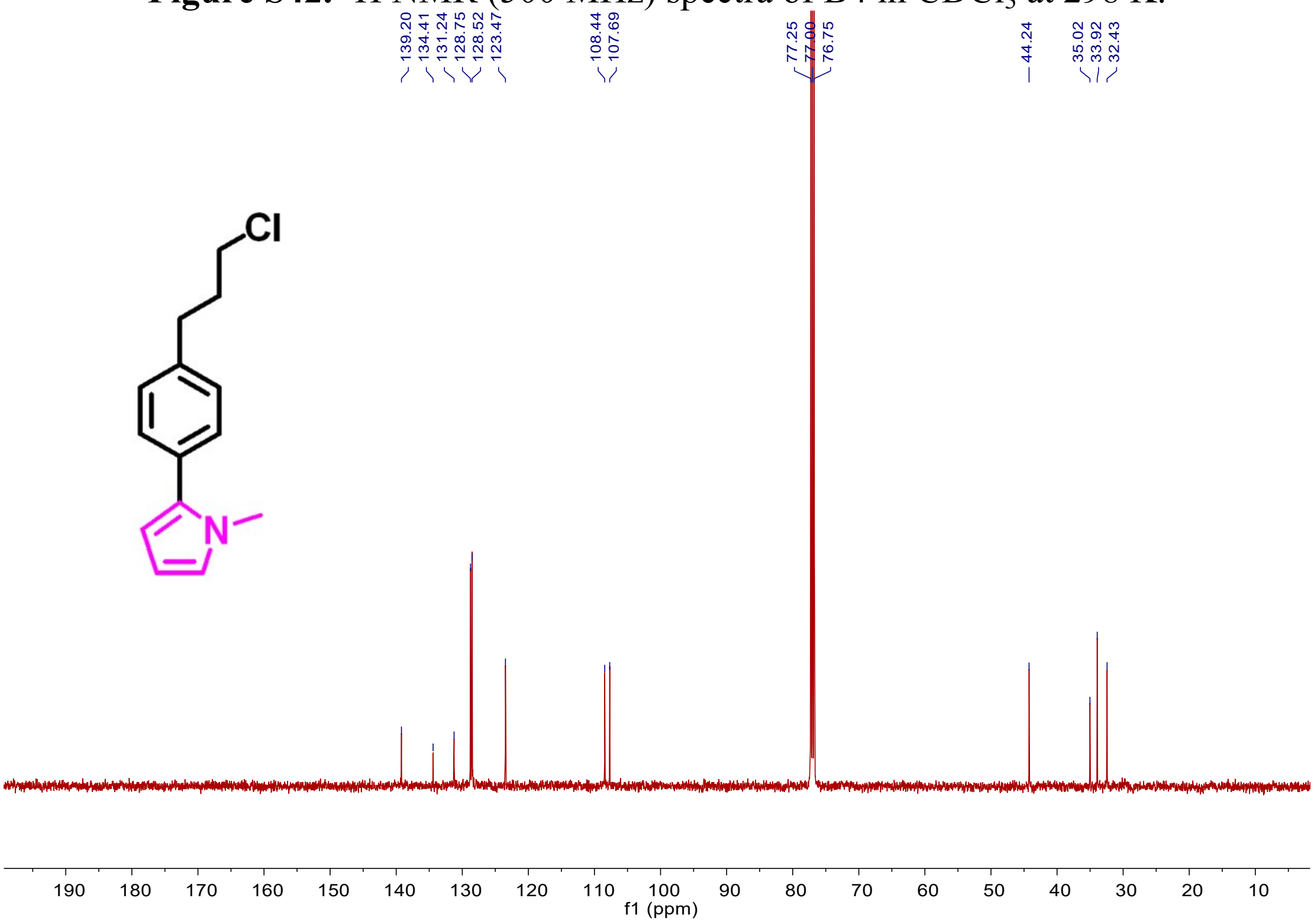

Figure S43. ^{13}C NMR (126 MHz) spectra of B4 in $CDCl_3$ at 298 K.

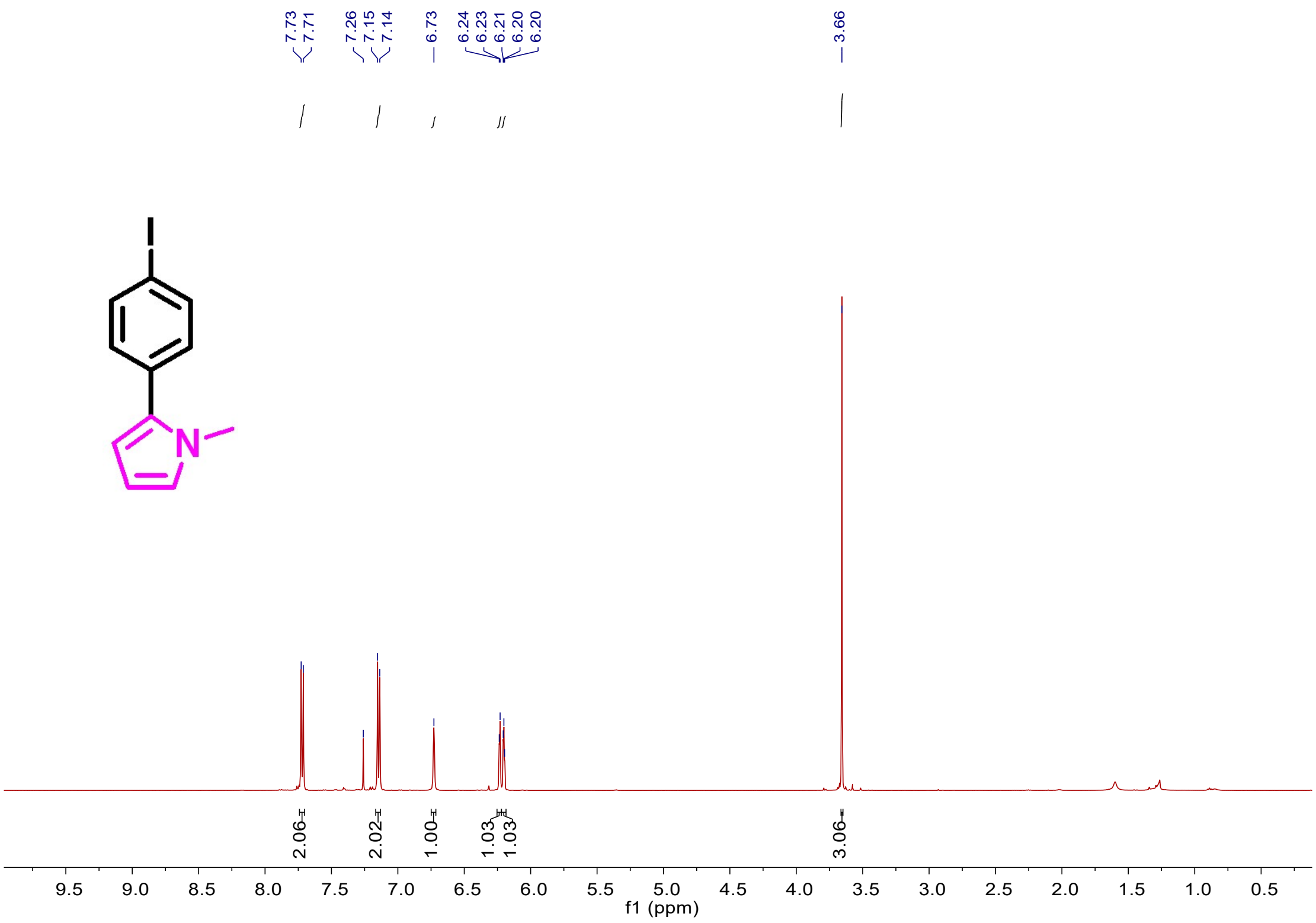

Figure S44. ^{1}H NMR (500 MHz) spectra of B5 in $CDCl_3$ at 298 K.

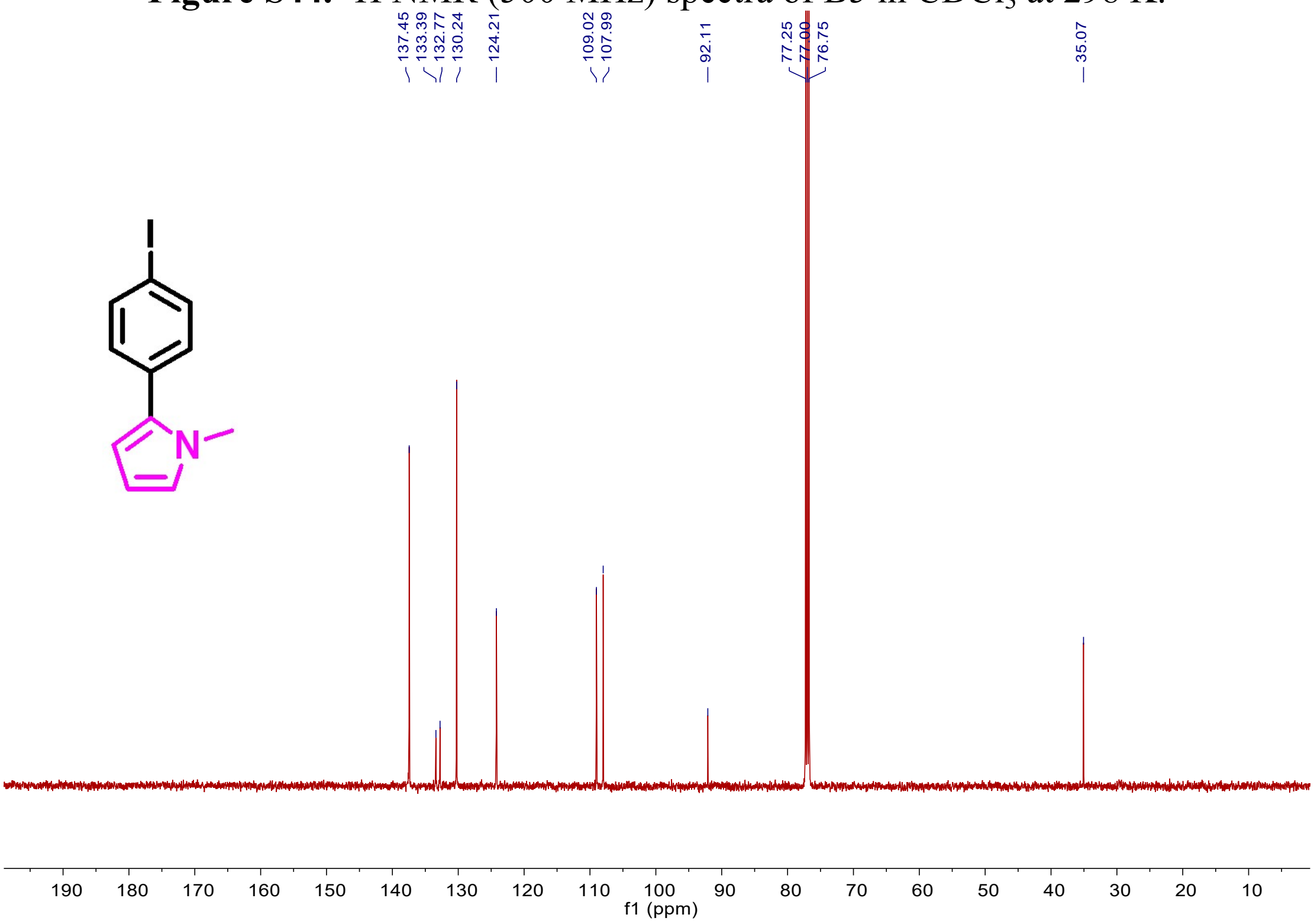

Figure S45. ^{13}C NMR (126 MHz) spectra of B5 in $CDCl_3$ at 298 K.

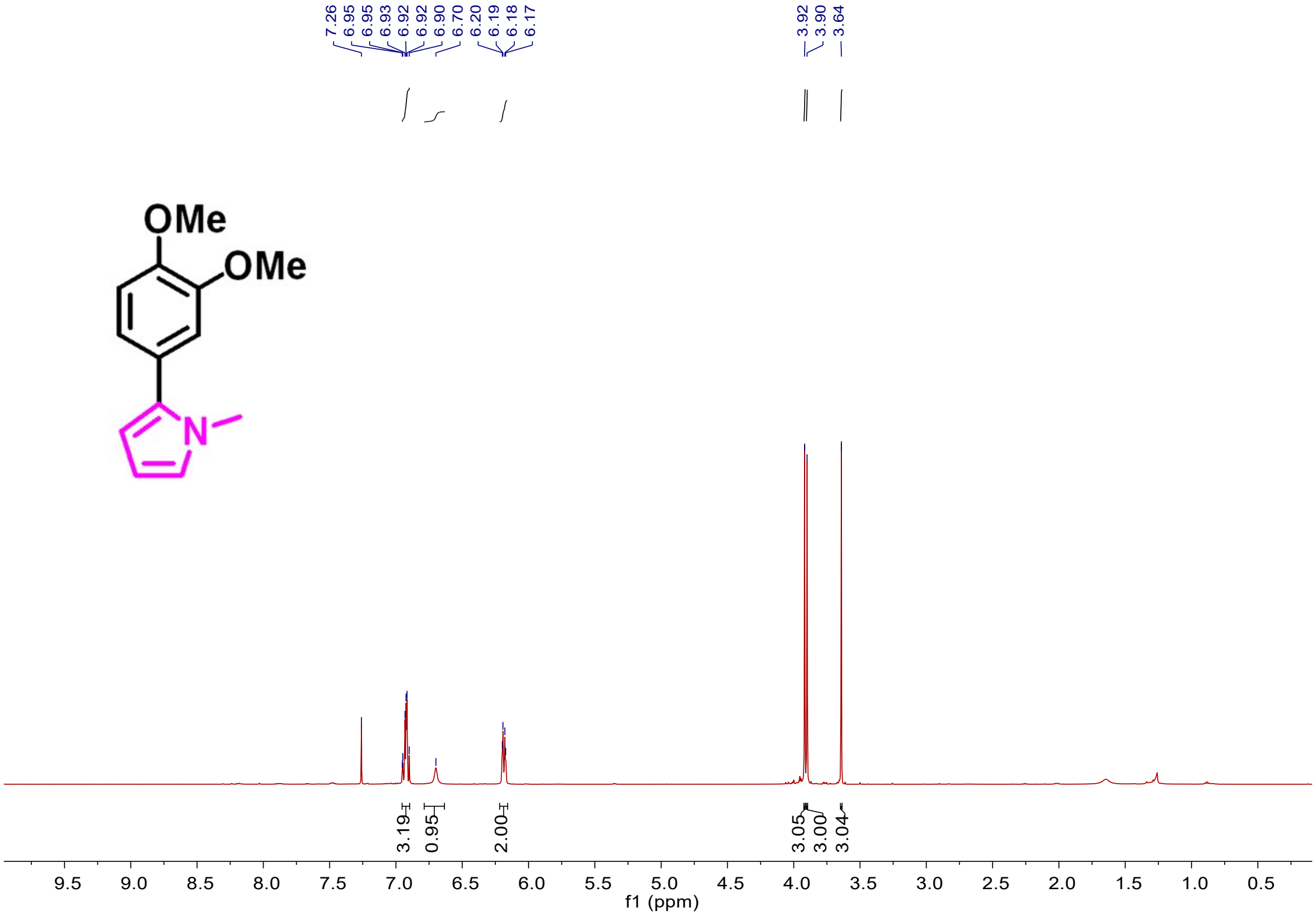

Figure S46. ^{1}H NMR (500 MHz) spectra of B6 in $CDCl_3$ at 298 K.

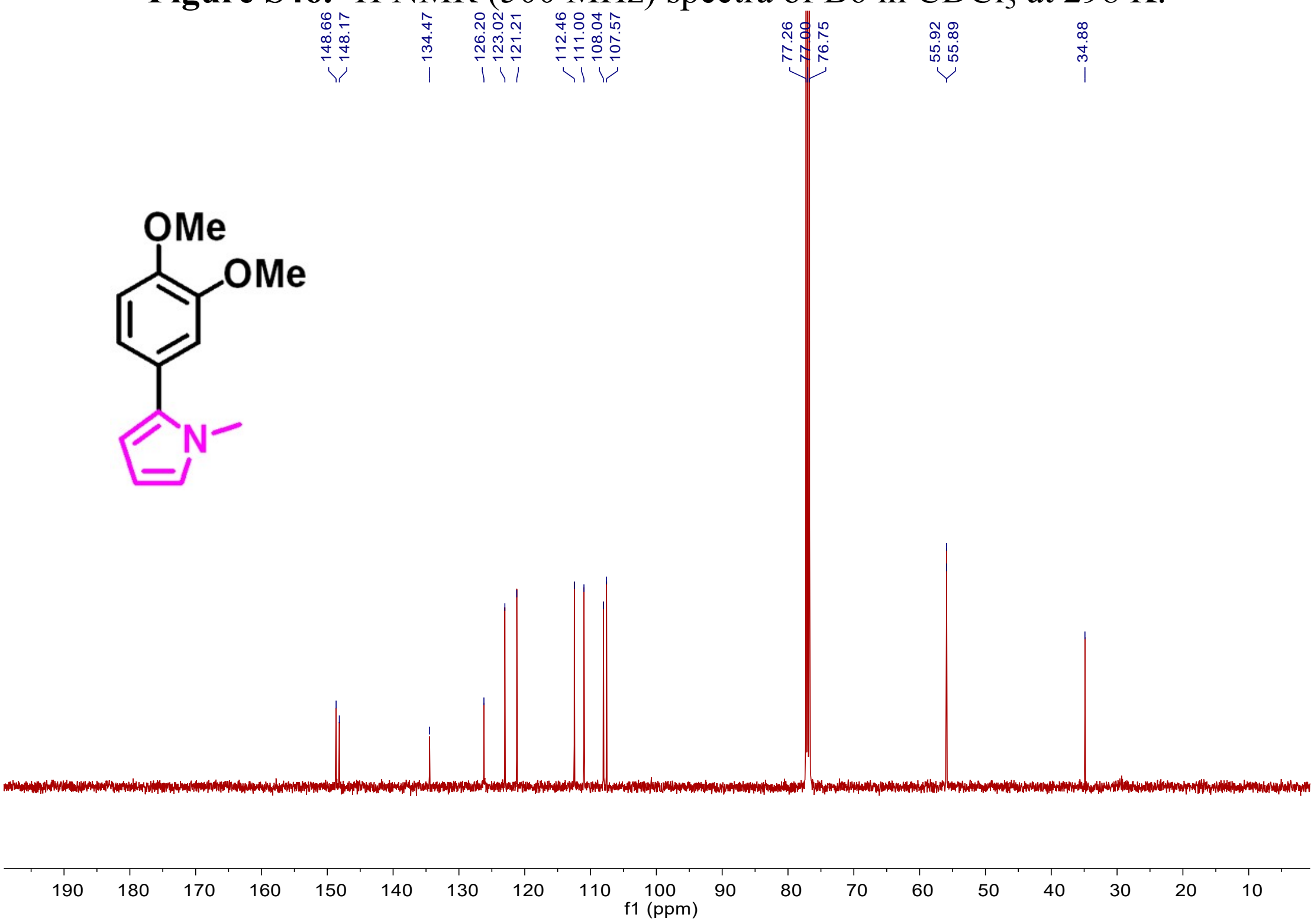

Figure S47. ^{13}C NMR (126 MHz) spectra of B6 in $CDCl_3$ at 298 K.

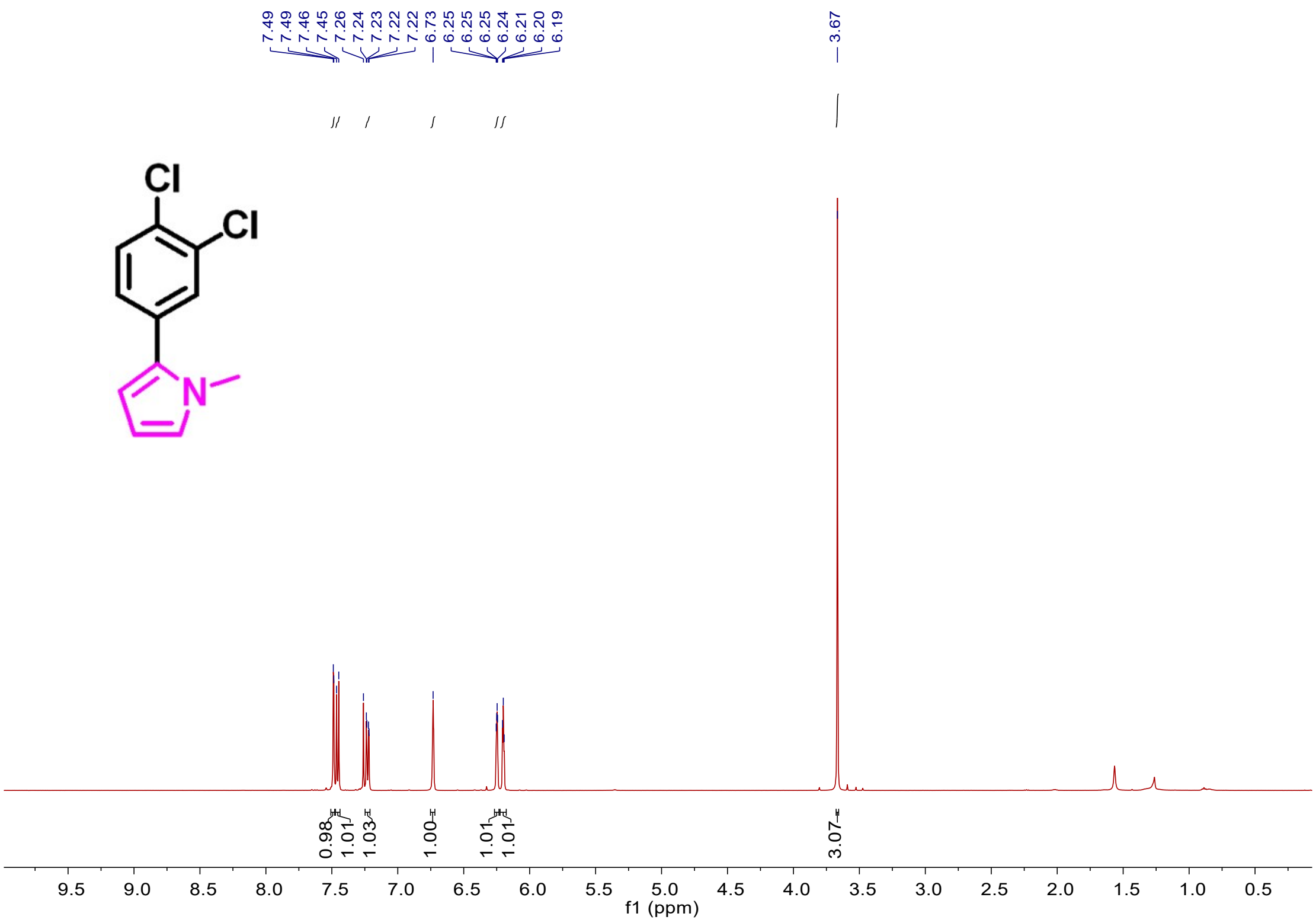

Figure S48. ^{1}H NMR (500 MHz) spectra of B7 in $CDCl_3$ at 298 K.

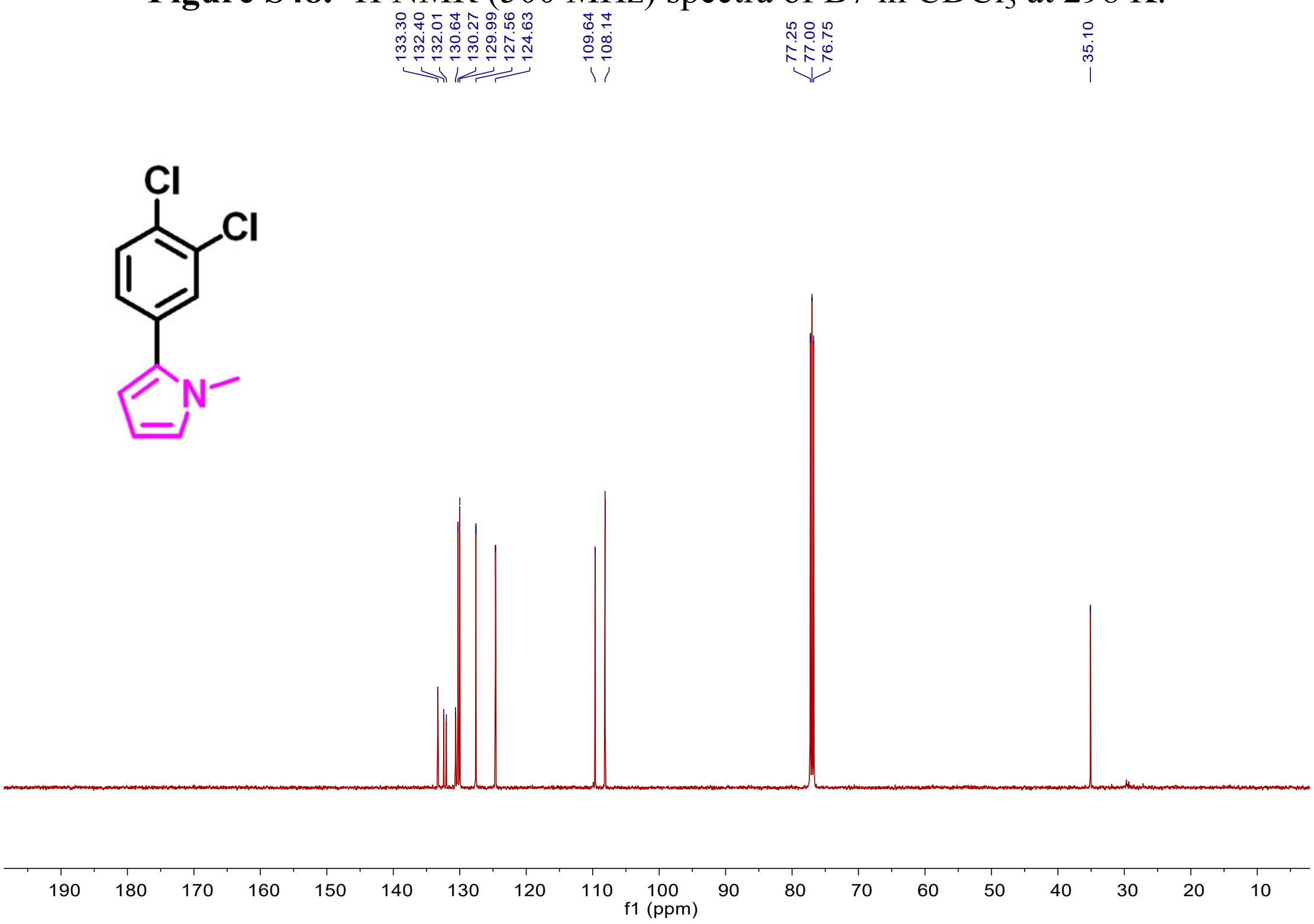

Figure S49. ^{13}C NMR (126 MHz) spectra of B7 in $CDCl_3$ at 298 K.

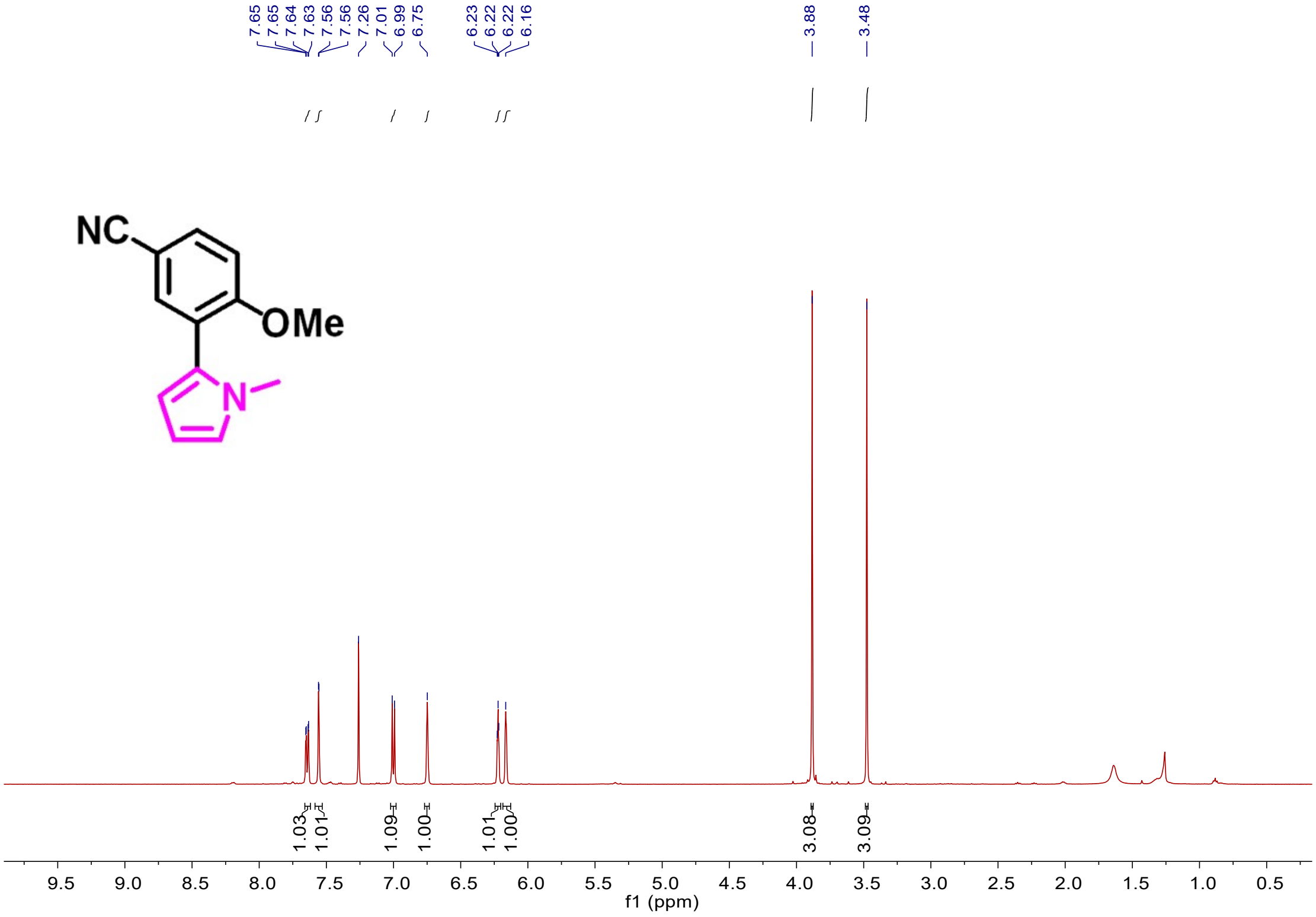

Figure S50. ^{1}H NMR (500 MHz) spectra of B8 in $CDCl_3$ at 298 K.

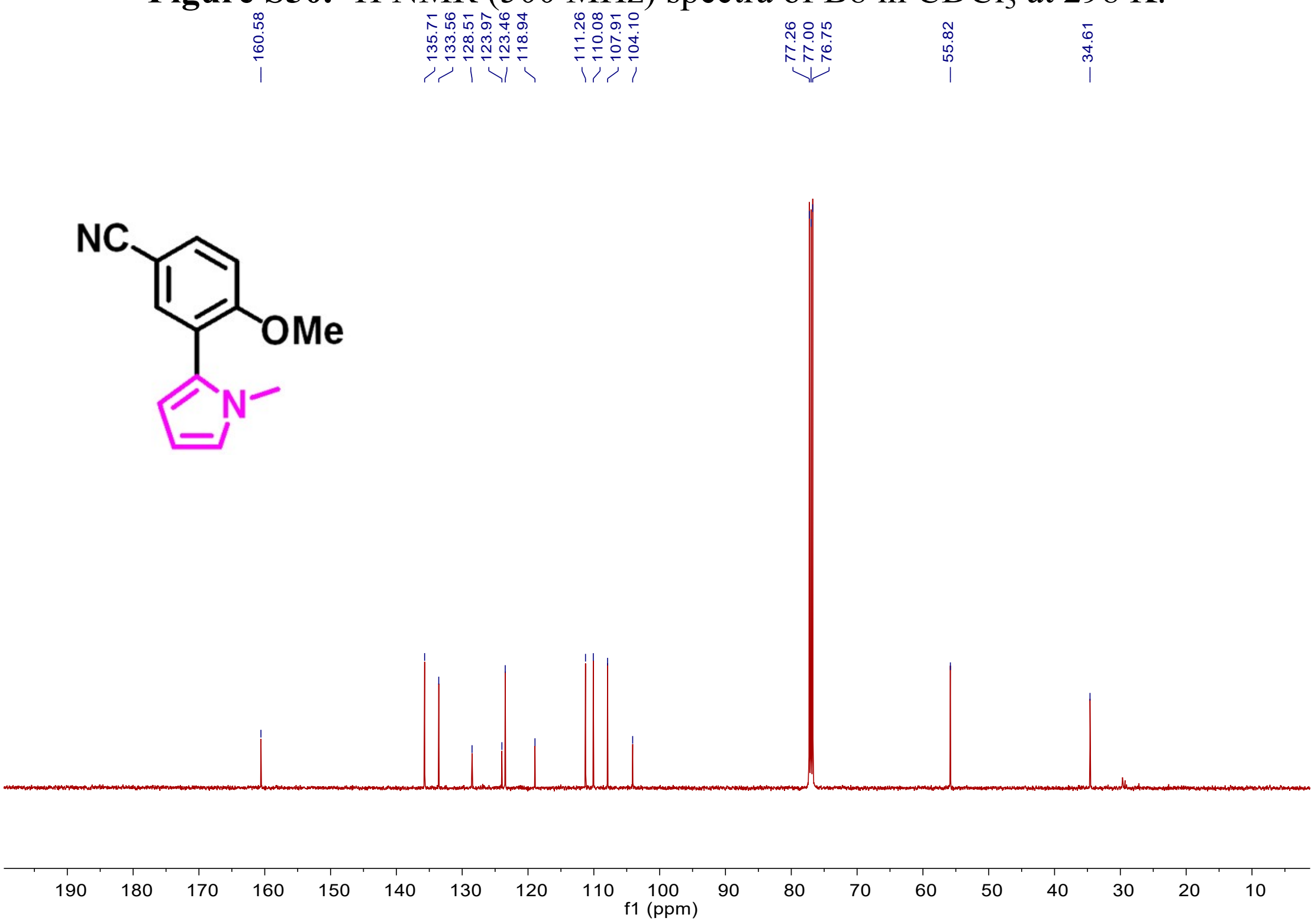

Figure S51. ^{13}C NMR (126 MHz) spectra of B8 in $CDCl_3$ at 298 K.

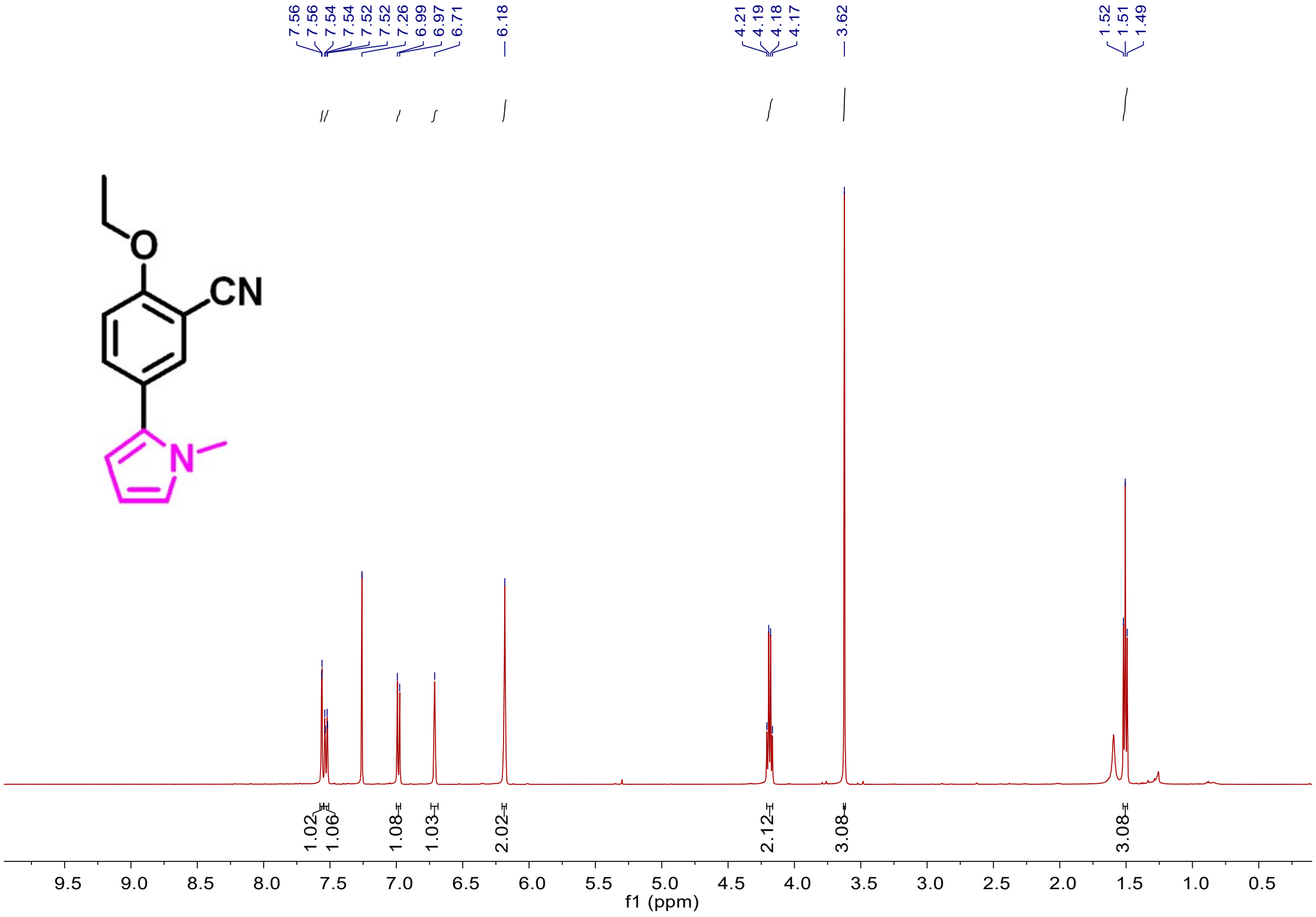

Figure S52. ^{1}H NMR (500 MHz) spectra of B9 in $CDCl_3$ at 298 K.

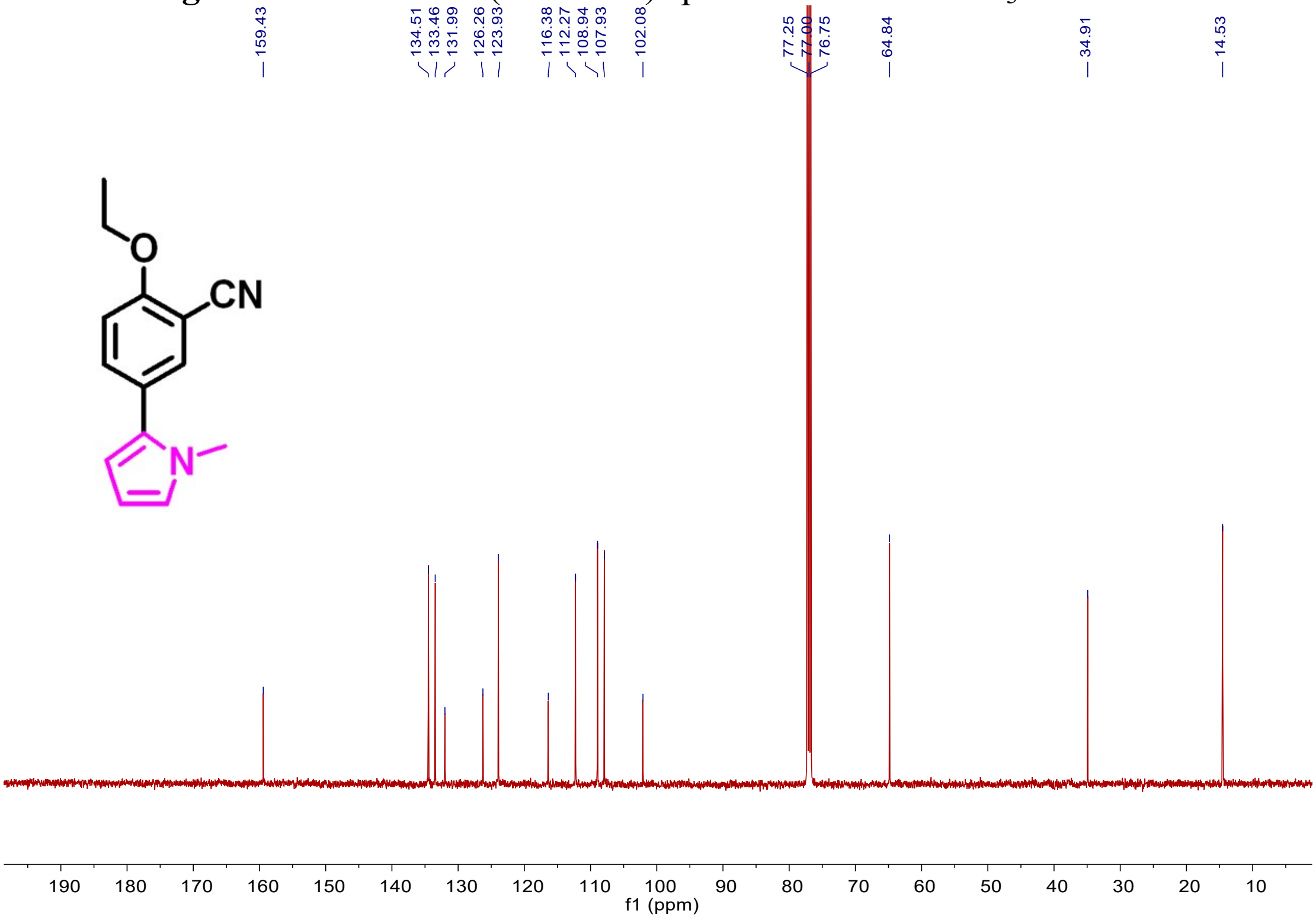

Figure S53. ^{13}C NMR (126 MHz) spectra of B9 in $CDCl_3$ at 298 K.

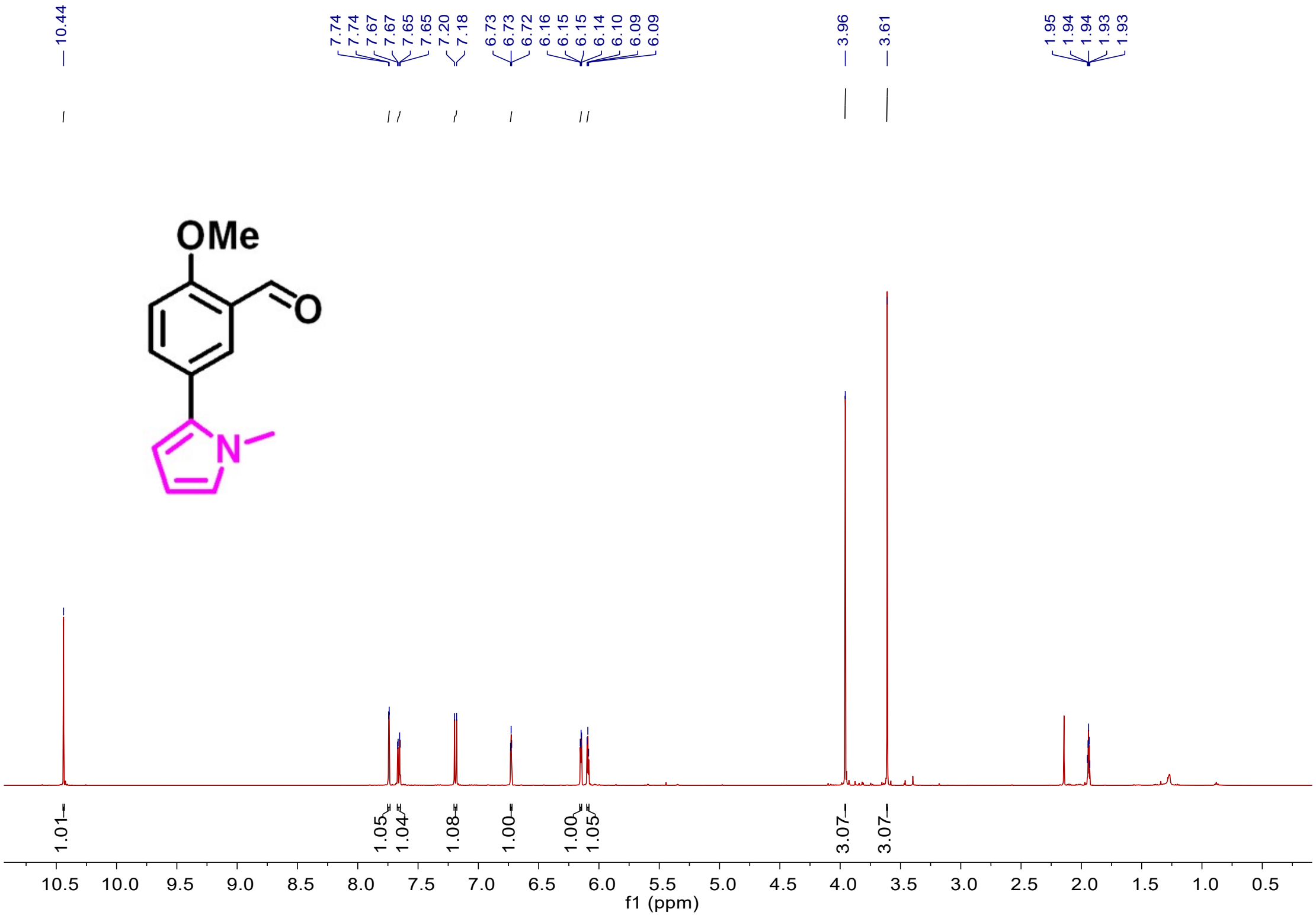

Figure S54. ^{1}H NMR (500 MHz) spectra of B10 in CD_3CN at 298 K.

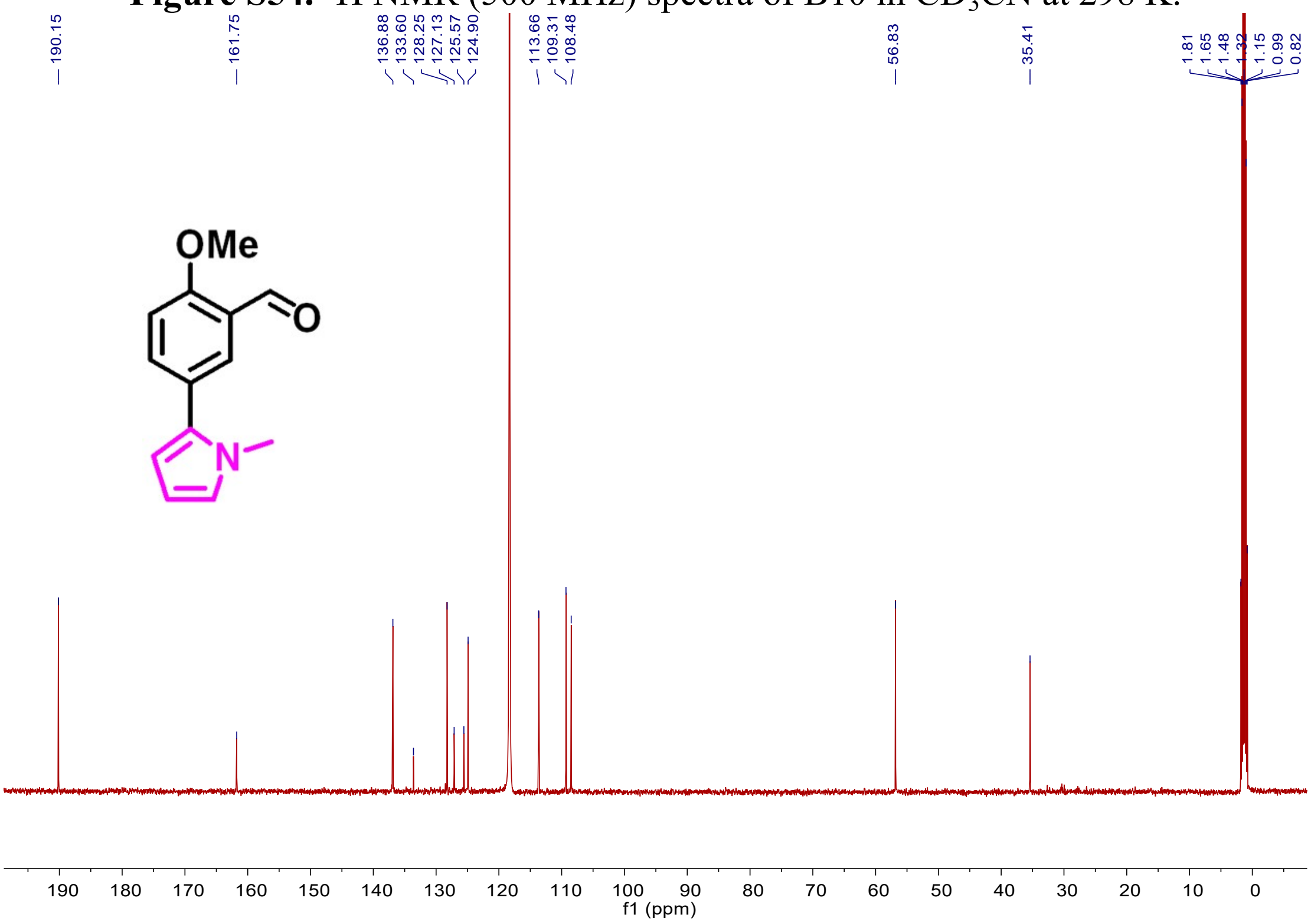

Figure S55. ^{13}C NMR (126 MHz) spectra of B10 in CD_3CN at 298 K.

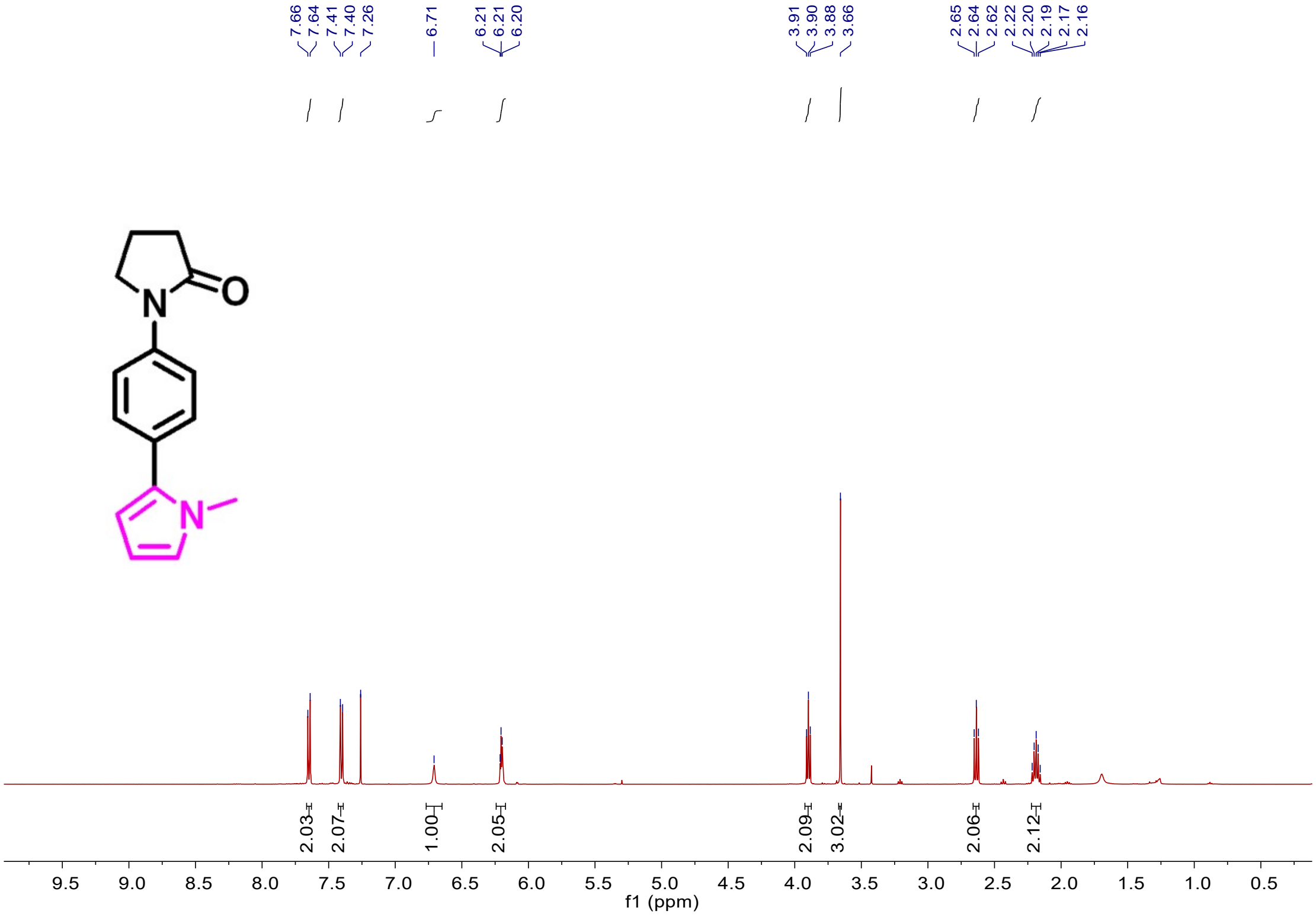

Figure S56. ^1H NMR (500 MHz) spectra of B11 in $CDCl_3$ at 298 K.

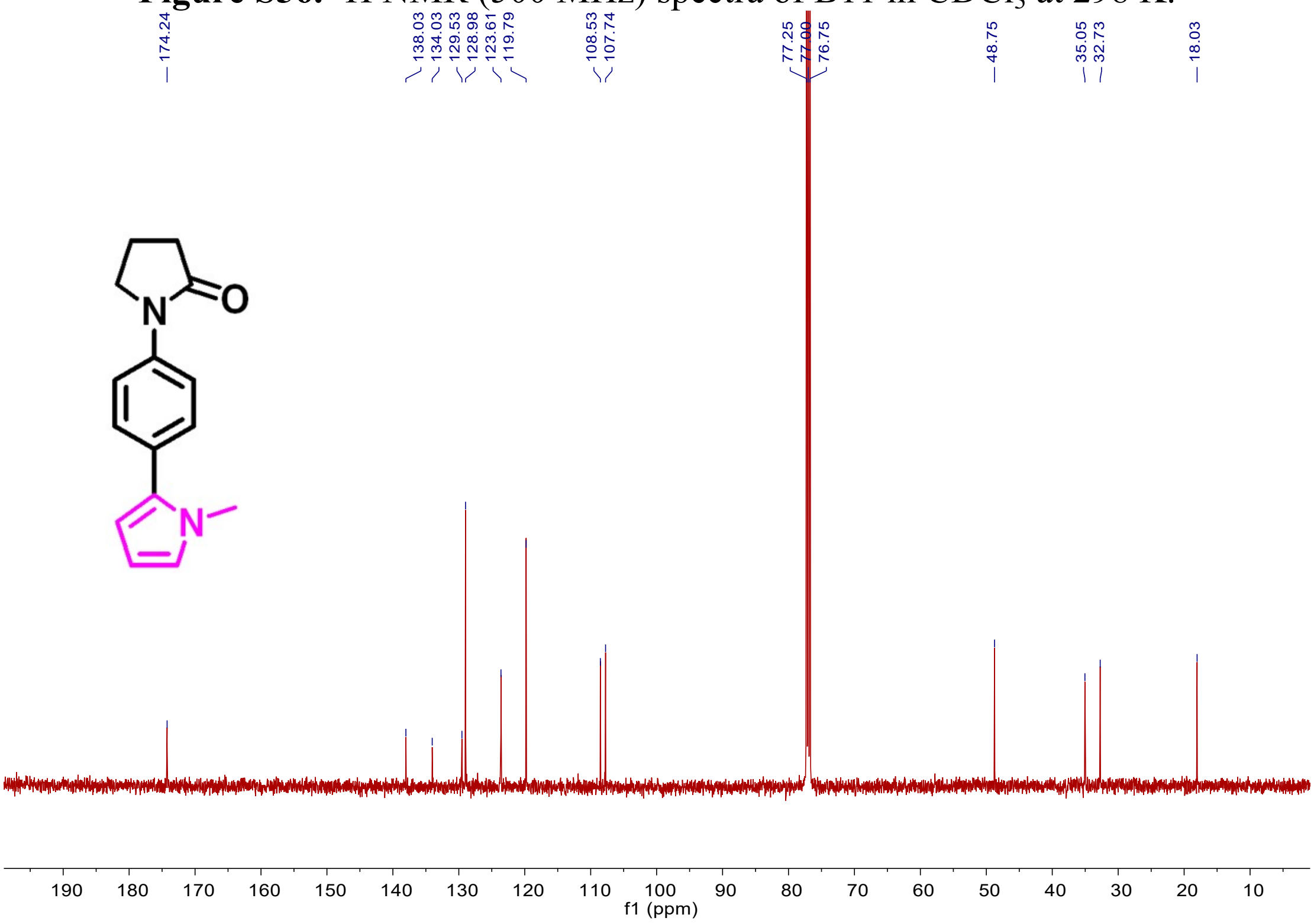

Figure S57. ^{13}C NMR (126 MHz) spectra of B11 in $CDCl_3$ at 298 K.

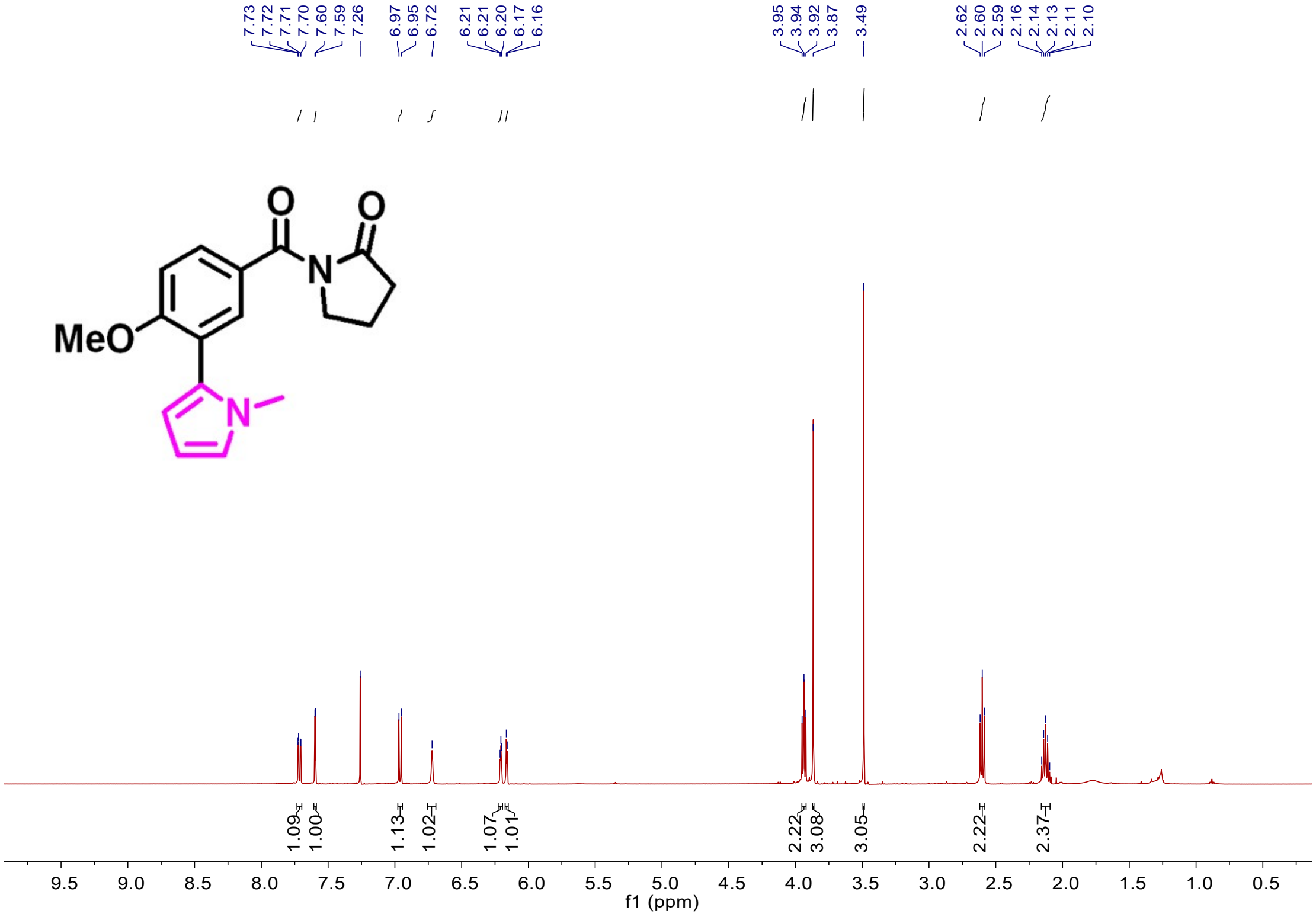

Figure S58. ^{1}H NMR (500 MHz) spectra of B12 in $CDCl_3$ at 298 K.

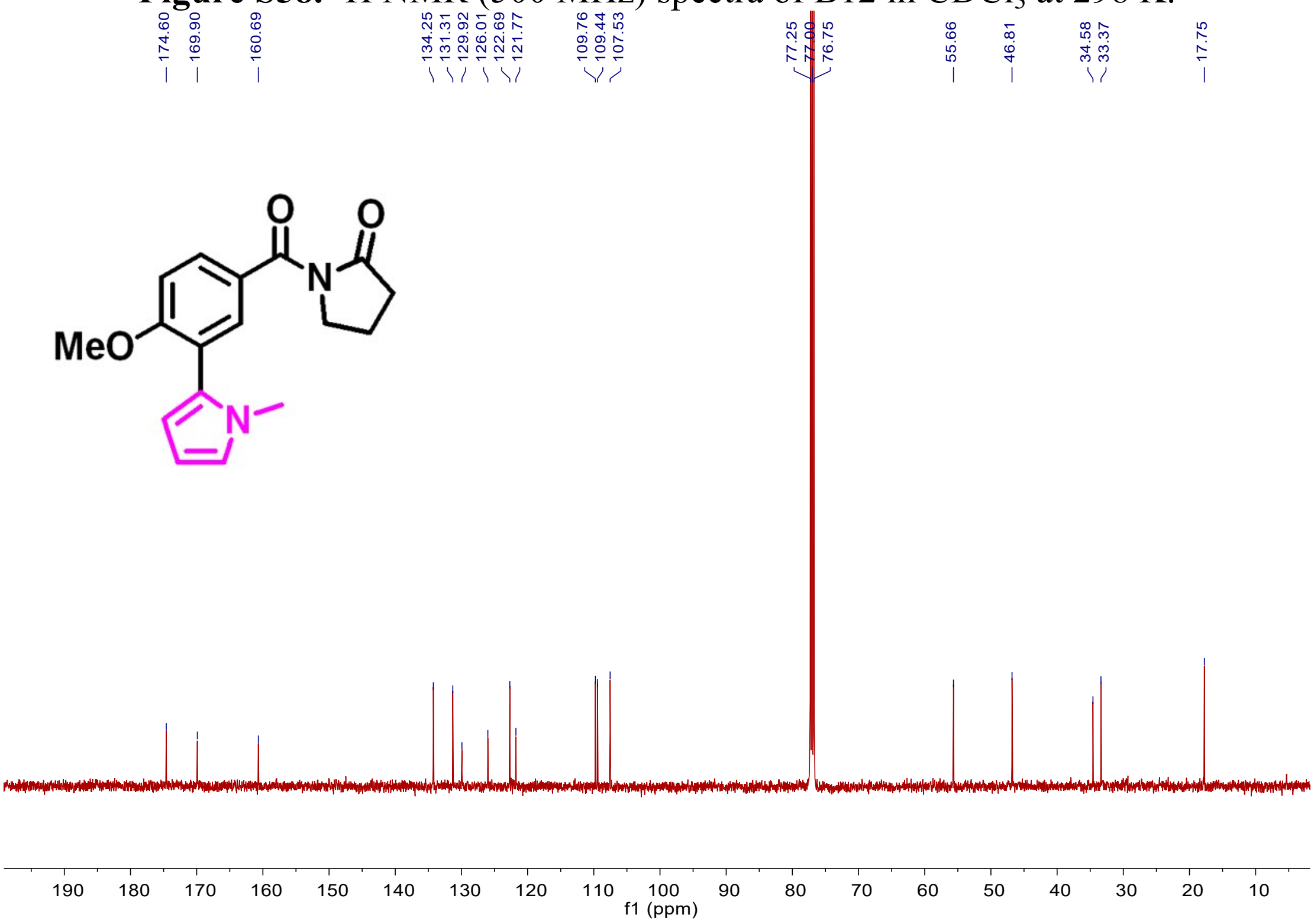

Figure S59. ^{13}C NMR (126 MHz) spectra of B12 in $CDCl_3$ at 298 K.

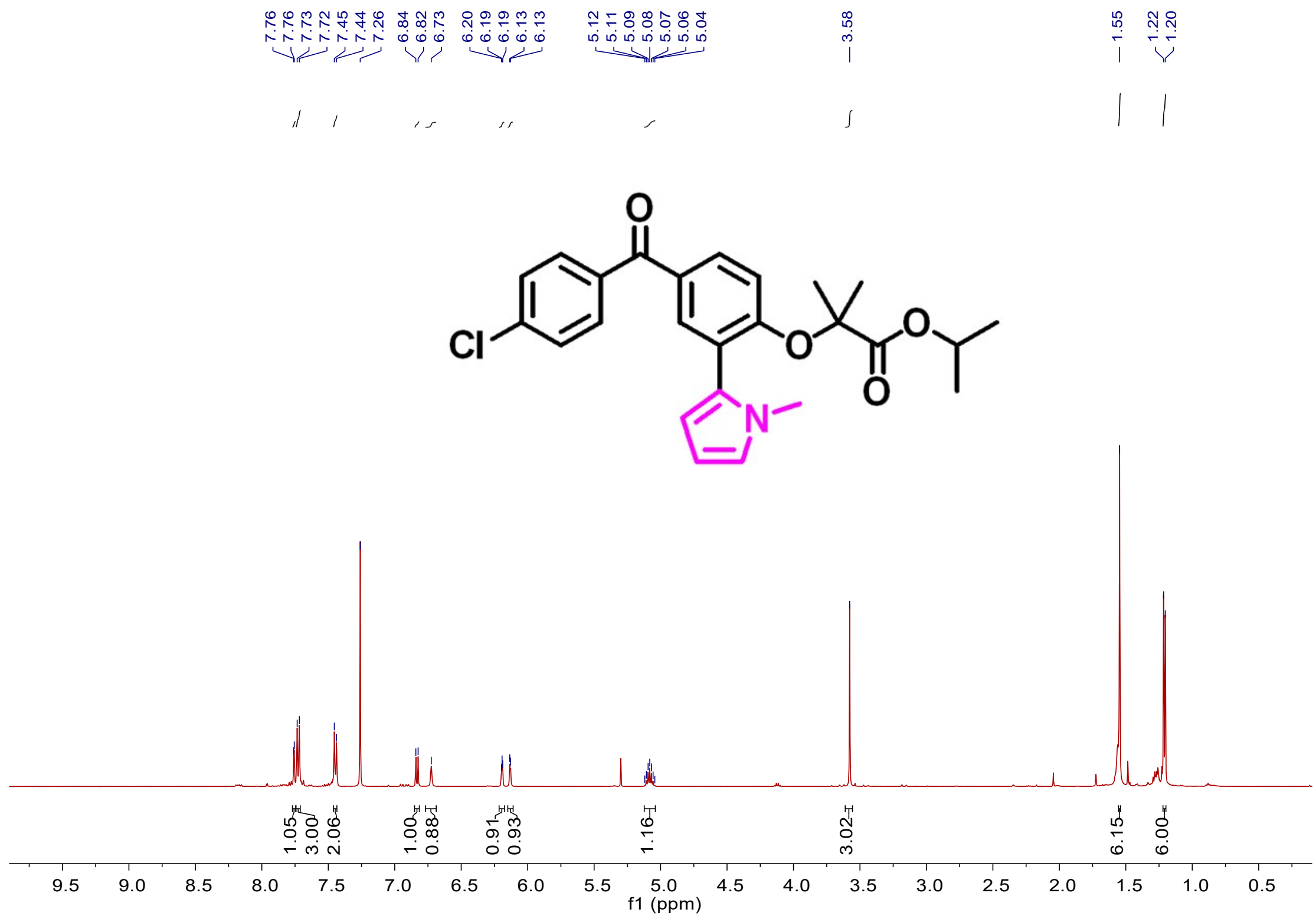

Figure S60. ^{1}H NMR (500 MHz) spectra of B13 in $CDCl_3$ at 298 K.

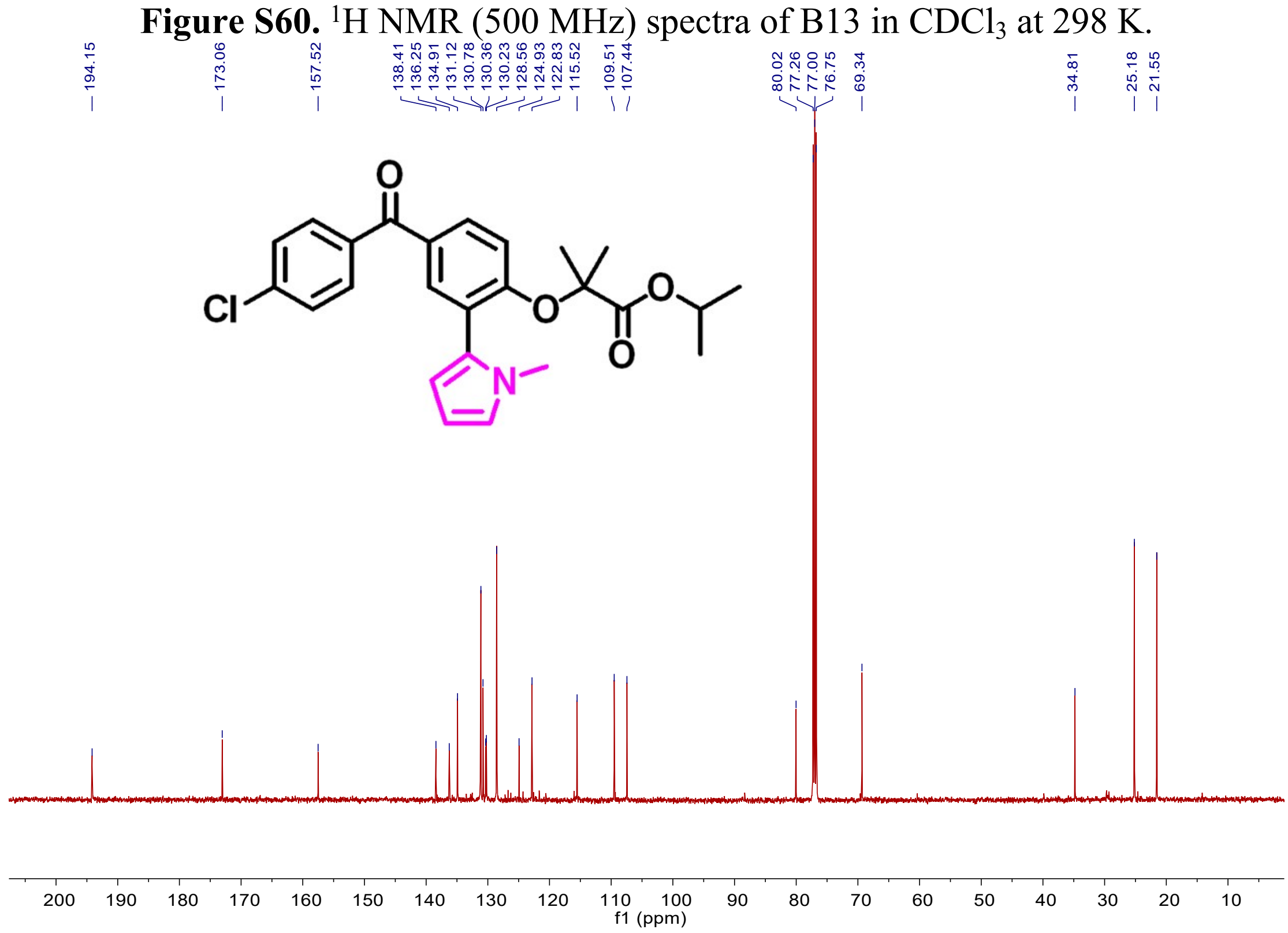

Figure S61. ^{13}C NMR (126 MHz) spectra of B13 in $CDCl_3$ at 298 K.

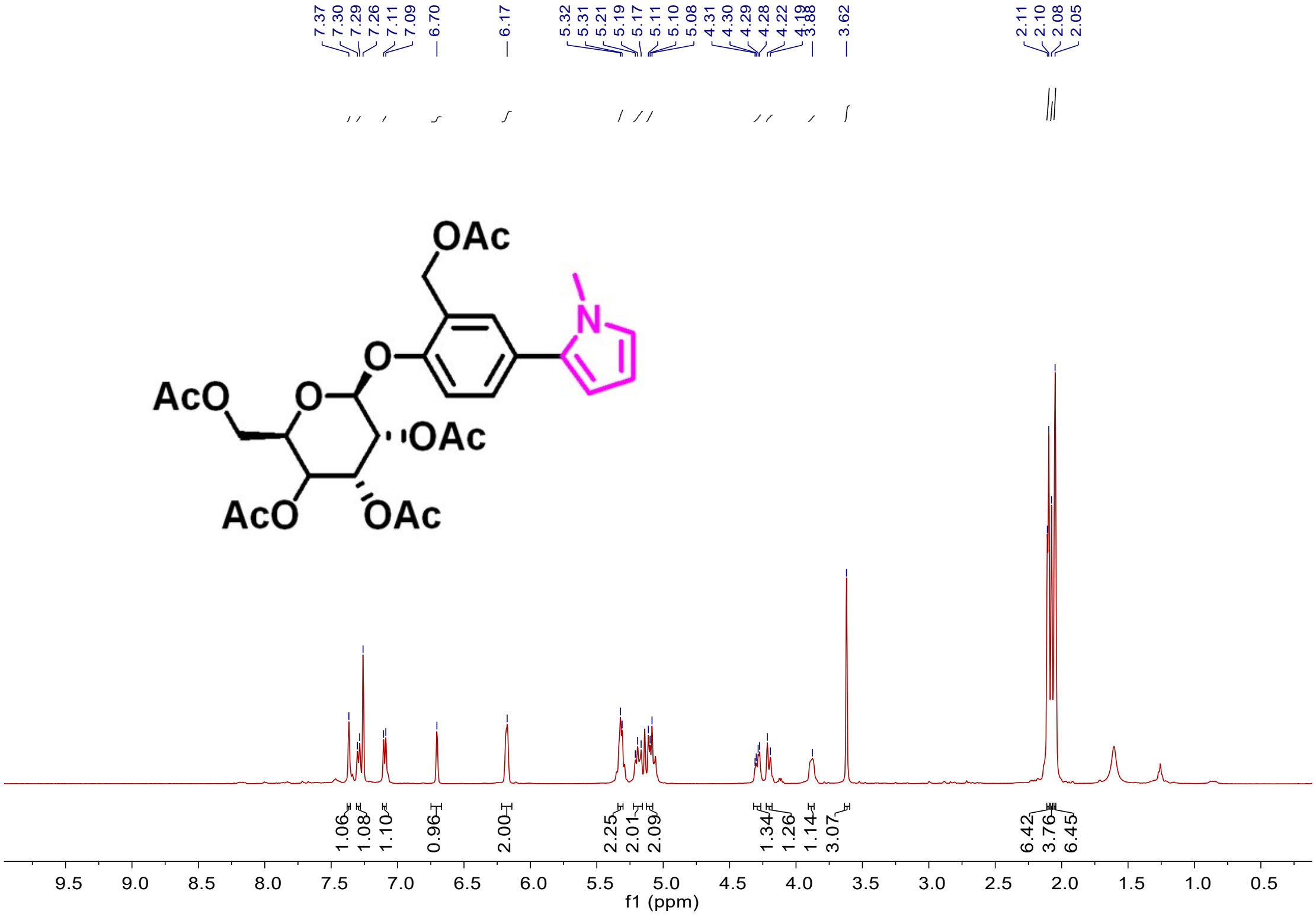

Figure S62. ^{1}H NMR (500 MHz) spectra of B14 in $CDCl_3$ at 298 K.

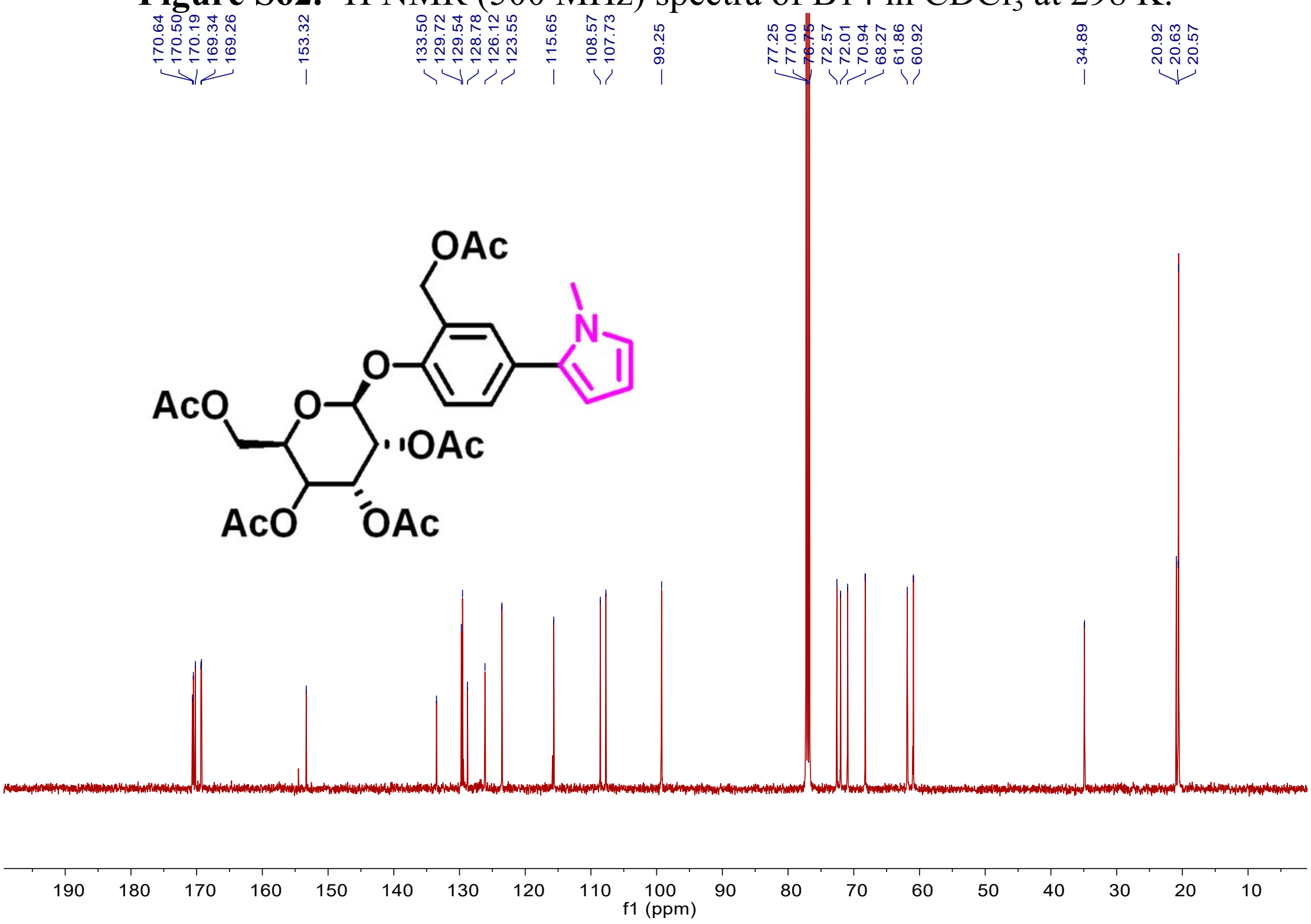

Figure S63. ^{13}C NMR (126 MHz) spectra of B14 in $CDCl_3$ at 298 K.

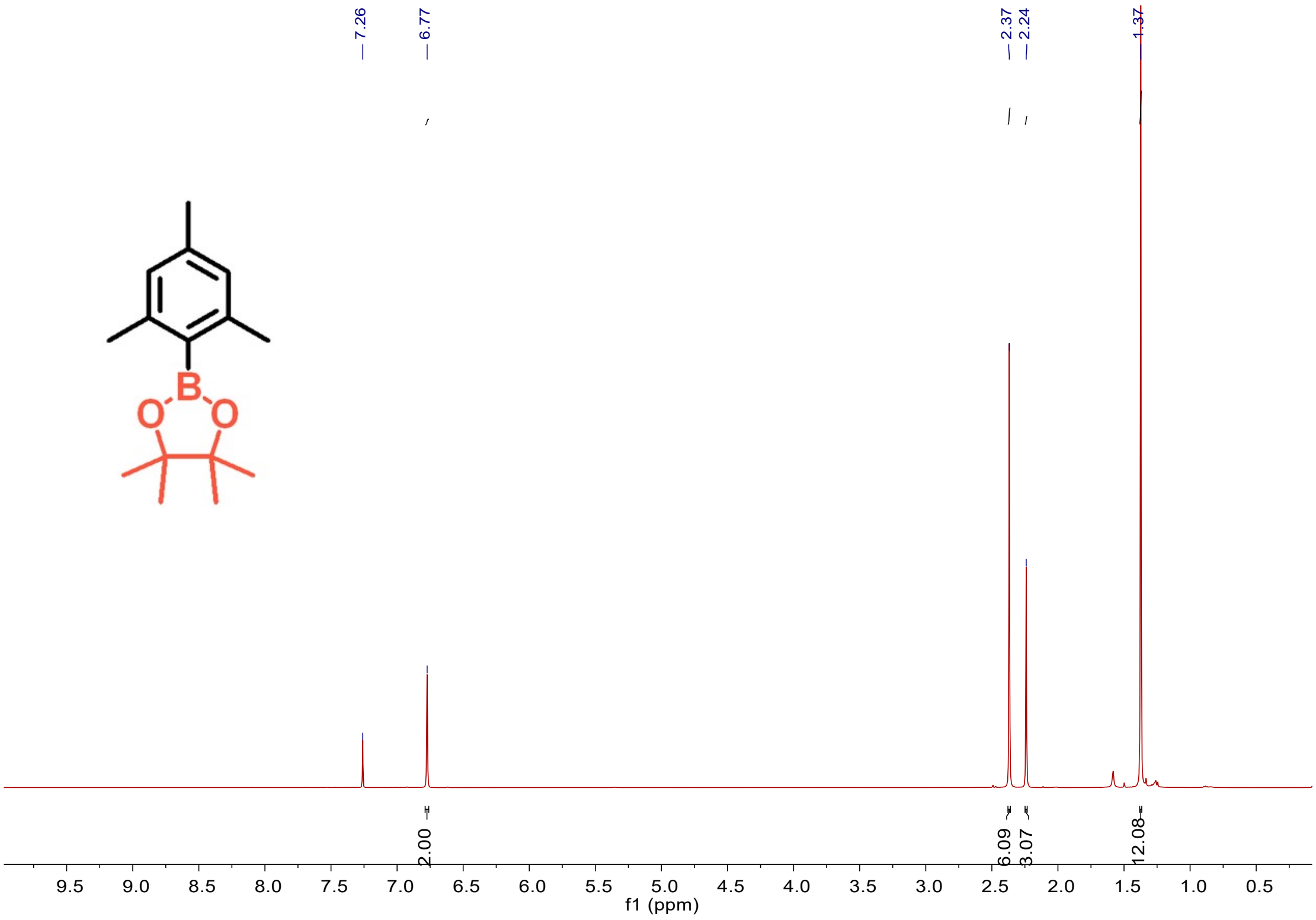

Figure S64. ^{1}H NMR (500 MHz) spectra of C1 in $CDCl_3$ at 298 K.

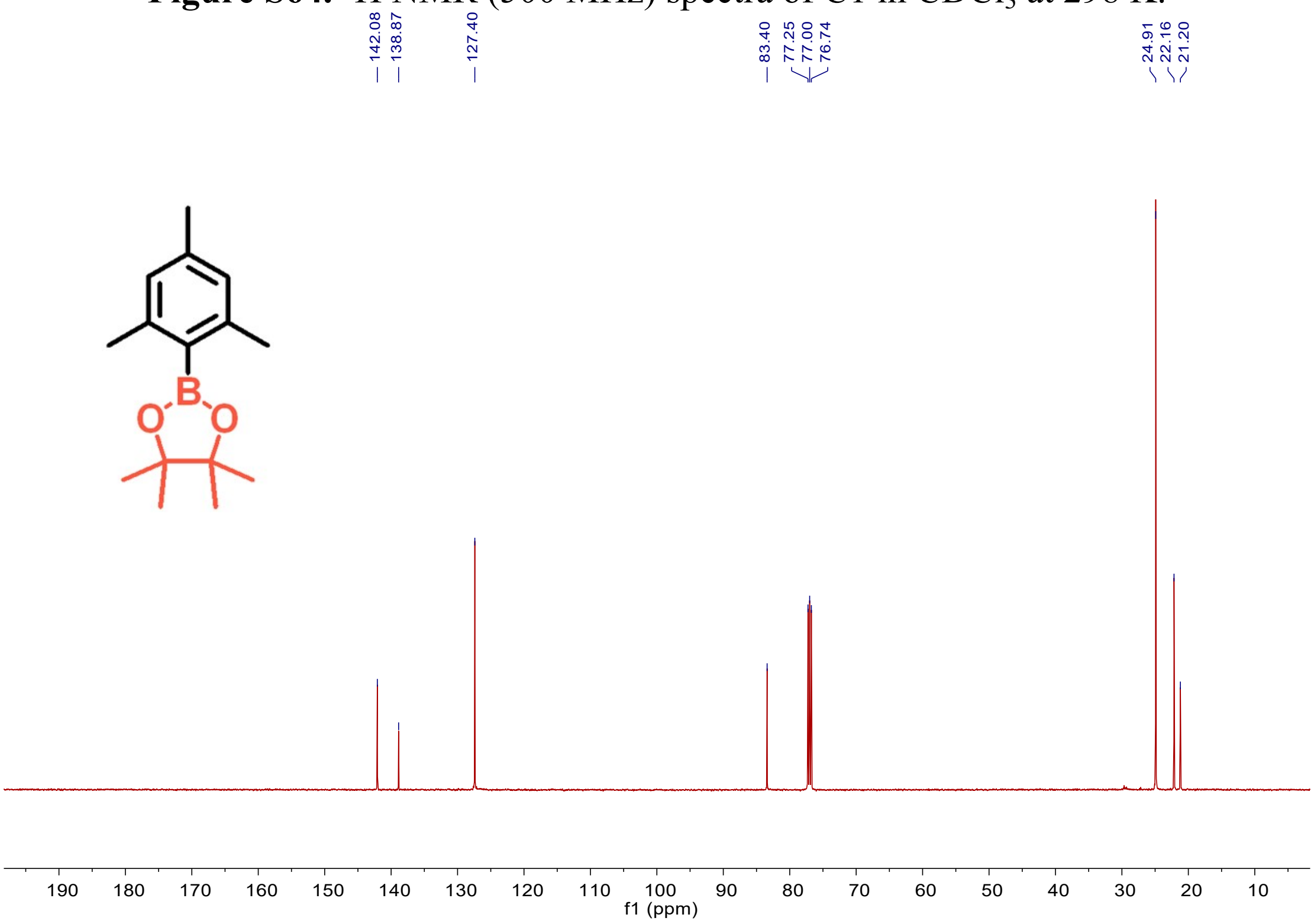

Figure S65. ^{13}C NMR (126 MHz) spectra of C1 in $CDCl_3$ at 298 K.

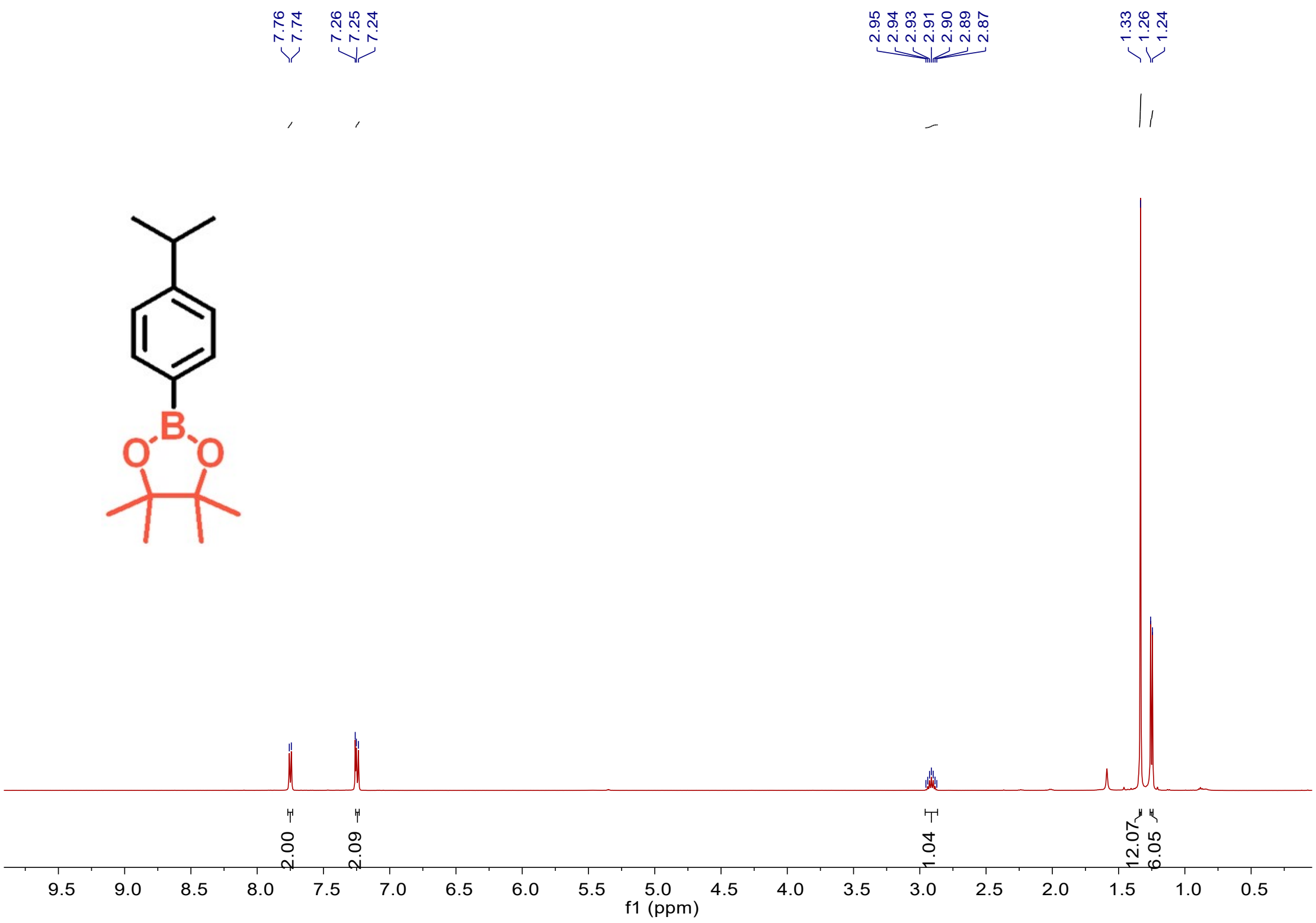

Figure S66. ^{1}H NMR (500 MHz) spectra of C2 in $CDCl_3$ at 298 K.

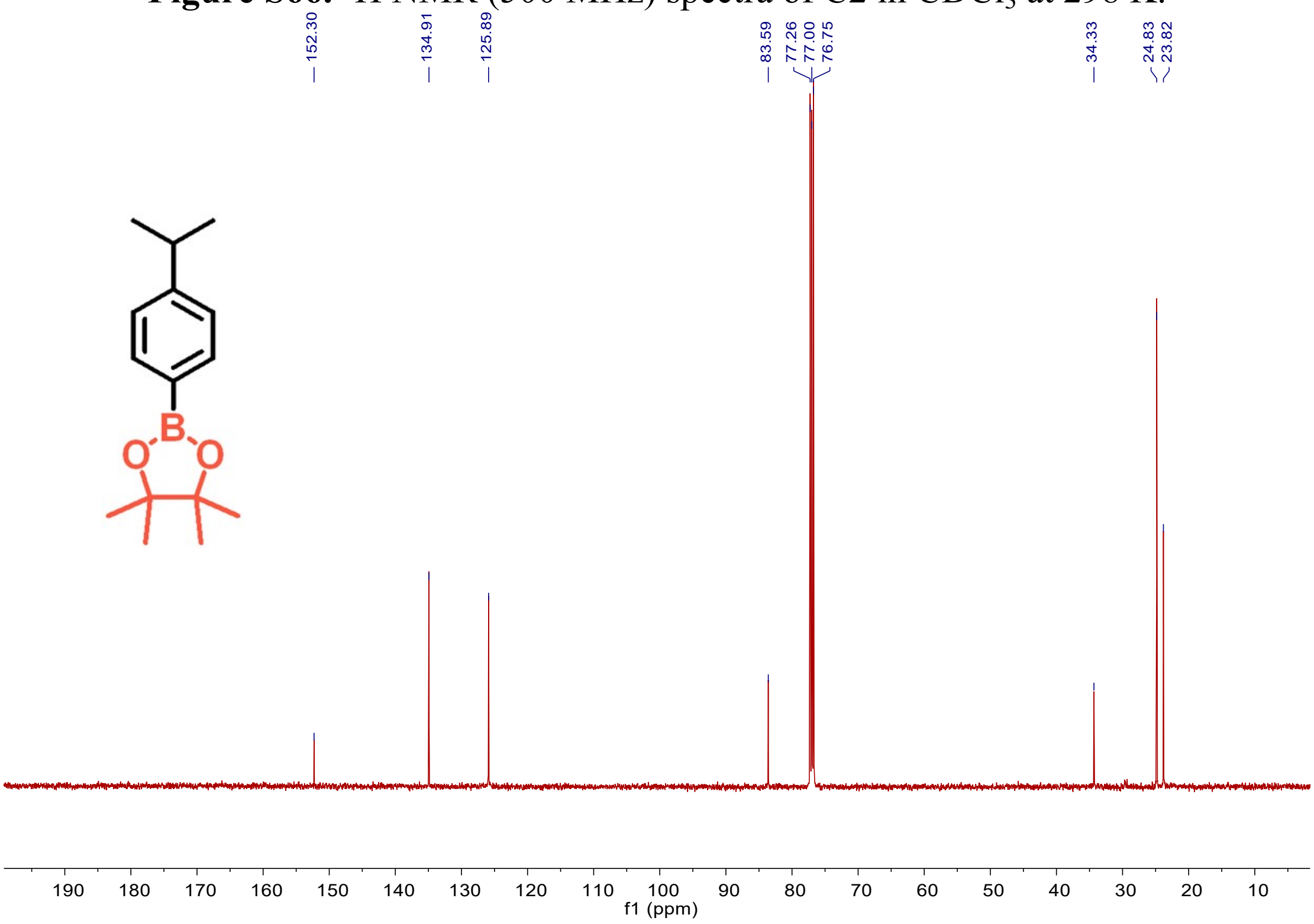

Figure S67. ^{13}C NMR (126 MHz) spectra of C2 in $CDCl_3$ at 298 K.

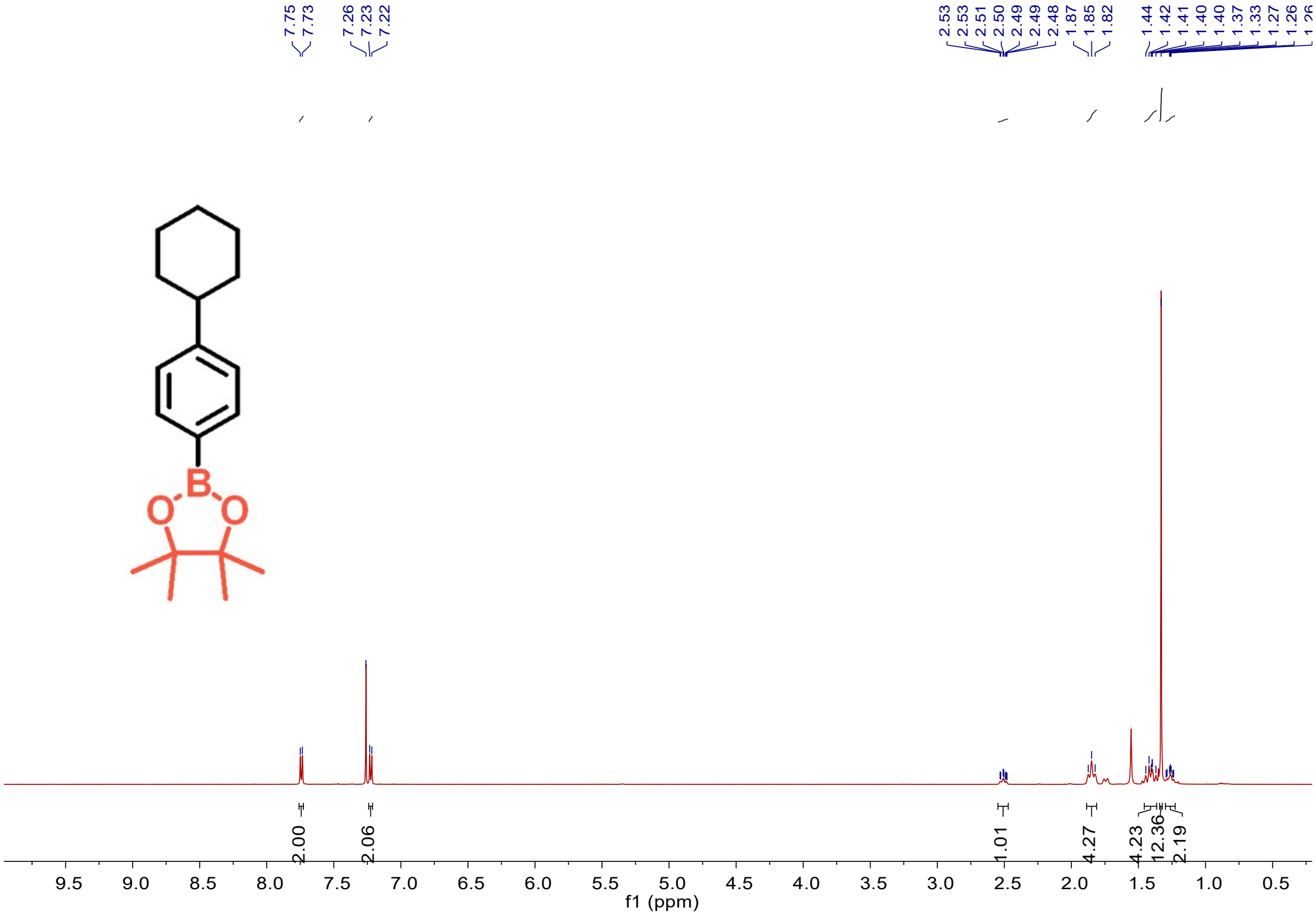

Figure S68. ^{1}H NMR (500 MHz) spectra of C3 in $CDCl_3$ at 298 K.

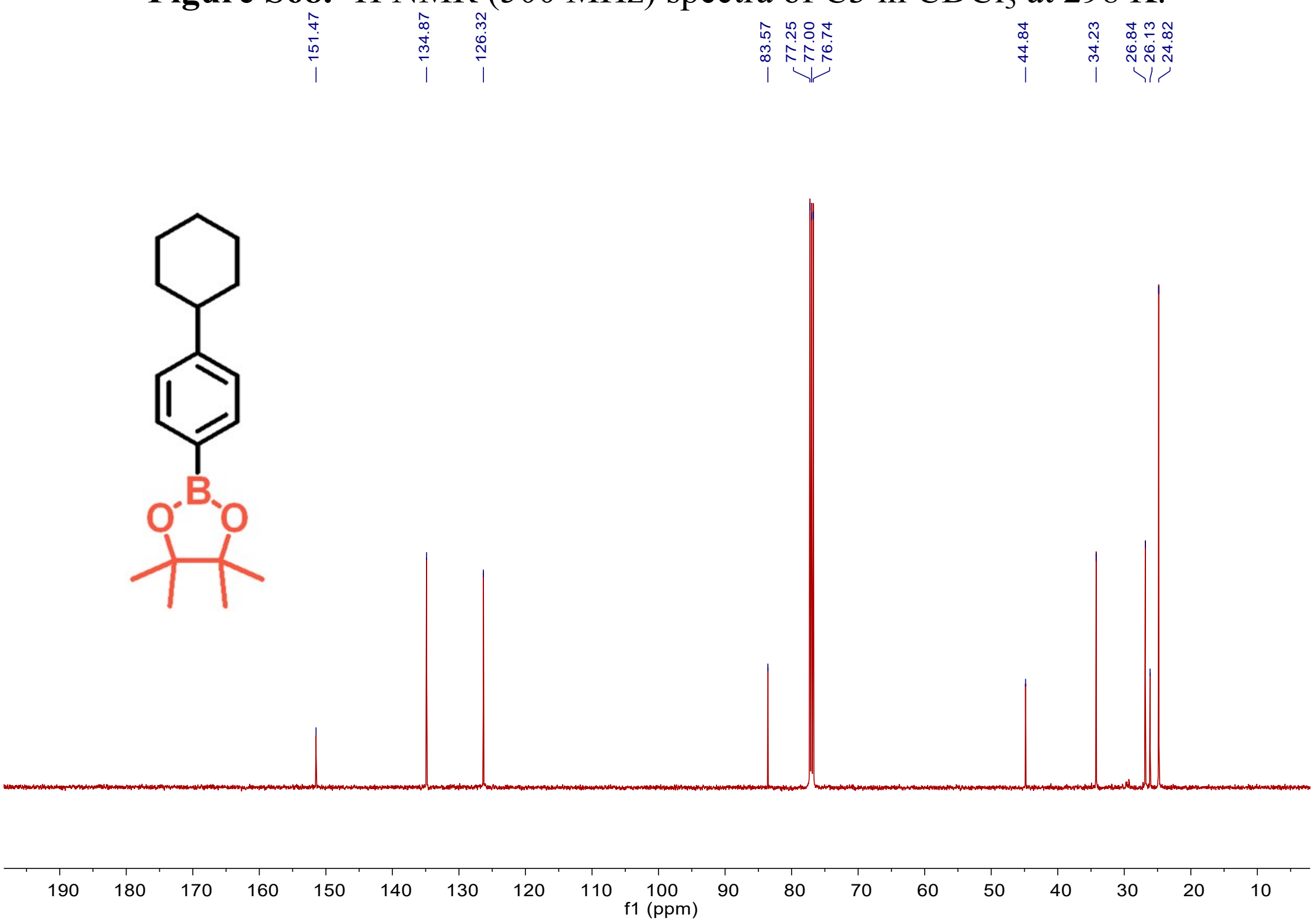

Figure S69. ^{13}C NMR (126 MHz) spectra of C3 in $CDCl_3$ at 298 K.

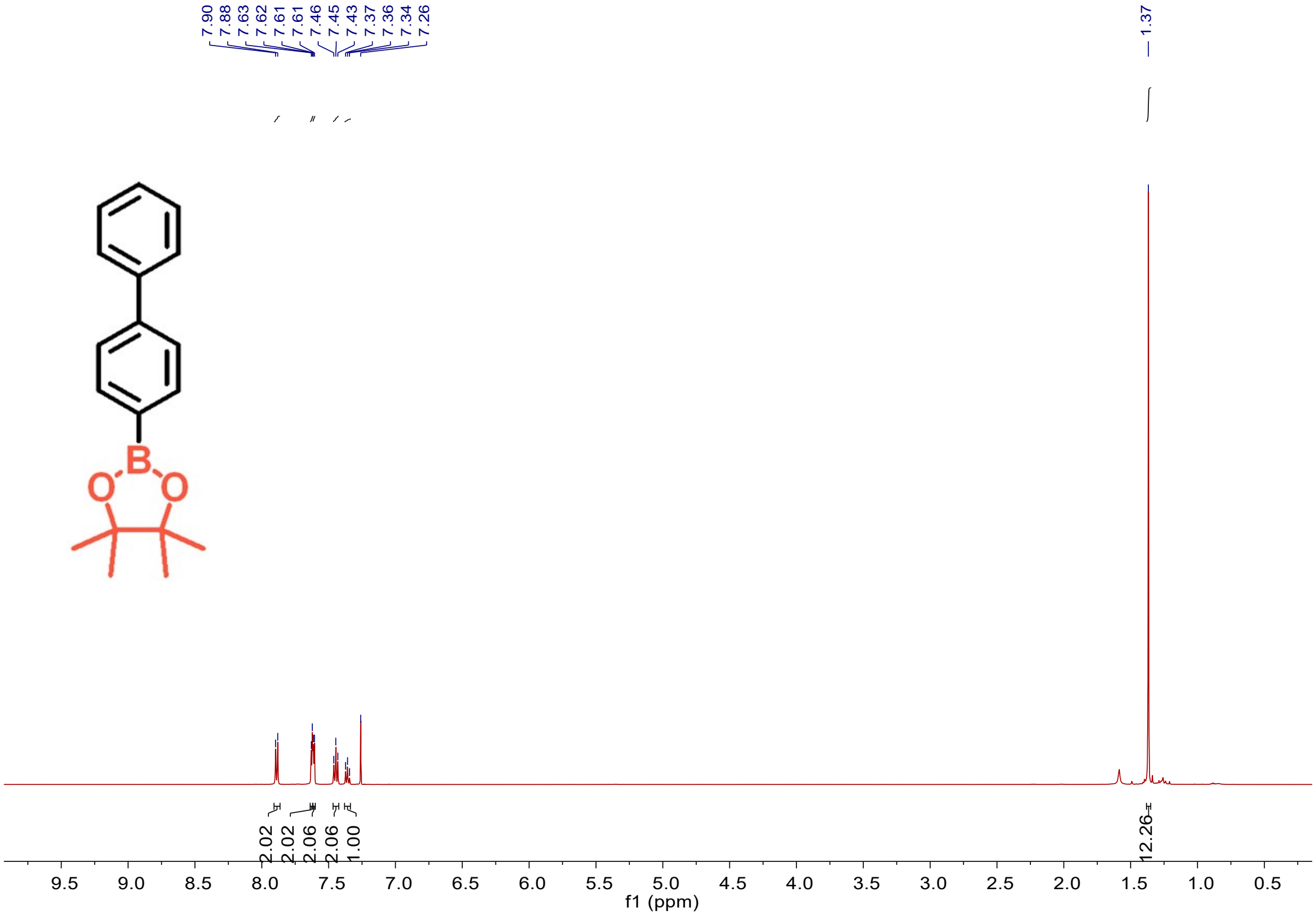

Figure S70. ^{1}H NMR (500 MHz) spectra of C4 in $CDCl_3$ at 298 K.

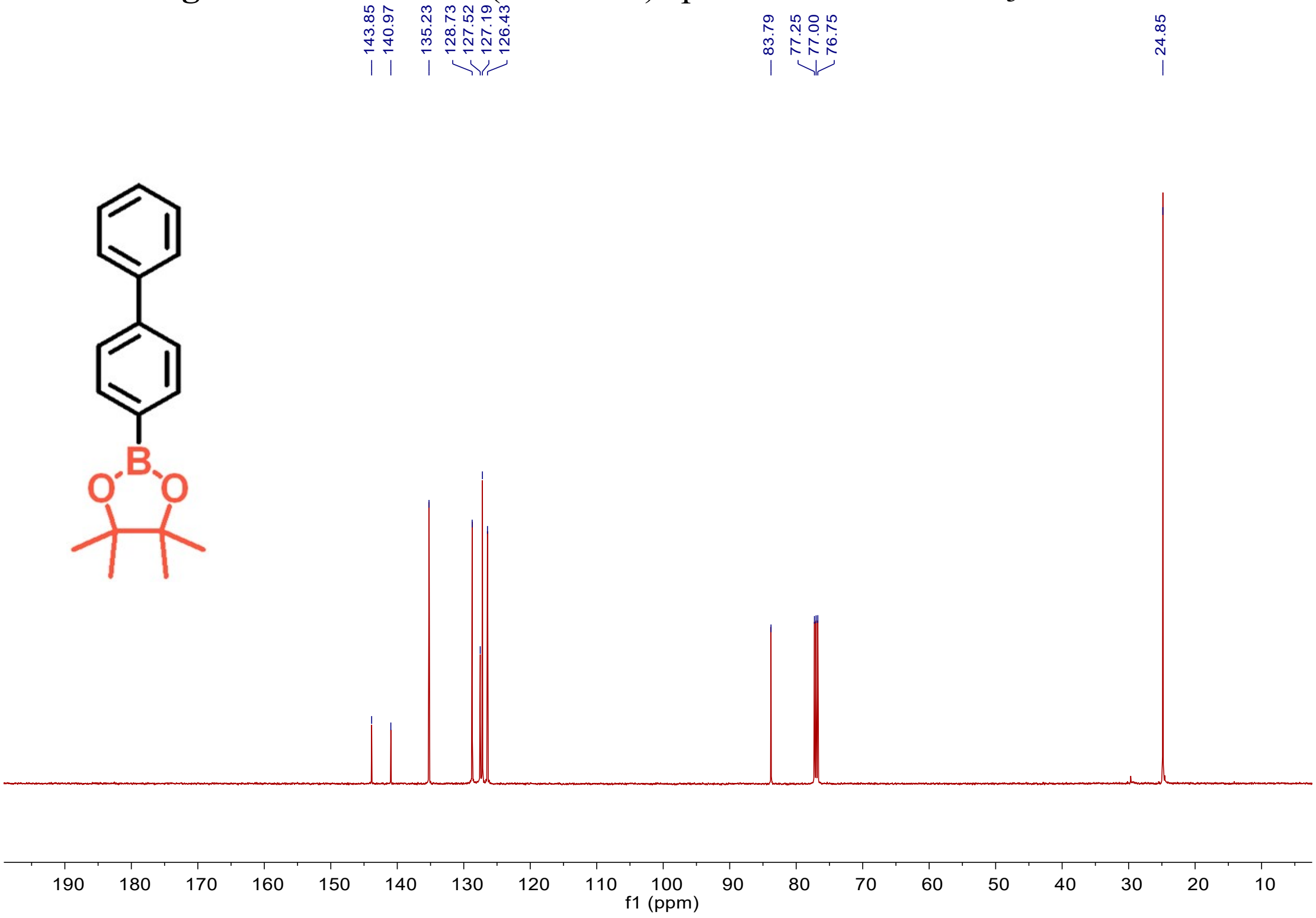

Figure S71. ^{13}C NMR (126 MHz) spectra of C4 in $CDCl_3$ at 298 K.

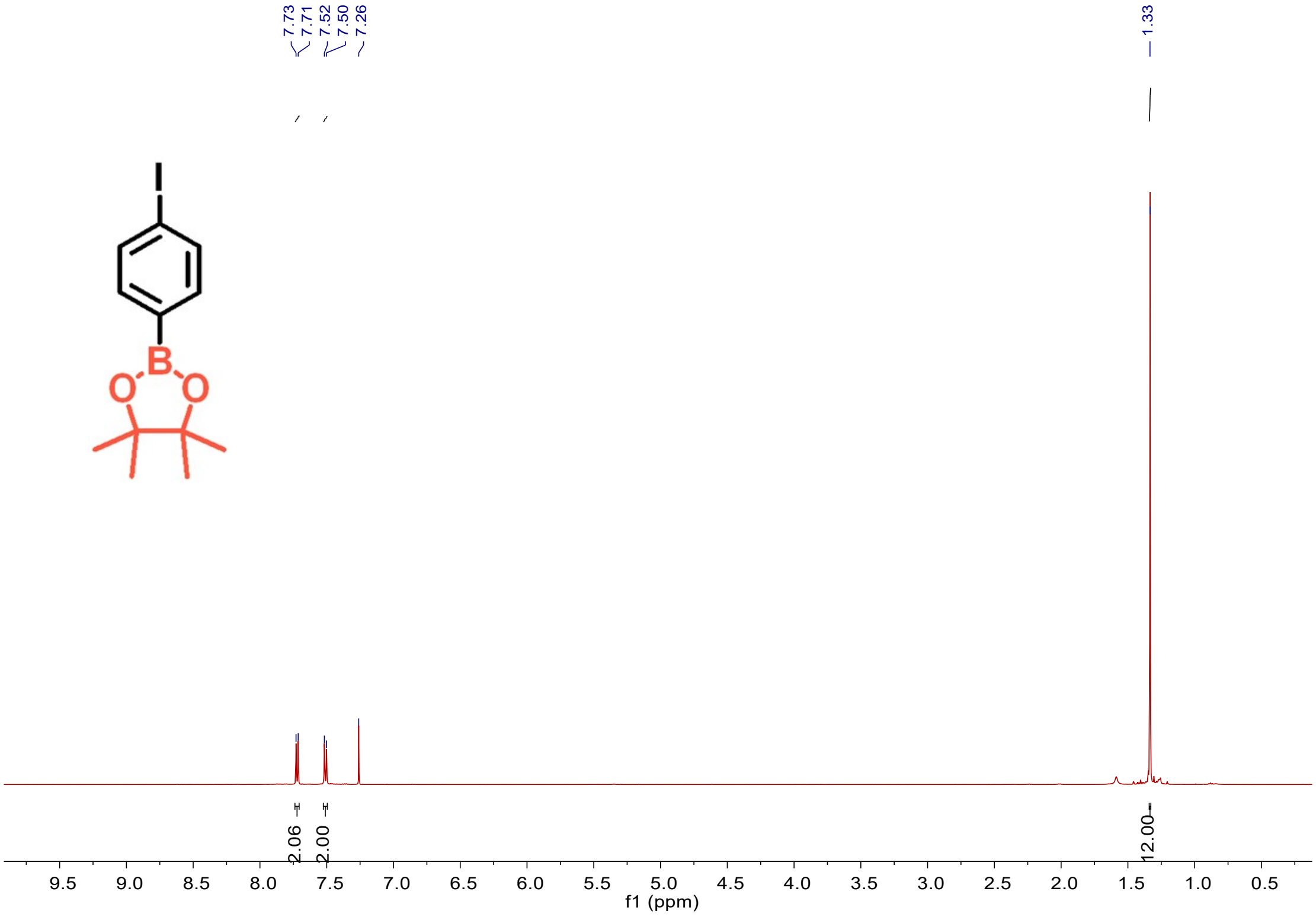

Figure S72. ^{1}H NMR (500 MHz) spectra of C5 in $CDCl_3$ at 298 K.

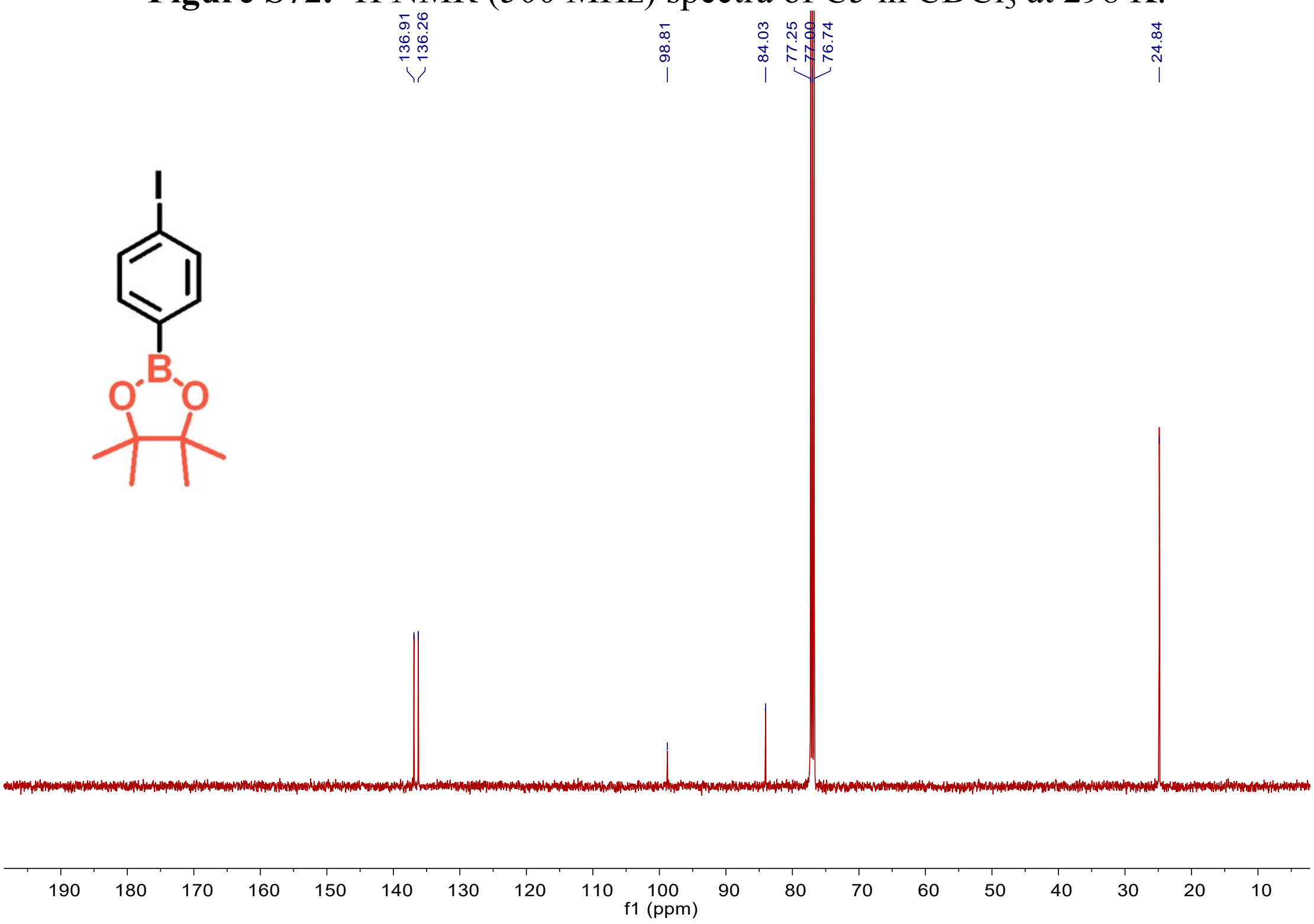

Figure S73. ^{13}C NMR (126 MHz) spectra of C5 in $CDCl_3$ at 298 K.

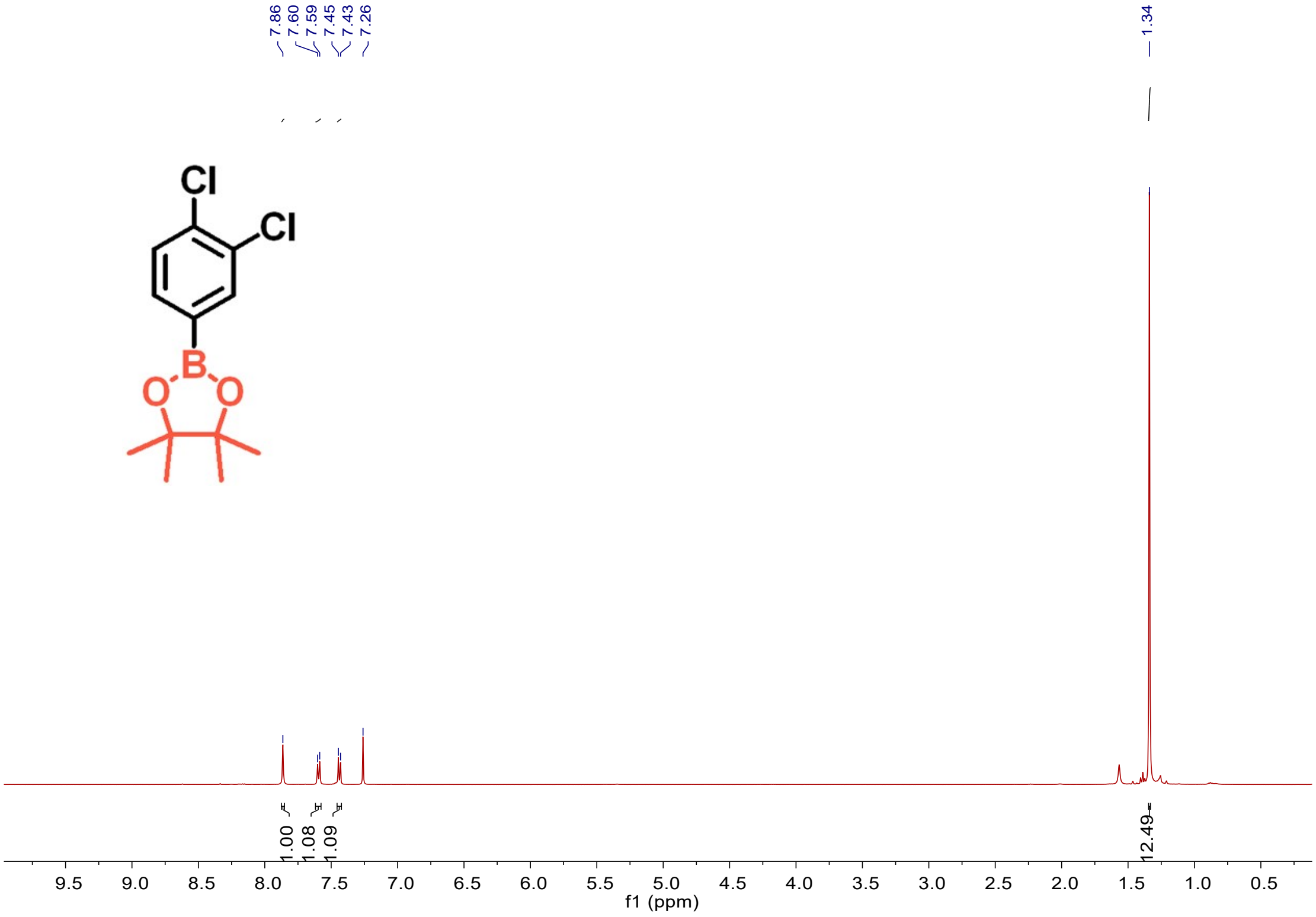

Figure S74. ^{1}H NMR (500 MHz) spectra of C6 in $CDCl_3$ at 298 K.

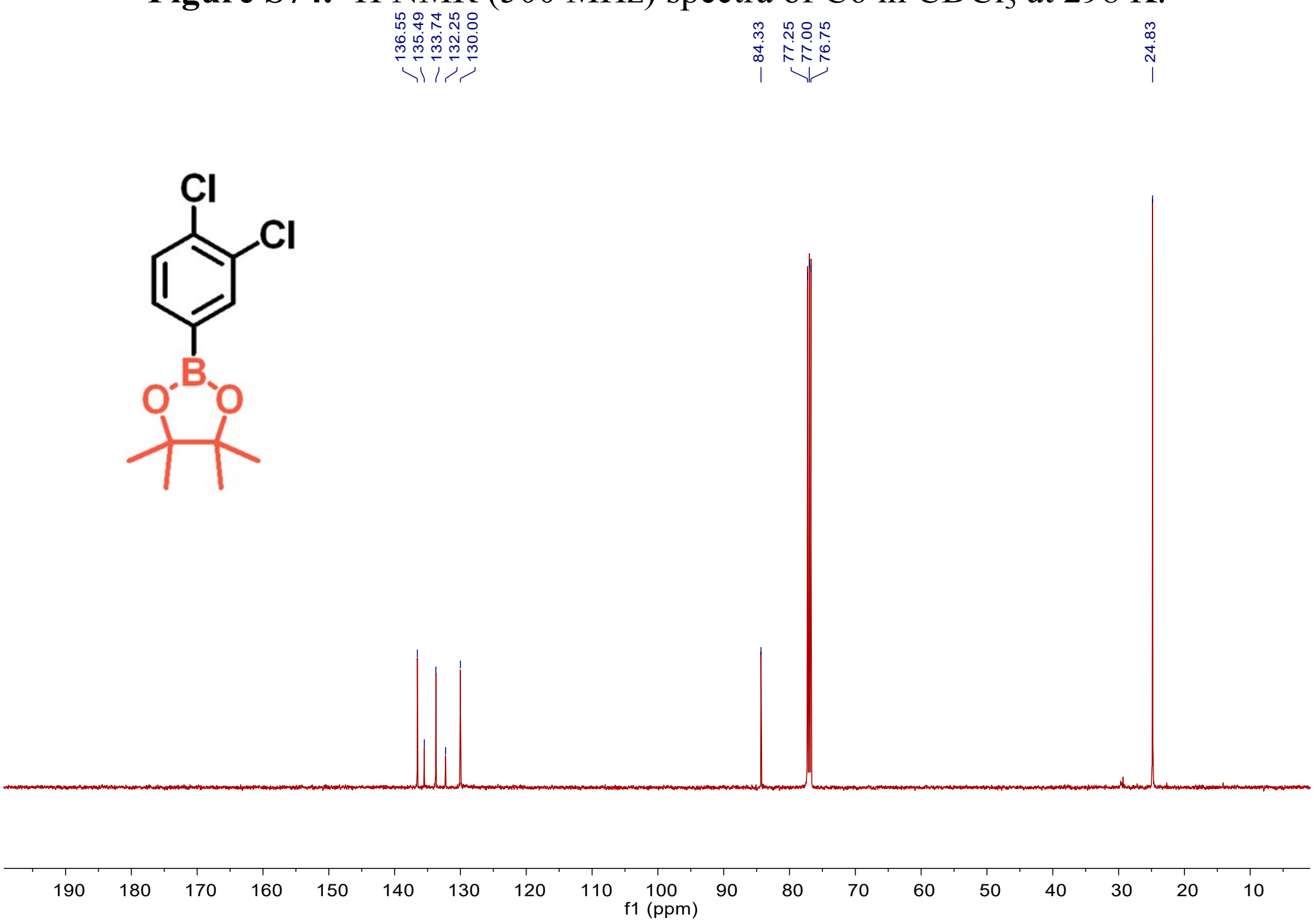

Figure S75. ^{13}C NMR (126 MHz) spectra of C6 in $CDCl_3$ at 298 K.

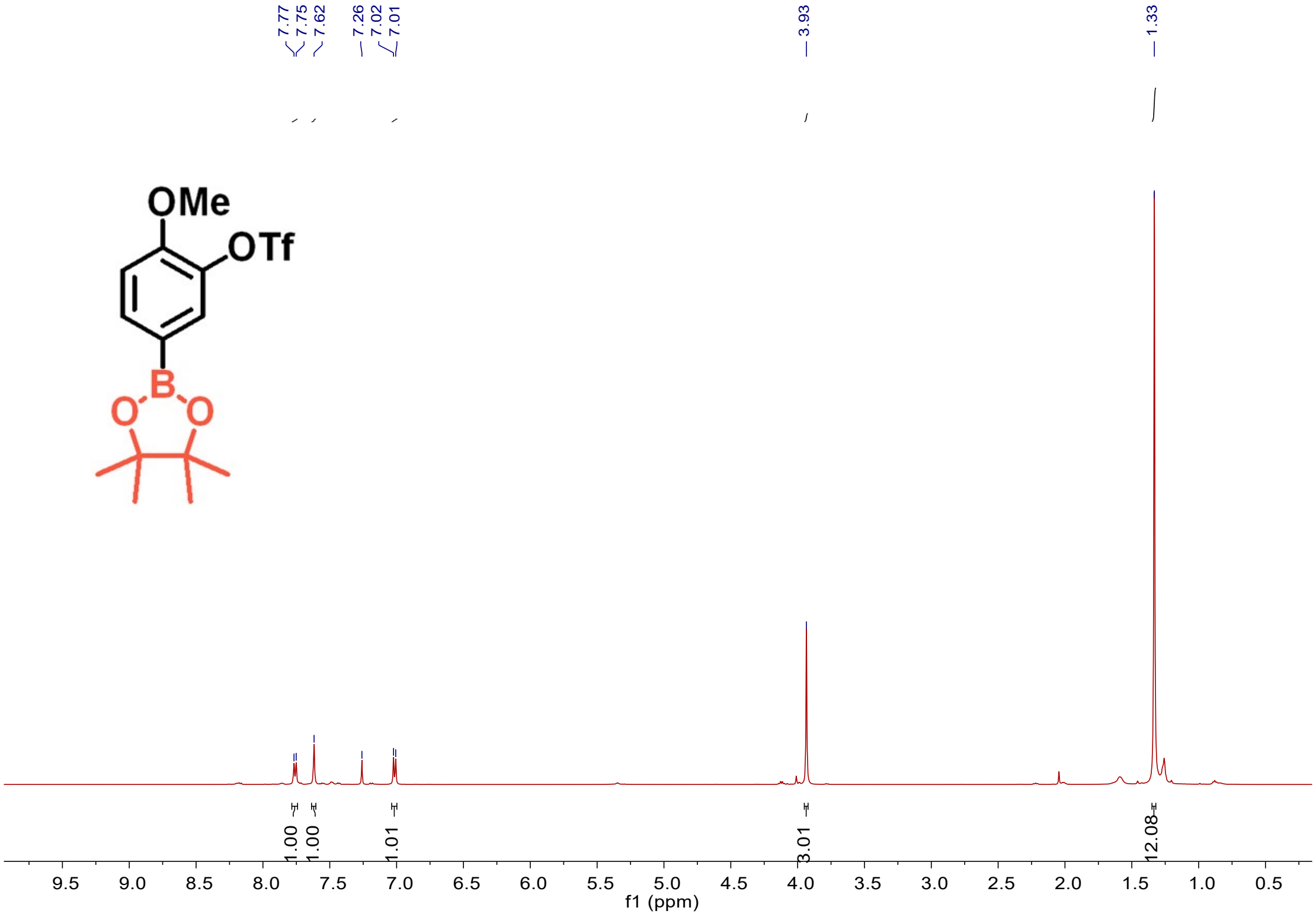

Figure S76. ^{1}H NMR (500 MHz) spectra of C7 in $CDCl_3$ at 298 K.

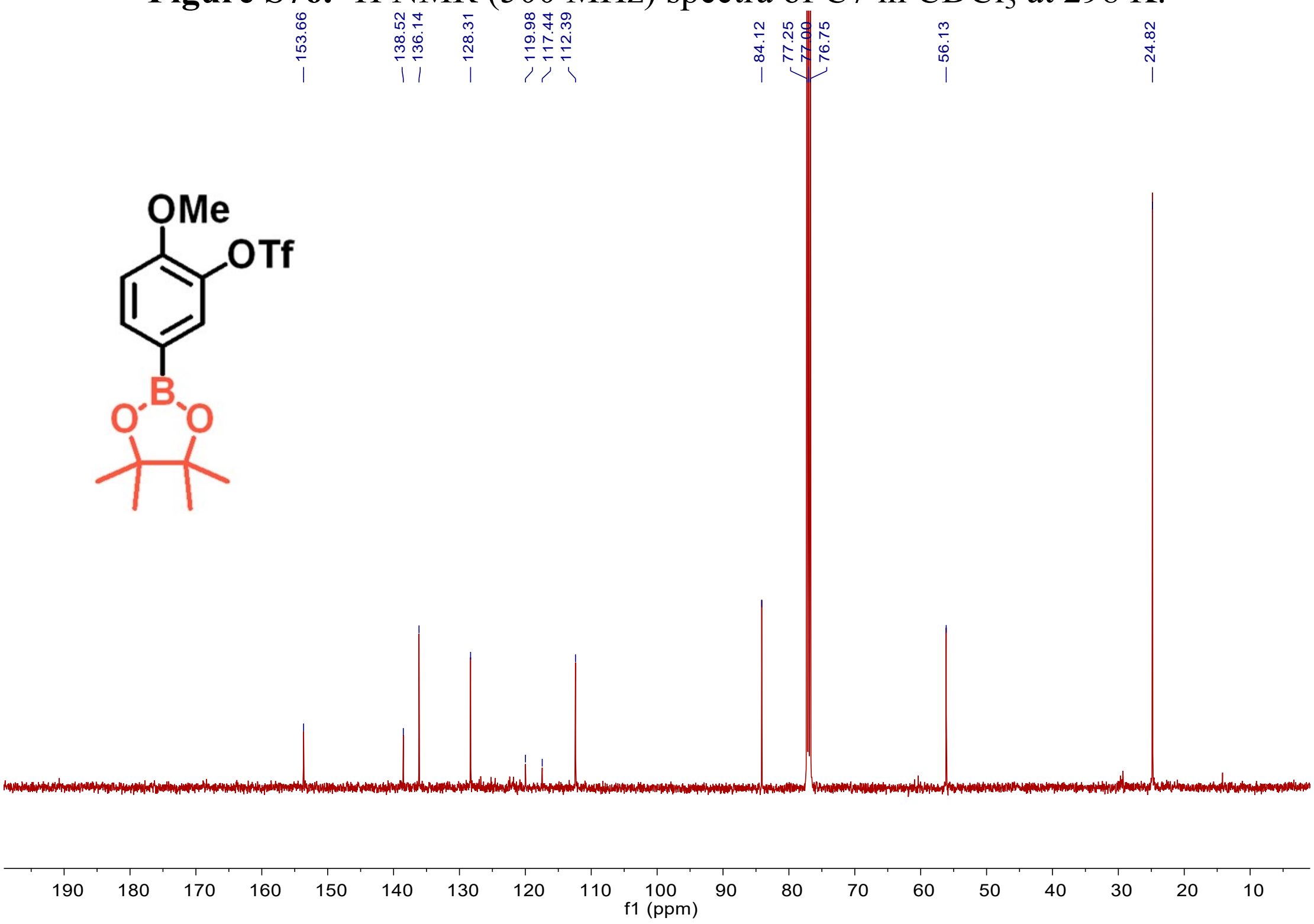

Figure S77. ^{13}C NMR (126 MHz) spectra of C7 in $CDCl_3$ at 298 K.

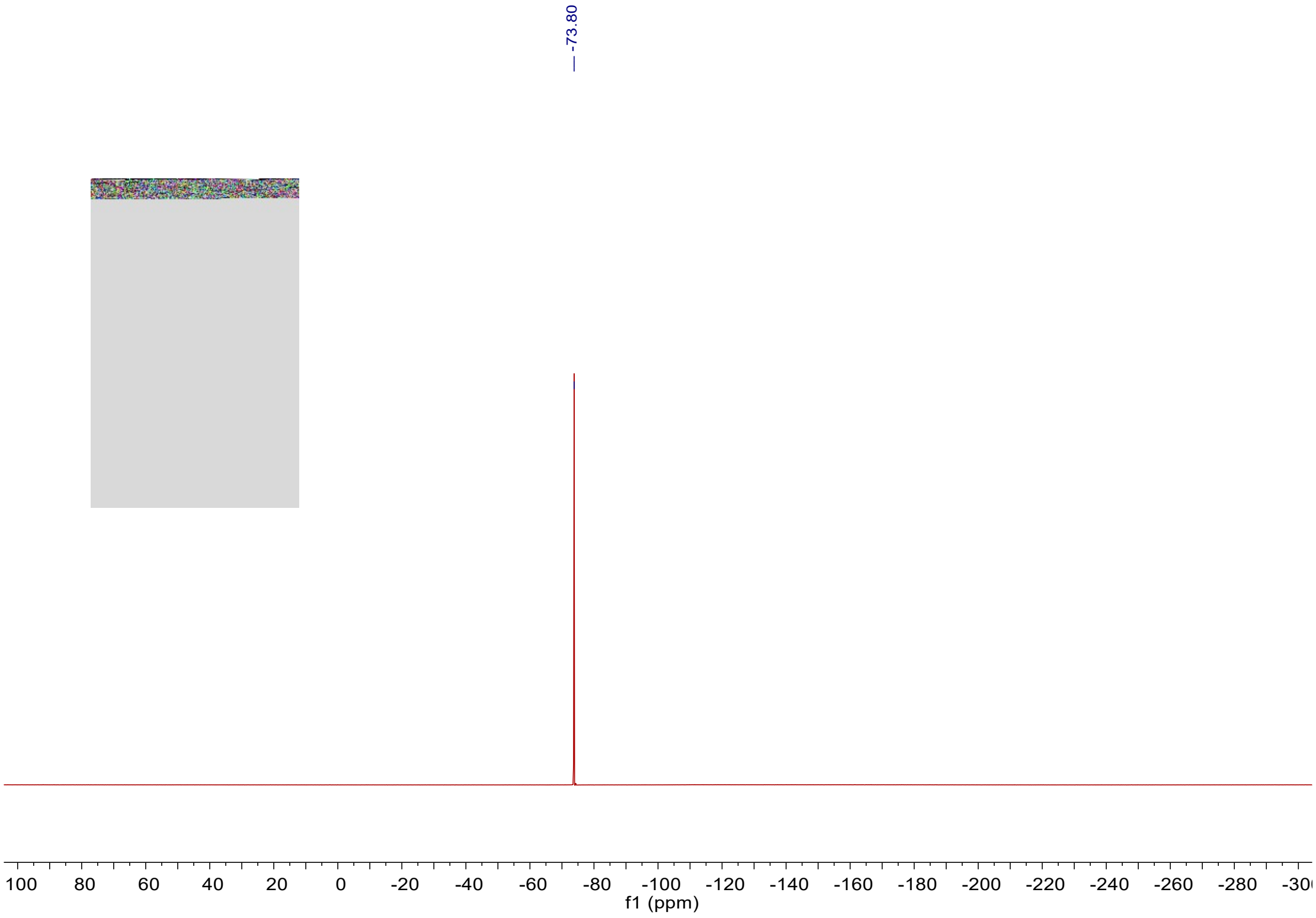

Figure S78. ^{19}F NMR (471 MHz) spectra of C7 in $CDCl_3$ at 298 K.

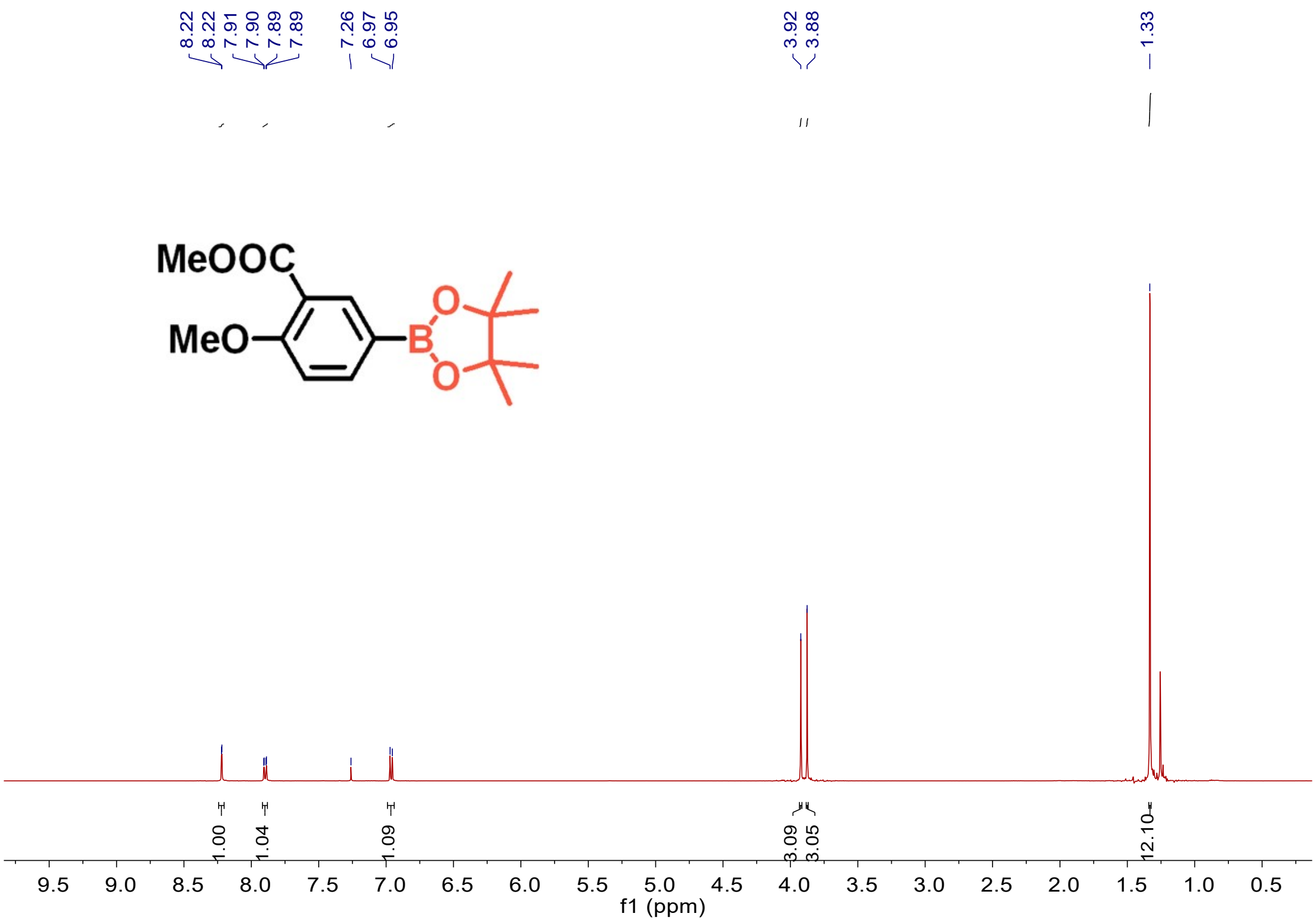

Figure S79. ^{1}H NMR (500 MHz) spectra of C8 in $CDCl_3$ at 298 K.

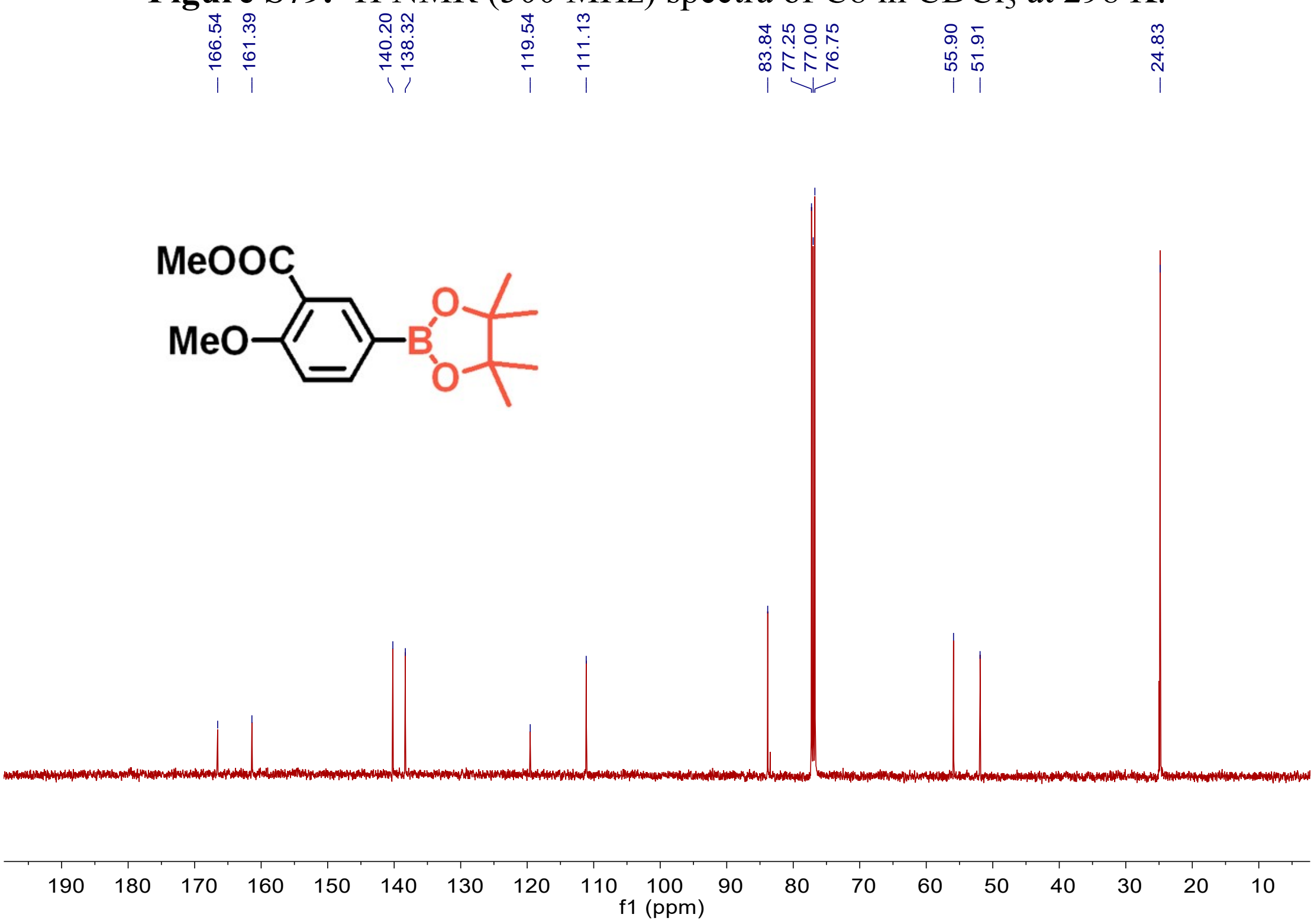

Figure S80. ^{13}C NMR (126 MHz) spectra of C8 in $CDCl_3$ at 298 K.

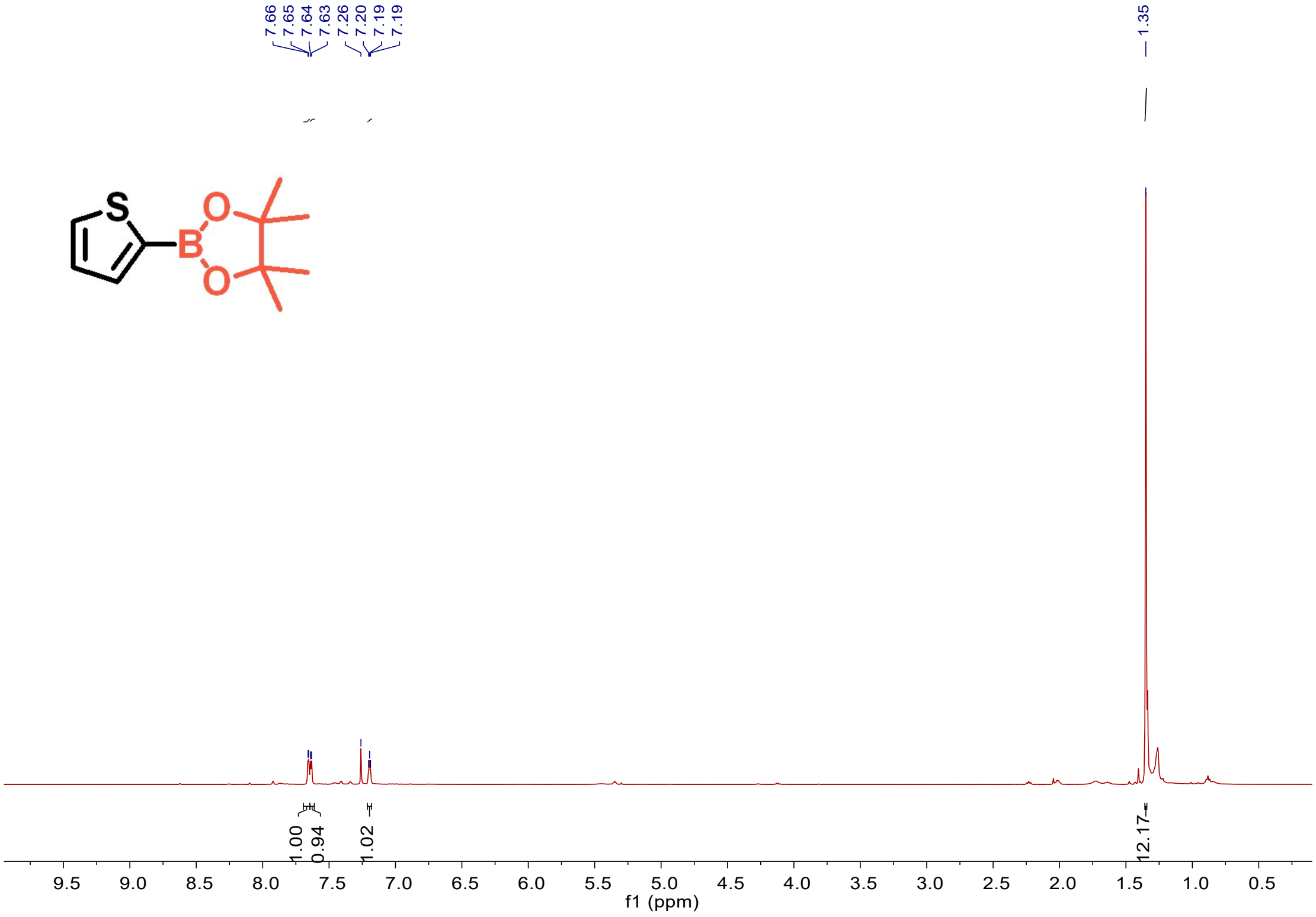

Figure S81. ^{1}H NMR (500 MHz) spectra of C9 in $CDCl_3$ at 298 K.

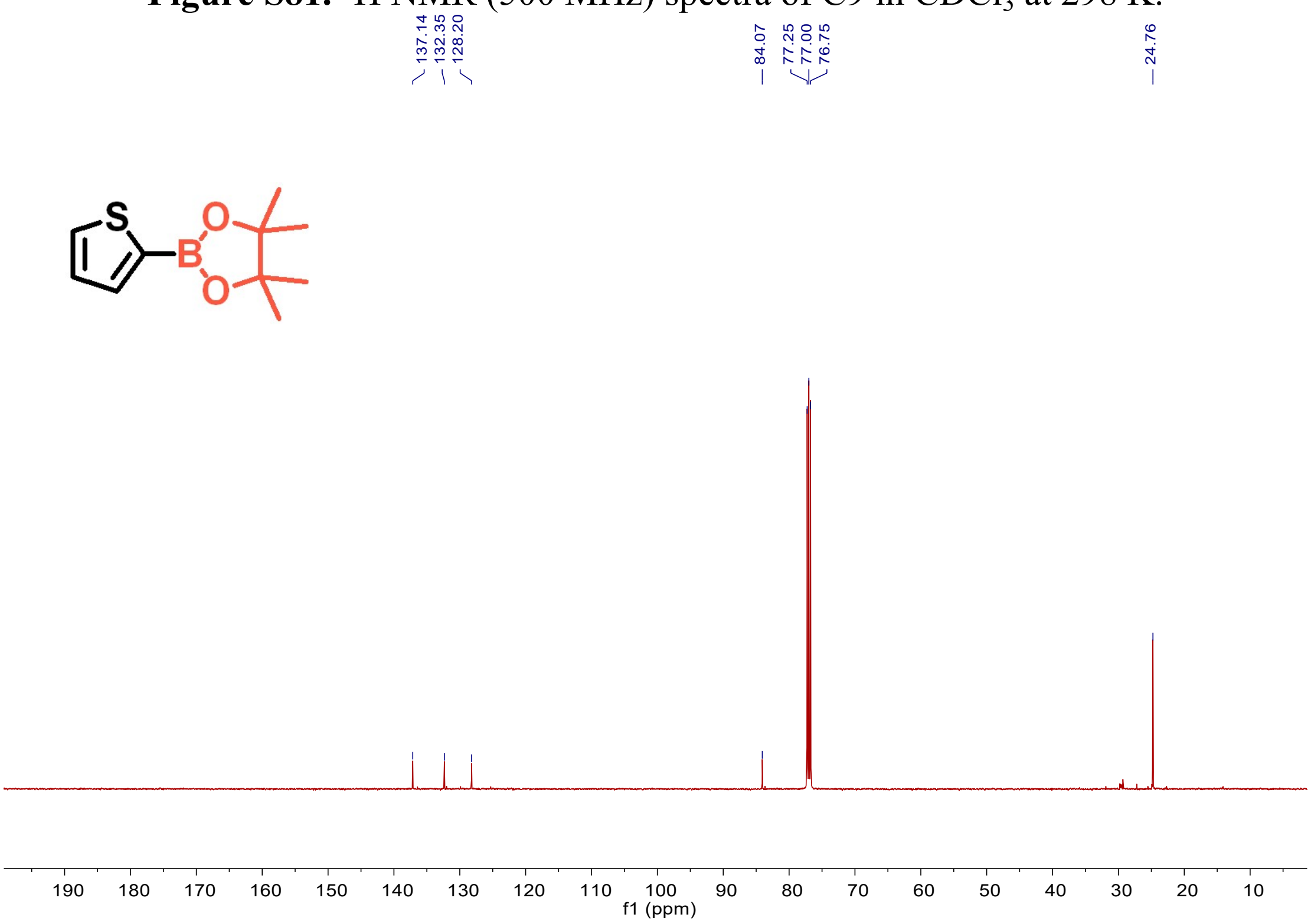

Figure S82. ^{13}C NMR (126 MHz) spectra of C9 in $CDCl_3$ at 298 K.

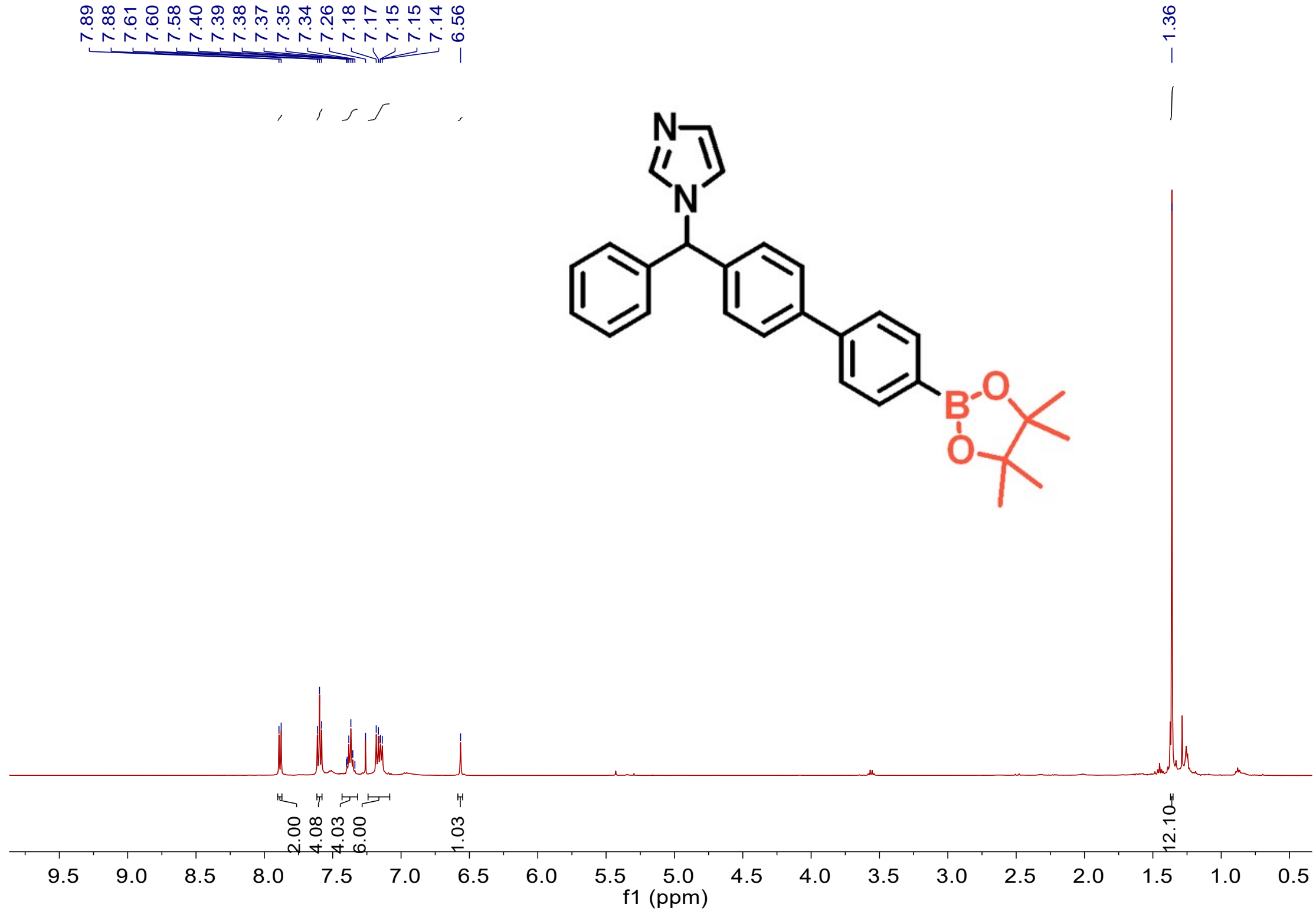

Figure S83. ^{1}H NMR (500 MHz) spectra of C10 in $CDCl_3$ at 298 K.

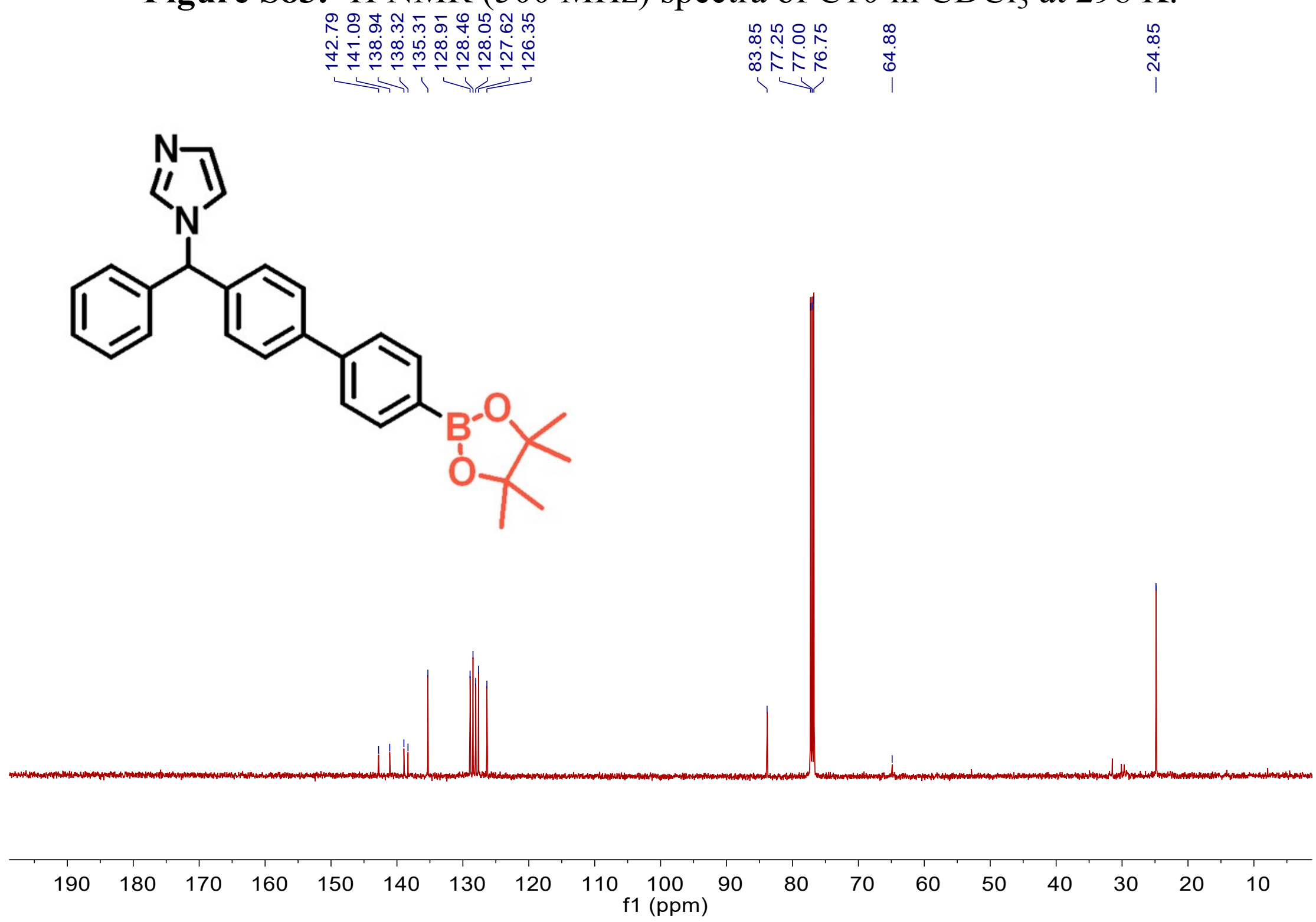

Figure S84. ^{13}C NMR (126 MHz) spectra of C10 in $CDCl_3$ at 298 K.

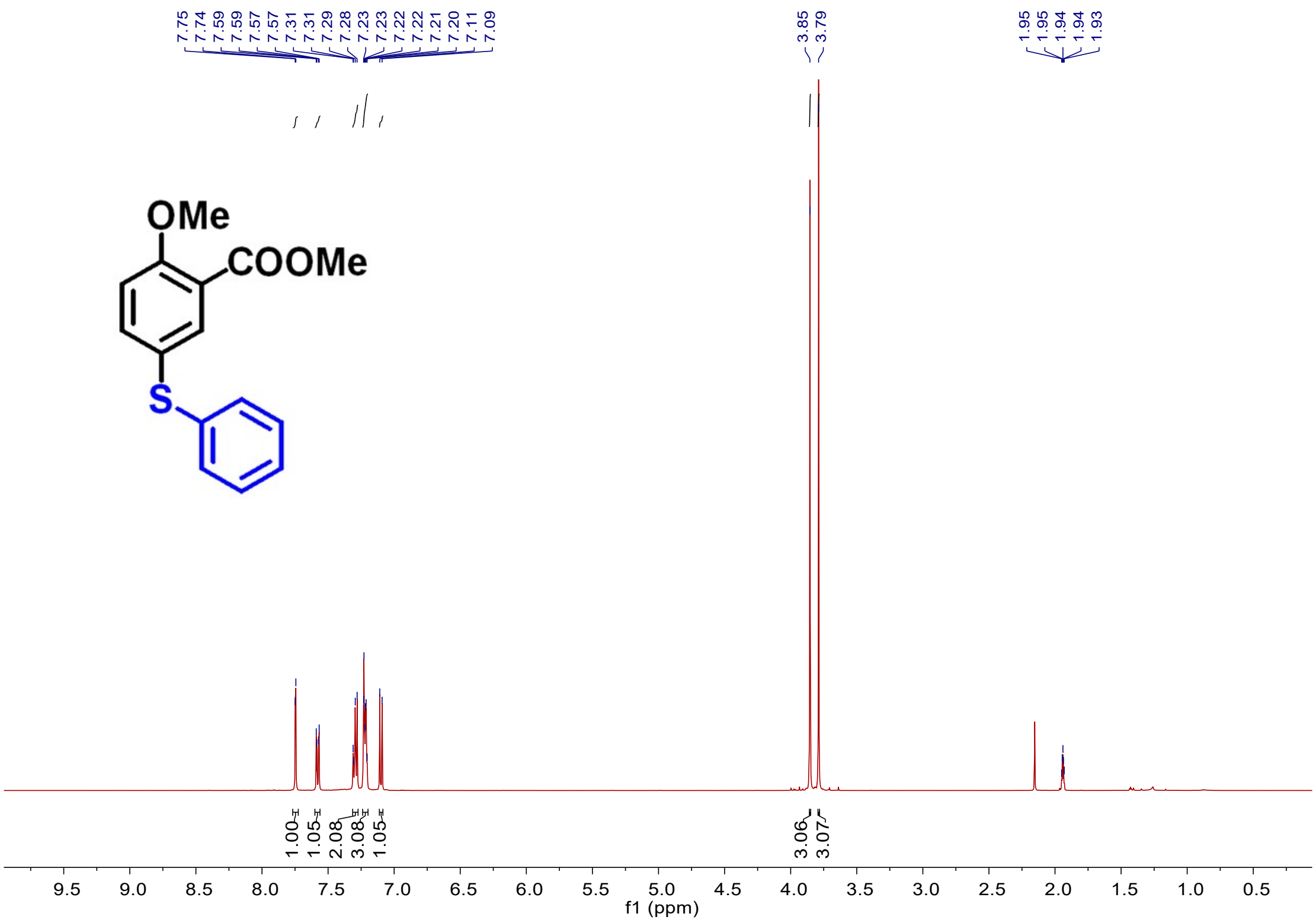

Figure S85. ^{1}H NMR (500 MHz) spectra of D1 in CD_3CN at 298 K.

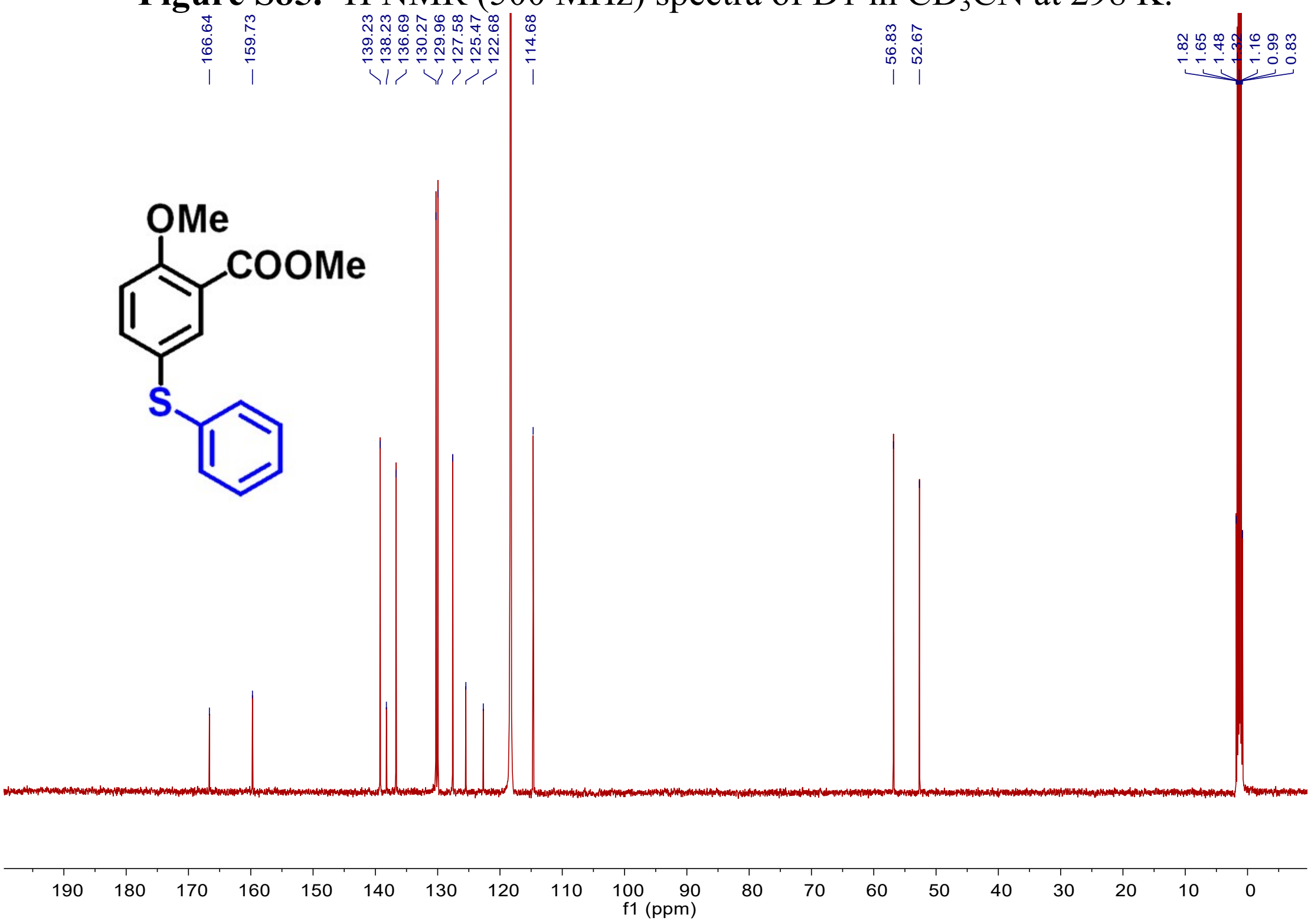

Figure S86. ^{13}C NMR (126 MHz) spectra of D1 in CD_3CN at 298 K.

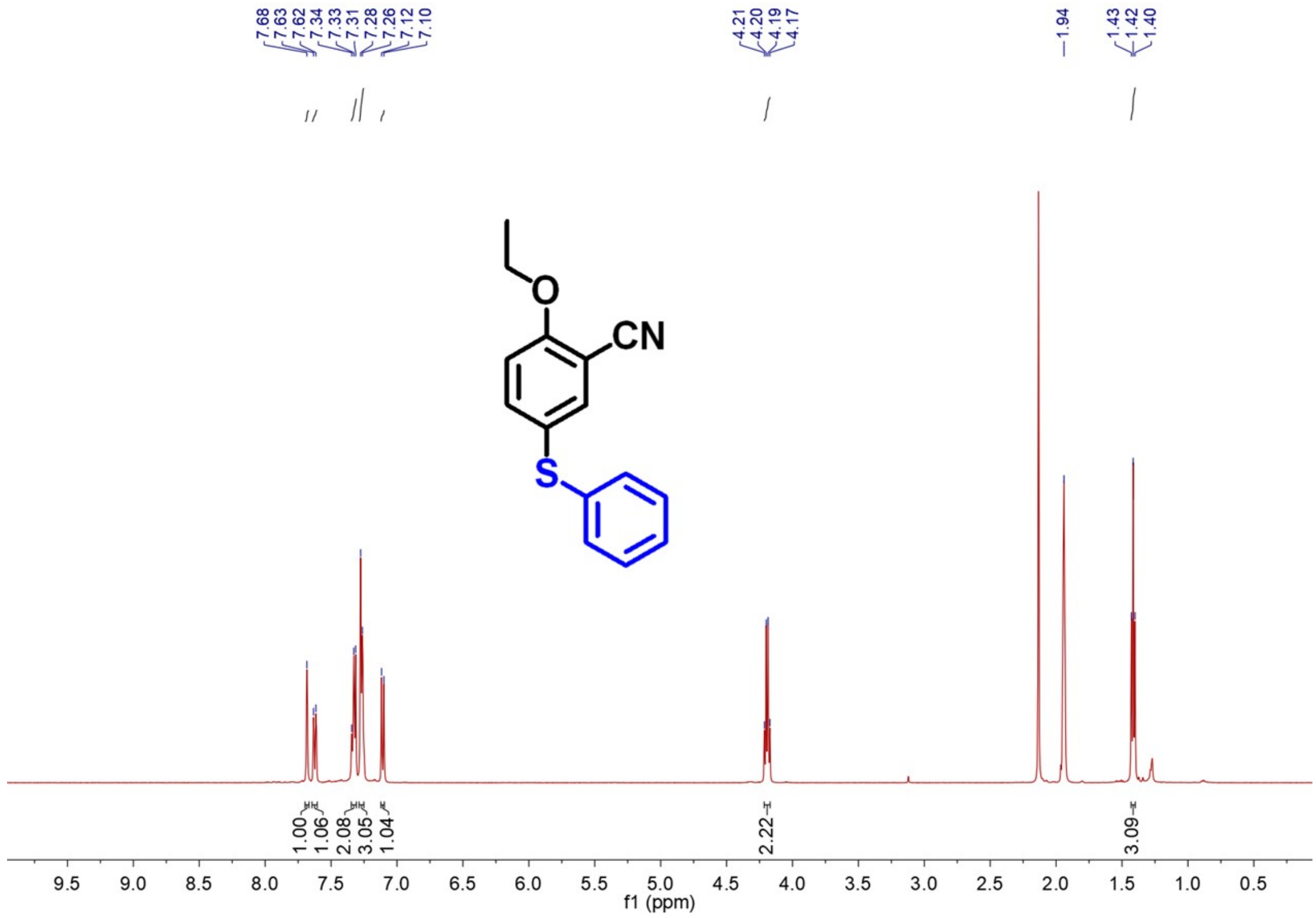

Figure S87. ^{1}H NMR (500 MHz) spectra of D2 in CD_3CN at 298 K.

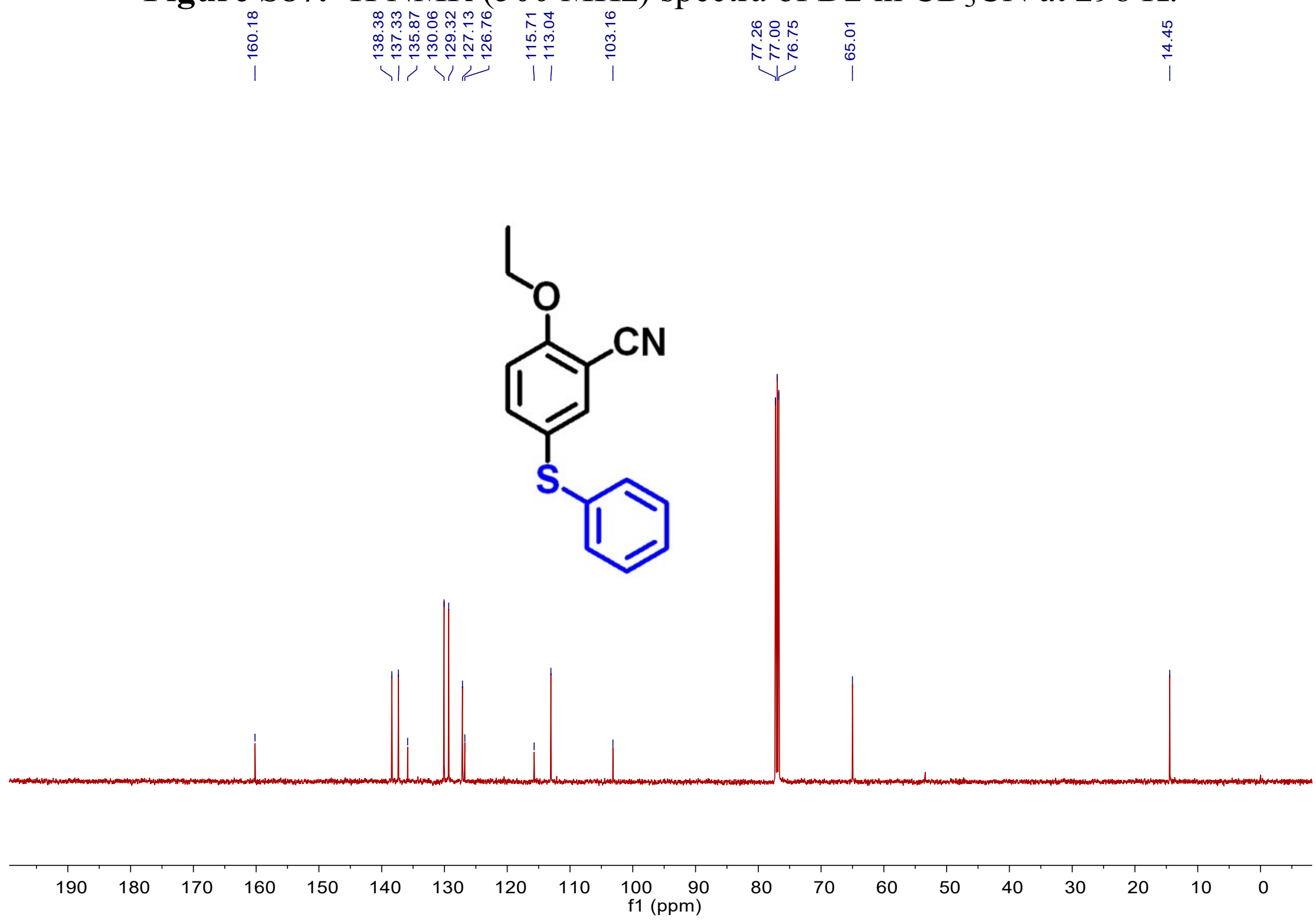

Figure S88. ^{13}C NMR (126 MHz) spectra of D2 in $CDCl_3$ at 298 K.

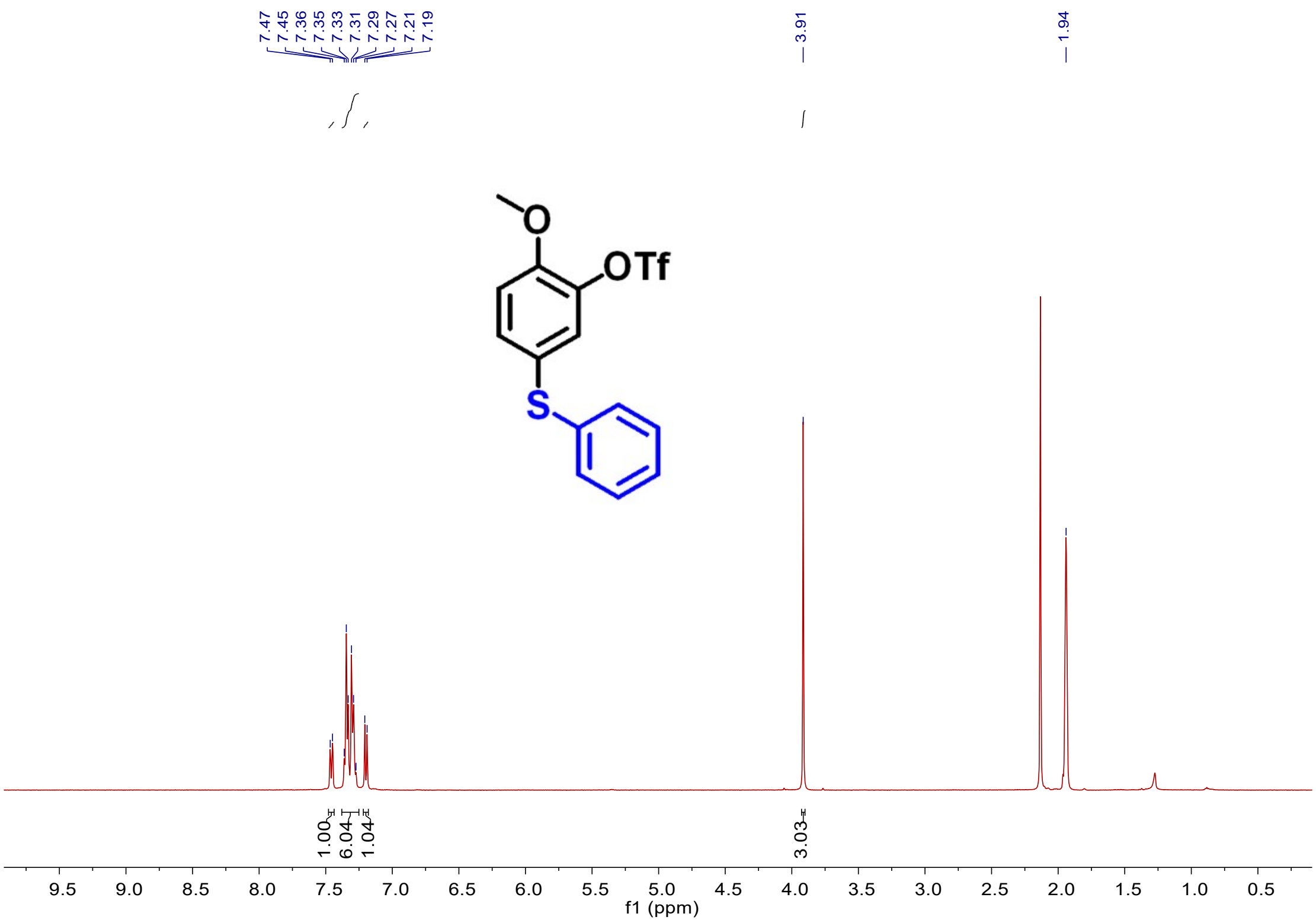

Figure S89. ^1H NMR (500 MHz) spectra of D3 in CD_3CN at 298 K.

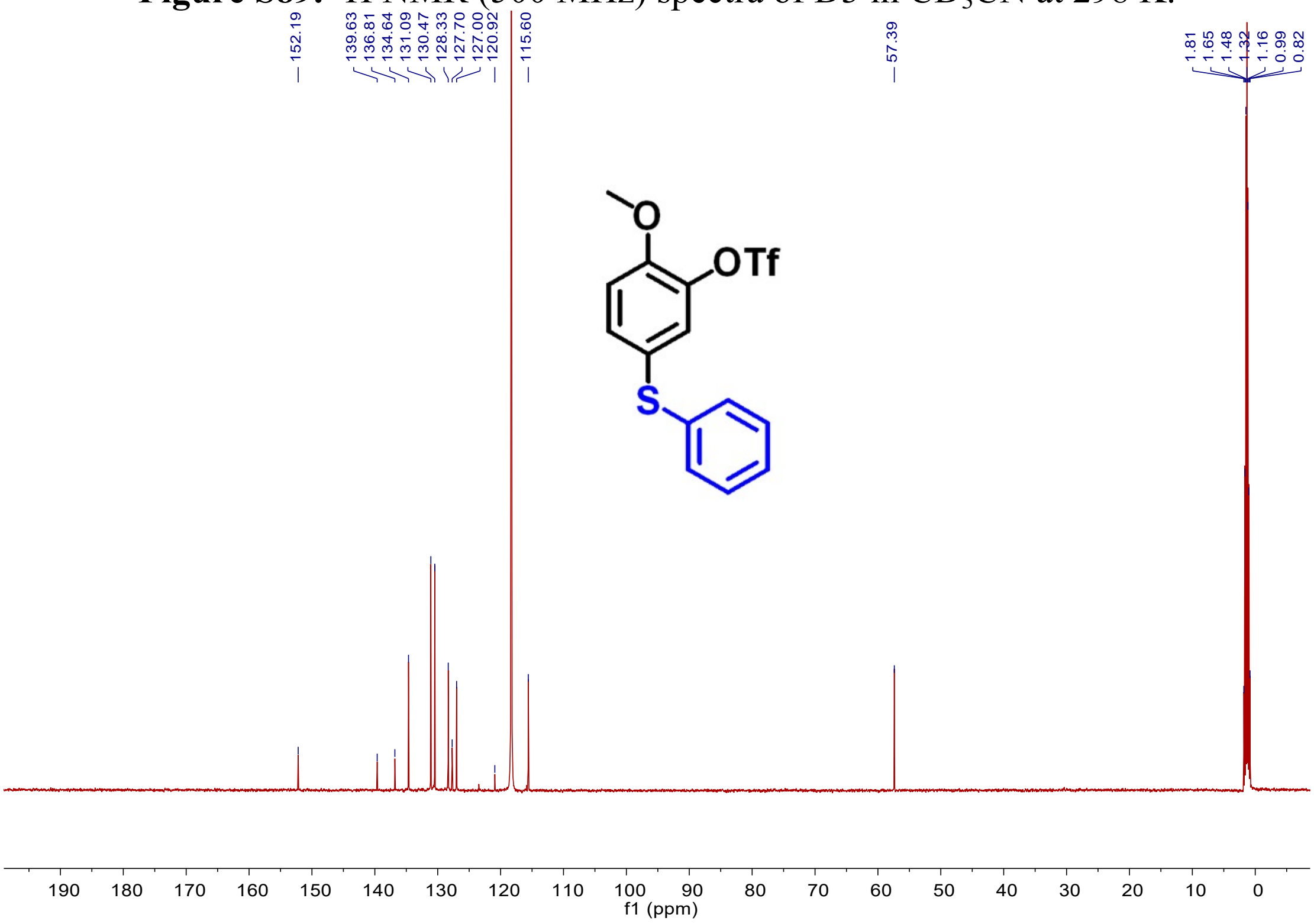

Figure S90. ^{13}C NMR (126 MHz) spectra of D3 in CD_3CN at 298 K.

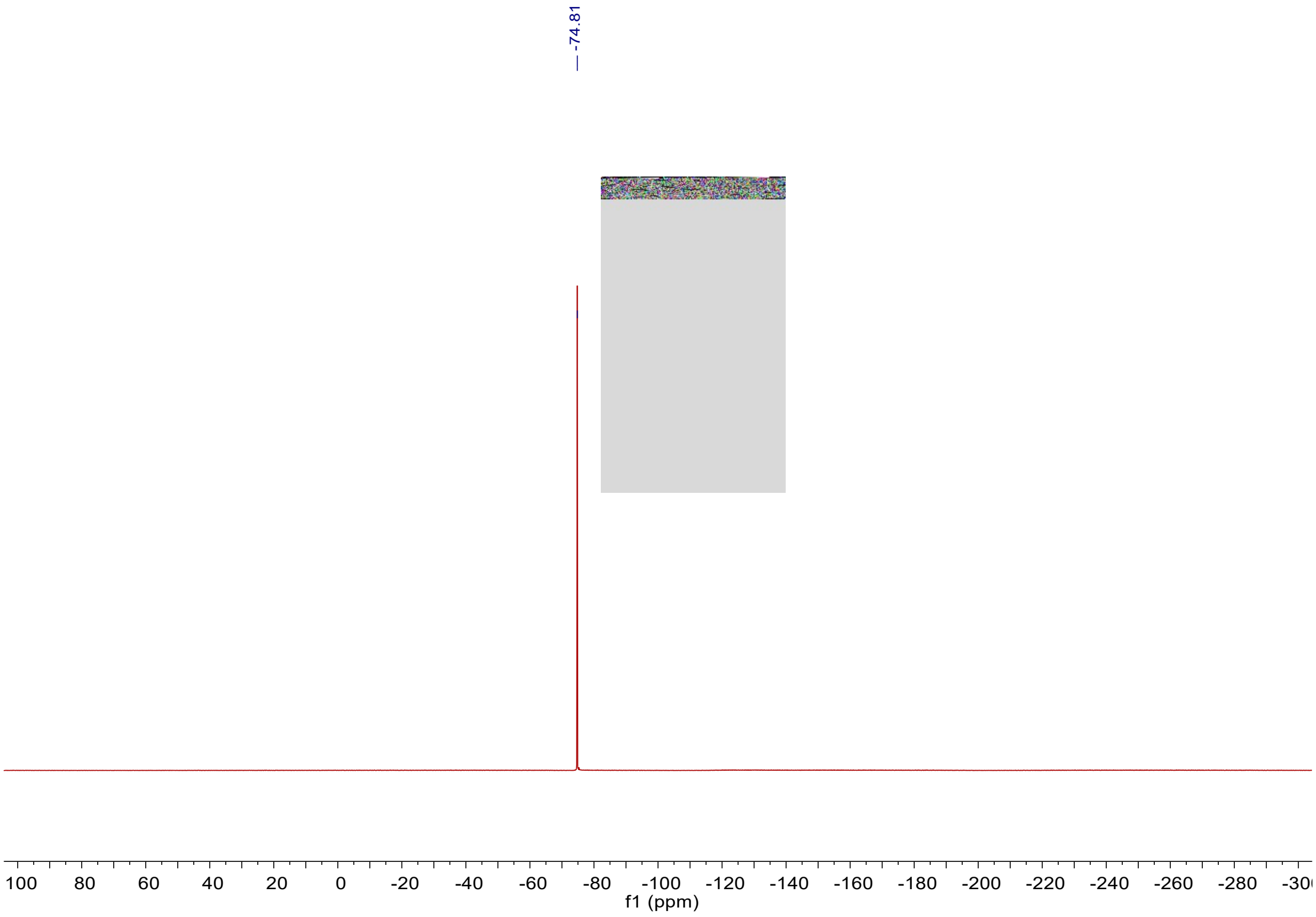

Figure S91. ^{19}F NMR (471 MHz) spectra of D3 in CD_3CN at 298 K.

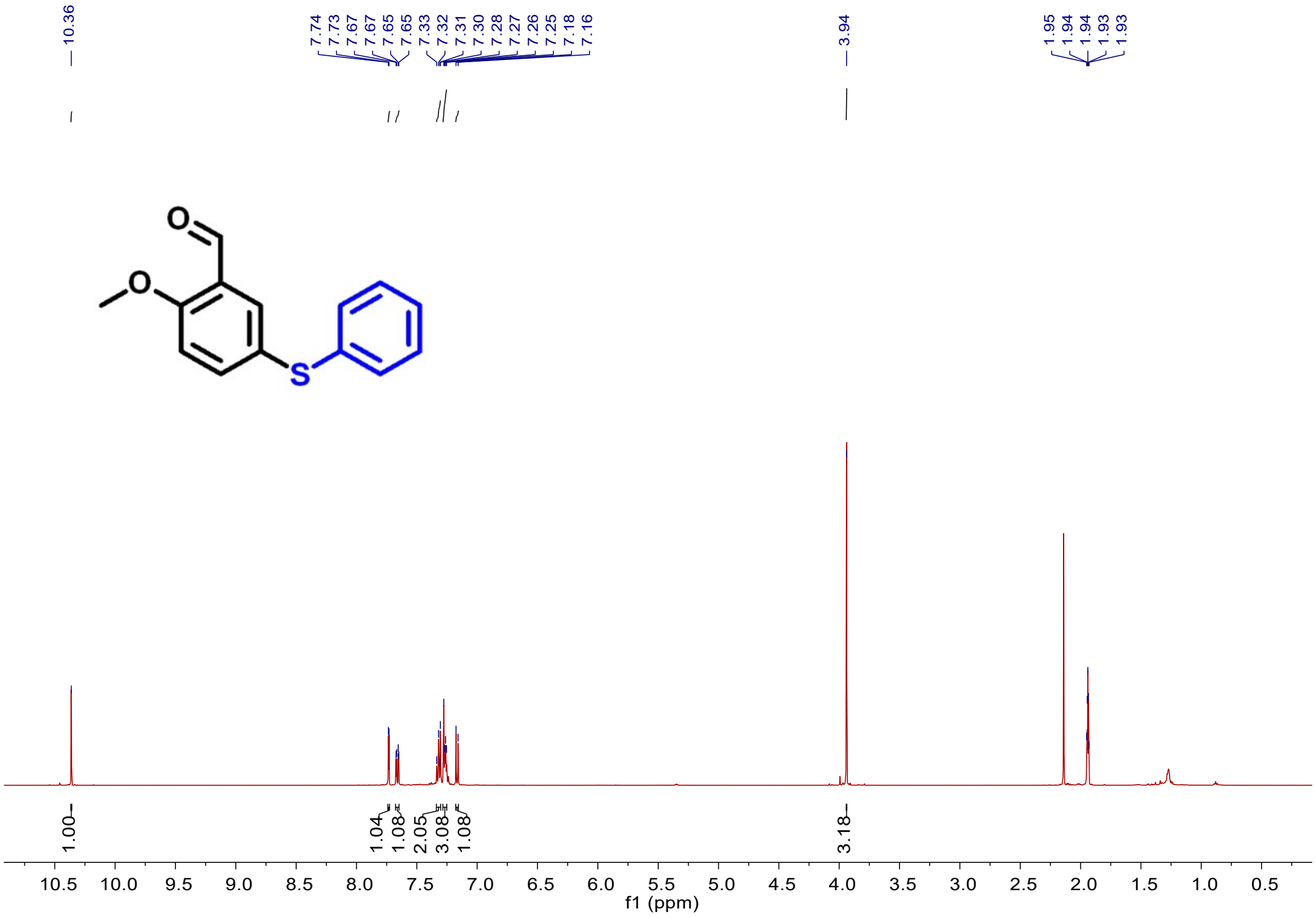

Figure S92. ^{1}H NMR (500 MHz) spectra of D4 in CD_3CN at 298 K.

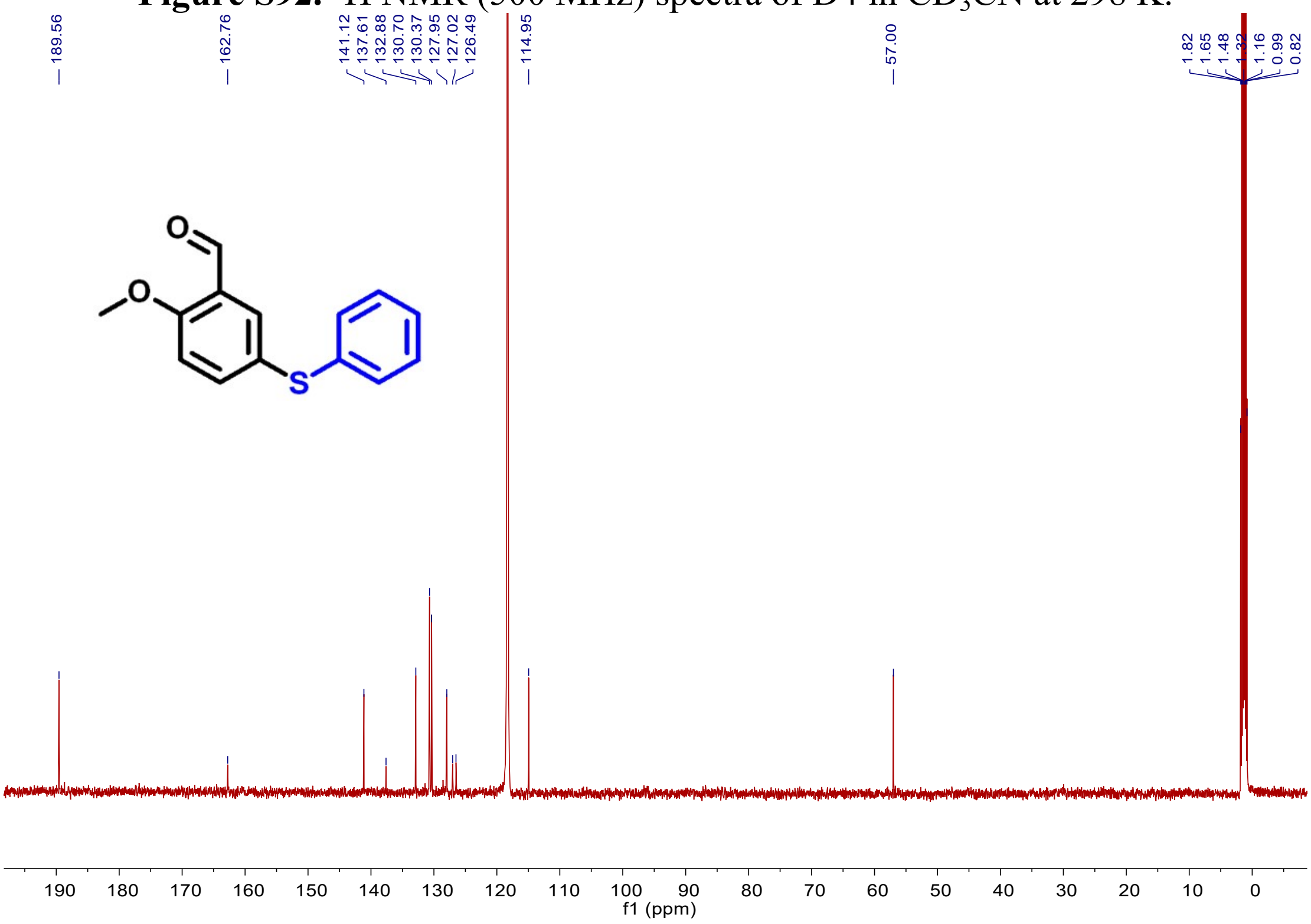

Figure S93. ^{13}C NMR (126 MHz) spectra of D4 in CD_3CN at 298 K.

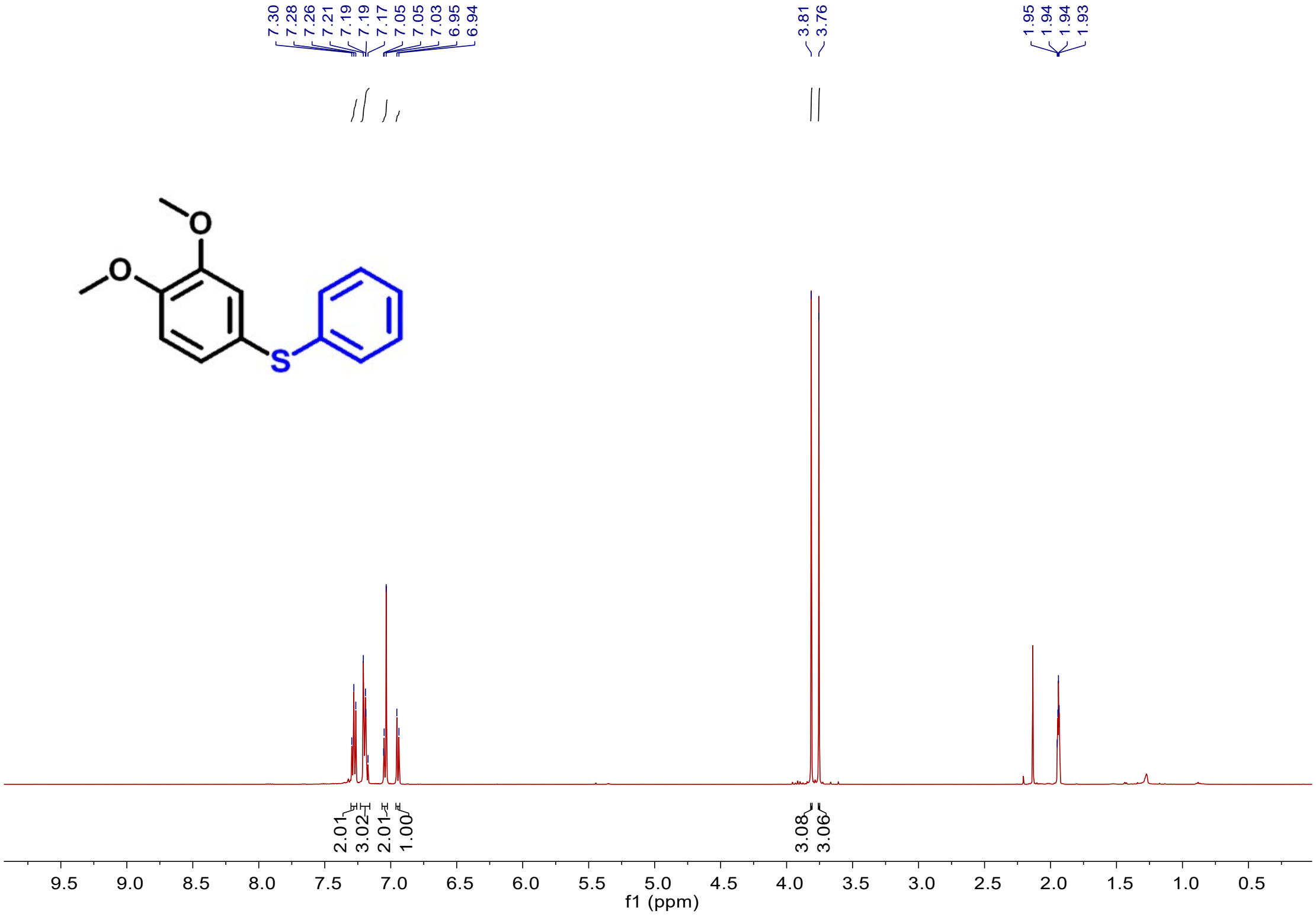

Figure S94. ^{1}H NMR (500 MHz) spectra of D5 in CD_3CN at 298 K.

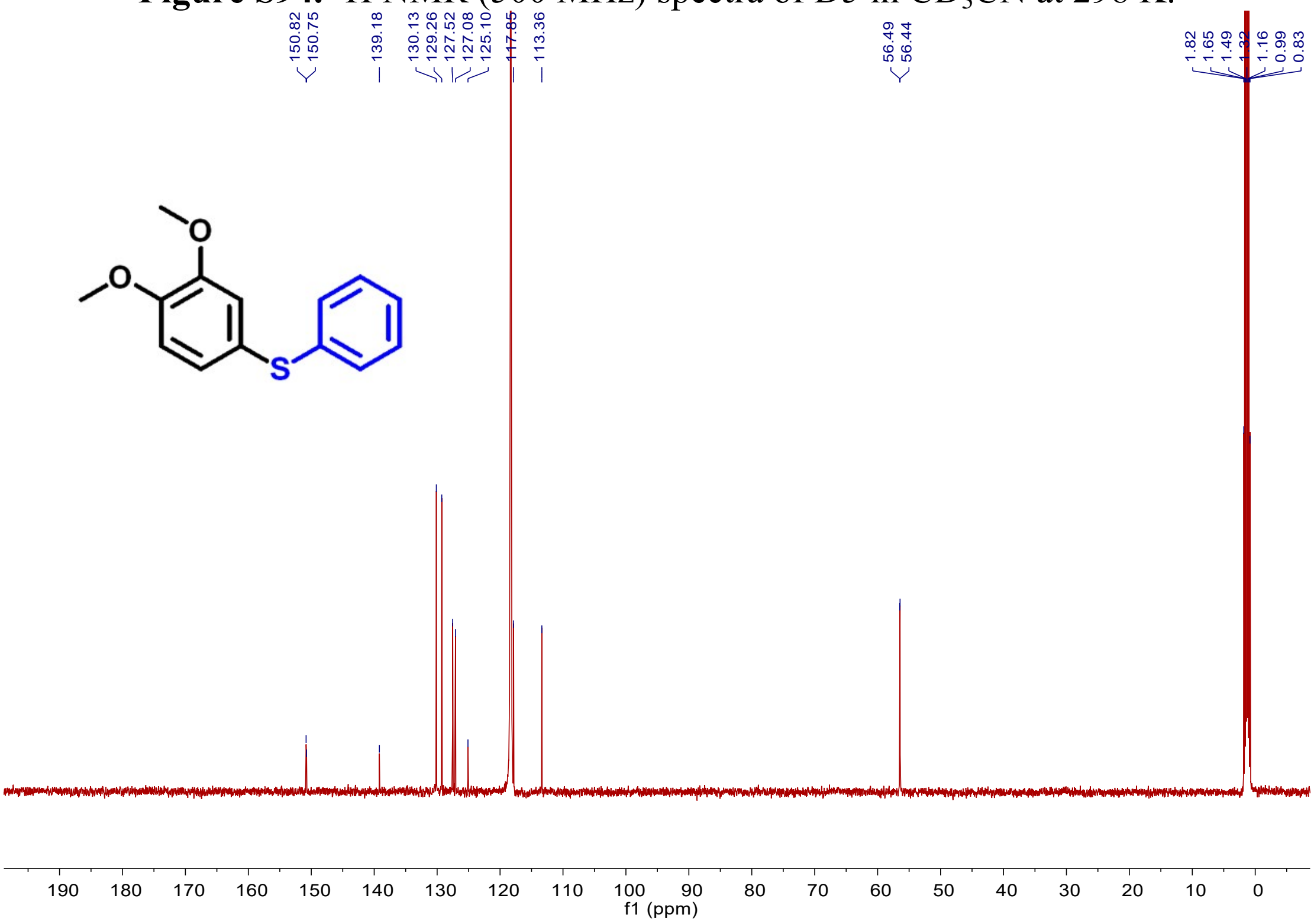

Figure S95. ^{13}C NMR (126 MHz) spectra of D5 in CD_3CN at 298 K.

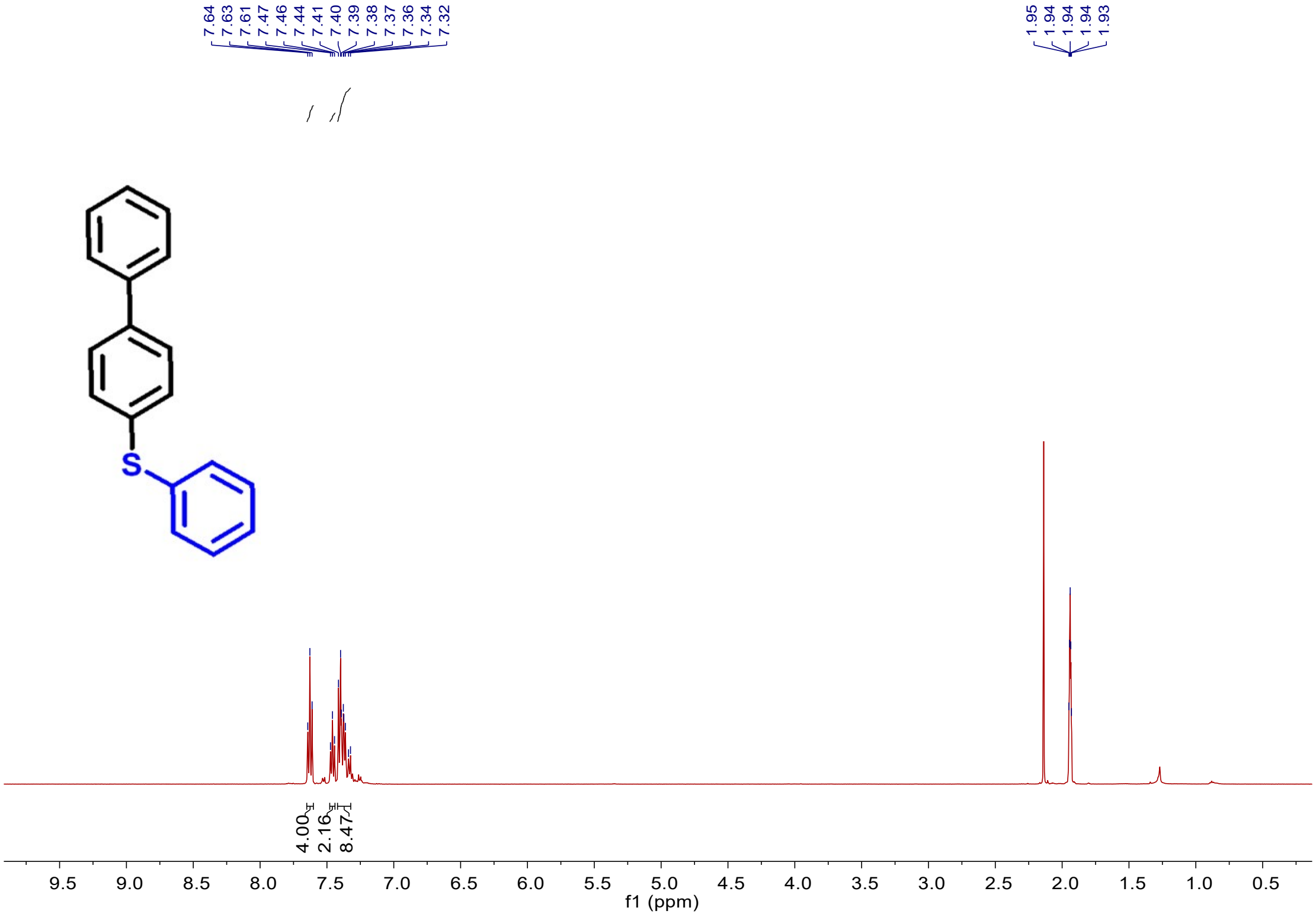

Figure S96. ^{1}H NMR (500 MHz) spectra of D6 in CD_3CN at 298 K.

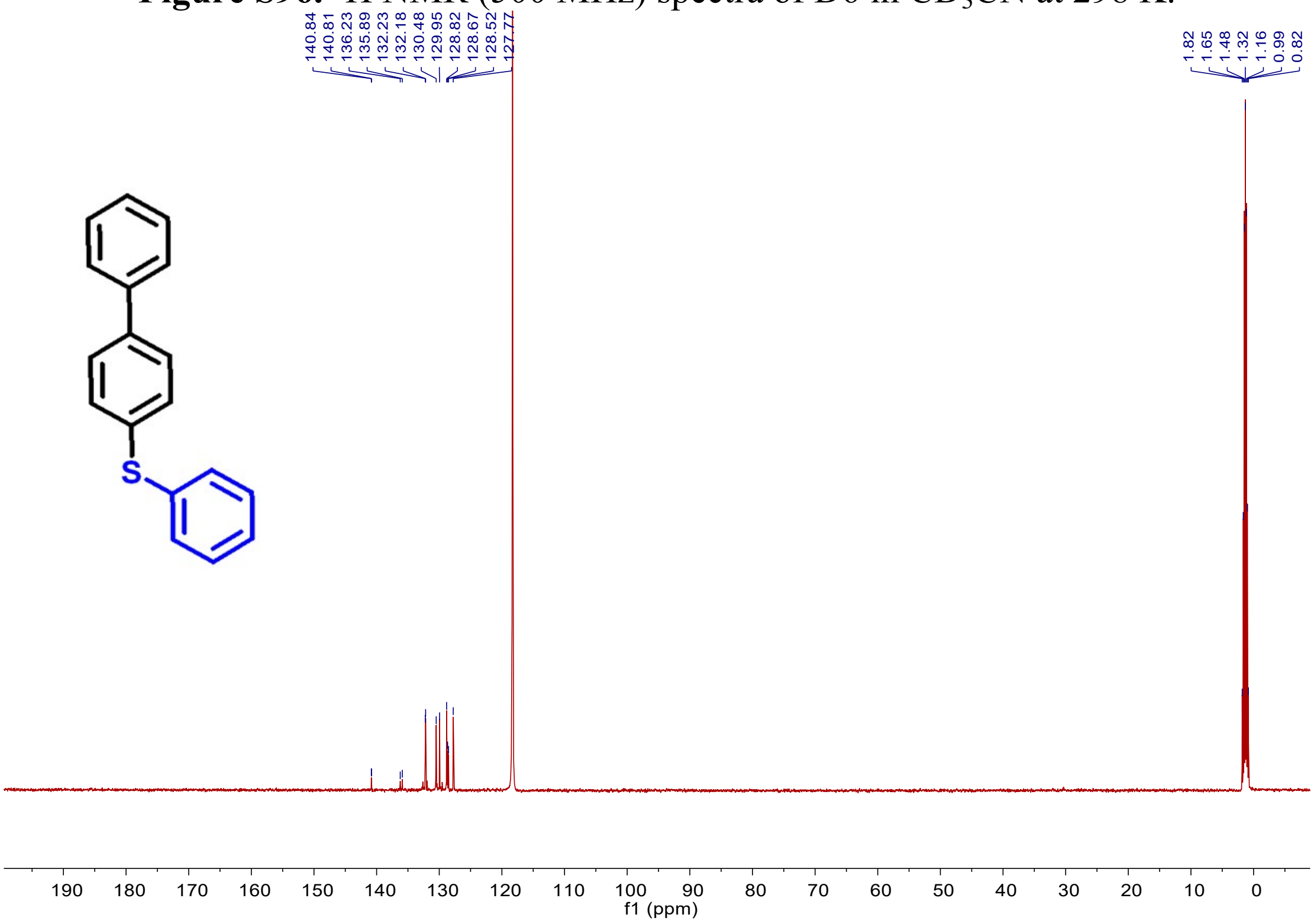

Figure S97. ^{13}C NMR (126 MHz) spectra of D6 in CD_3CN at 298 K.

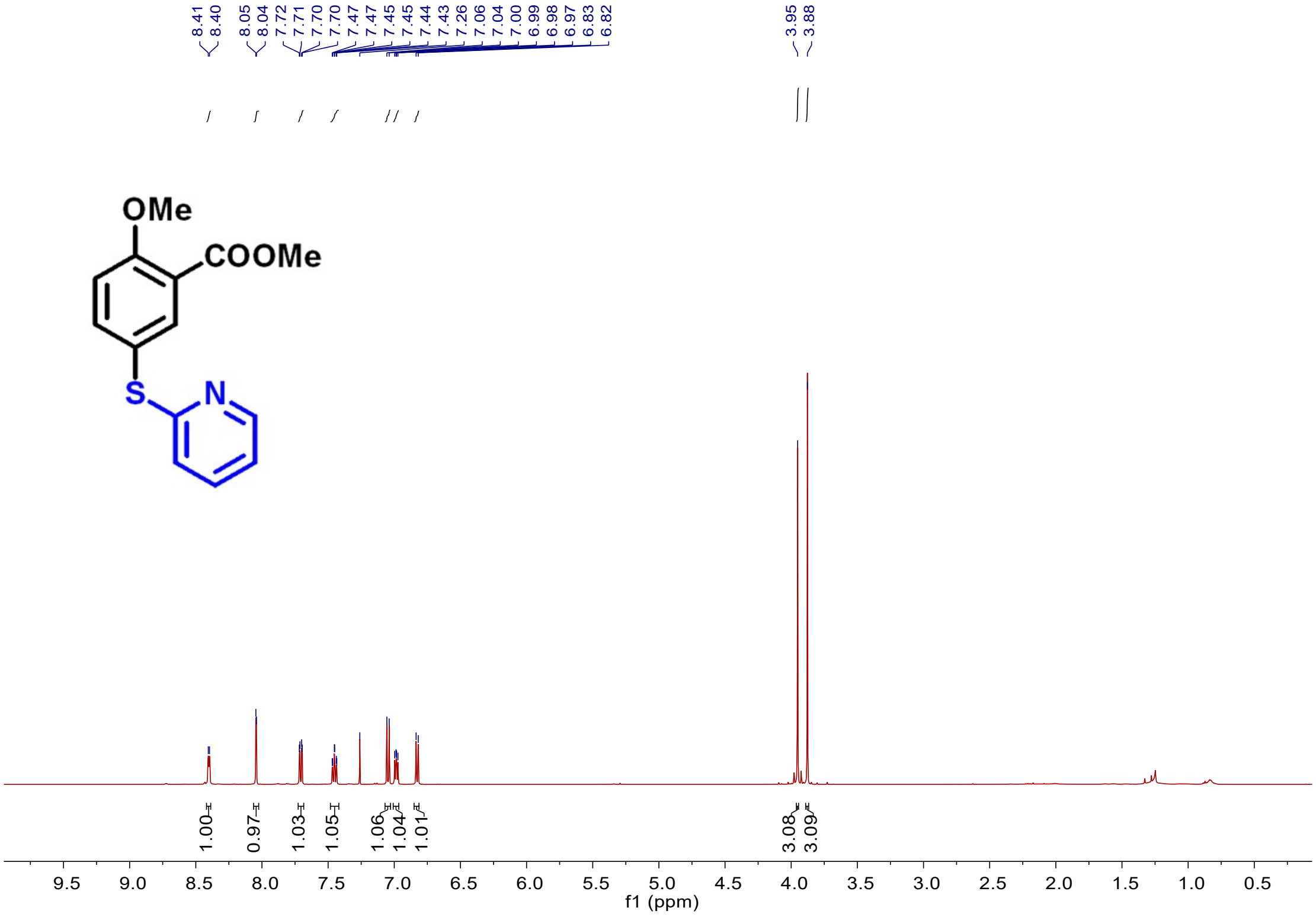

Figure S98. ^{1}H NMR (500 MHz) spectra of D7 in $CDCl_3$ at 298 K.

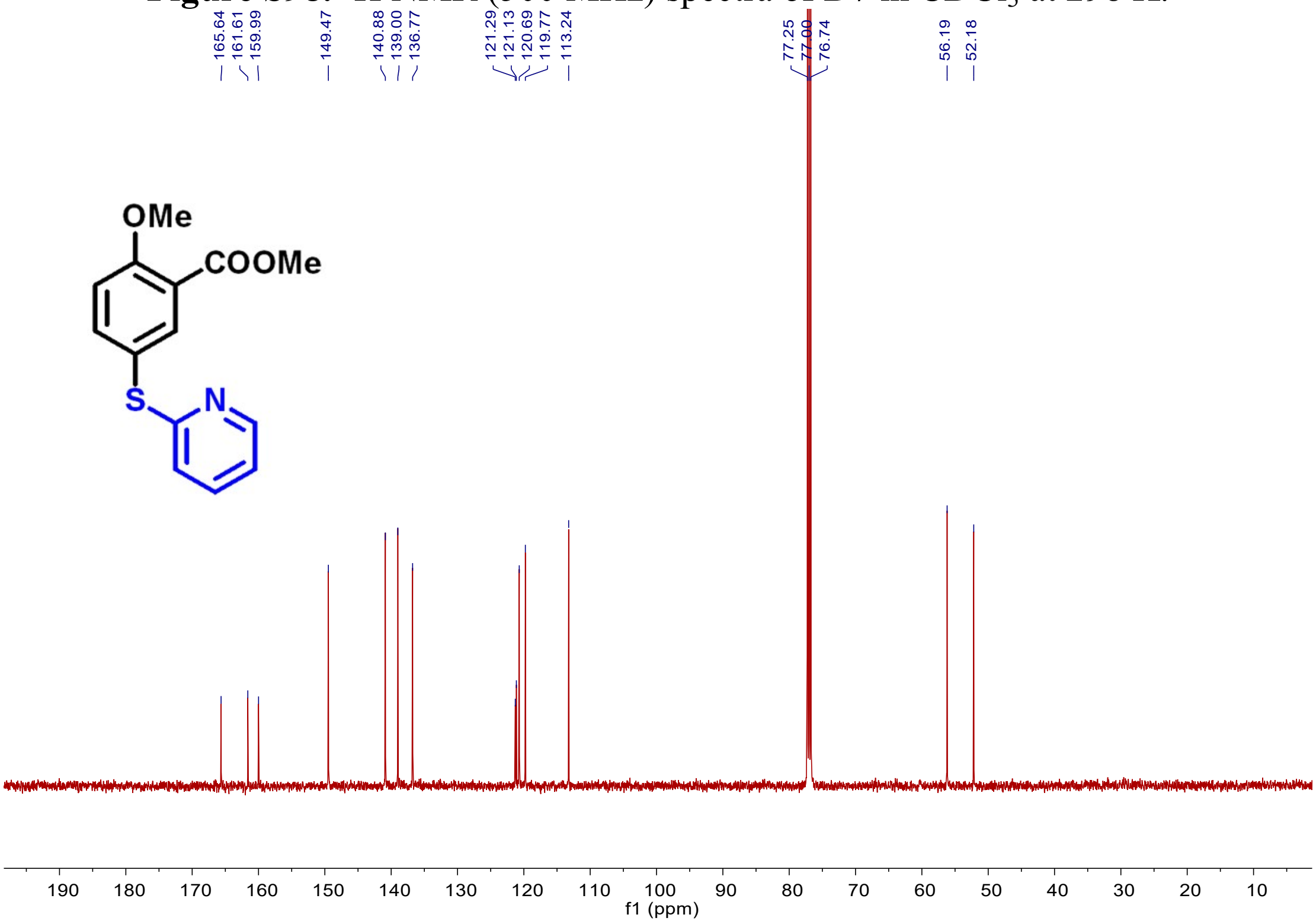

Figure S99. ^{13}C NMR (126 MHz) spectra of D7 in $CDCl_3$ at 298 K.

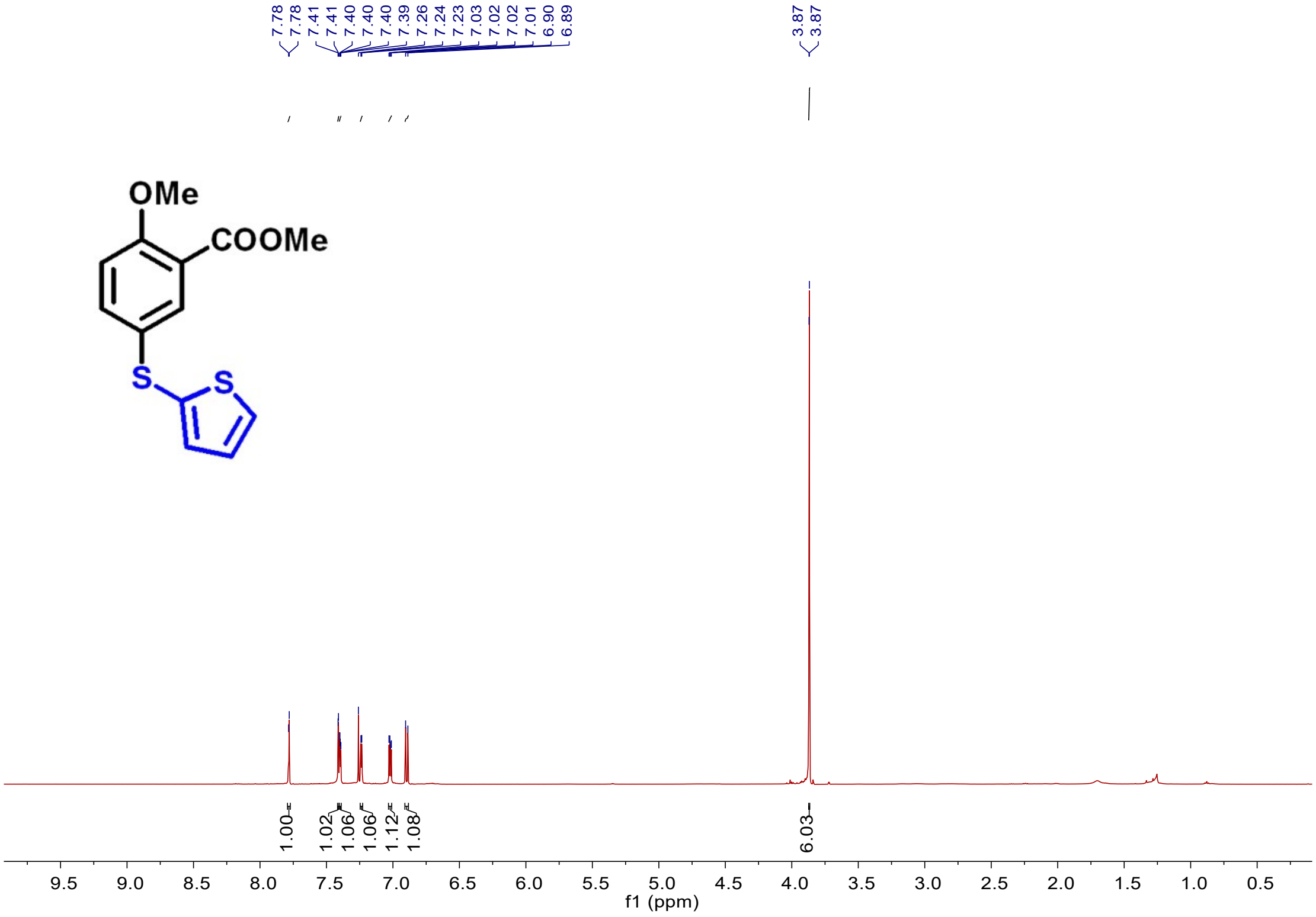

Figure S100. ^{1}H NMR (500 MHz) spectra of D8 in $CDCl_3$ at 298 K.

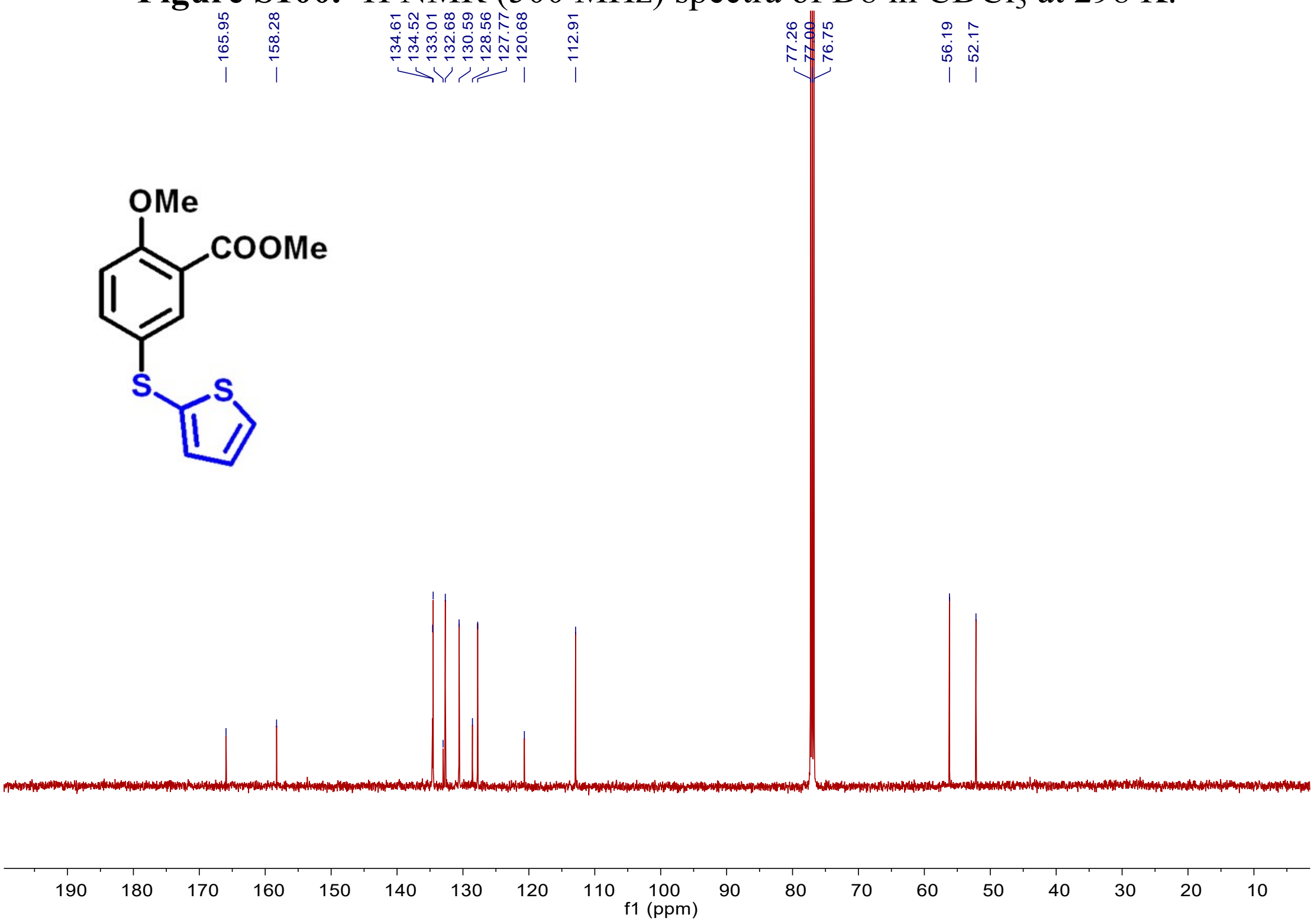

Figure S101. ^{13}C NMR (126 MHz) spectra of D8 in $CDCl_3$ at 298 K.

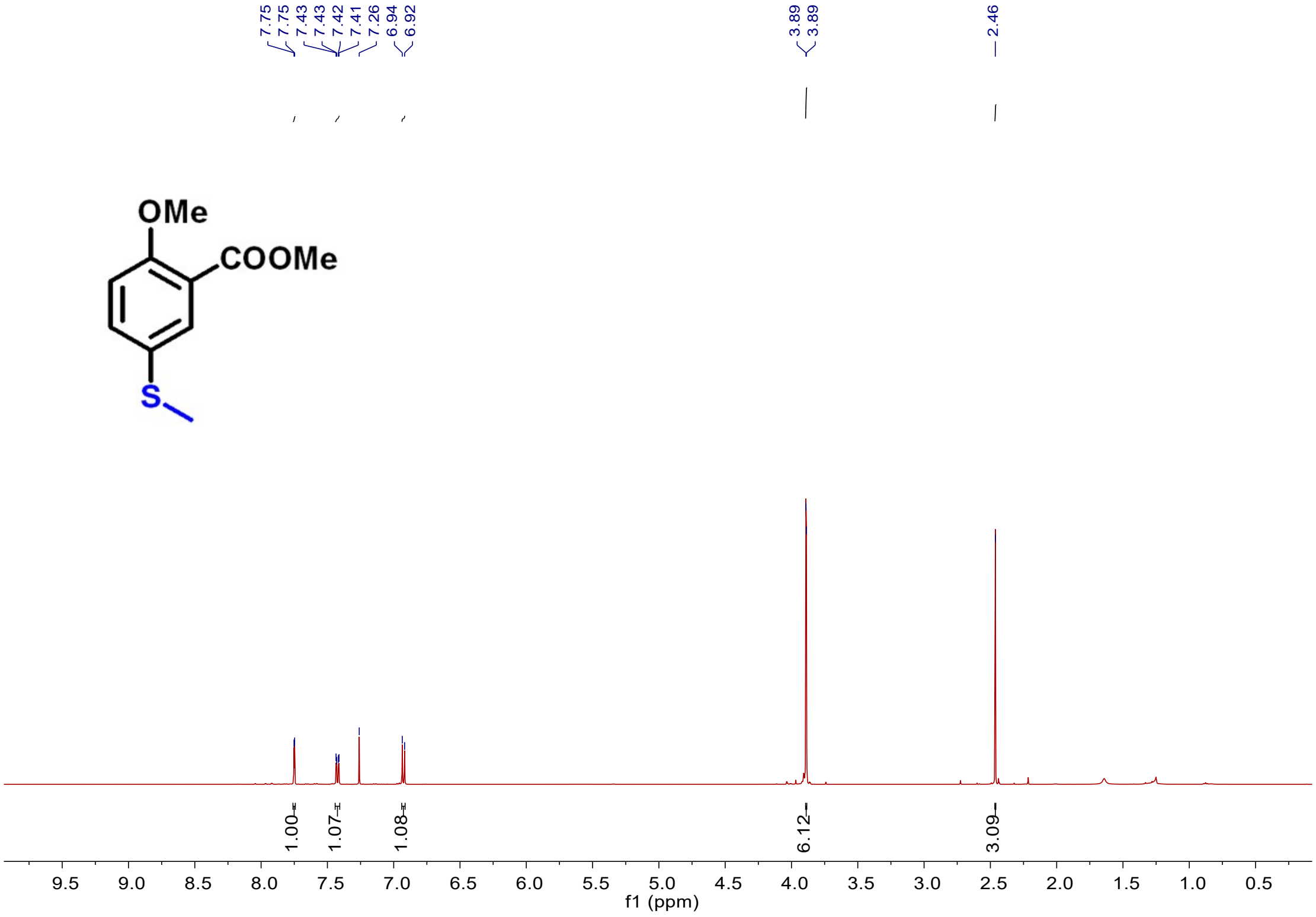

Figure S102. ^{1}H NMR (500 MHz) spectra of D9 in $CDCl_3$ at 298 K.

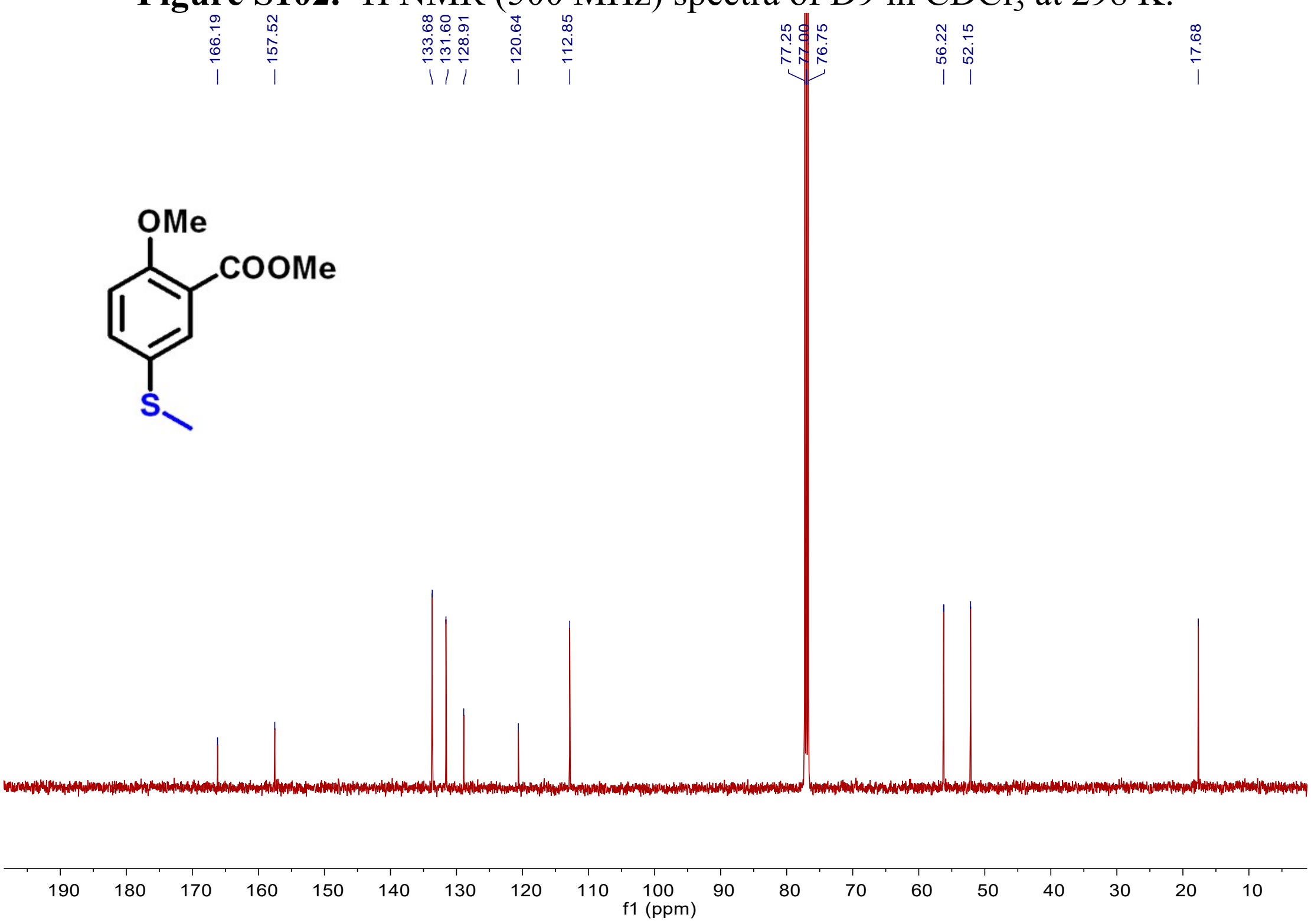

Figure S103. ^{13}C NMR (126 MHz) spectra of D9 in $CDCl_3$ at 298 K.

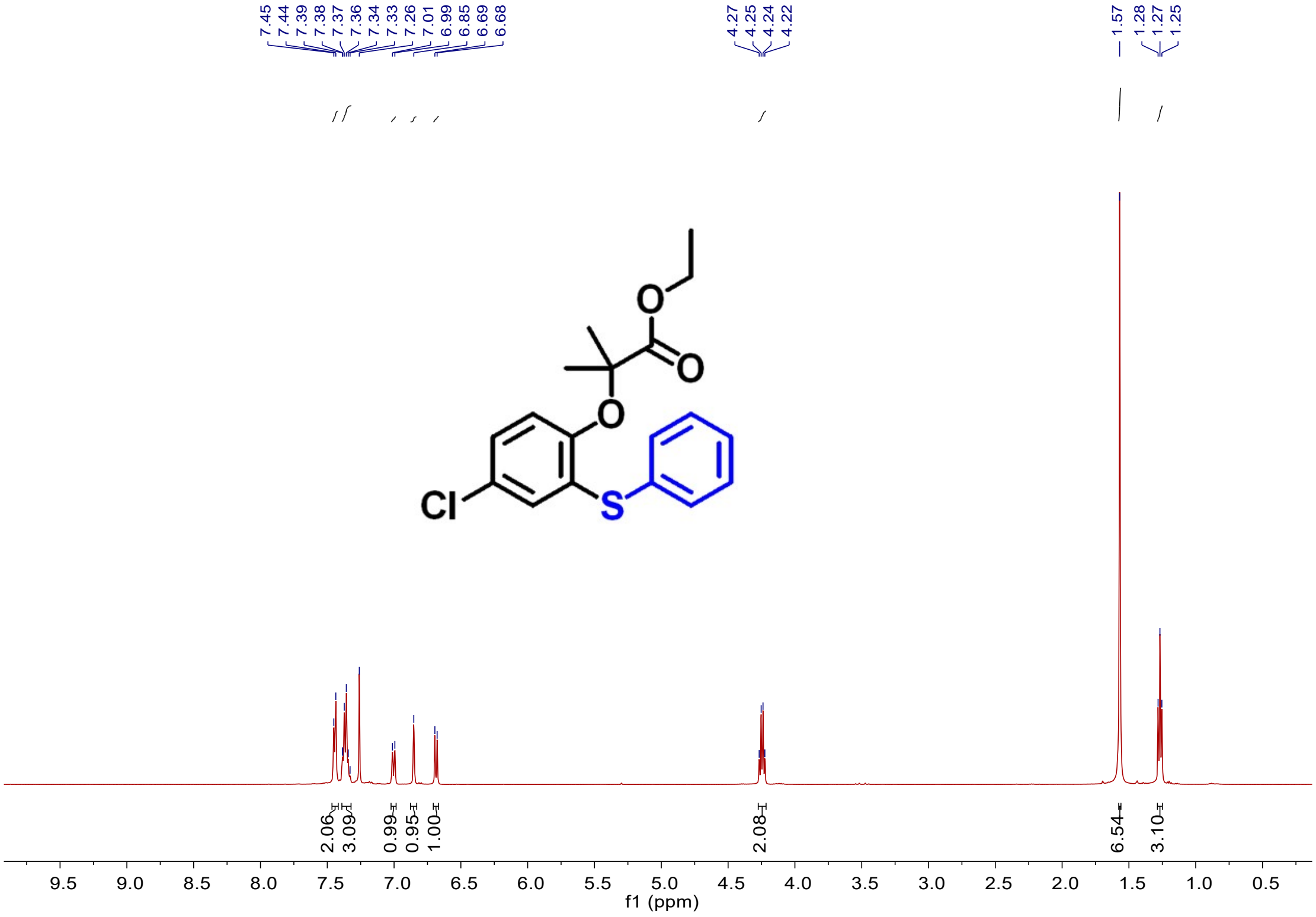

Figure S104. ^{1}H NMR (500 MHz) spectra of D10 in $CDCl_3$ at 298 K.

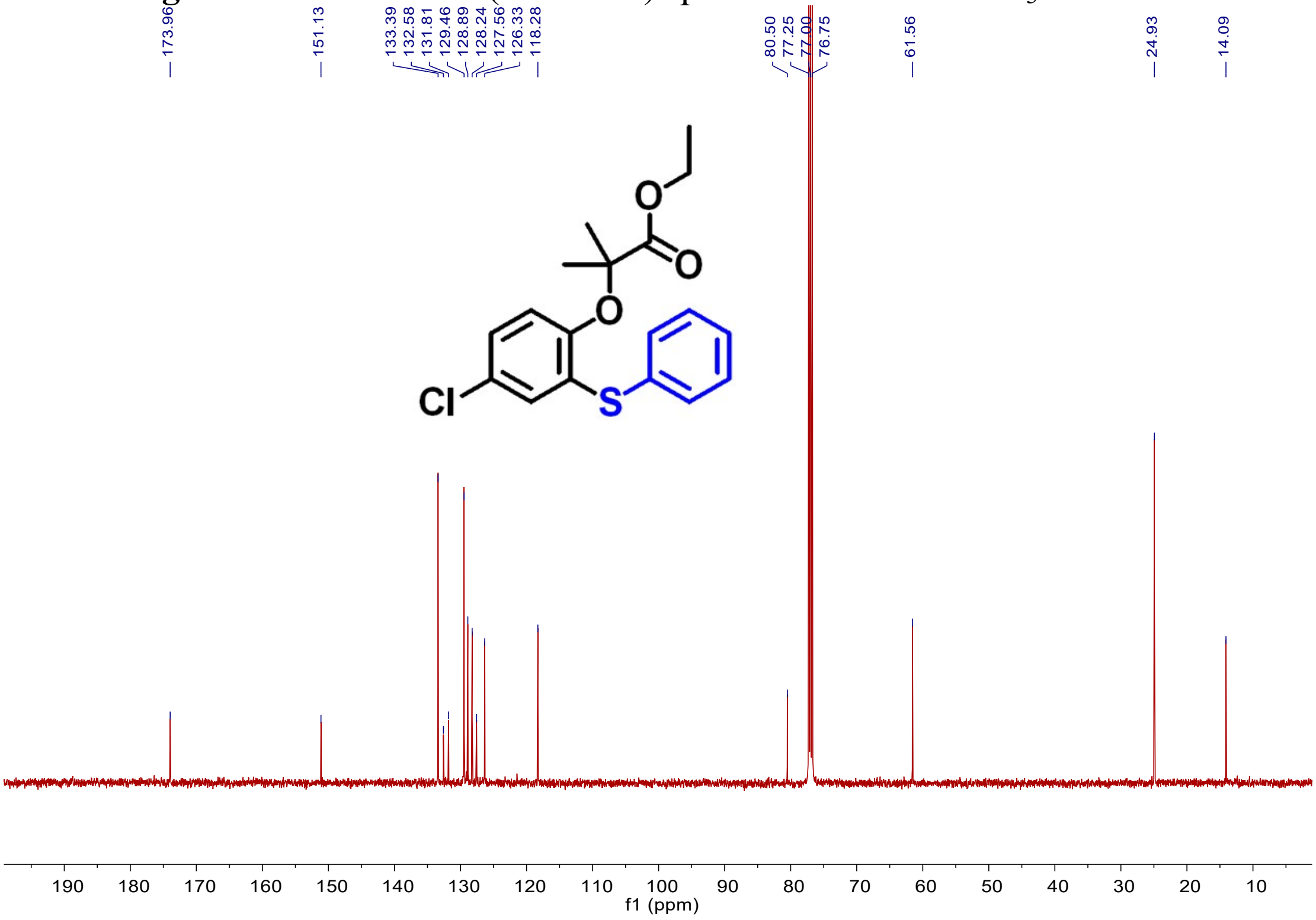

Figure S105. ^{13}C NMR (126 MHz) spectra of D10 in $CDCl_3$ at 298 K.

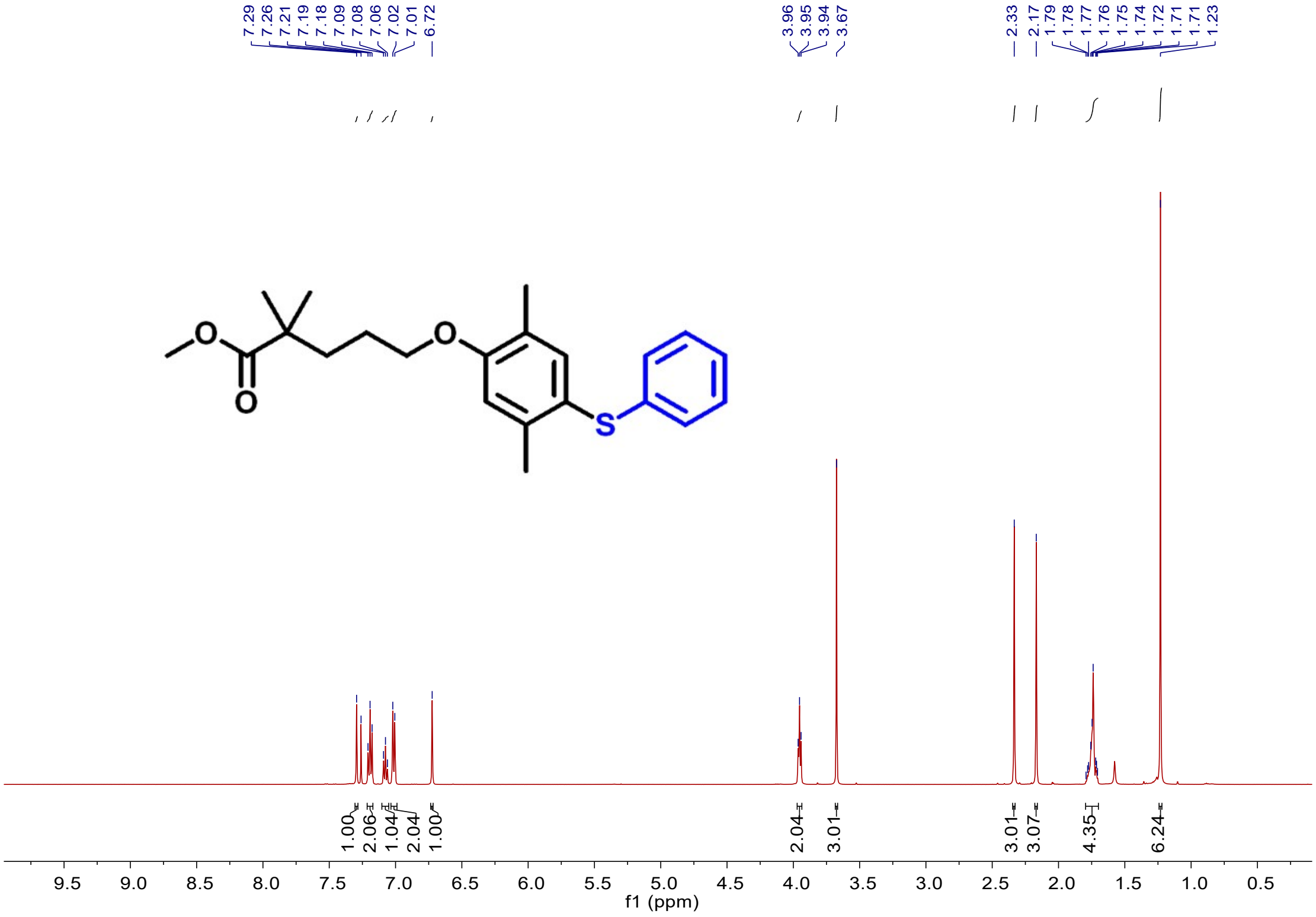

Figure S106. ^{1}H NMR (500 MHz) spectra of D11 in $CDCl_3$ at 298 K.

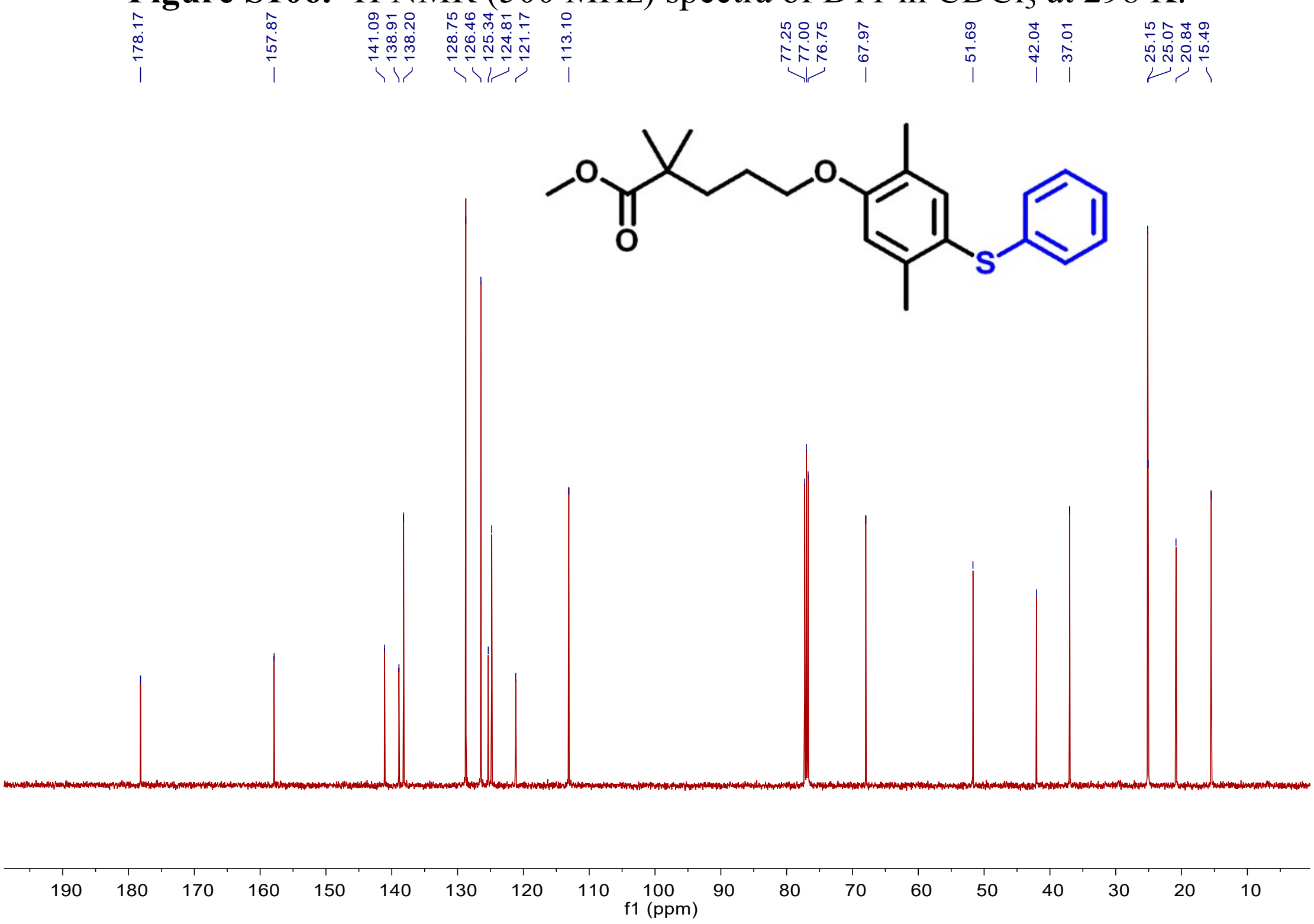

Figure S107. ^{13}C NMR (126 MHz) spectra of D11 in $CDCl_3$ at 298 K.

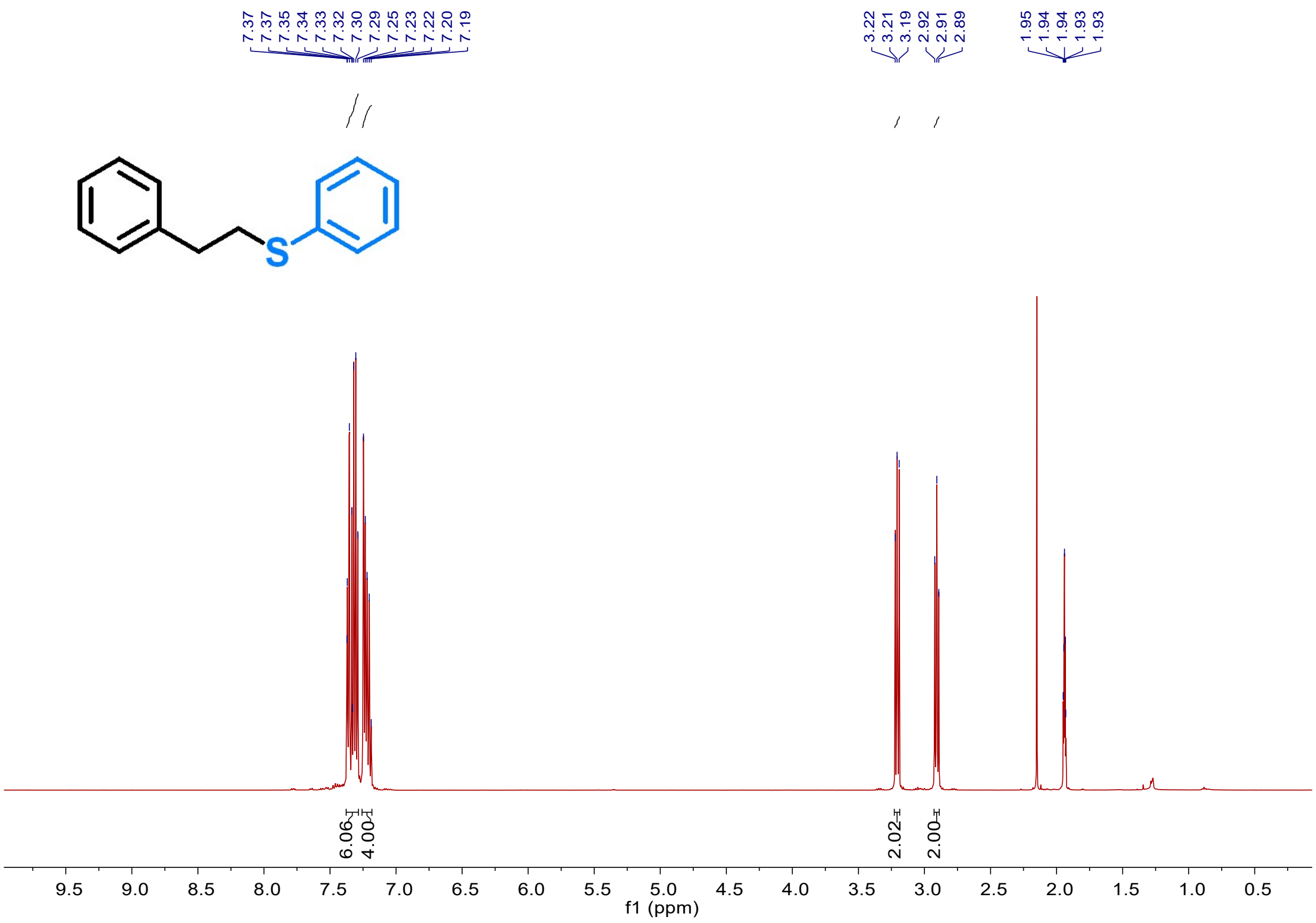

Figure S108. ^{1}H NMR (500 MHz) spectra of E1 in CD_3CN at 298 K.

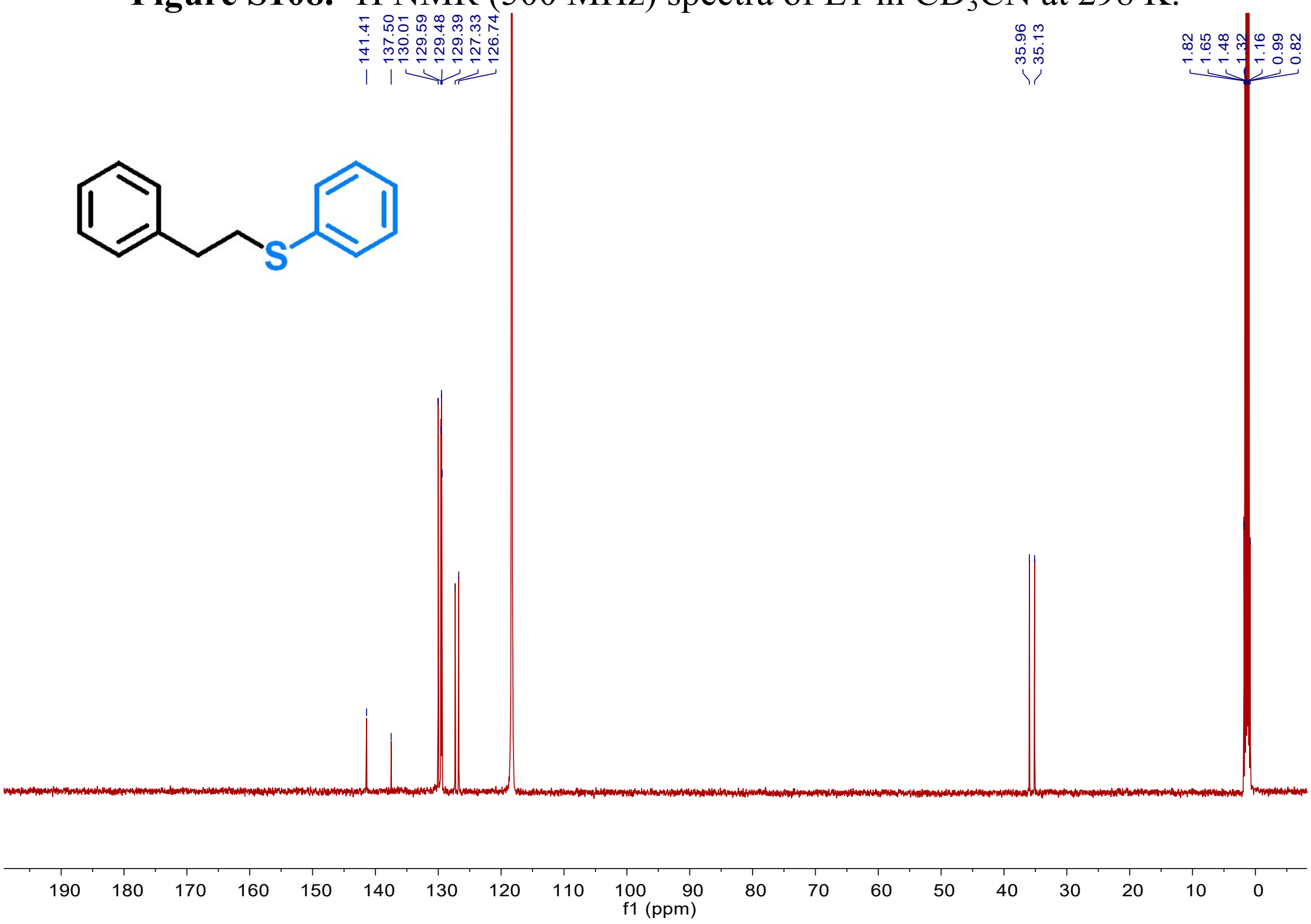

Figure S109. ^{13}C NMR (126 MHz) spectra of E1 in CD_3CN at 298 K.

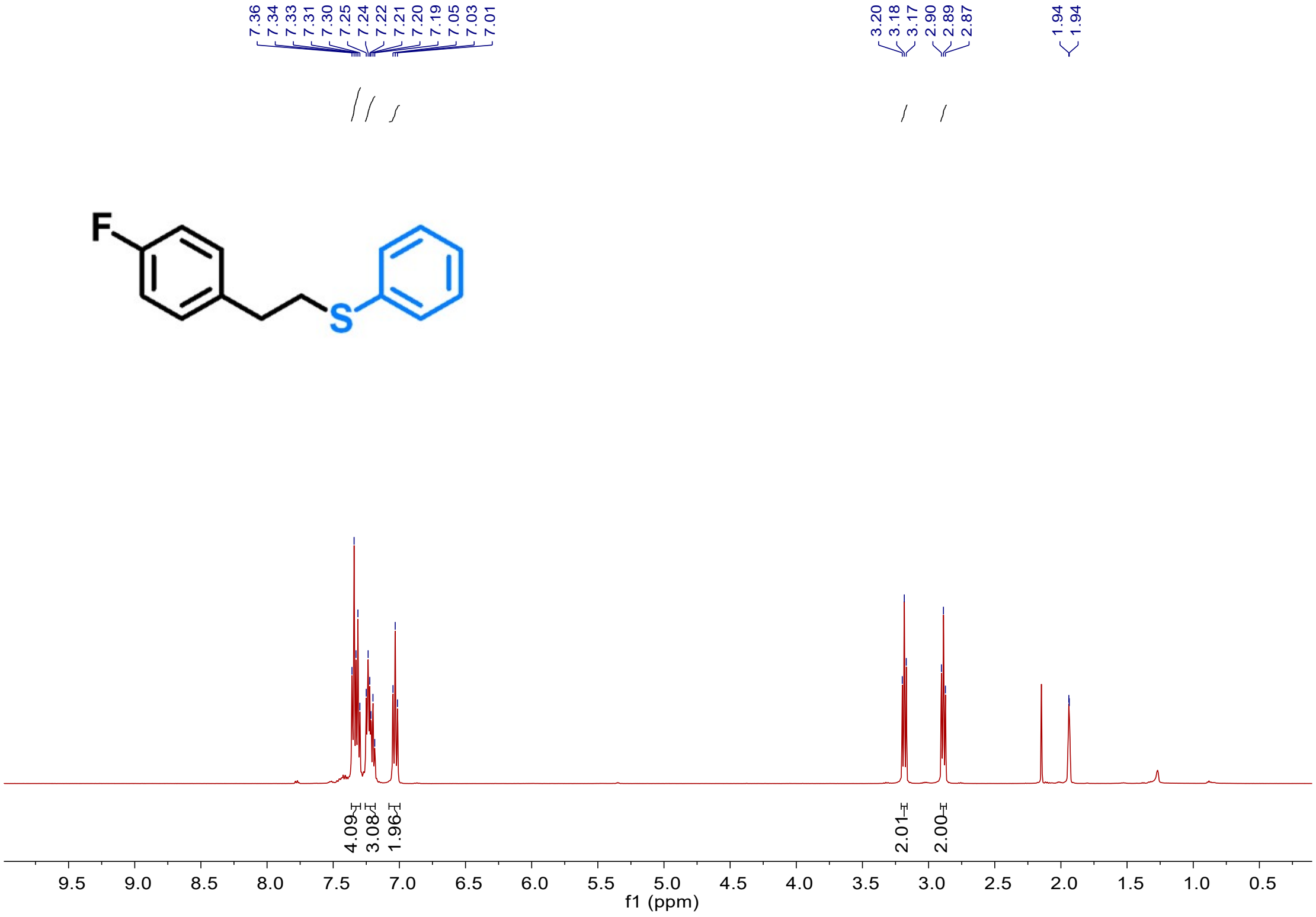

Figure S110. ^{1}H NMR (500 MHz) spectra of E2 in CD_3CN at 298 K.

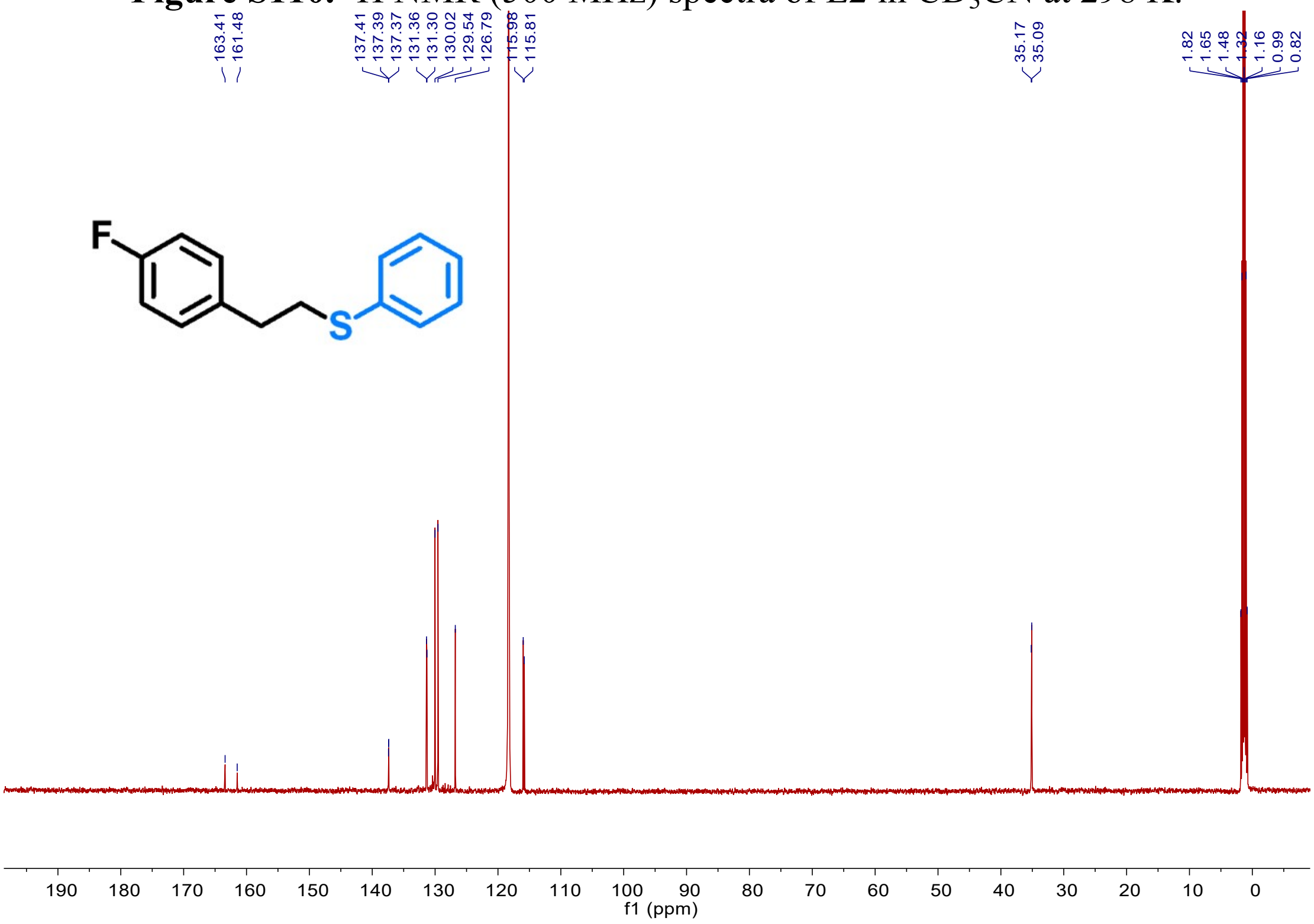

Figure S111. ^{13}C NMR (126 MHz) spectra of E2 in CD_3CN at 298 K.

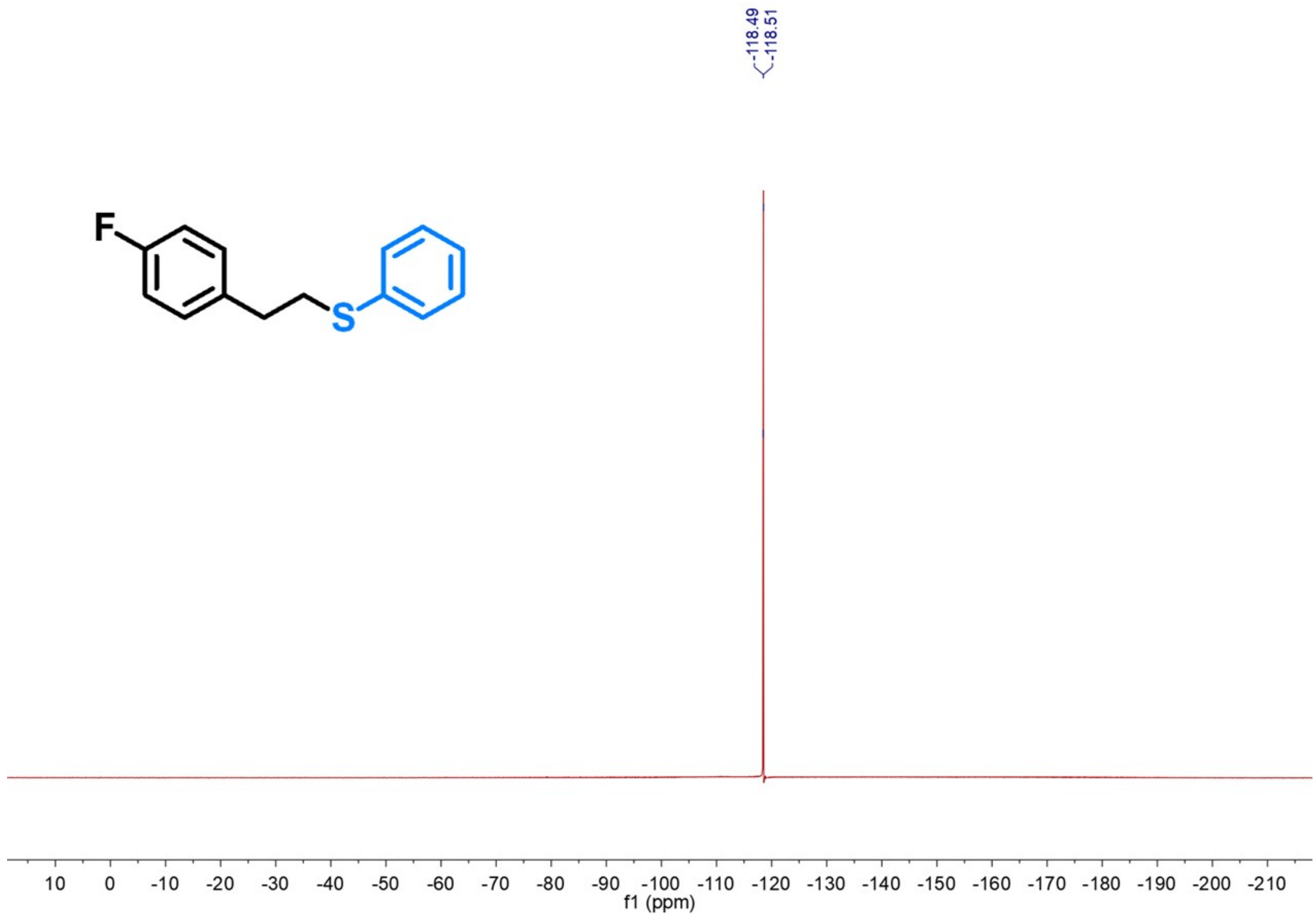

Figure S112. ^{19}F NMR (471 MHz) spectra of E2 in CD_3CN at 298 K.

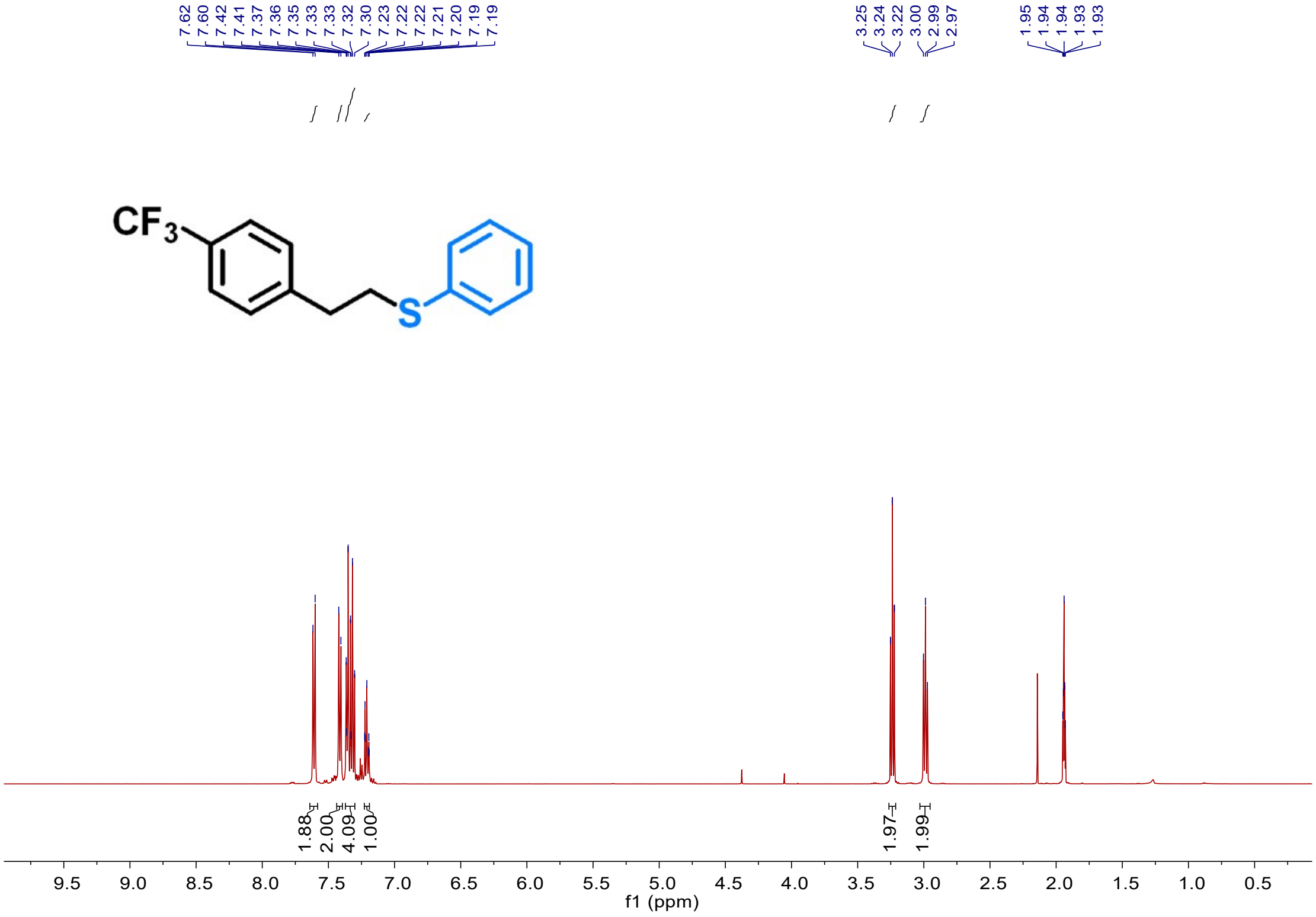

Figure S113. ^{1}H NMR (500 MHz) spectra of E3 in CD_3CN at 298 K.

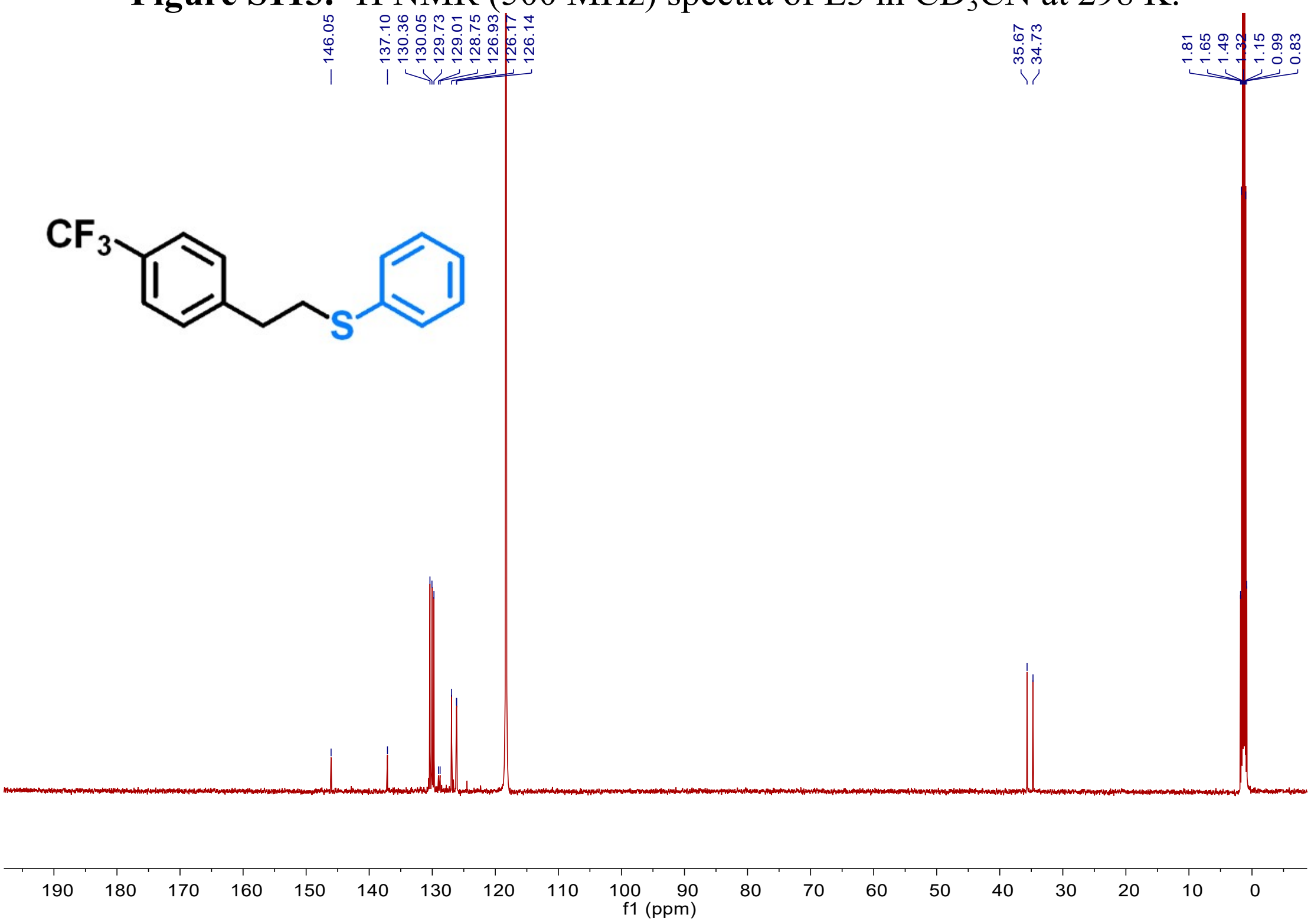

Figure S114. ^{13}C NMR (126 MHz) spectra of E3 in CD_3CN at 298 K.

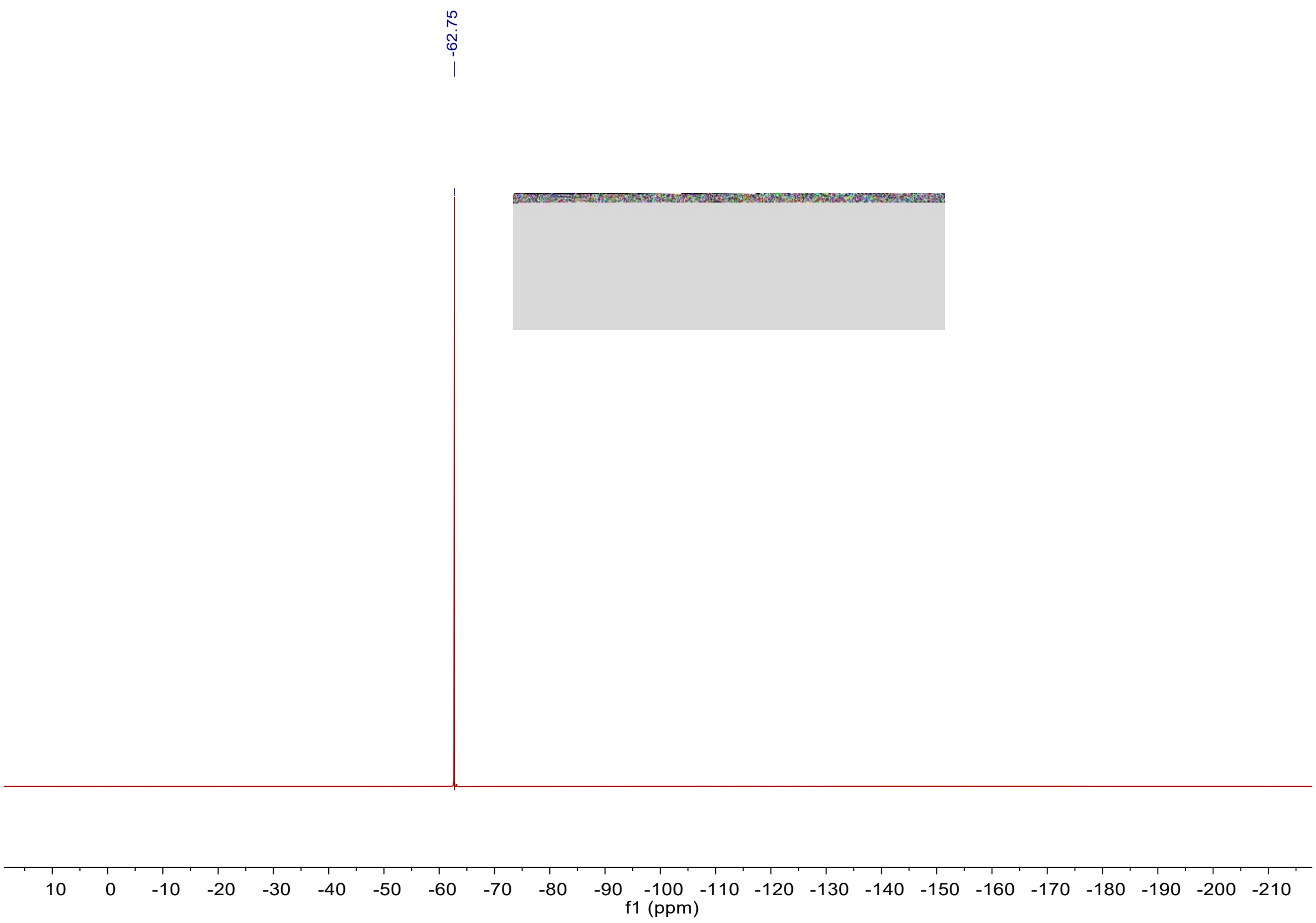

Figure S115. ^{19}F NMR (471 MHz) spectra of E3 in CD_3CN at 298 K.

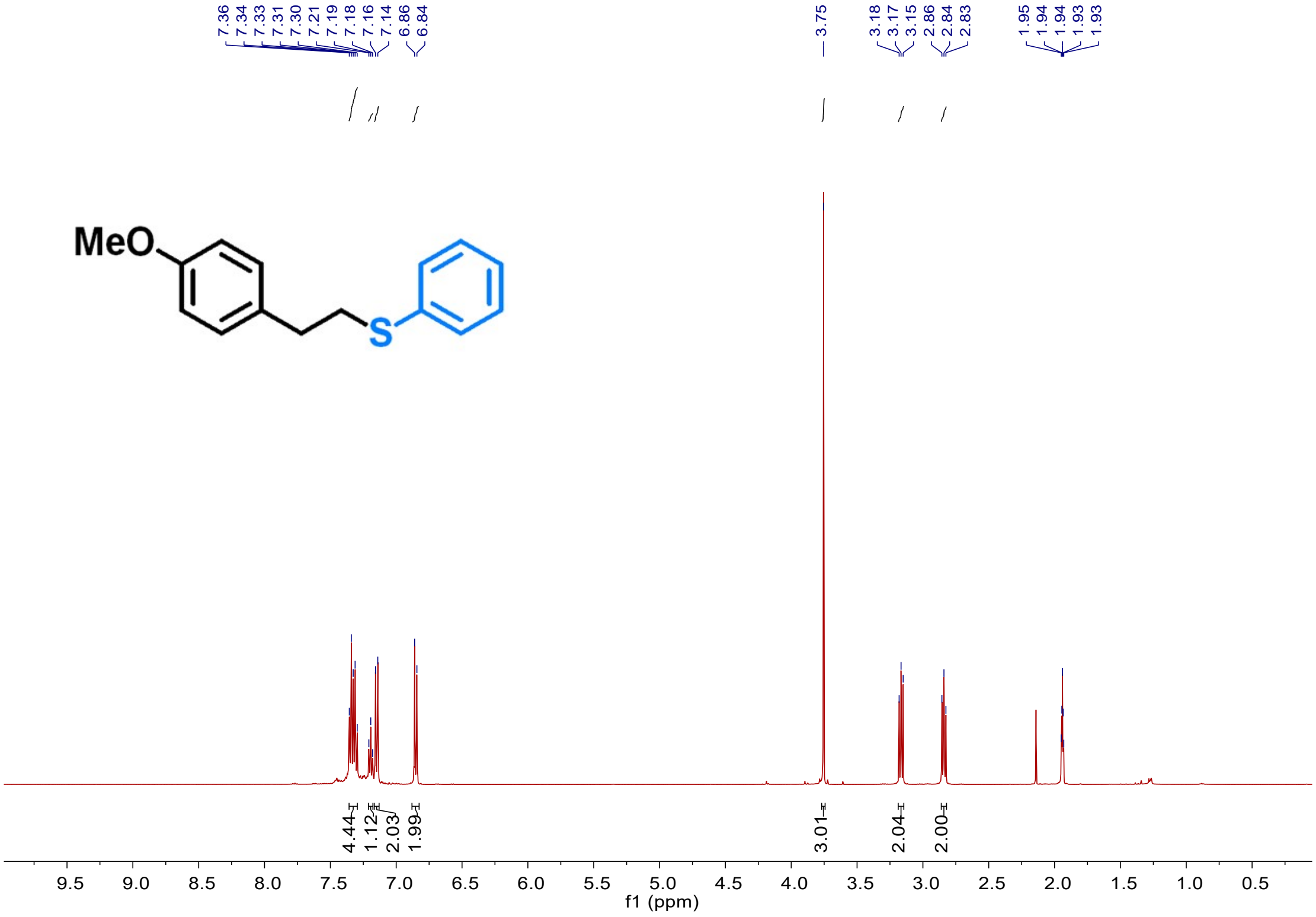

Figure S116. ^{1}H NMR (500 MHz) spectra of E4 in CD_3CN at 298 K.

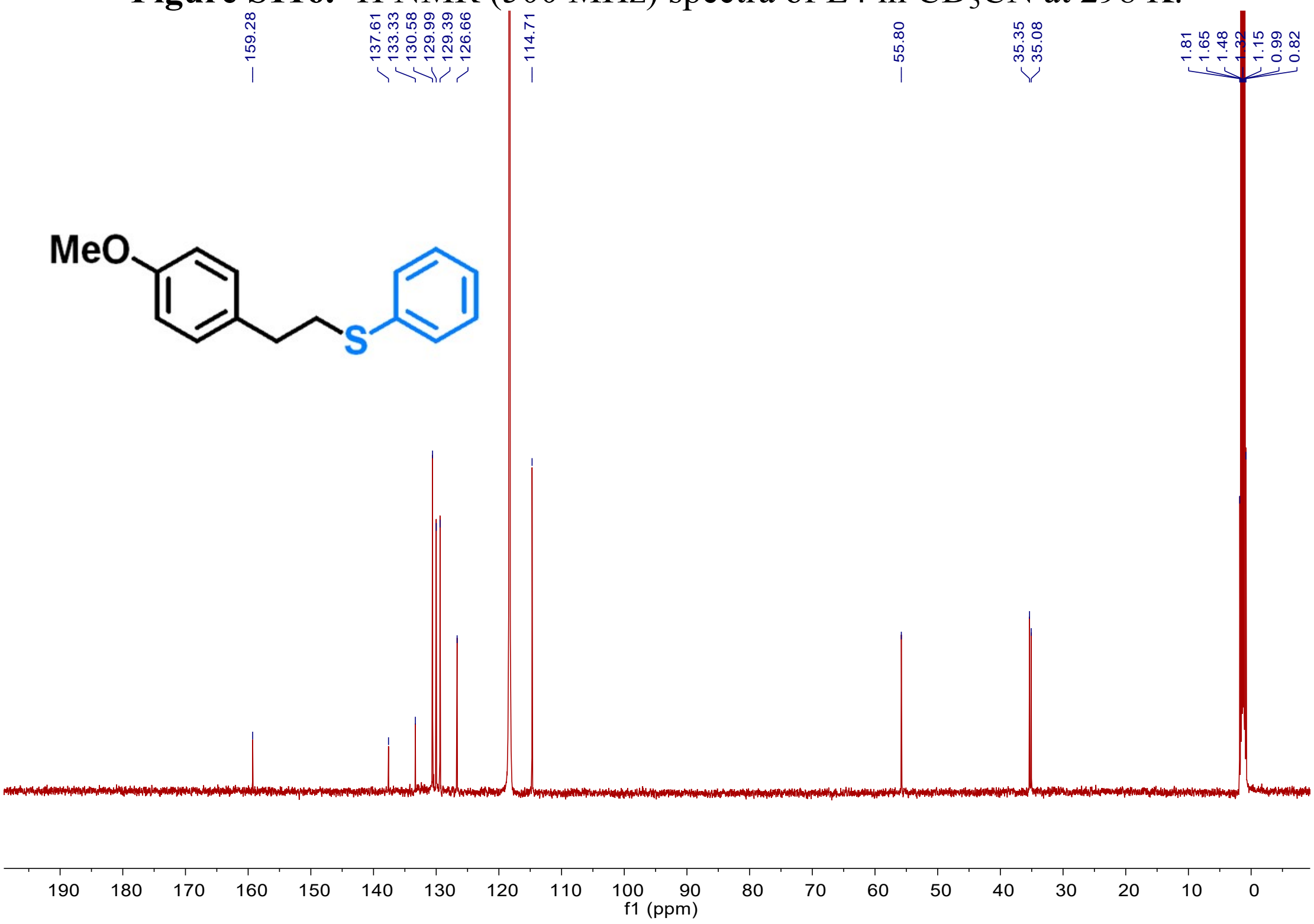

Figure S117. ^{13}C NMR (126 MHz) spectra of E4 in CD_3CN at 298 K.

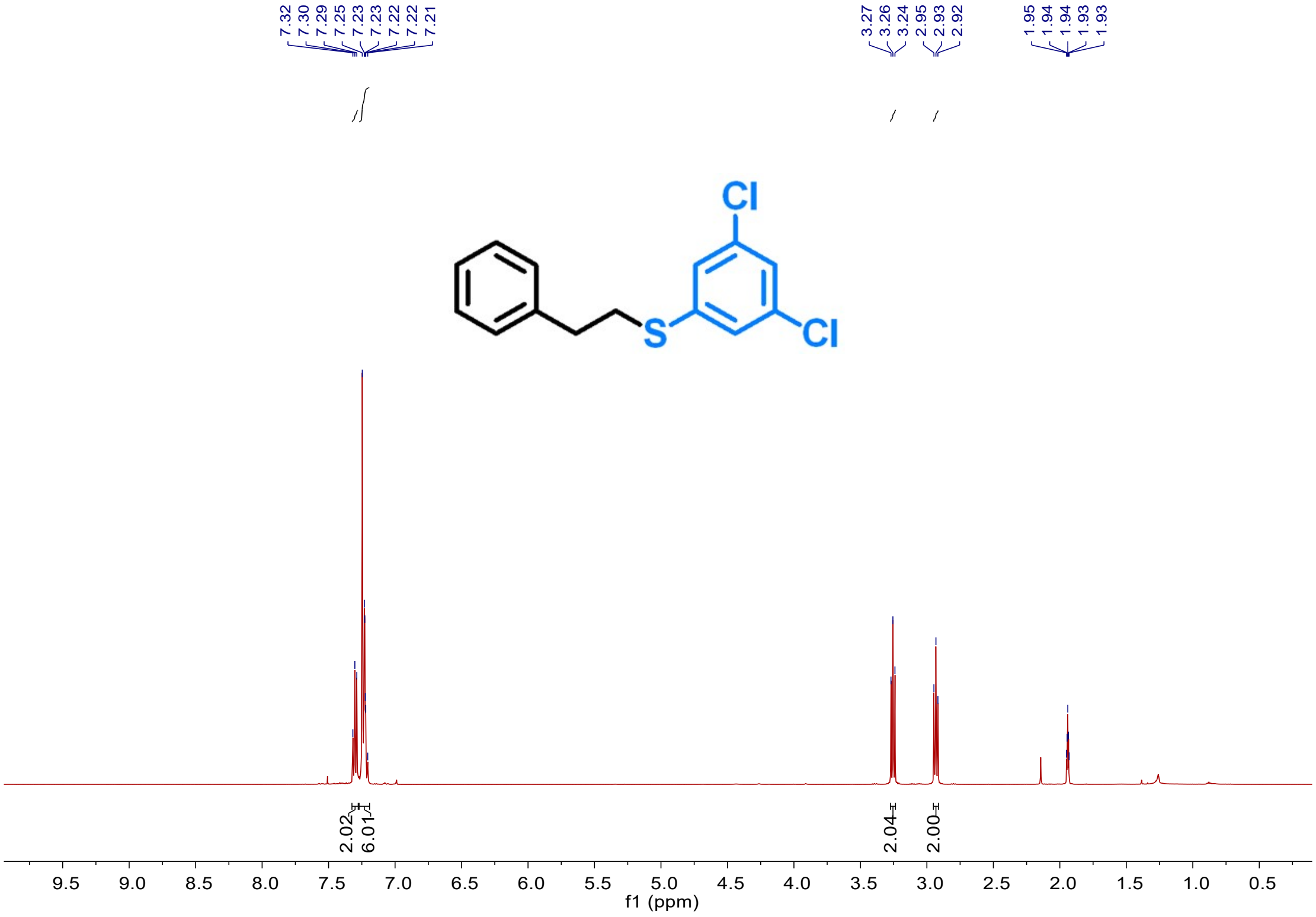

Figure S118. ^{1}H NMR (500 MHz) spectra of E5 in CD_3CN at 298 K.

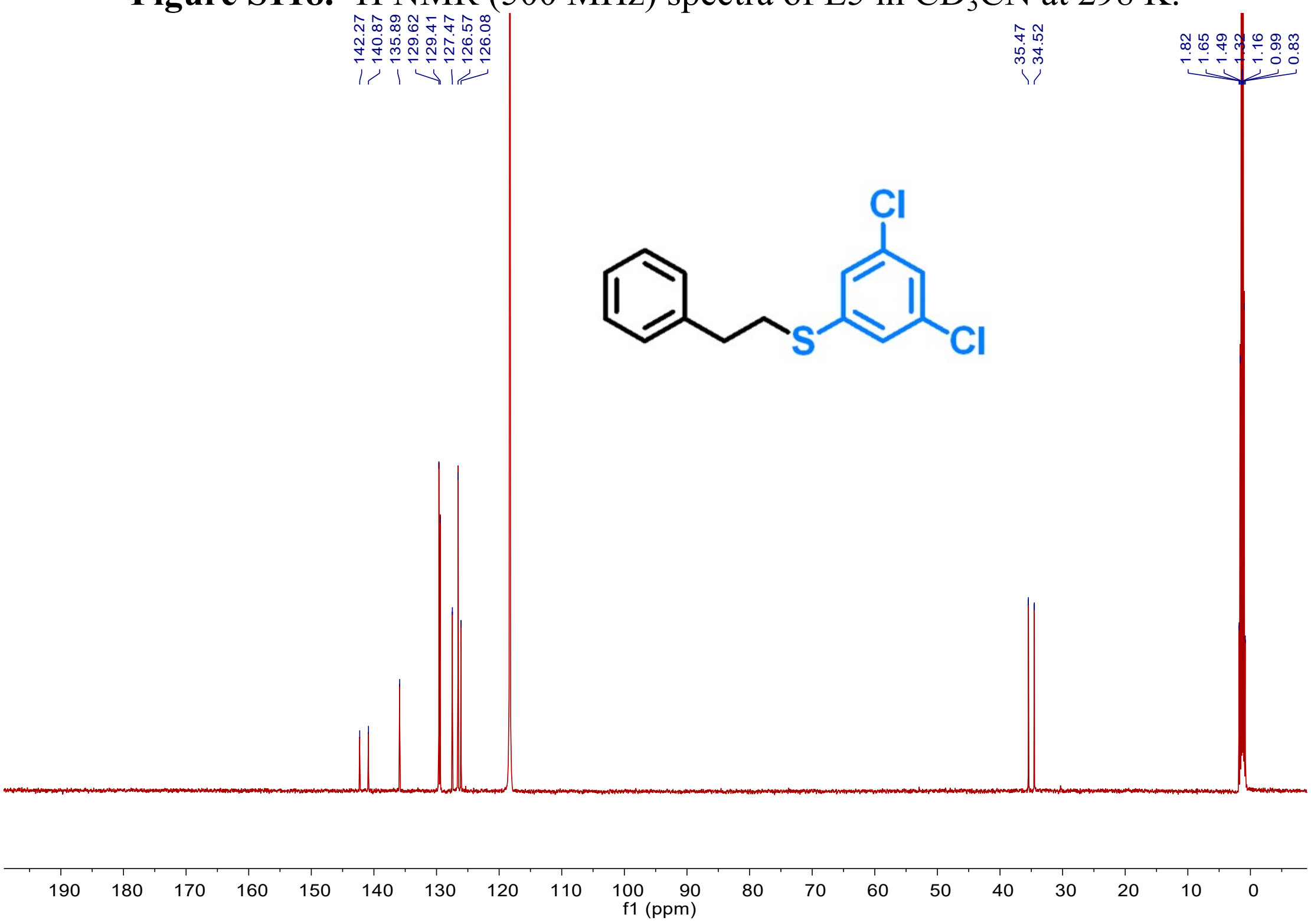

Figure S119. ^{13}C NMR (126 MHz) spectra of E5 in CD_3CN at 298 K.

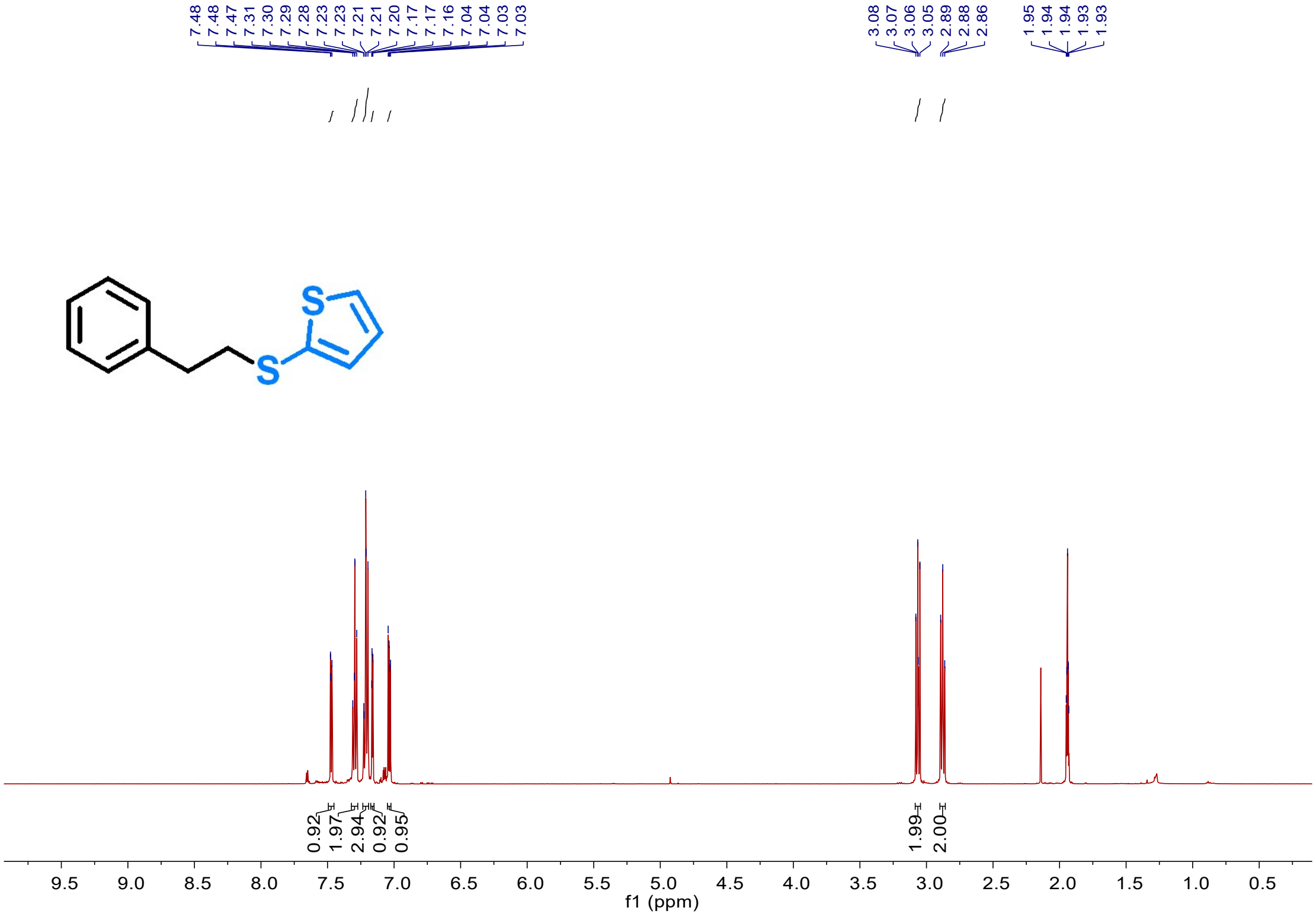

Figure S120. ^{1}H NMR (500 MHz) spectra of E6 in CD_3CN at 298 K.

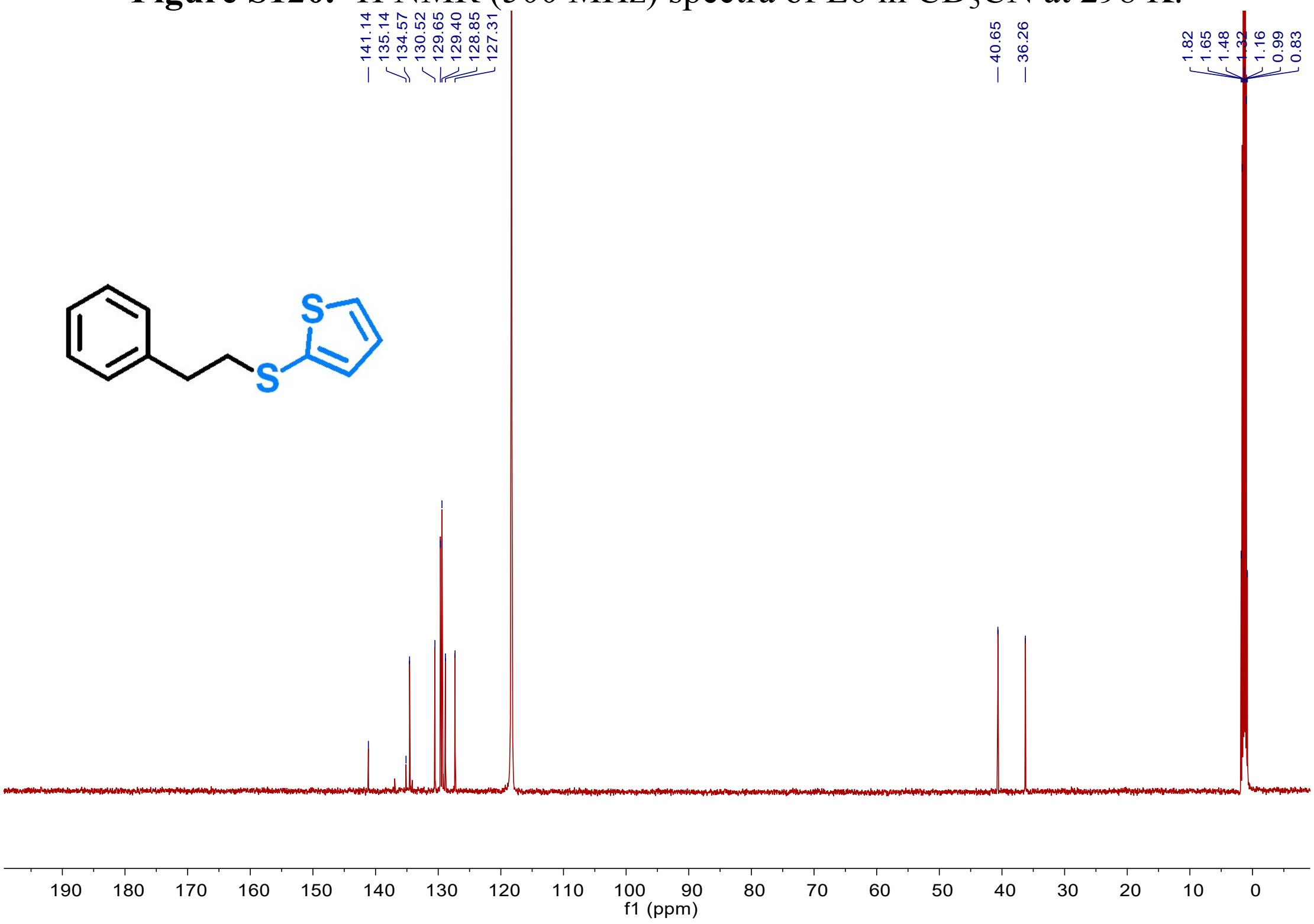

Figure S121. ^{13}C NMR (126 MHz) spectra of E6 in CD_3CN at 298 K.

References

1. M. H. Aukland, M. Šiaučiulis, A. West, G. J. P. Perry and D. J. Procter, *Nat. Catal.* 2020, **3**, 163-169.
2. P. Xu, D. Zhao, F. Berger, A. Hamad, J. Rickmeier, R. Petzold, M. Kondratiuk, K. Bohdan and T. Ritter, *Angew. Chem. Int. Ed.* 2020, **59**, 1956-1960.
3. C. Mi, B.-B. Zhang, G. Zhang, A. Peng, Z.-X. Wang, Q. Shi and H. Huang, *Chem. Eur. J.* 2024, **30**, e202303857.
4. K. Kafuta, A. Korzun, M. Böhm, C. Golz and M. Alcarazo, *Angew. Chem. Int. Ed.* 2020, **59**, 1950-1955.
5. M. Kawamura, E. Tsurumaki and S. Toyota, *Synthesis* 2018, **50**, 134-138.
6. H.-H. Hseuh, M.-Y. Hsu, T.-L. Wu and R.-S. Liu, *J. Org. Chem.* 2009, **74**, 8448-8451.
7. L. Ma, S. Wang, Y. Li, Q. Shi, W. Xie, H. Chen, X. Wang, W. Zhu, L. Jiang, R. Chen, Q. Peng and H. Huang, *CCS Chem.* 2022, **4**, 3669-3676.
8. Q. Shi, X. Shi, C. Feng, Y. Wu, N. Zheng, J. Liu, X. Wu, H. Chen, A. Peng, J. Li, L. Jiang, H. Fu, Z. Xie, S. R. Marder, S. B. Blakey and H. Huang, *Angew. Chem. Int. Ed.* 2021, **60**, 2924-2928.
9. F. Ye, F. Berger, H. Jia, J. Ford, A. Wortman, J. Börgel, C. Genicot and T. Ritter, *Angew. Chem. Int. Ed.* 2019, **58**, 14615-14619.
10. G. Shi, C. Shao, S. Pan, J. Yu and Y. Zhang, *Org. Lett.* 2015, **17**, 38-41.
11. X. Lin , C. Hou , H. Li and Z. Weng, *Chem. Eur. J.* 2016, **22**, 2075-2084.
12. K. Iwanaga, J. Kobayashi and T. Kawashima, *Tetrahedron* 2007, **63**, 10127-10132.
13. K. Natte, R. V. Jagadeesh, L. He, J. Rabeah, J. Chen, C. Taeschler, S. Ellinger, F. Zaragoza, H. Neumann, A. Brückner and M. Beller, *Angew. Chem. Int. Ed.* 2016, **55**, 2782-2786.
14. R. Jin, K. Yuan, E. Chatelain, J.-F. Soulé and H. Doucet, *Adv. Synth. Catal.* 2014, **356**, 3831-3841.
15. H. Sheng, Q. Liu, B.-B. Zhang, Z.-X. Wang and X.-Y. Chen, *Angew. Chem. Int. Ed.* 2023, **62**, e202218468.
16. E. Kocaoğlu, M. A. Karaman, H. Tokgöz and O. Talaz, *ACS Omega* 2017, **2**, 5000-5004.
17. S. K. Bose and T. B. Marder, *Org. Lett.* 2014, **16**, 4562-4565.
18. W. Liu, X. Yang, Y. Gao and C.-J. Li, *J. Am. Chem. Soc.* 2017, **139**, 8621-8627.
19. S. Kubosaki, H. Takeuchi, Y. Iwata, Y. Tanaka, K. Osaka, M. Yamawaki, T. Morita and Y. Yoshimi, *J. Org. Chem.* 2020, **85**, 5362-5369.
20. E. D. Slack and T. J. Colacot, *Org. Lett.* 2021, **23**, 1561-1565.
21. Q. Wang, X. Hao, K. Jin, R. Zhang, C. Duan and Y. Li, *Org. Biomol. Chem.* 2022, **20**, 4427-4430.
22. W. K. Chow, O. Y. Yuen, C. M. So, W. T. Wong and F. Y. Kwong, *J. Org. Chem.* 2012, **77**, 3543-3548.
23. J. A. Fernández-Salas, A. P. Pulis and D. J. Procter, *Chem. Commun.* 2016, **52**, 12364-12367.
24. Z.-J. Zheng, C. Jiang, P.-C. Shao, W.-F. Liu, T.-T. Zhao, P.-F. Xu and H. Wei, *Chem. Commun.* 2019, **55**, 1907-1910.
25. H.-J. Xu, Y.-Q. Zhao, T. Feng and Y.-S. Feng, *J. Org. Chem.* 2012, **77**, 2878-2884.
26. M. J. Cabrera-Afonso, A. Granados and G. A. Molander, *Angew. Chem. Int. Ed.* 2022, **61**, e202202706.
27. F. Tan, X.-D. Su, Q.-S. Zhang, B.-B. Zhang, L. He, Q. Liu and X.-Y. Chen, *Org. Chem. Front.* 2024, **11**, 654-660.
28. E. L. Tyson, M. S. Ament and T. P. Yoon, *J. Org. Chem.* 2013, **78**, 2046-2050.
29. N. W. J. Ang and L. Ackermann, *Chem. Eur. J.* 2021, **27**, 4883-4887.

30. X. Fan, D. Zhang, X. Xiu, B. Xu, Y. Yuan, F. Chen and P. Gao, *Beilstein J. Org. Chem.* 2024, **20**, 257-263.
